# Supplementary material for: Real-Time Musculoskeletal Kinematics and Dynamics Analysis Using Marker- and IMU-Based Solutions in Rehabilitation
Source: Sensors (Basel). 2021 Mar 5;21(5):1804. doi: 10.3390/s21051804 (PMC7961635; doi:10.3390/s21051804)
Supplement: Supplementary file 1 [file sensors-21-01804-s001.zip › supplementary_filtering.pdf]

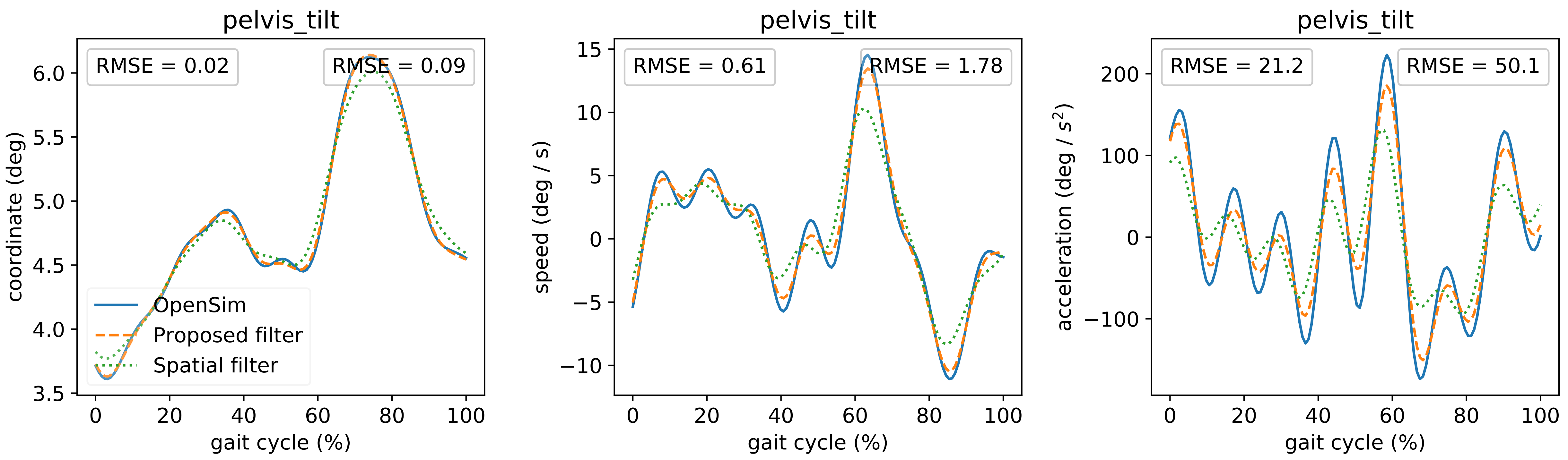
pelvis_tilt
RMSE = 0.02
RMSE = 0.09
coordinate (deg)
gait cycle (%)
OpenSim
Proposed filter
Spatial filter
pelvis_tilt
RMSE = 0.61
RMSE = 1.78
speed (deg / s)
gait cycle (%)
pelvis_tilt
RMSE = 21.2
RMSE = 50.1
acceleration (deg / s^2)
gait cycle (%)

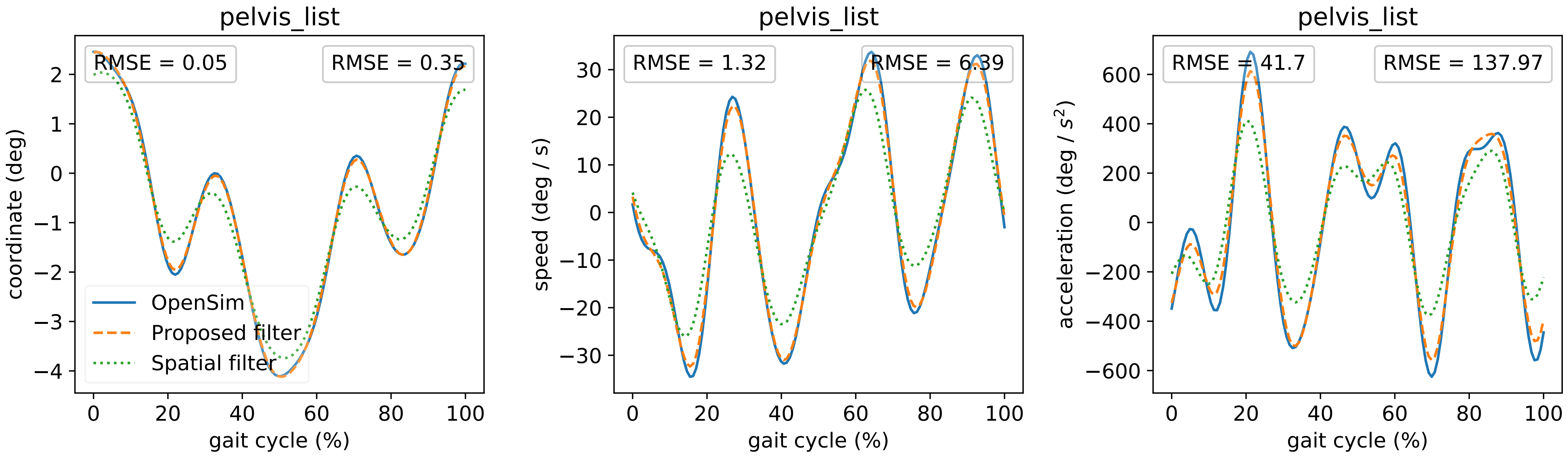
pelvis_list
RMSE = 0.05
RMSE = 0.35
coordinate (deg)
gait cycle (%)
OpenSim
Proposed filter
Spatial filter
pelvis_list
RMSE = 1.32
RMSE = 6.39
speed (deg / s)
gait cycle (%)
pelvis_list
RMSE = 41.7
RMSE = 137.97
acceleration (deg / s^2)
gait cycle (%)

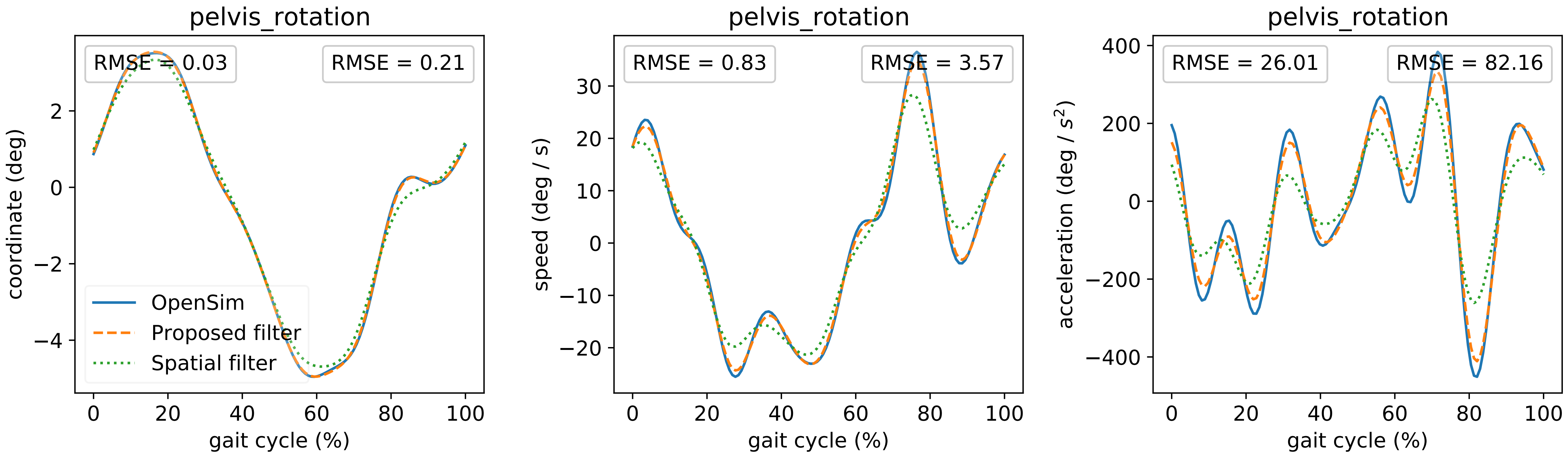

pelvis_rotation
RMSE = 0.03
RMSE = 0.21
coordinate (deg)
gait cycle (%)
OpenSim
Proposed filter
Spatial filter
pelvis_rotation
RMSE = 0.83
RMSE = 3.57
speed (deg / s)
gait cycle (%)
pelvis_rotation
RMSE = 26.01
RMSE = 82.16
acceleration (deg / s^2)
gait cycle (%)

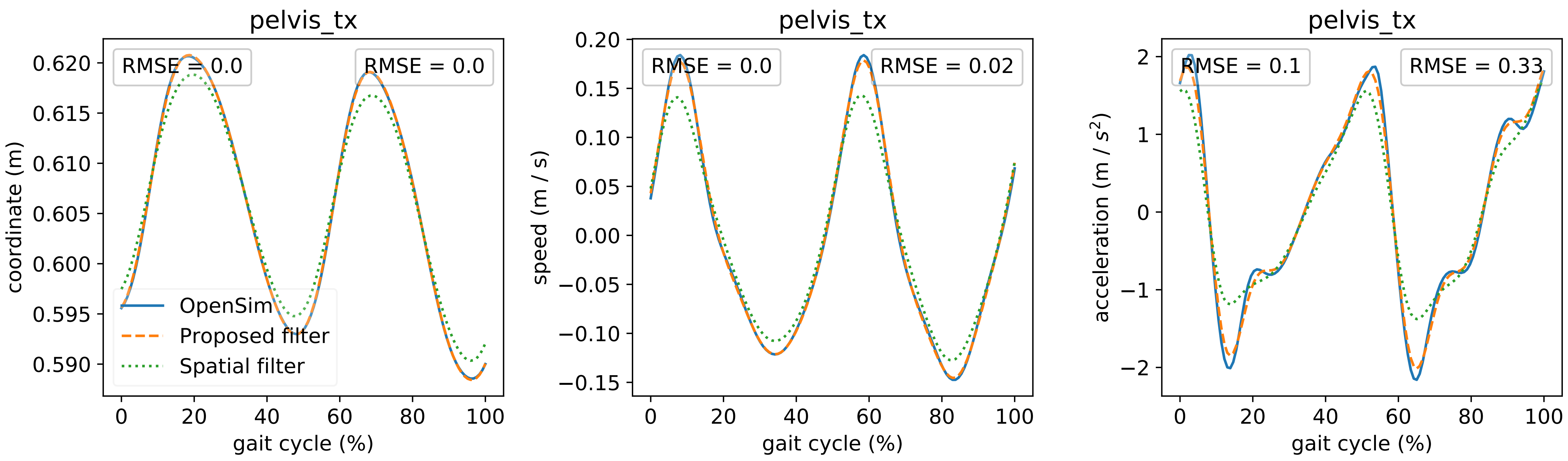

pelvis_tx
RMSE = 0.0
RMSE = 0.0
coordinate (m)
0.620
0.615
0.610
0.605
0.600
0.595
0.590
0
20
40
60
80
100
gait cycle (%)
OpenSim
Proposed filter
Spatial filter
pelvis_tx
RMSE = 0.0
RMSE = 0.02
speed (m / s)
0.20
0.15
0.10
0.05
0.00
−0.05
−0.10
−0.15
0
20
40
60
80
100
gait cycle (%)
pelvis_tx
RMSE = 0.1
RMSE = 0.33
acceleration (m / s^2)
2
1
0
−1
−2
0
20
40
60
80
100
gait cycle (%)

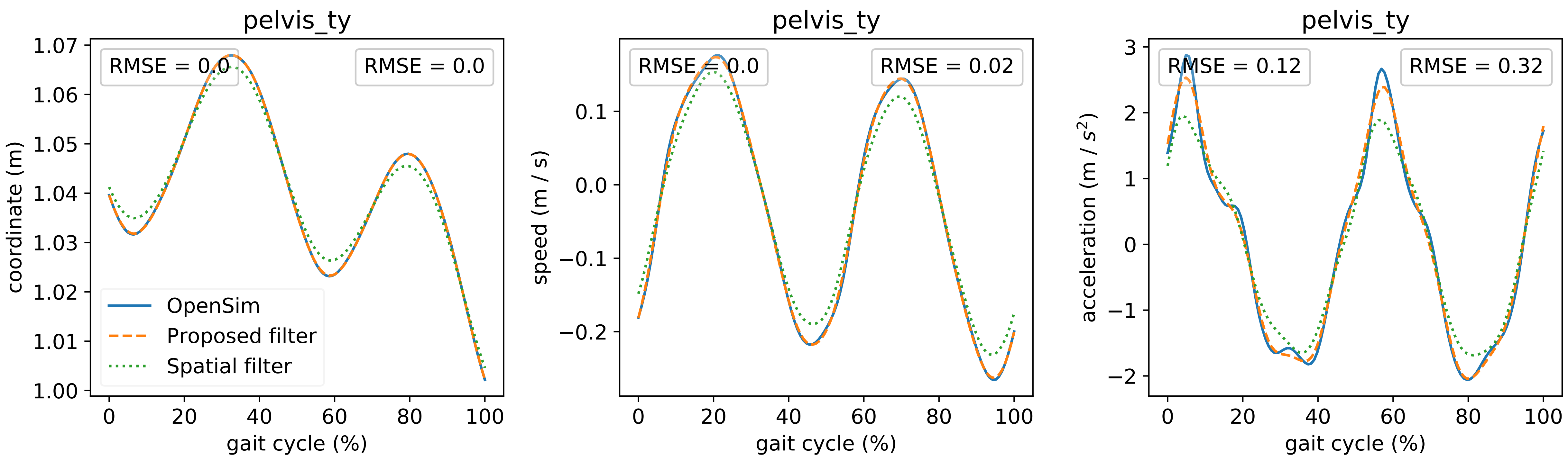

pelvis_ty
RMSE = 0.0
RMSE = 0.0
coordinate (m)
1.07
1.06
1.05
1.04
1.03
1.02
1.01
1.00
OpenSim
Proposed filter
Spatial filter
0
20
40
60
80
100
gait cycle (%)
pelvis_ty
RMSE = 0.0
RMSE = 0.02
speed (m / s)
0.1
0.0
−0.1
−0.2
0
20
40
60
80
100
gait cycle (%)
pelvis_ty
RMSE = 0.12
RMSE = 0.32
acceleration (m / s^2)
3
2
1
0
−1
−2
0
20
40
60
80
100
gait cycle (%)

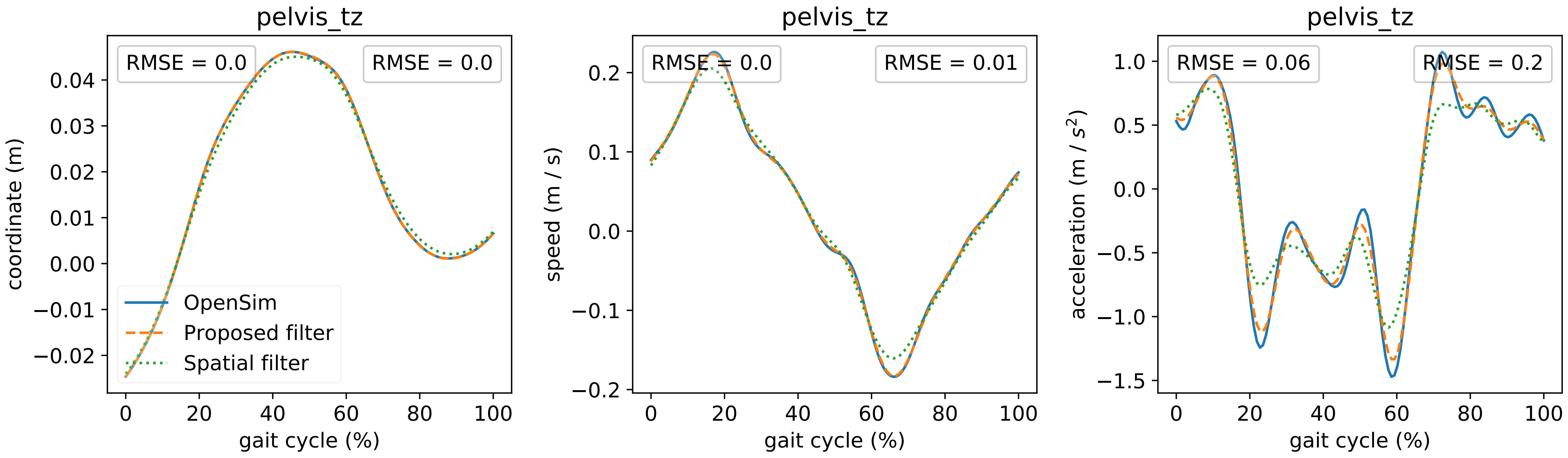

pelvis_tz
RMSE = 0.0
RMSE = 0.0
coordinate (m)
gait cycle (%)
OpenSim
Proposed filter
Spatial filter
pelvis_tz
RMSE = 0.0
RMSE = 0.01
speed (m / s)
gait cycle (%)
pelvis_tz
RMSE = 0.06
RMSE = 0.2
acceleration (m / s^2)
gait cycle (%)

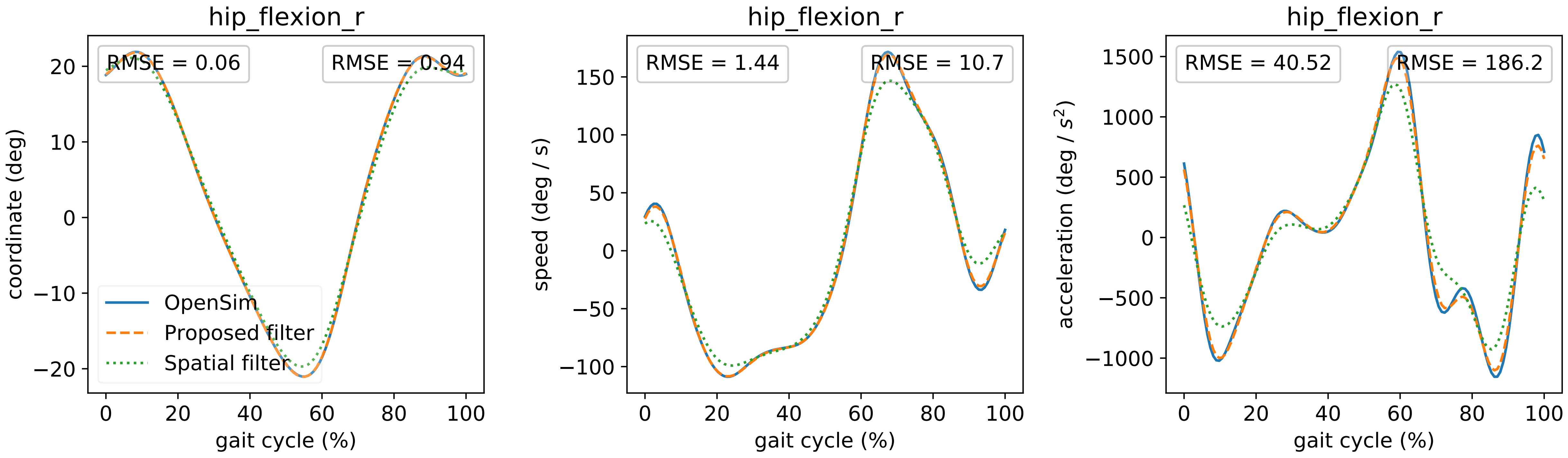

hip_flexion_r
RMSE = 0.06
RMSE = 0.94
coordinate (deg)
gait cycle (%)
OpenSim
Proposed filter
Spatial filter
hip_flexion_r
RMSE = 1.44
RMSE = 10.7
speed (deg / s)
gait cycle (%)
hip_flexion_r
RMSE = 40.52
RMSE = 186.2
acceleration (deg / s^2)
gait cycle (%)

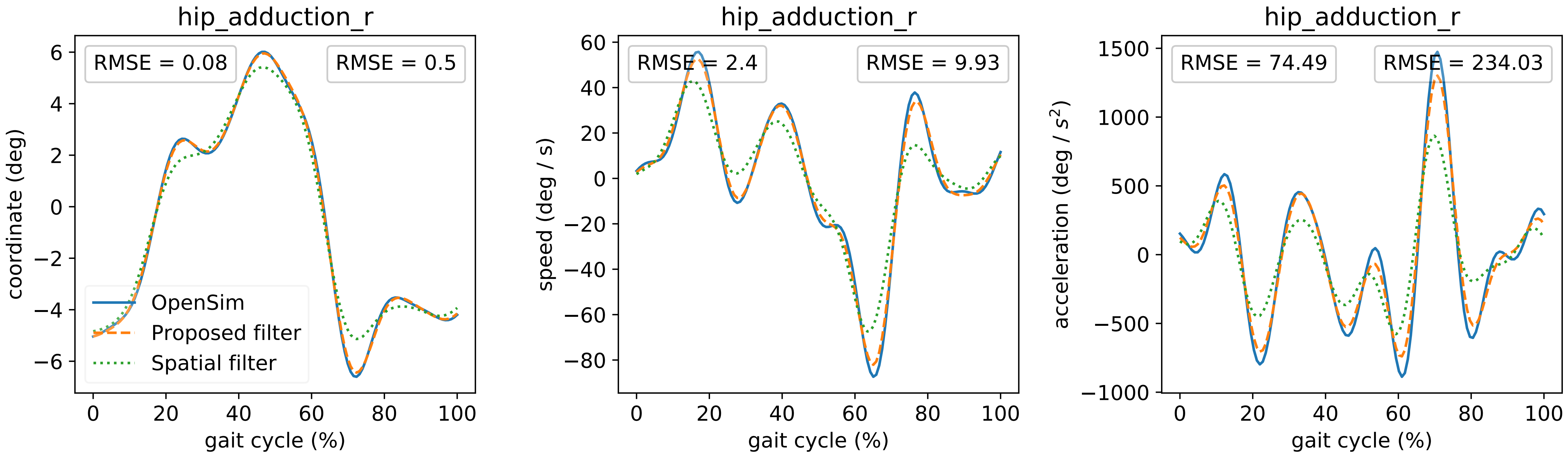

hip_adduction_r
RMSE = 0.08
RMSE = 0.5
coordinate (deg)
gait cycle (%)
OpenSim
Proposed filter
Spatial filter
hip_adduction_r
RMSE = 2.4
RMSE = 9.93
speed (deg / s)
gait cycle (%)
hip_adduction_r
RMSE = 74.49
RMSE = 234.03
acceleration (deg / s^2)
gait cycle (%)

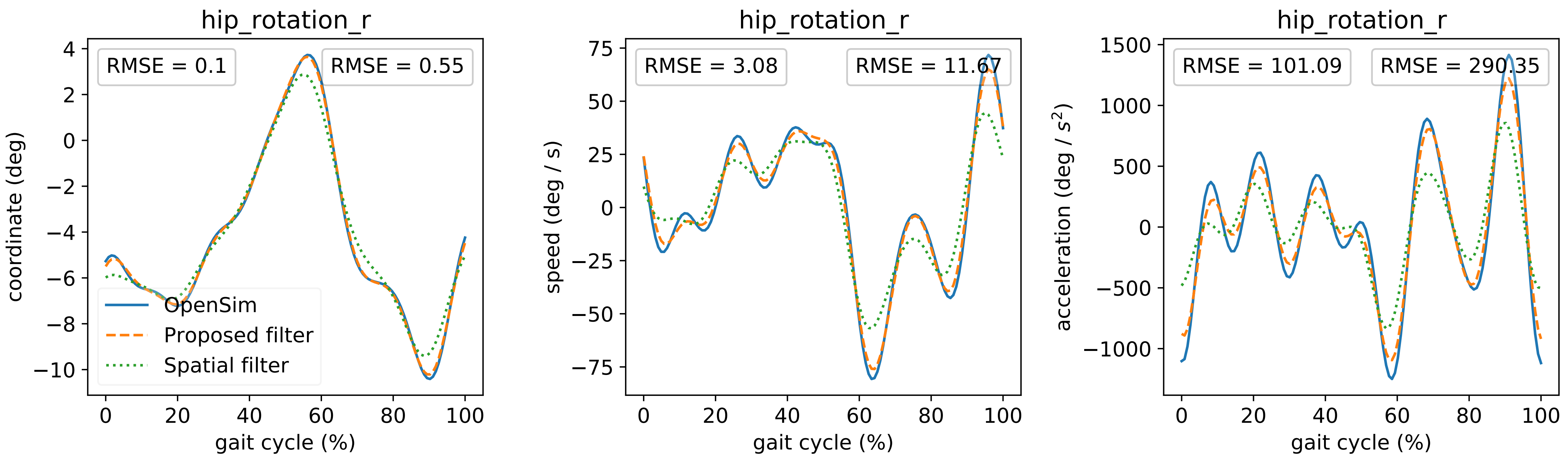
hip_rotation_r
RMSE = 0.1
RMSE = 0.55
coordinate (deg)
gait cycle (%)
OpenSim
Proposed filter
Spatial filter
hip_rotation_r
RMSE = 3.08
RMSE = 11.67
speed (deg / s)
gait cycle (%)
hip_rotation_r
RMSE = 101.09
RMSE = 290.35
acceleration (deg / s^2)
gait cycle (%)

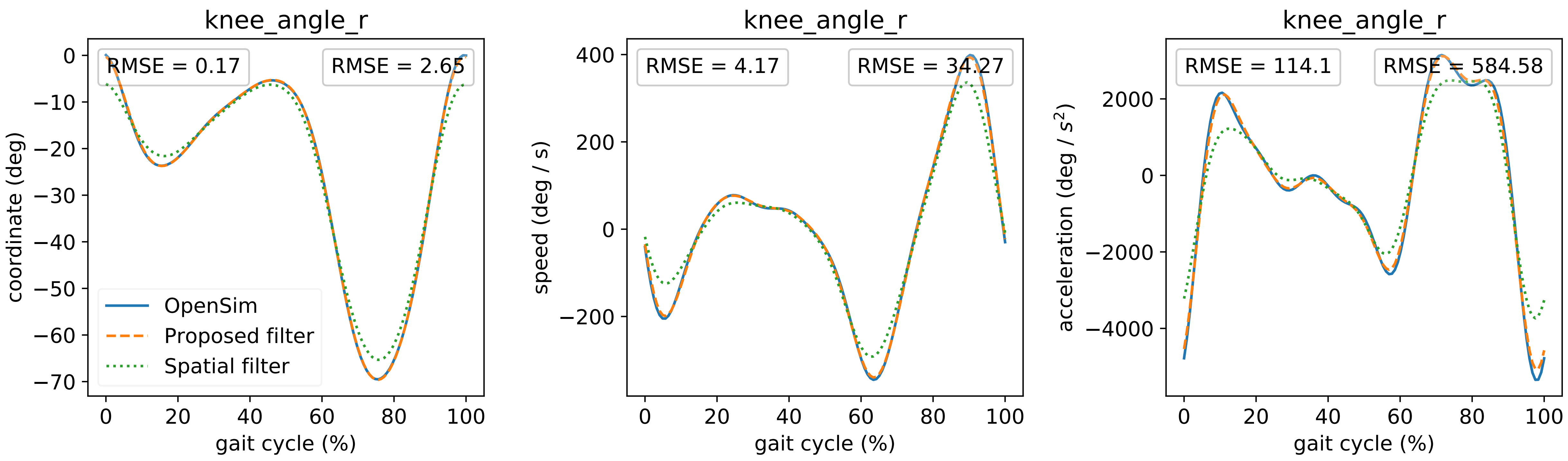

knee_angle_r
RMSE = 0.17
RMSE = 2.65
coordinate (deg)
gait cycle (%)
OpenSim
Proposed filter
Spatial filter
knee_angle_r
RMSE = 4.17
RMSE = 34.27
speed (deg / s)
gait cycle (%)
knee_angle_r
RMSE = 114.1
RMSE = 584.58
acceleration (deg / s²)
gait cycle (%)

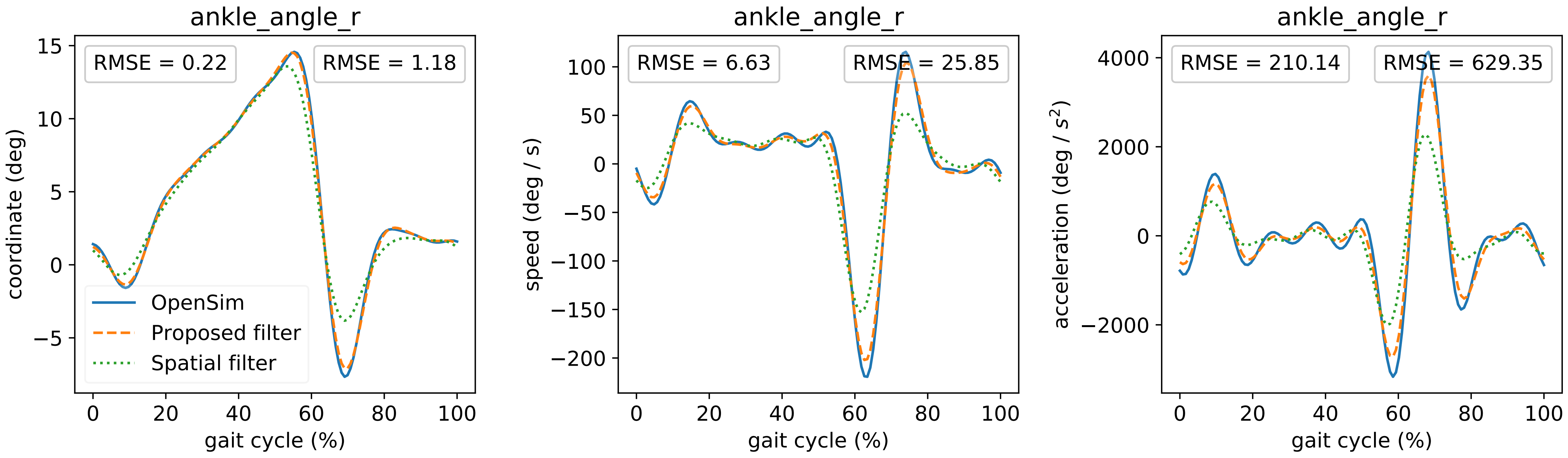
ankle_angle_r
RMSE = 0.22
RMSE = 1.18
coordinate (deg)
gait cycle (%)
OpenSim
Proposed filter
Spatial filter
ankle_angle_r
RMSE = 6.63
RMSE = 25.85
speed (deg / s)
gait cycle (%)
ankle_angle_r
RMSE = 210.14
RMSE = 629.35
acceleration (deg / s^2)
gait cycle (%)

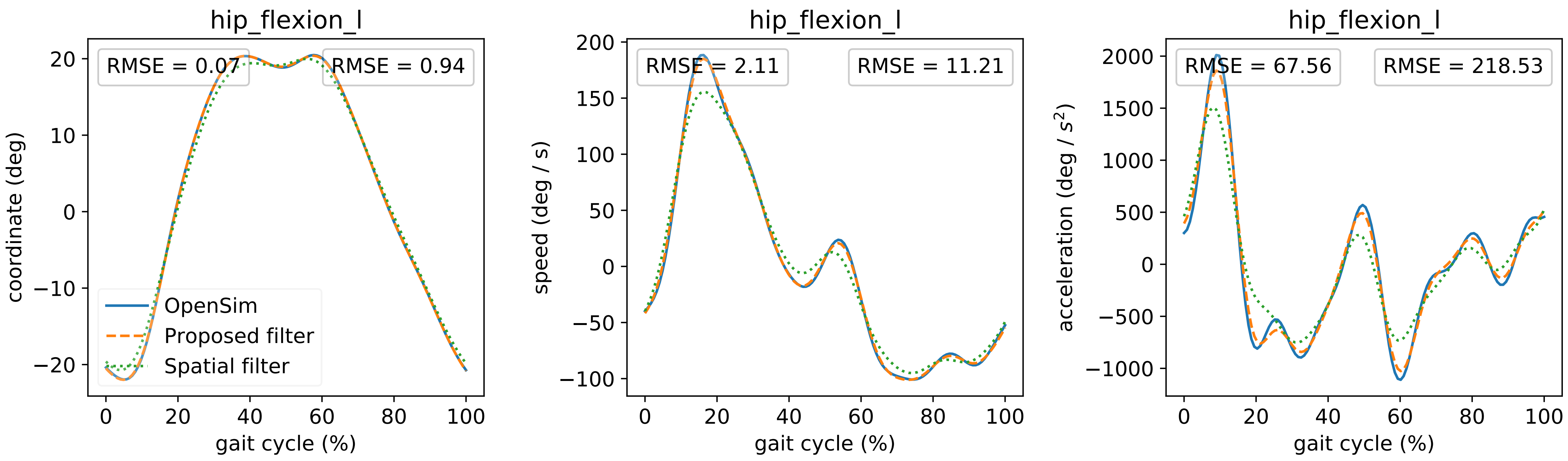

hip_flexion_l
RMSE = 0.07
RMSE = 0.94
coordinate (deg)
20
10
0
−10
−20
0
20
40
60
80
100
gait cycle (%)
OpenSim
Proposed filter
Spatial filter
hip_flexion_l
RMSE = 2.11
RMSE = 11.21
speed (deg / s)
200
150
100
50
0
−50
−100
gait cycle (%)
hip_flexion_l
RMSE = 67.56
RMSE = 218.53
acceleration (deg / s^2)
2000
1500
1000
500
0
−500
−1000
gait cycle (%)

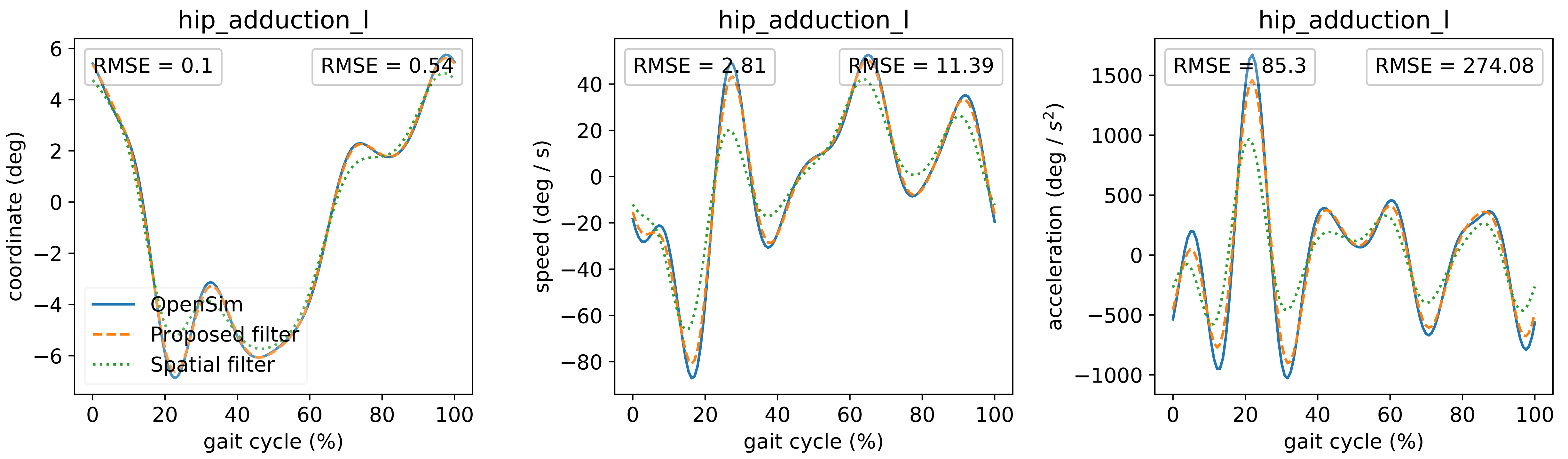

hip_adduction_l
RMSE = 0.1
RMSE = 0.54
coordinate (deg)
OpenSim
Proposed filter
Spatial filter
gait cycle (%)
hip_adduction_l
RMSE = 2.81
RMSE = 11.39
speed (deg / s)
gait cycle (%)
hip_adduction_l
RMSE = 85.3
RMSE = 274.08
acceleration (deg / s^2)
gait cycle (%)

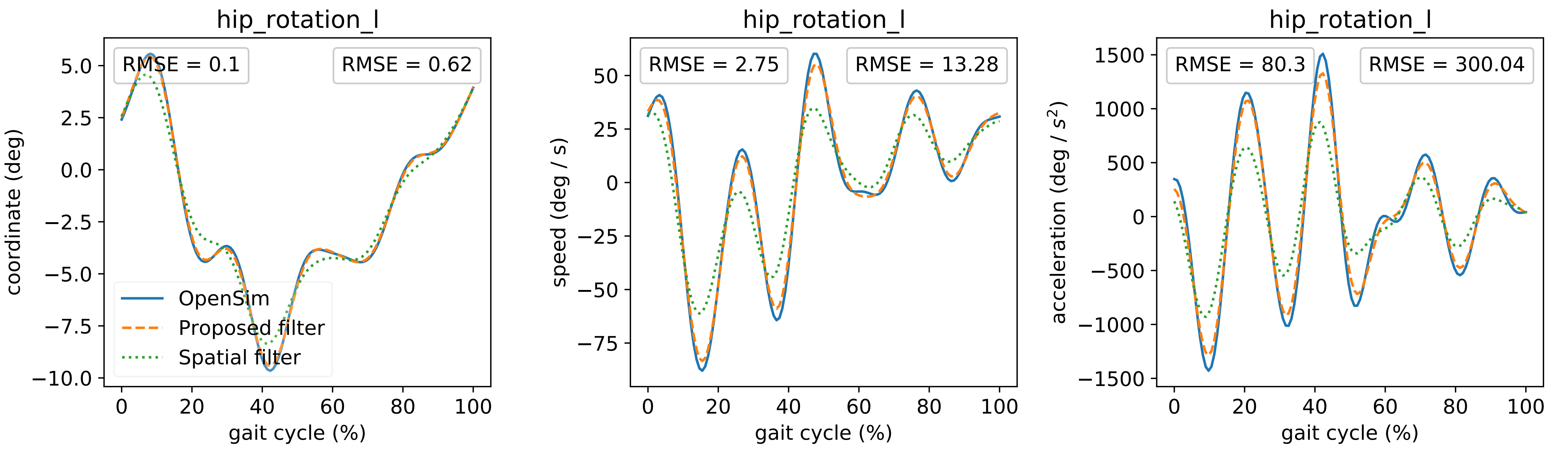
hip_rotation_l
RMSE = 0.1
RMSE = 0.62
coordinate (deg)
gait cycle (%)
OpenSim
Proposed filter
Spatial filter
hip_rotation_l
RMSE = 2.75
RMSE = 13.28
speed (deg / s)
gait cycle (%)
hip_rotation_l
RMSE = 80.3
RMSE = 300.04
acceleration (deg / s^2)
gait cycle (%)

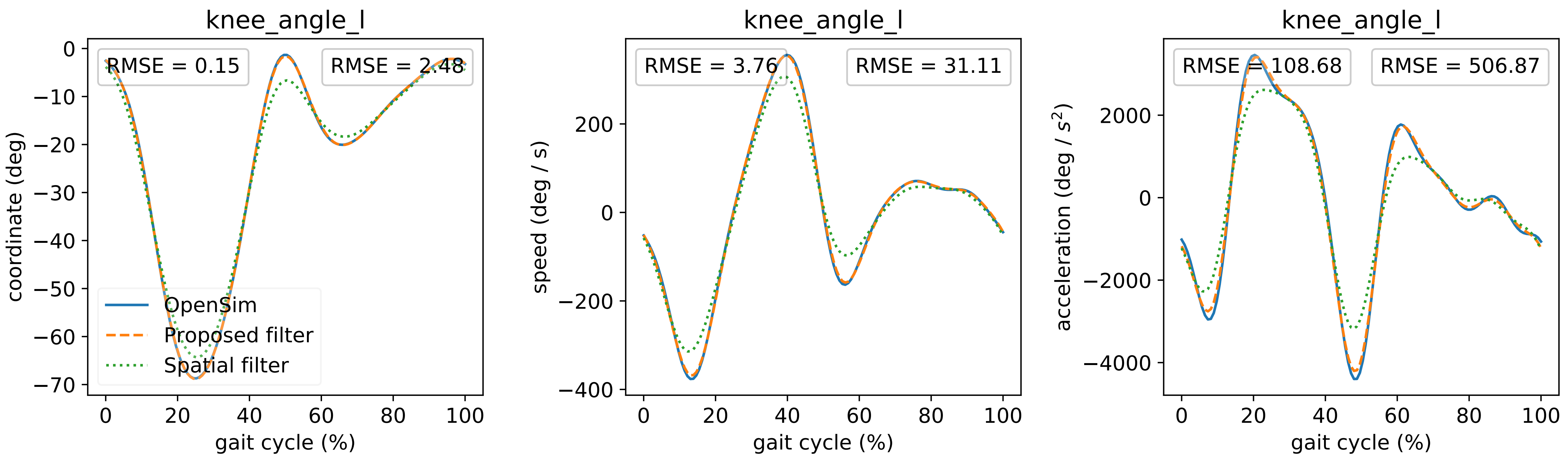
knee_angle_l
RMSE = 0.15
RMSE = 2.48
coordinate (deg)
gait cycle (%)
OpenSim
Proposed filter
Spatial filter
knee_angle_l
RMSE = 3.76
RMSE = 31.11
speed (deg / s)
gait cycle (%)
knee_angle_l
RMSE = 108.68
RMSE = 506.87
acceleration (deg / s^2)
gait cycle (%)

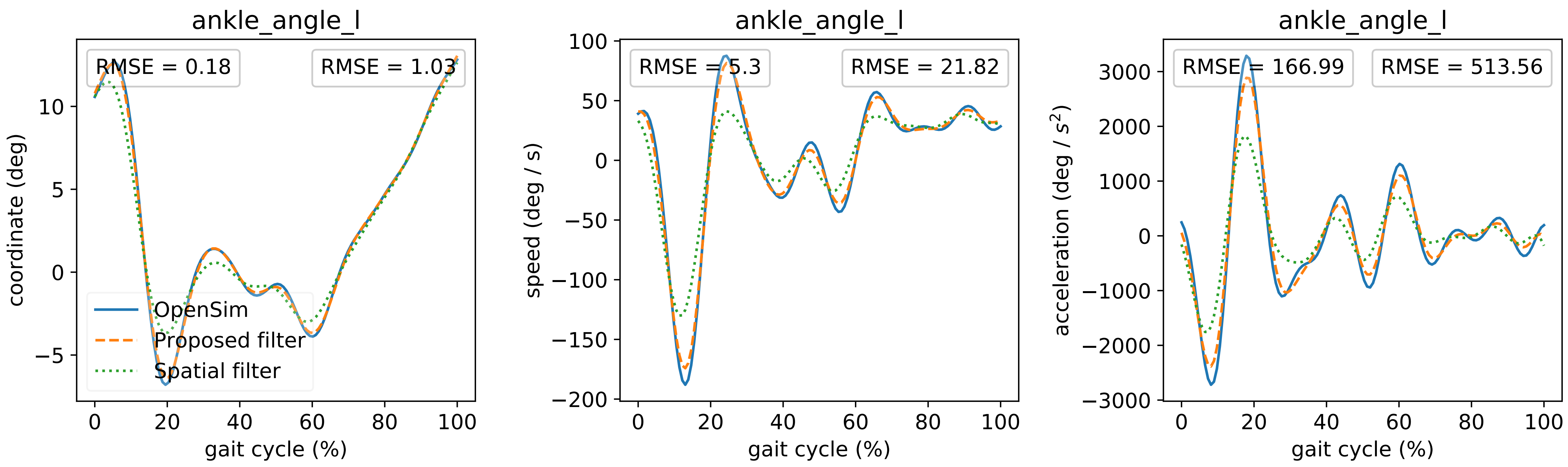
ankle_angle_l
RMSE = 0.18
RMSE = 1.03
coordinate (deg)
gait cycle (%)
OpenSim
Proposed filter
Spatial filter
ankle_angle_l
RMSE = 5.3
RMSE = 21.82
speed (deg / s)
gait cycle (%)
ankle_angle_l
RMSE = 166.99
RMSE = 513.56
acceleration (deg / s^2)
gait cycle (%)

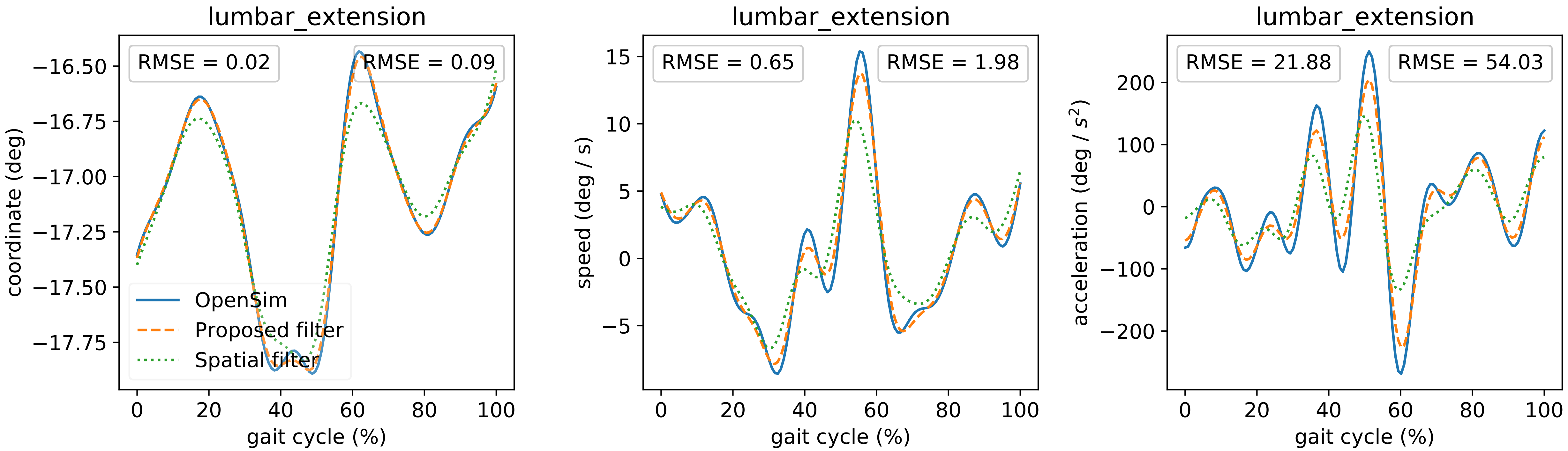

lumbar_extension
RMSE = 0.02
RMSE = 0.09
coordinate (deg)
gait cycle (%)
OpenSim
Proposed filter
Spatial filter
lumbar_extension
RMSE = 0.65
RMSE = 1.98
speed (deg / s)
gait cycle (%)
lumbar_extension
RMSE = 21.88
RMSE = 54.03
acceleration (deg / s^2)
gait cycle (%)

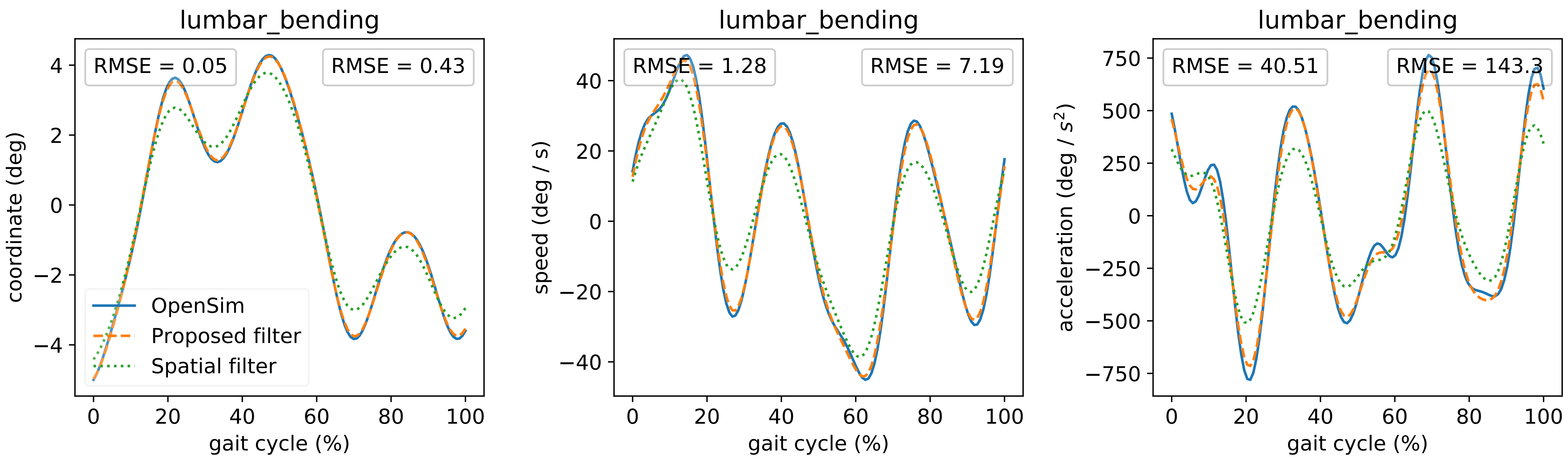
lumbar_bending
RMSE = 0.05
RMSE = 0.43
coordinate (deg)
gait cycle (%)
OpenSim
Proposed filter
Spatial filter
lumbar_bending
RMSE = 1.28
RMSE = 7.19
speed (deg / s)
gait cycle (%)
lumbar_bending
RMSE = 40.51
RMSE = 143.3
acceleration (deg / s^2)
gait cycle (%)

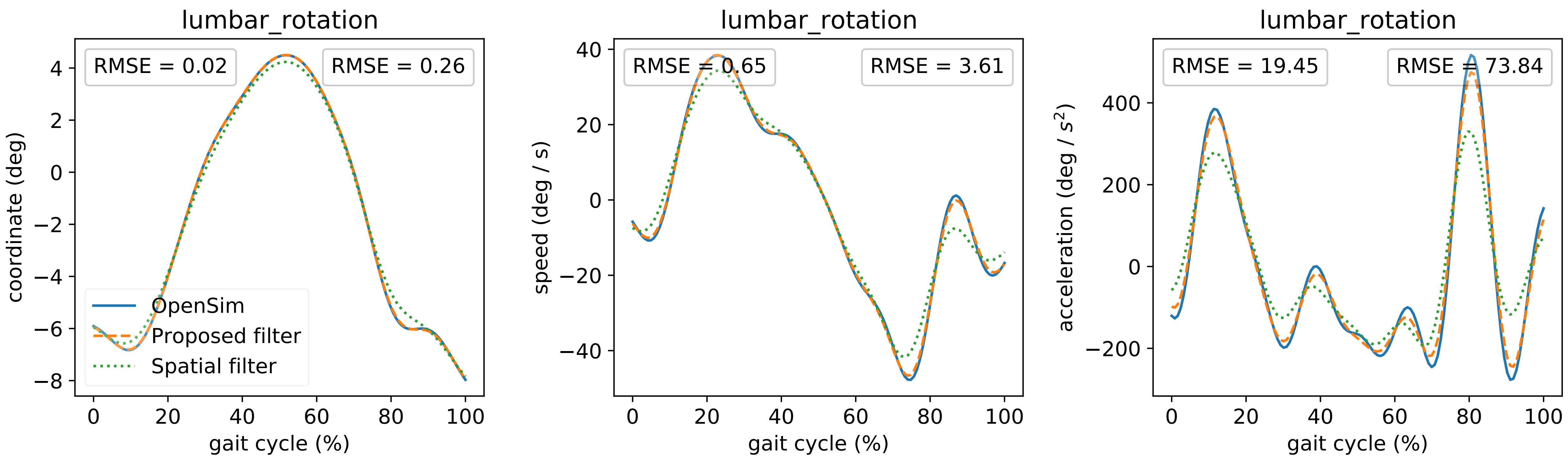

lumbar_rotation
RMSE = 0.02
RMSE = 0.26
coordinate (deg)
gait cycle (%)
OpenSim
Proposed filter
Spatial filter
lumbar_rotation
RMSE = 0.65
RMSE = 3.61
speed (deg / s)
gait cycle (%)
lumbar_rotation
RMSE = 19.45
RMSE = 73.84
acceleration (deg / s^2)
gait cycle (%)
