# Supplementary material for: Real-Time Musculoskeletal Kinematics and Dynamics Analysis Using Marker- and IMU-Based Solutions in Rehabilitation
Source: Sensors (Basel). 2021 Mar 5;21(5):1804. doi: 10.3390/s21051804 (PMC7961635; doi:10.3390/s21051804)
Supplement: Supplementary file 1 [file sensors-21-01804-s001.zip › supplementary_inverse_dynamics.pdf]

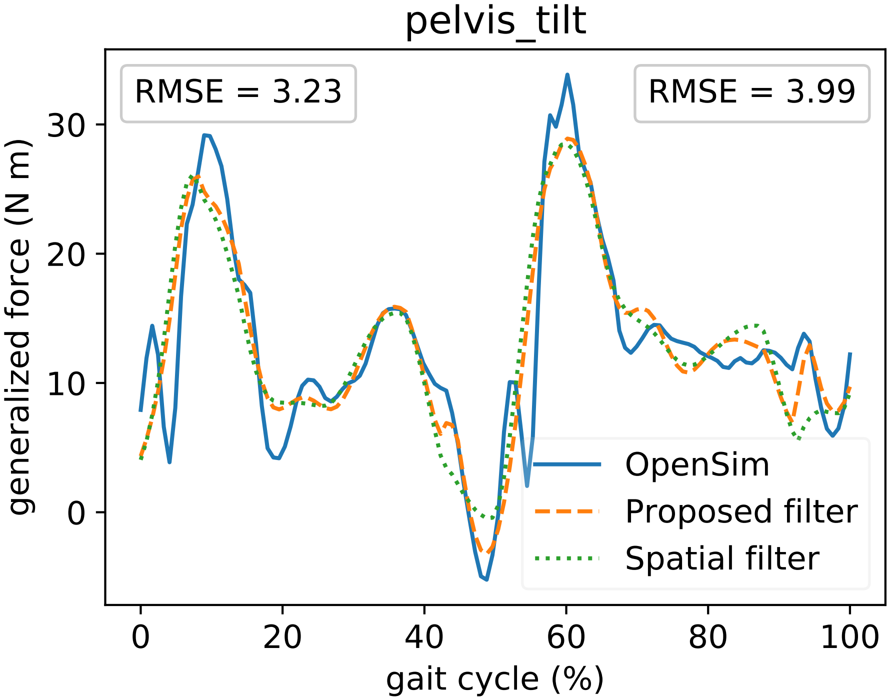

pelvis_tilt
RMSE = 3.23
RMSE = 3.99
generalized force (N m)
0
10
20
30
0
20
40
60
80
100
gait cycle (%)
OpenSim
Proposed filter
Spatial filter

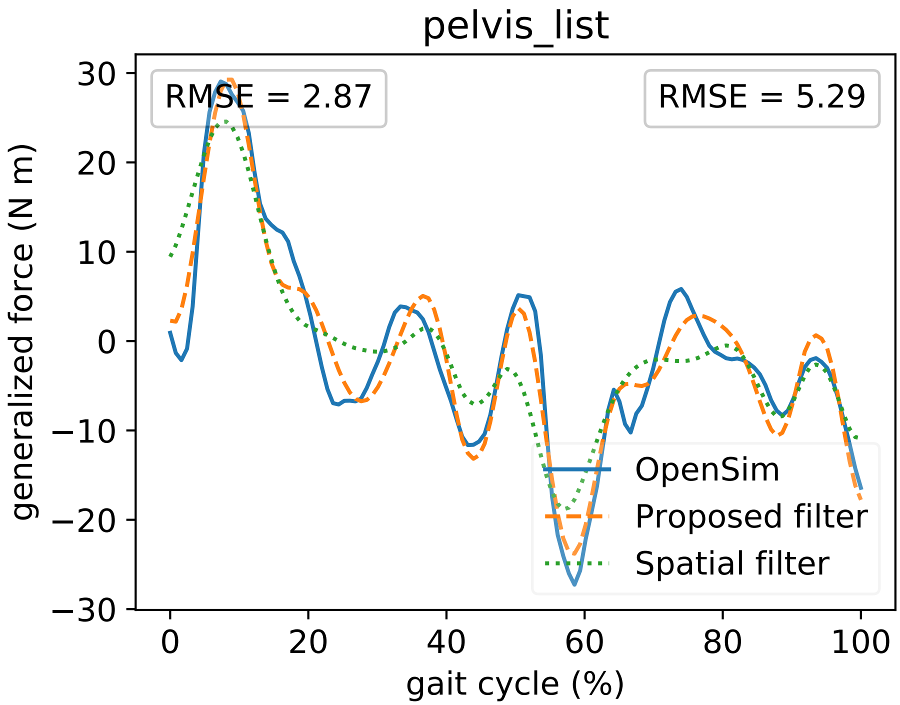
pelvis_list
RMSE = 2.87
RMSE = 5.29
generalized force (N m)
gait cycle (%)
OpenSim
Proposed filter
Spatial filter
30
20
10
0
−10
−20
−30
0
20
40
60
80
100

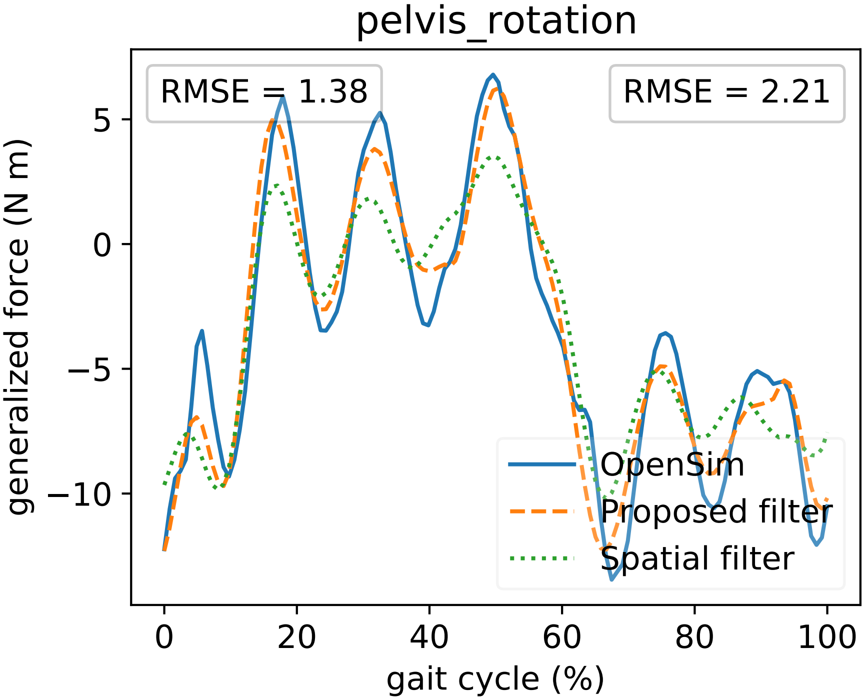
pelvis_rotation
RMSE = 1.38
RMSE = 2.21
generalized force (N m)
5
0
−5
−10
0
20
40
60
80
100
gait cycle (%)
OpenSim
Proposed filter
Spatial filter

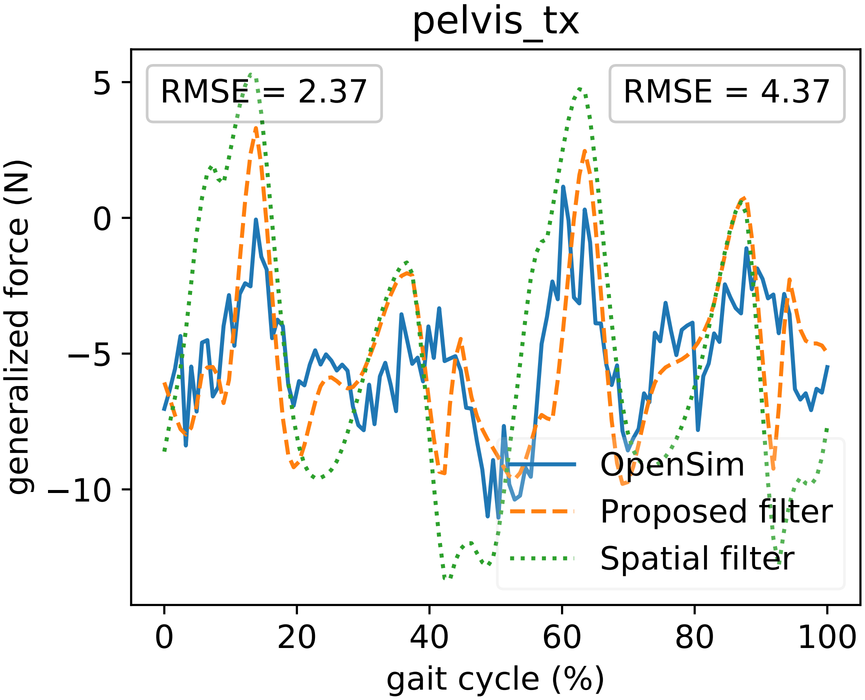
pelvis_tx
RMSE = 2.37
RMSE = 4.37
generalized force (N)
5
0
−5
−10
0
20
40
60
80
100
gait cycle (%)
OpenSim
Proposed filter
Spatial filter

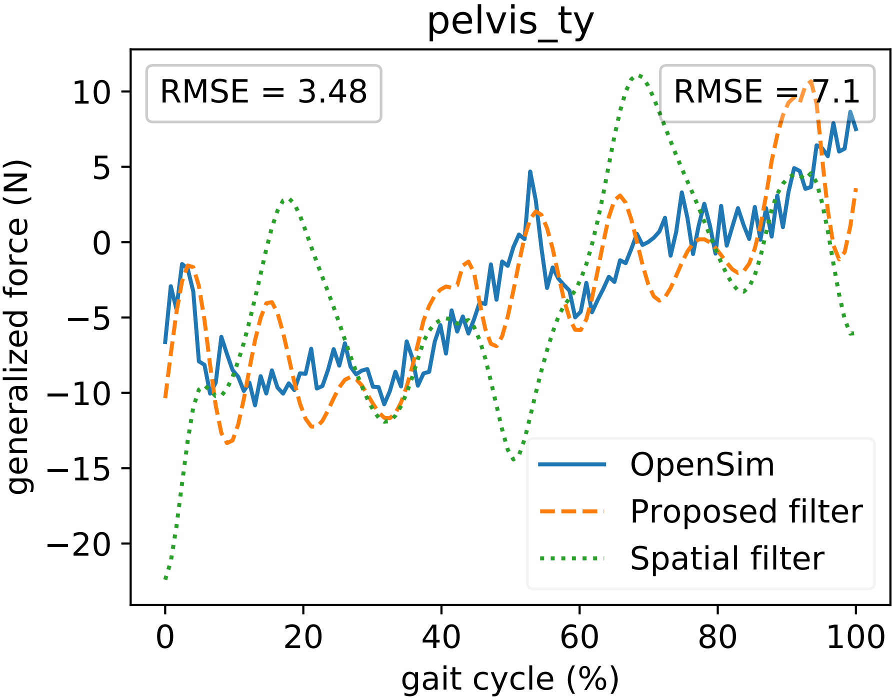

pelvis_ty
RMSE = 3.48
RMSE = 7.1
generalized force (N)
gait cycle (%)
10
5
0
−5
−10
−15
−20
0
20
40
60
80
100
OpenSim
Proposed filter
Spatial filter

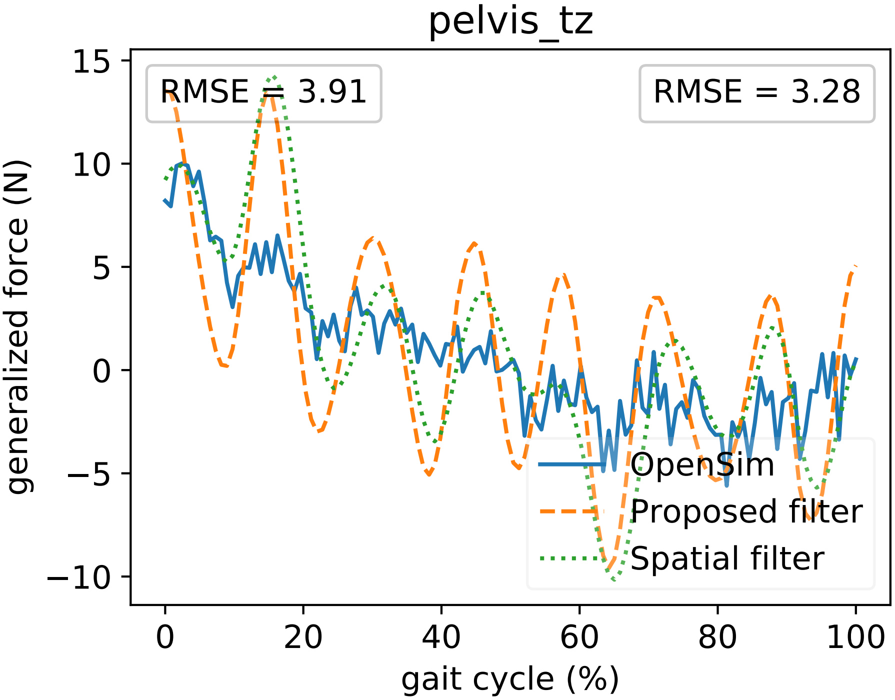

pelvis_tz
RMSE = 3.91
RMSE = 3.28
generalized force (N)
15
10
5
0
−5
−10
0
20
40
60
80
100
gait cycle (%)
OpenSim
Proposed filter
Spatial filter

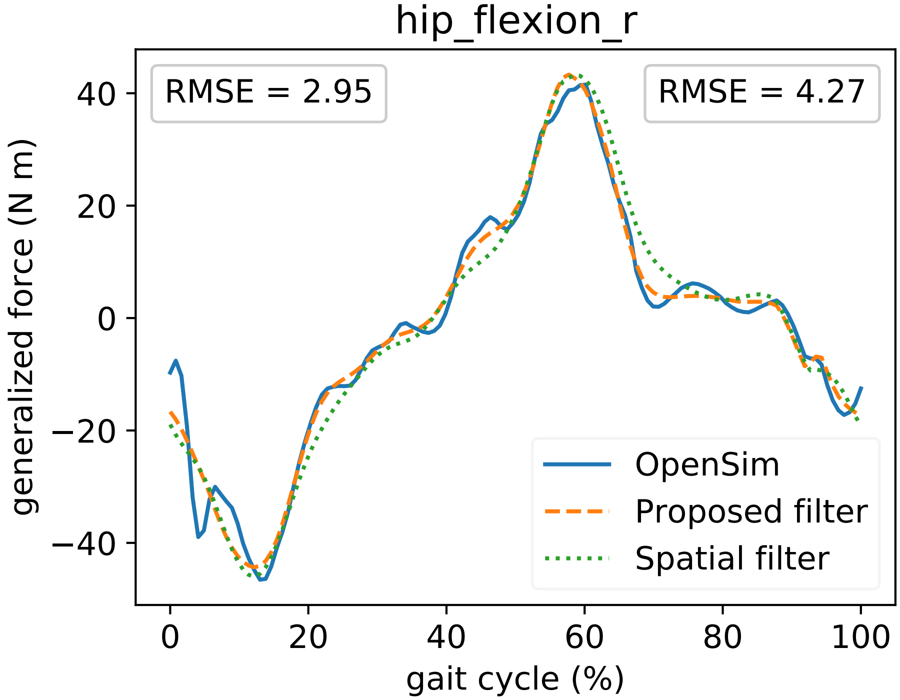
hip_flexion_r
RMSE = 2.95
RMSE = 4.27
generalized force (N m)
40
20
0
−20
−40
0
20
40
60
80
100
gait cycle (%)
OpenSim
Proposed filter
Spatial filter

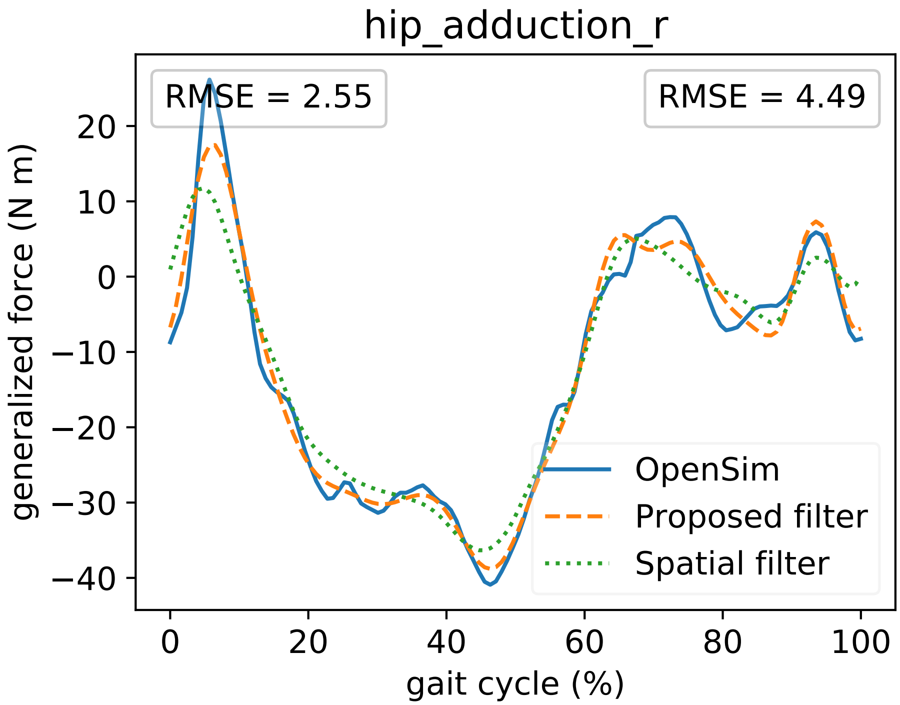
hip_adduction_r
RMSE = 2.55
RMSE = 4.49
generalized force (N m)
20
10
0
−10
−20
−30
−40
0
20
40
60
80
100
gait cycle (%)
OpenSim
Proposed filter
Spatial filter

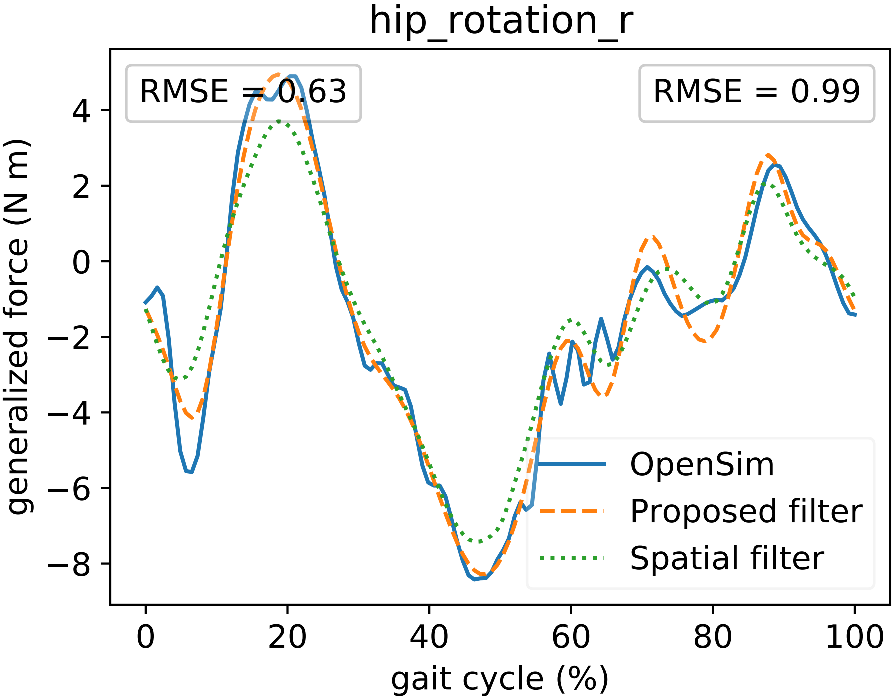

hip_rotation_r
RMSE = 0.63
RMSE = 0.99
generalized force (N m)
4
2
0
−2
−4
−6
−8
0
20
40
60
80
100
gait cycle (%)
OpenSim
Proposed filter
Spatial filter

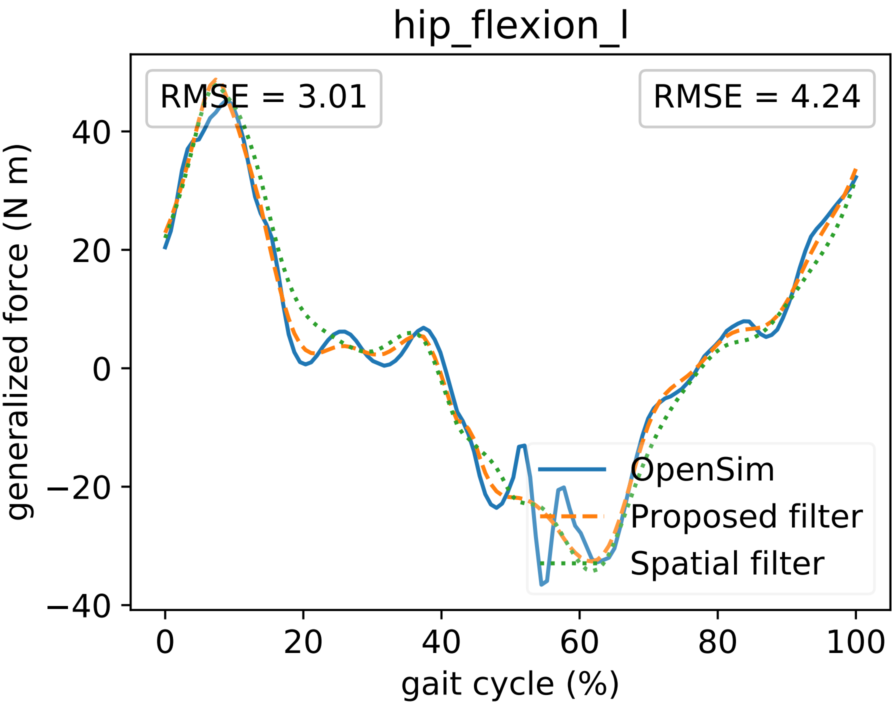

hip_flexion_l
RMSE = 3.01
RMSE = 4.24
OpenSim
Proposed filter
Spatial filter
generalized force (N m)
gait cycle (%)
40
20
0
−20
−40
0
20
40
60
80
100

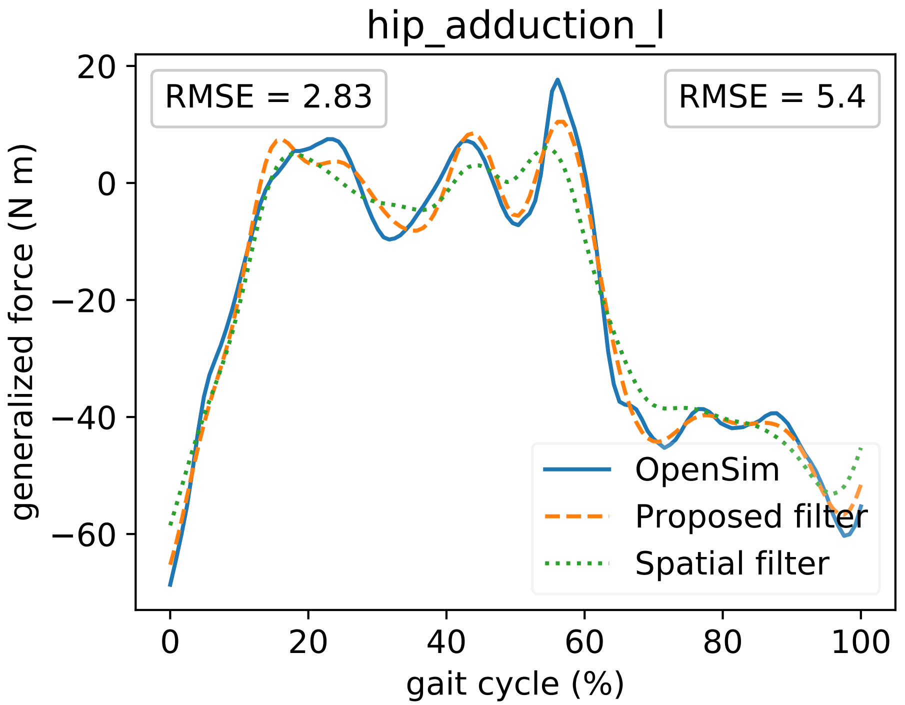
hip_adduction_l
RMSE = 2.83
RMSE = 5.4
generalized force (N m)
gait cycle (%)
OpenSim
Proposed filter
Spatial filter
20
0
−20
−40
−60
0
20
40
60
80
100

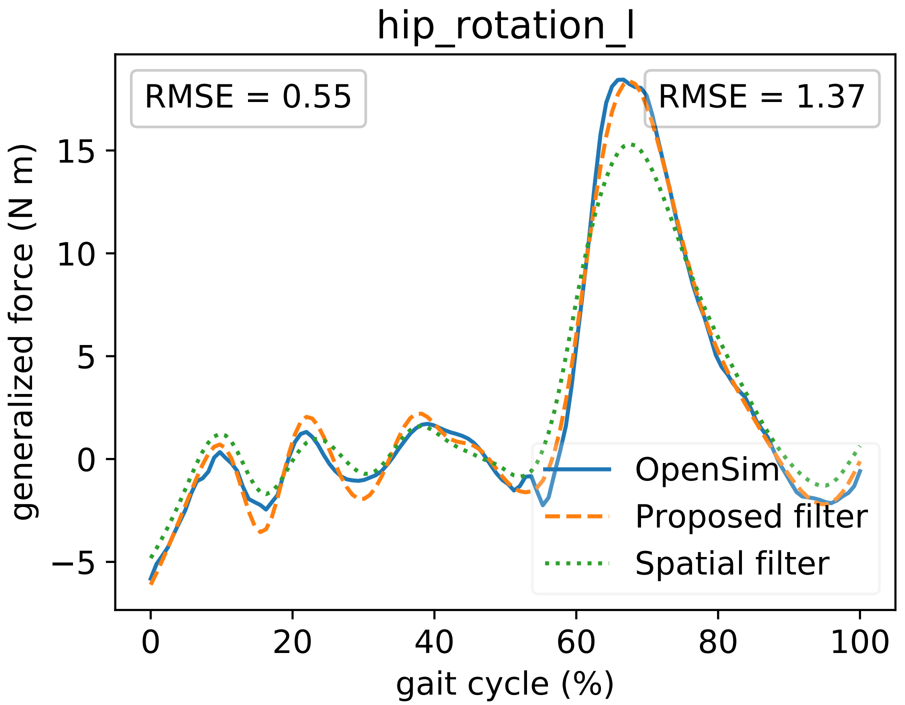
hip_rotation_l
RMSE = 0.55
RMSE = 1.37
generalized force (N m)
15
10
5
0
−5
0
20
40
60
80
100
gait cycle (%)
OpenSim
Proposed filter
Spatial filter

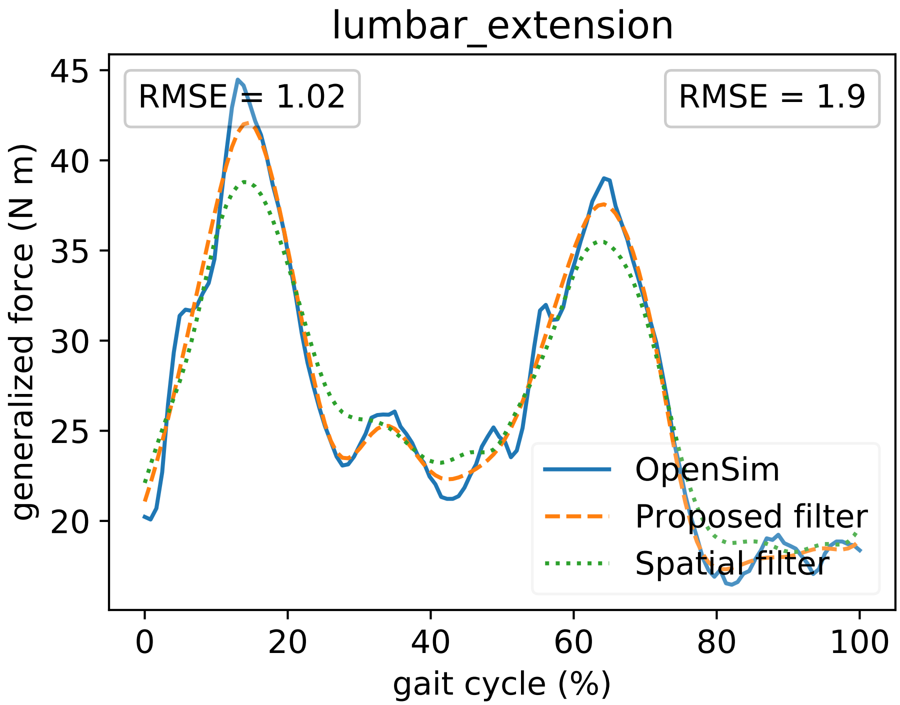

lumbar_extension
RMSE = 1.02
RMSE = 1.9
generalized force (N m)
45
40
35
30
25
20
0
20
40
60
80
100
gait cycle (%)
OpenSim
Proposed filter
Spatial filter

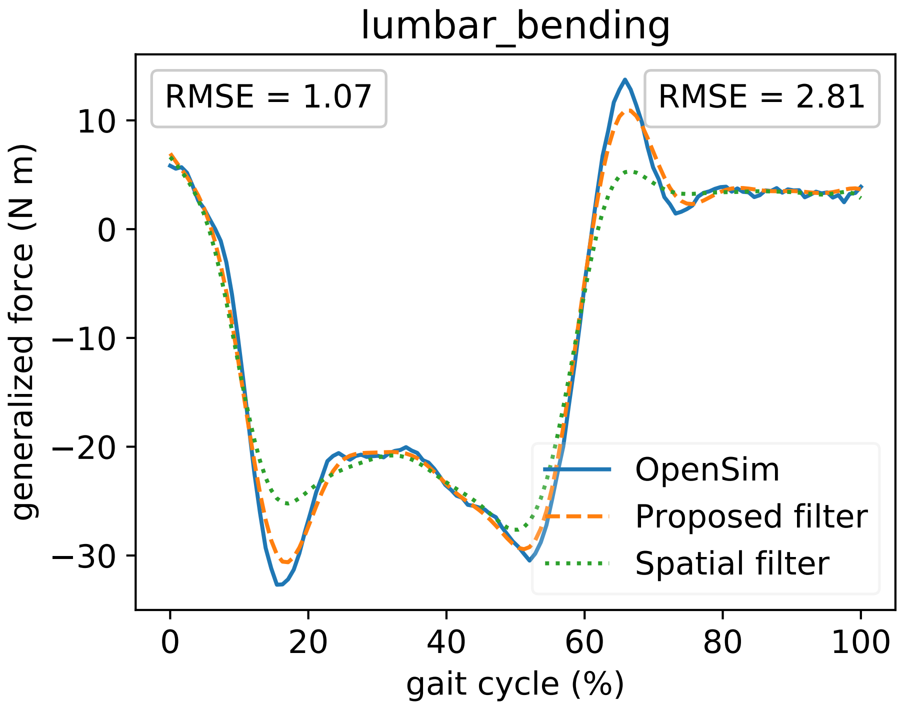

lumbar_bending
RMSE = 1.07
RMSE = 2.81
generalized force (N m)
10
0
−10
−20
−30
0
20
40
60
80
100
gait cycle (%)
OpenSim
Proposed filter
Spatial filter

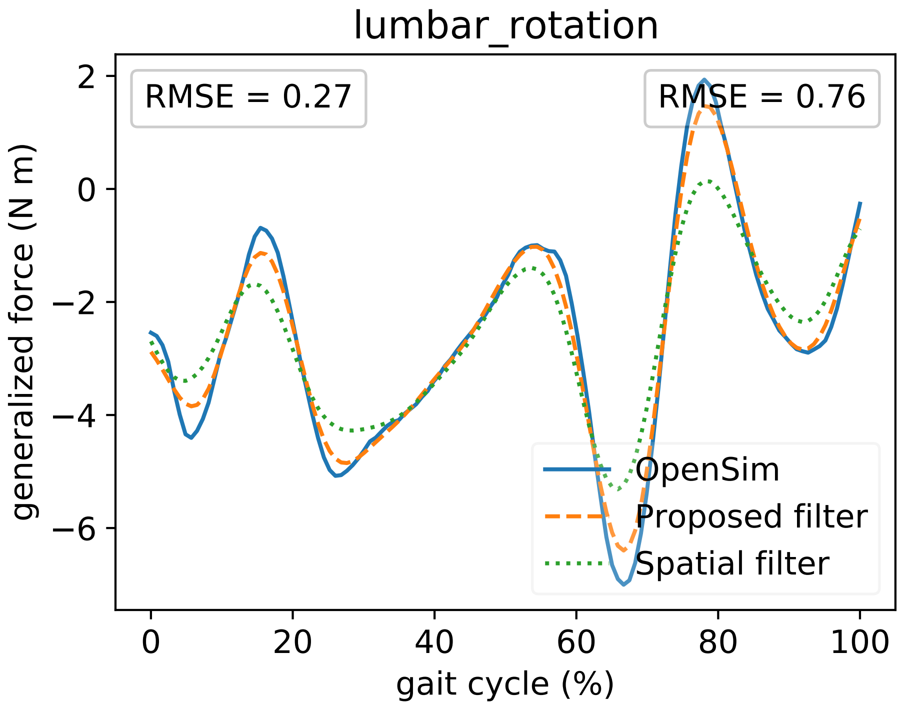

lumbar_rotation
RMSE = 0.27
RMSE = 0.76
generalized force (N m)
gait cycle (%)
OpenSim
Proposed filter
Spatial filter
2
0
−2
−4
−6
0
20
40
60
80
100

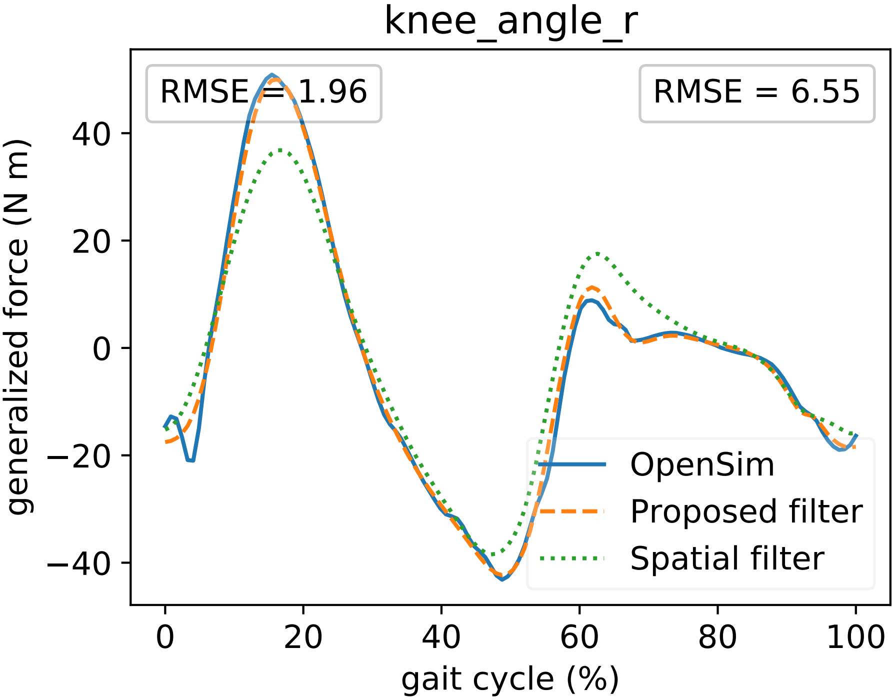

knee_angle_r
RMSE = 1.96
RMSE = 6.55
generalized force (N m)
40
20
0
−20
−40
0
20
40
60
80
100
gait cycle (%)
OpenSim
Proposed filter
Spatial filter

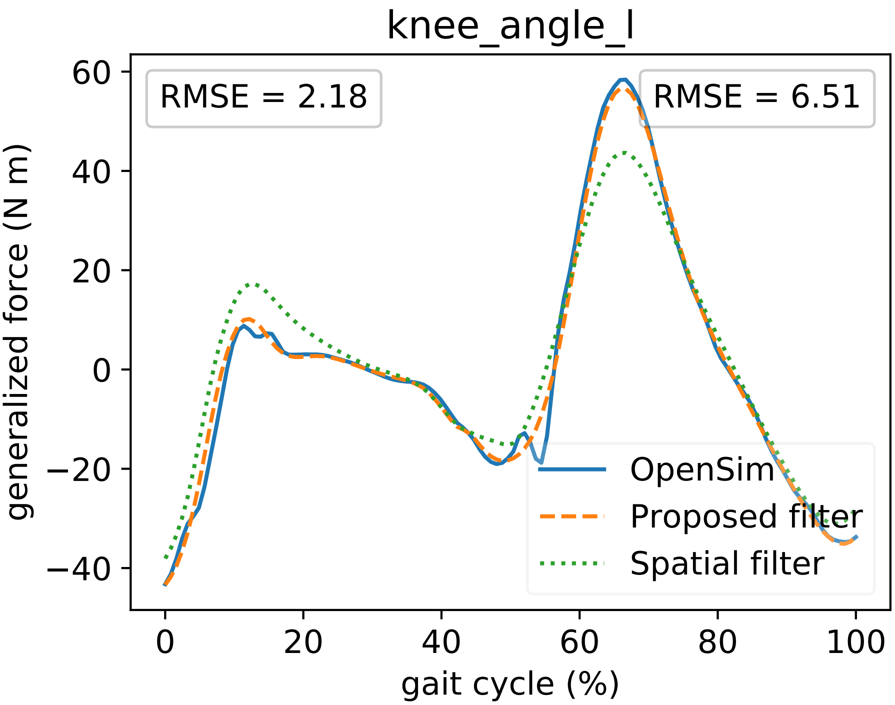
knee_angle_l
RMSE = 2.18
RMSE = 6.51
generalized force (N m)
60
40
20
0
−20
−40
0
20
40
60
80
100
gait cycle (%)
OpenSim
Proposed filter
Spatial filter

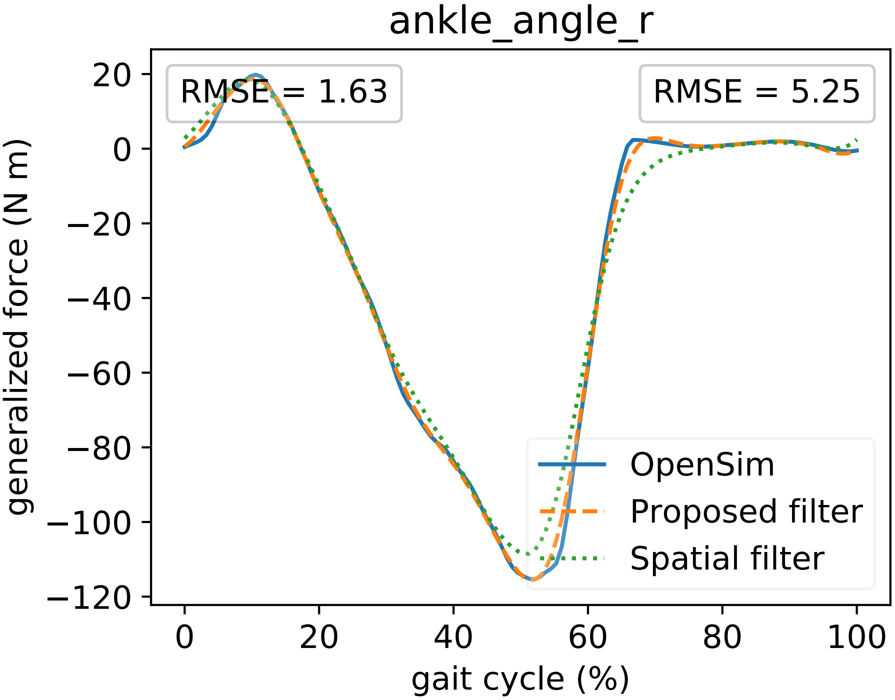

ankle_angle_r
RMSE = 1.63
RMSE = 5.25
20
0
−20
−40
−60
−80
−100
−120
generalized force (N m)
0
20
40
60
80
100
gait cycle (%)
OpenSim
Proposed filter
Spatial filter

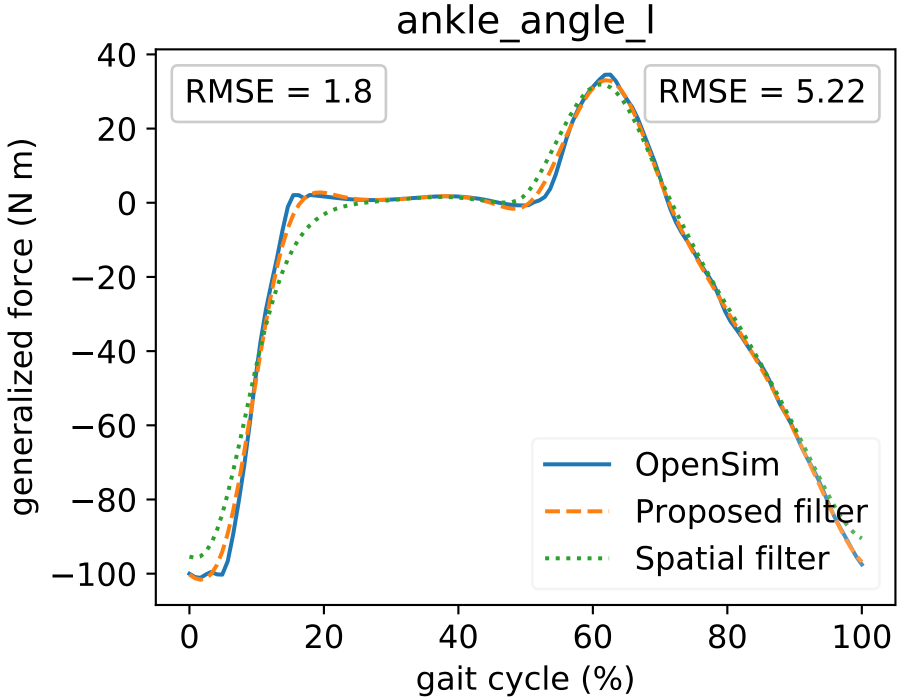
ankle_angle_l
RMSE = 1.8
RMSE = 5.22
generalized force (N m)
40
20
0
−20
−40
−60
−80
−100
0
20
40
60
80
100
gait cycle (%)
OpenSim
Proposed filter
Spatial filter
