# Supplementary material for: Real-Time Musculoskeletal Kinematics and Dynamics Analysis Using Marker- and IMU-Based Solutions in Rehabilitation
Source: Sensors (Basel). 2021 Mar 5;21(5):1804. doi: 10.3390/s21051804 (PMC7961635; doi:10.3390/s21051804)
Supplement: Supplementary file 1 [file sensors-21-01804-s001.zip › supplementary_inverse_kinematics.pdf]

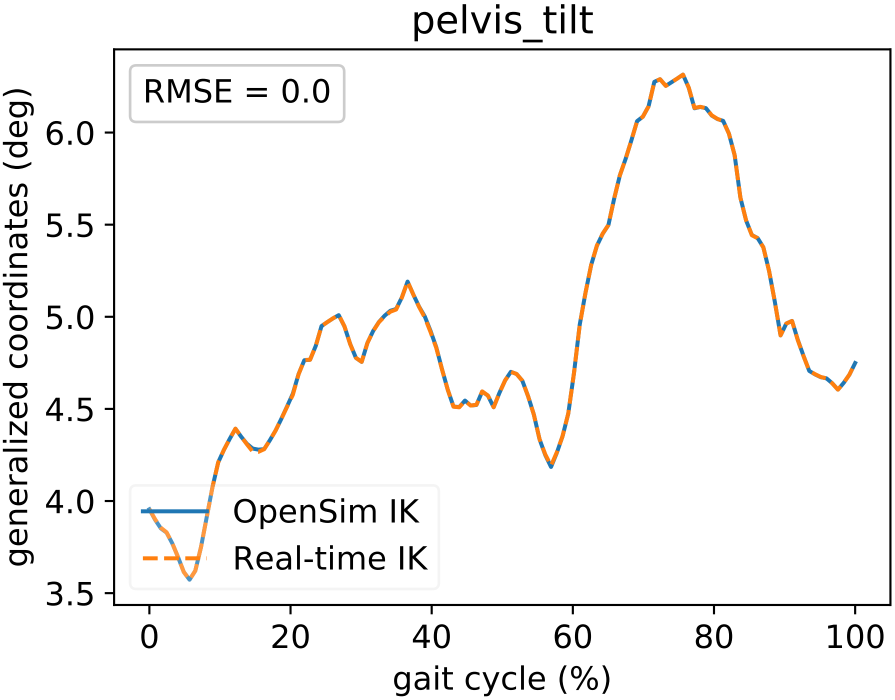
pelvis_tilt
RMSE = 0.0
OpenSim IK
Real-time IK
generalized coordinates (deg)
gait cycle (%)
3.5
4.0
4.5
5.0
5.5
6.0
0
20
40
60
80
100

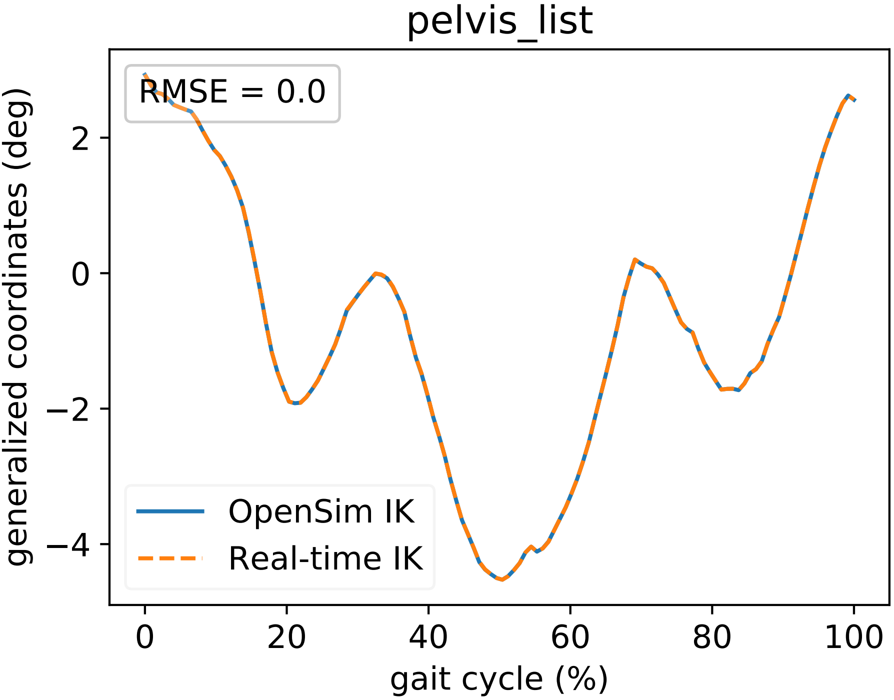
pelvis_list
RMSE = 0.0
OpenSim IK
Real-time IK
generalized coordinates (deg)
2
0
−2
−4
0
20
40
60
80
100
gait cycle (%)

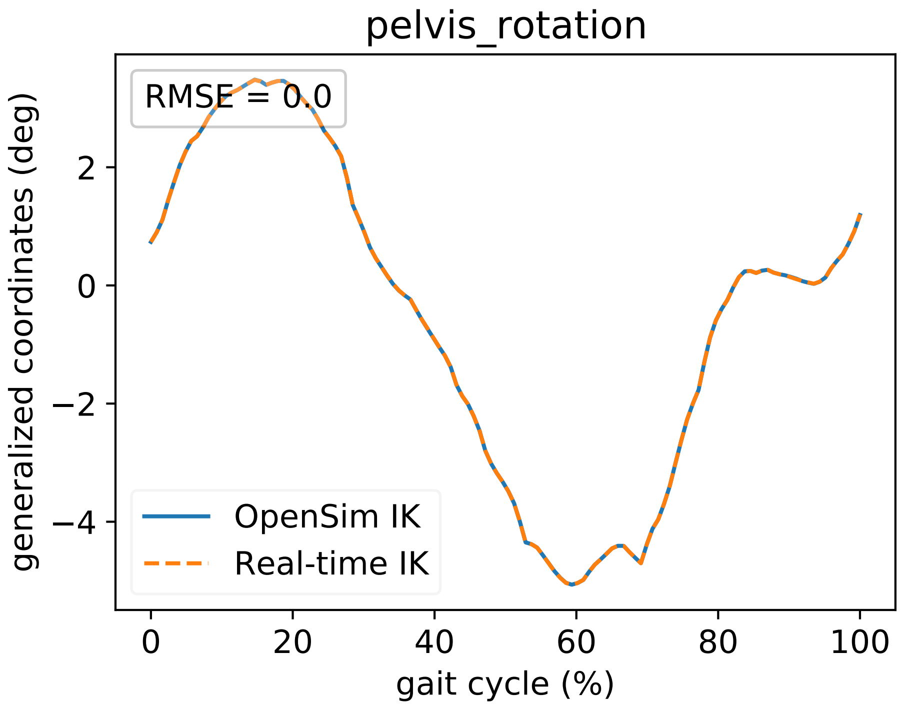

pelvis_rotation
RMSE = 0.0
OpenSim IK
Real-time IK
generalized coordinates (deg)
gait cycle (%)
2
0
−2
−4
0
20
40
60
80
100

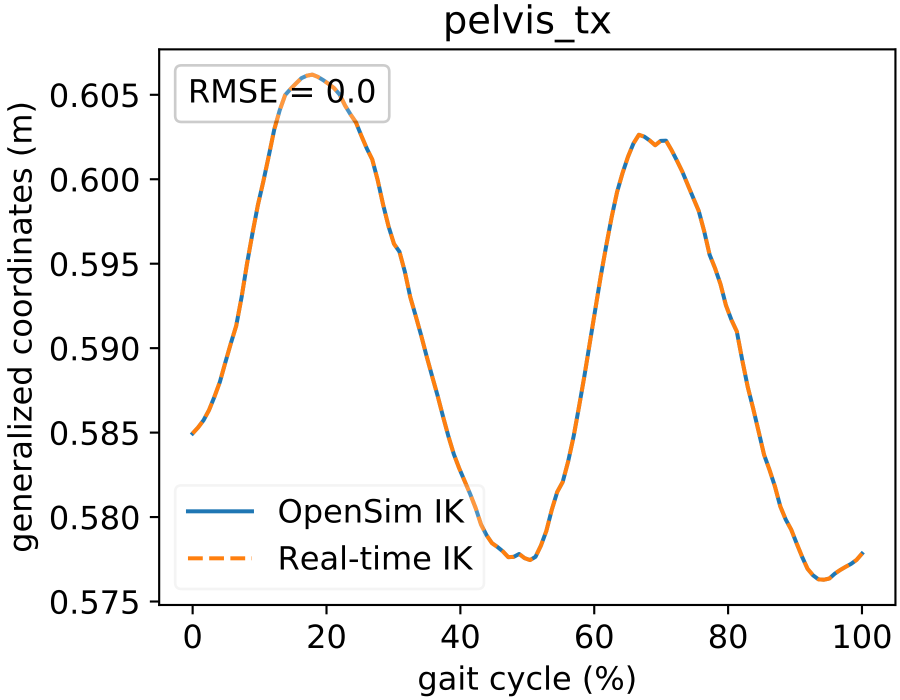

pelvis_tx
RMSE = 0.0
OpenSim IK
Real-time IK
generalized coordinates (m)
0.605
0.600
0.595
0.590
0.585
0.580
0.575
0
20
40
60
80
100
gait cycle (%)

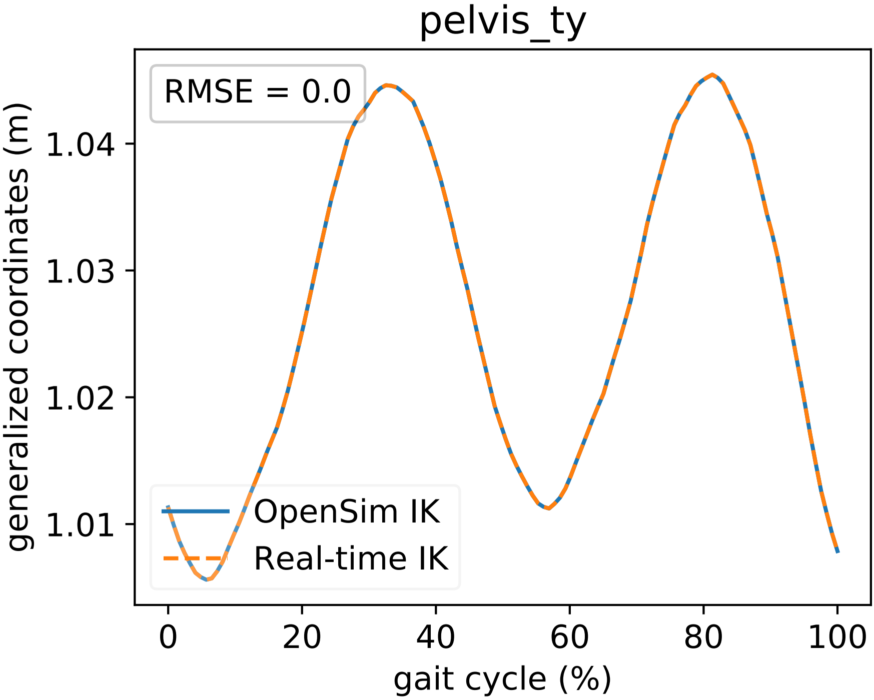
pelvis_ty
RMSE = 0.0
OpenSim IK
Real-time IK
generalized coordinates (m)
1.01
1.02
1.03
1.04
0
20
40
60
80
100
gait cycle (%)

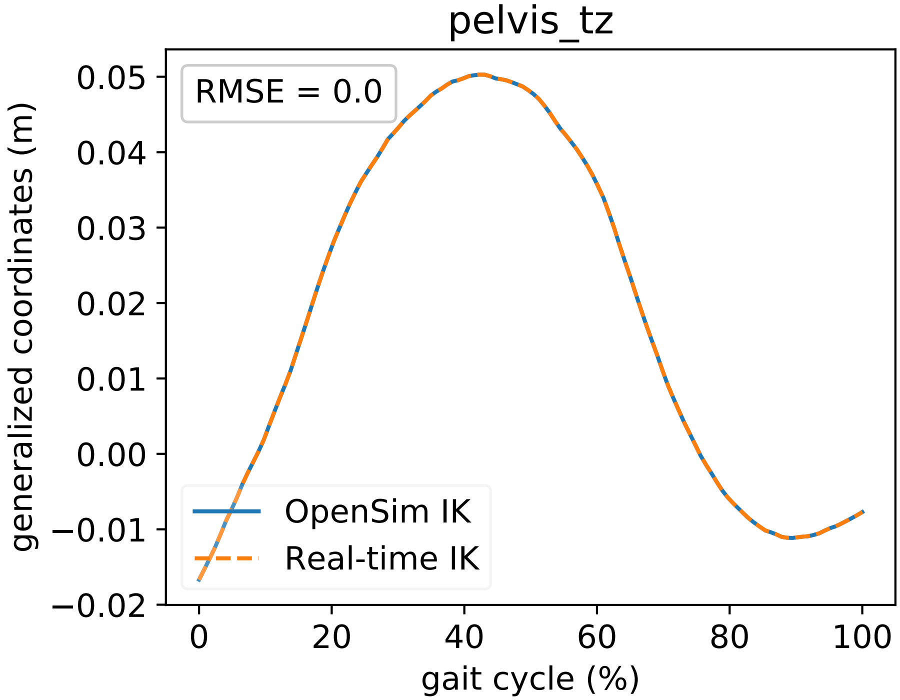

pelvis_tz
RMSE = 0.0
OpenSim IK
Real-time IK
generalized coordinates (m)
gait cycle (%)
0.05
0.04
0.03
0.02
0.01
0.00
−0.01
−0.02
0
20
40
60
80
100

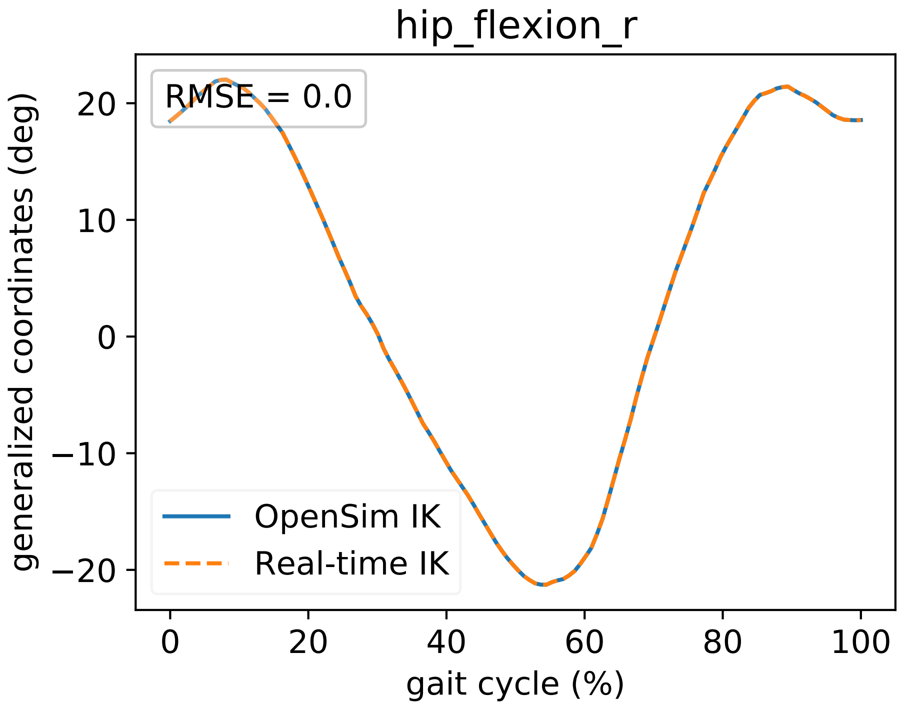
hip_flexion_r
RMSE = 0.0
OpenSim IK
Real-time IK
generalized coordinates (deg)
gait cycle (%)
20
10
0
−10
−20
0
20
40
60
80
100

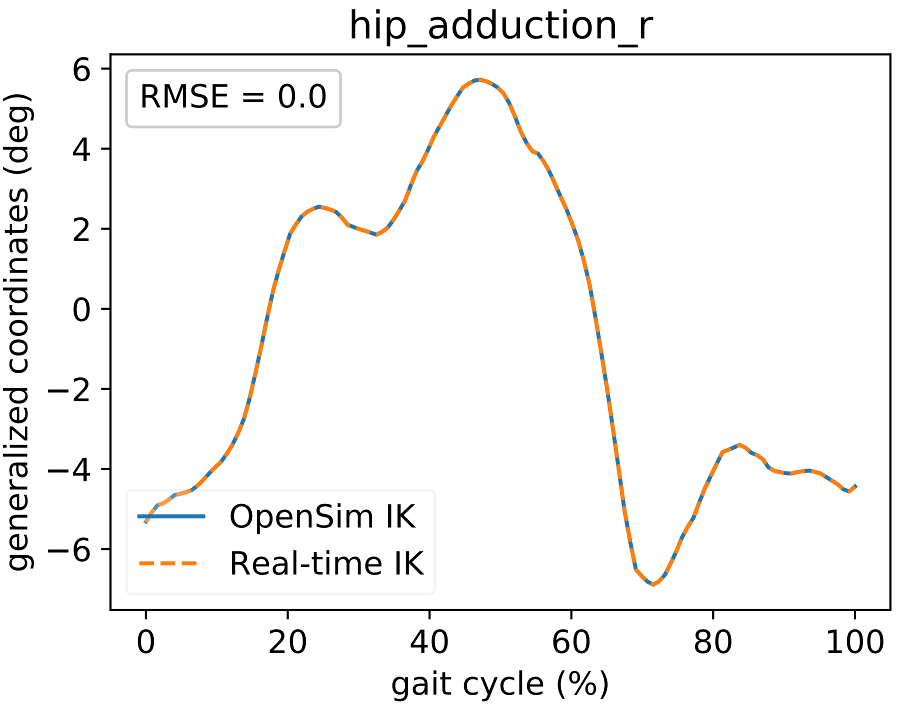

hip_adduction_r
RMSE = 0.0
OpenSim IK
Real-time IK
generalized coordinates (deg)
gait cycle (%)
6
4
2
0
−2
−4
−6
0
20
40
60
80
100

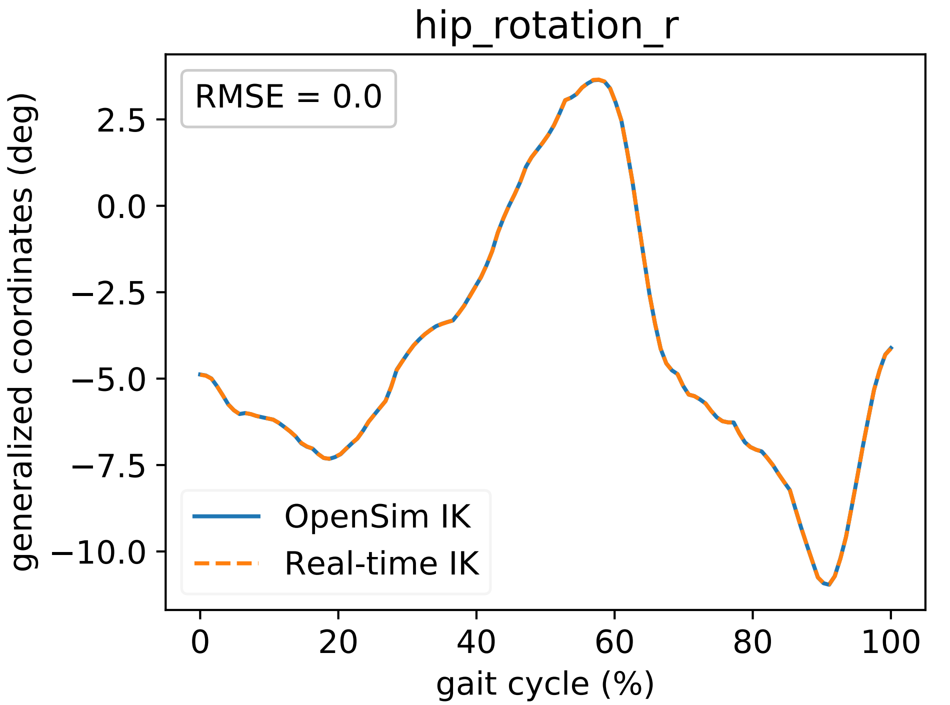

hip_rotation_r
RMSE = 0.0
OpenSim IK
Real-time IK
generalized coordinates (deg)
2.5
0.0
−2.5
−5.0
−7.5
−10.0
0
20
40
60
80
100
gait cycle (%)

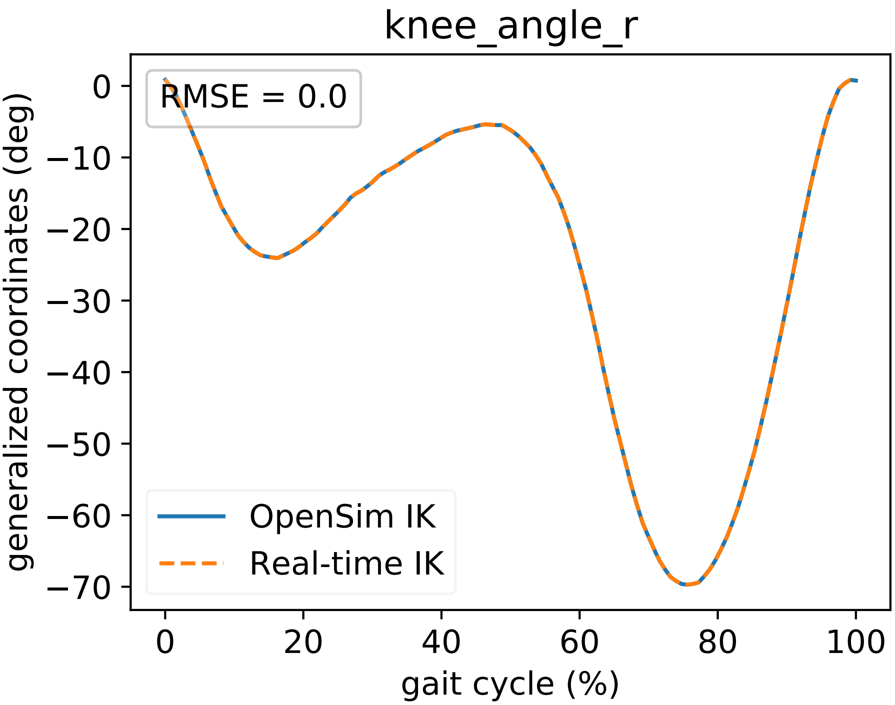

knee_angle_r
RMSE = 0.0
OpenSim IK
Real-time IK
generalized coordinates (deg)
gait cycle (%)
0
−10
−20
−30
−40
−50
−60
−70
0
20
40
60
80
100

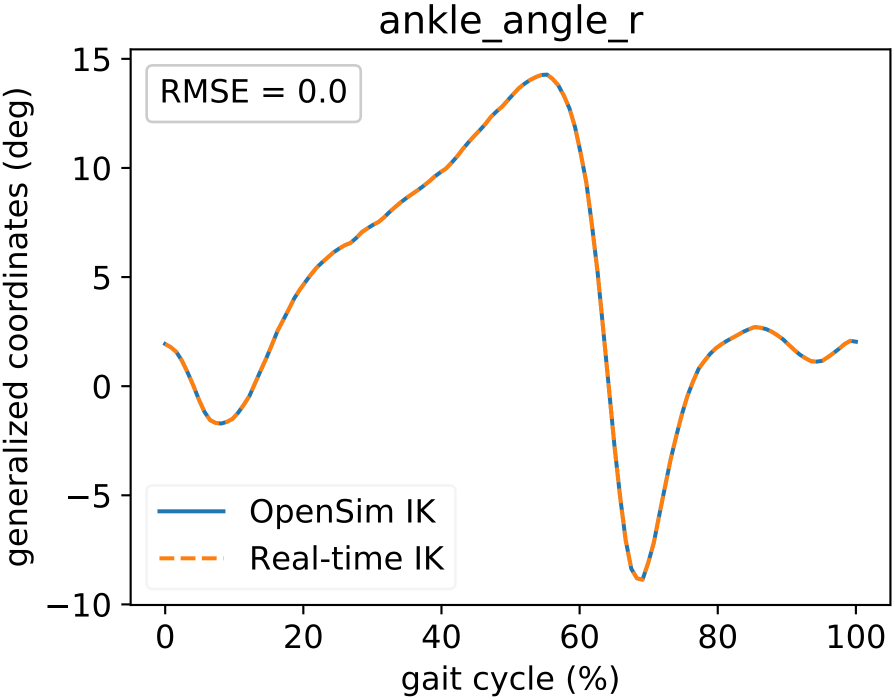

ankle_angle_r
RMSE = 0.0
OpenSim IK
Real-time IK
generalized coordinates (deg)
gait cycle (%)
15
10
5
0
−5
−10
0
20
40
60
80
100

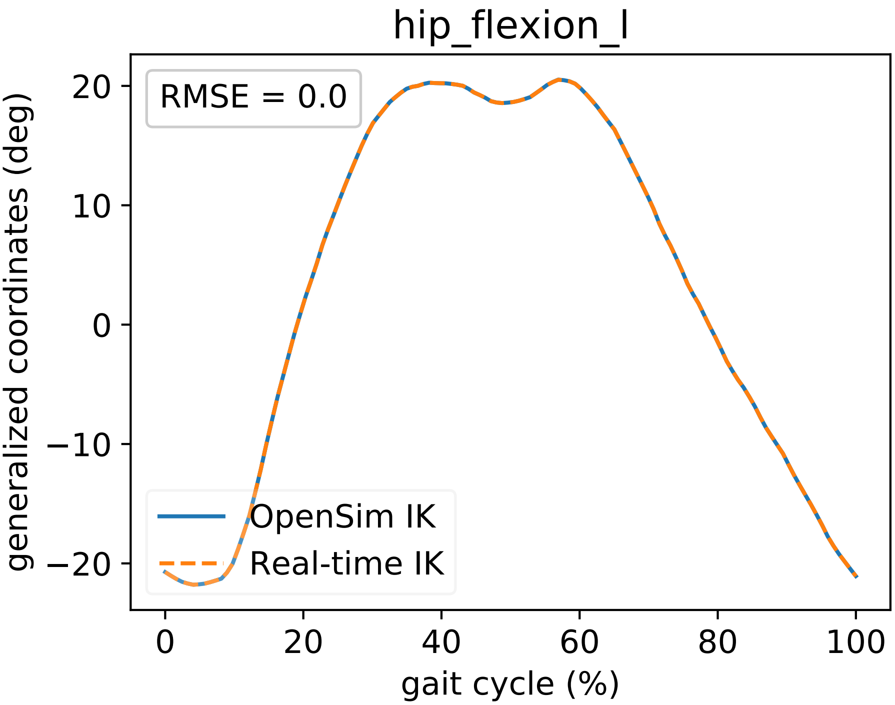
hip_flexion_l
RMSE = 0.0
OpenSim IK
Real-time IK
generalized coordinates (deg)
gait cycle (%)
20
10
0
−10
−20
0
20
40
60
80
100

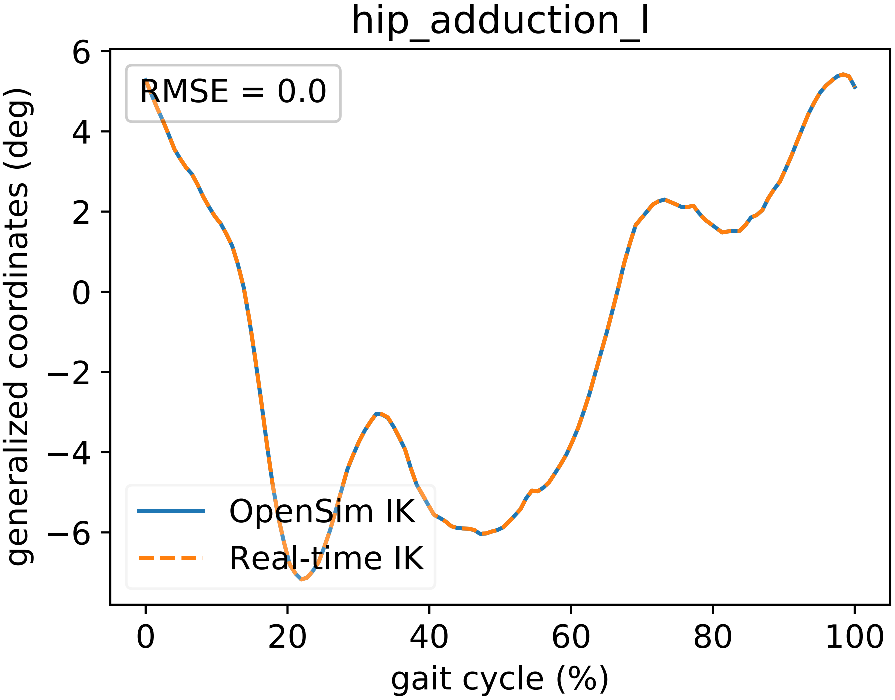
hip_adduction_l
RMSE = 0.0
OpenSim IK
Real-time IK
generalized coordinates (deg)
gait cycle (%)
6
4
2
0
−2
−4
−6
0
20
40
60
80
100

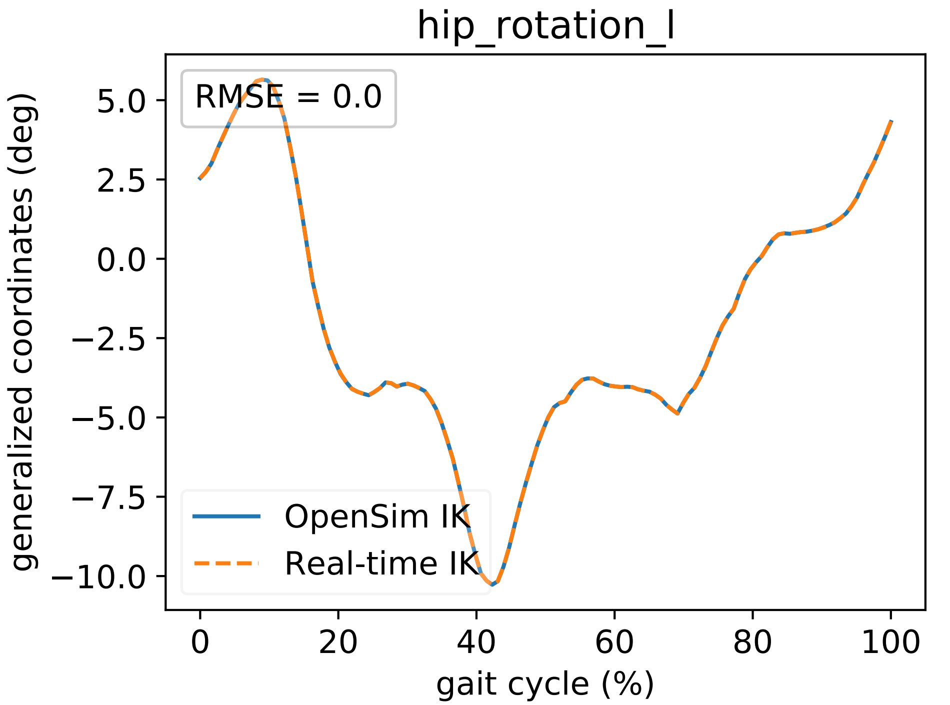
hip_rotation_l
RMSE = 0.0
OpenSim IK
Real-time IK
generalized coordinates (deg)
gait cycle (%)
5.0
2.5
0.0
−2.5
−5.0
−7.5
−10.0
0
20
40
60
80
100

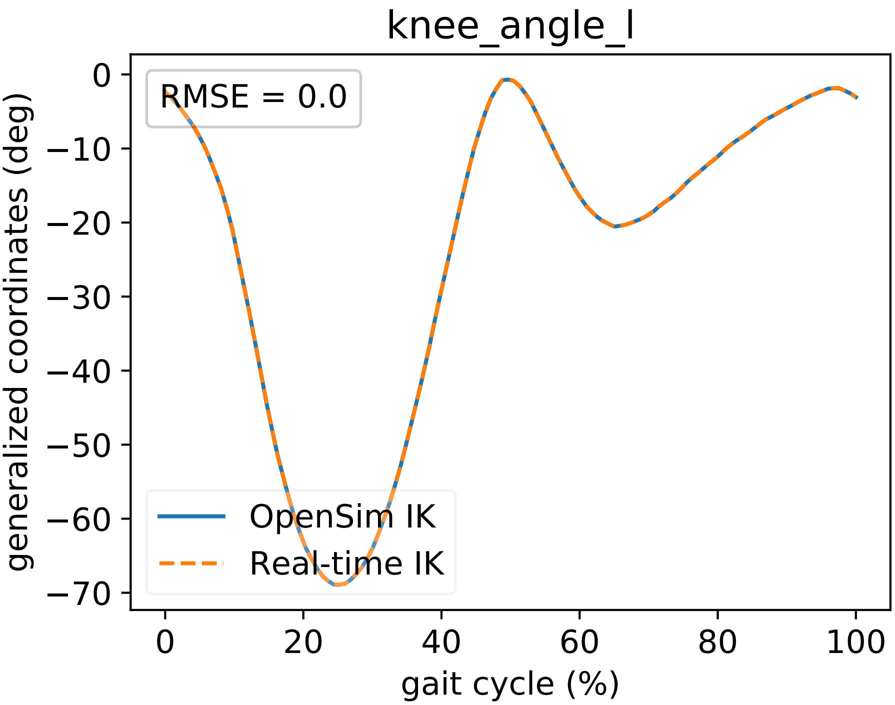

knee_angle_l
RMSE = 0.0
OpenSim IK
Real-time IK
generalized coordinates (deg)
gait cycle (%)
0
−10
−20
−30
−40
−50
−60
−70
0
20
40
60
80
100

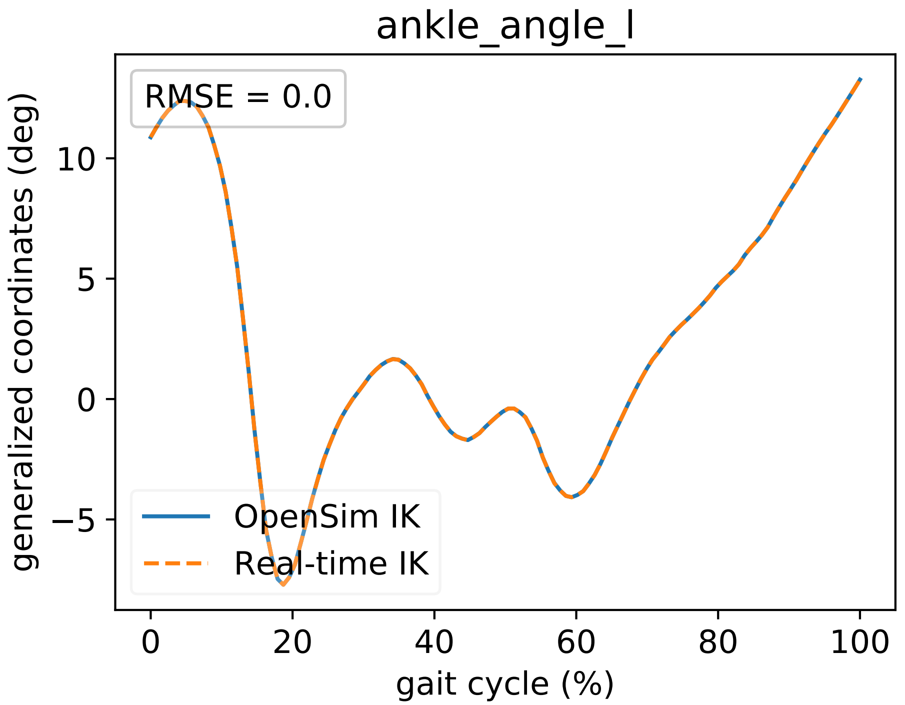
ankle_angle_l
RMSE = 0.0
generalized coordinates (deg)
10
5
0
−5
0
20
40
60
80
100
gait cycle (%)
OpenSim IK
Real-time IK

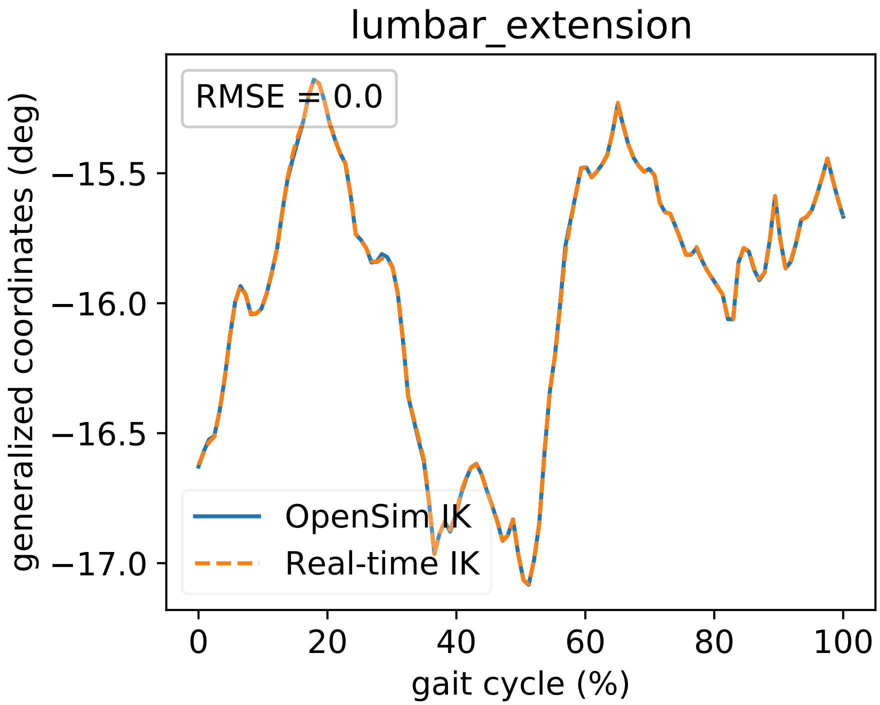
lumbar_extension
RMSE = 0.0
OpenSim IK
Real-time IK
generalized coordinates (deg)
−15.5
−16.0
−16.5
−17.0
0
20
40
60
80
100
gait cycle (%)

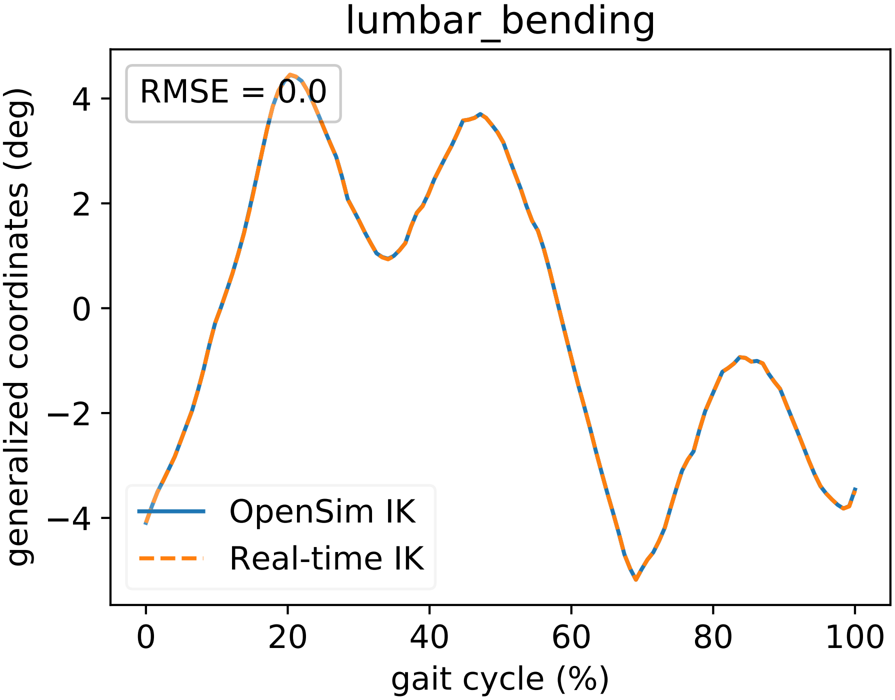
lumbar_bending
RMSE = 0.0
OpenSim IK
Real-time IK
generalized coordinates (deg)
gait cycle (%)
4
2
0
−2
−4
0
20
40
60
80
100

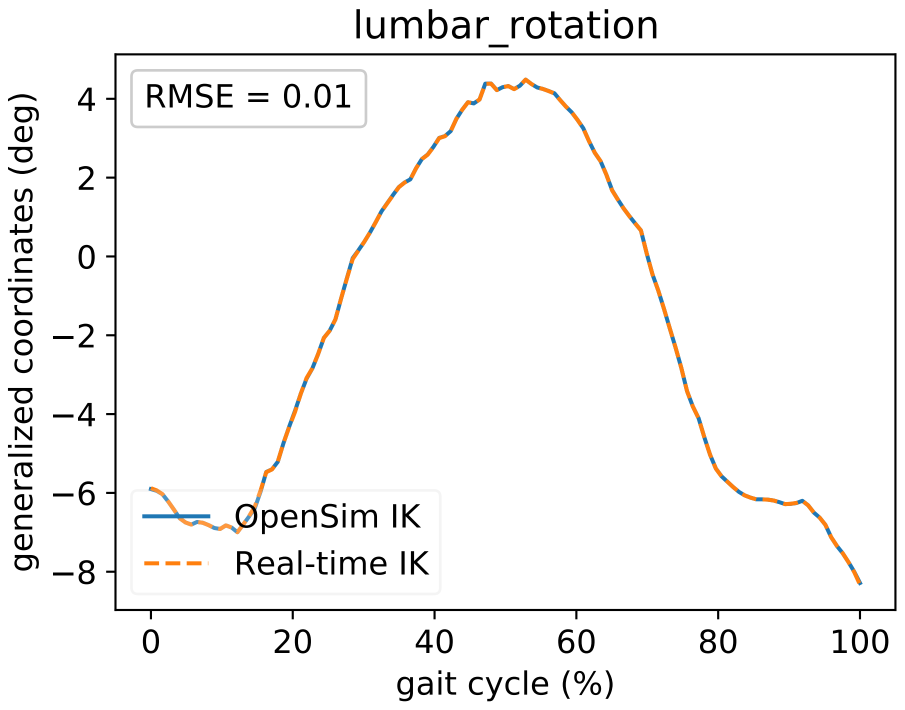

lumbar_rotation
RMSE = 0.01
OpenSim IK
Real-time IK
generalized coordinates (deg)
gait cycle (%)
4
2
0
−2
−4
−6
−8
0
20
40
60
80
100
