# Supplementary material for: Real-Time Musculoskeletal Kinematics and Dynamics Analysis Using Marker- and IMU-Based Solutions in Rehabilitation
Source: Sensors (Basel). 2021 Mar 5;21(5):1804. doi: 10.3390/s21051804 (PMC7961635; doi:10.3390/s21051804)
Supplement: Supplementary file 1 [file sensors-21-01804-s001.zip › supplementary_joint_reactions.pdf]

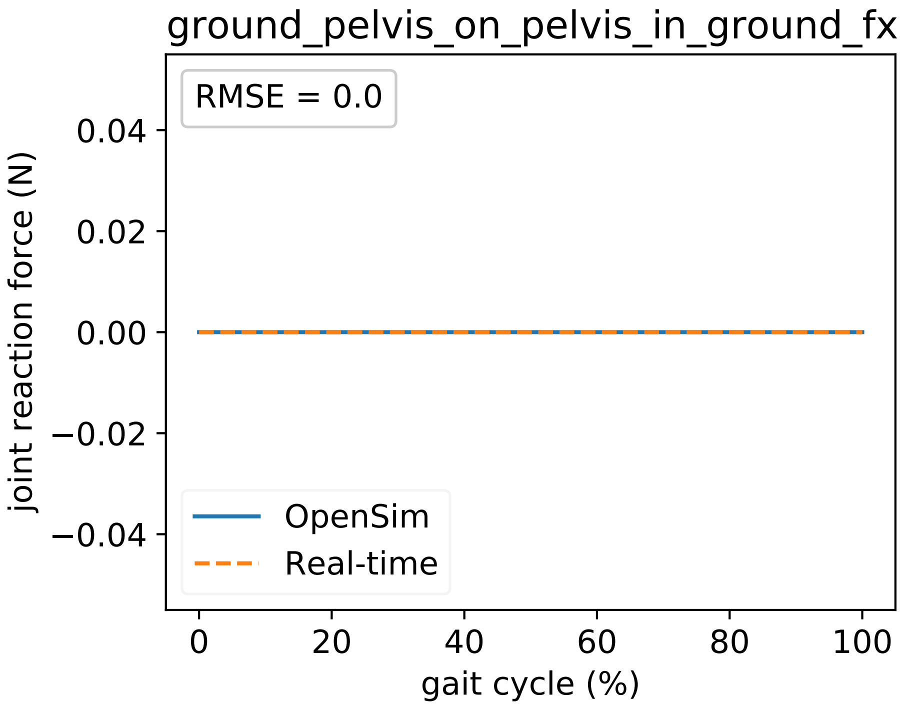
ground_pelvis_on_pelvis_in_ground_fx
RMSE = 0.0
joint reaction force (N)
0.04
0.02
0.00
−0.02
−0.04
OpenSim
Real-time
0
20
40
60
80
100
gait cycle (%)

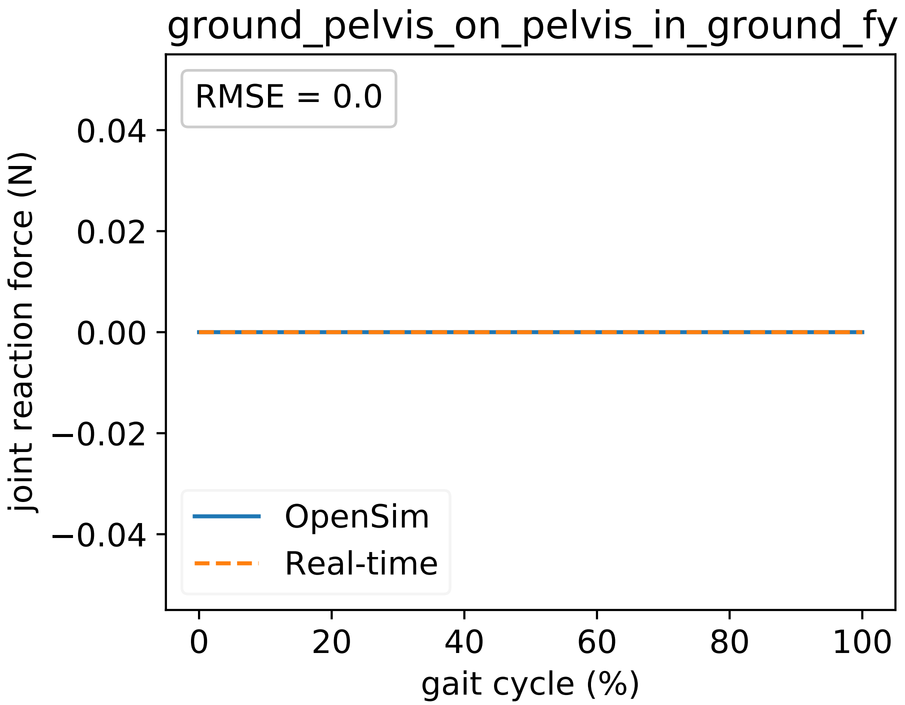
ground_pelvis_on_pelvis_in_ground_fy
RMSE = 0.0
0.04
0.02
0.00
−0.02
−0.04
joint reaction force (N)
OpenSim
Real-time
0
20
40
60
80
100
gait cycle (%)

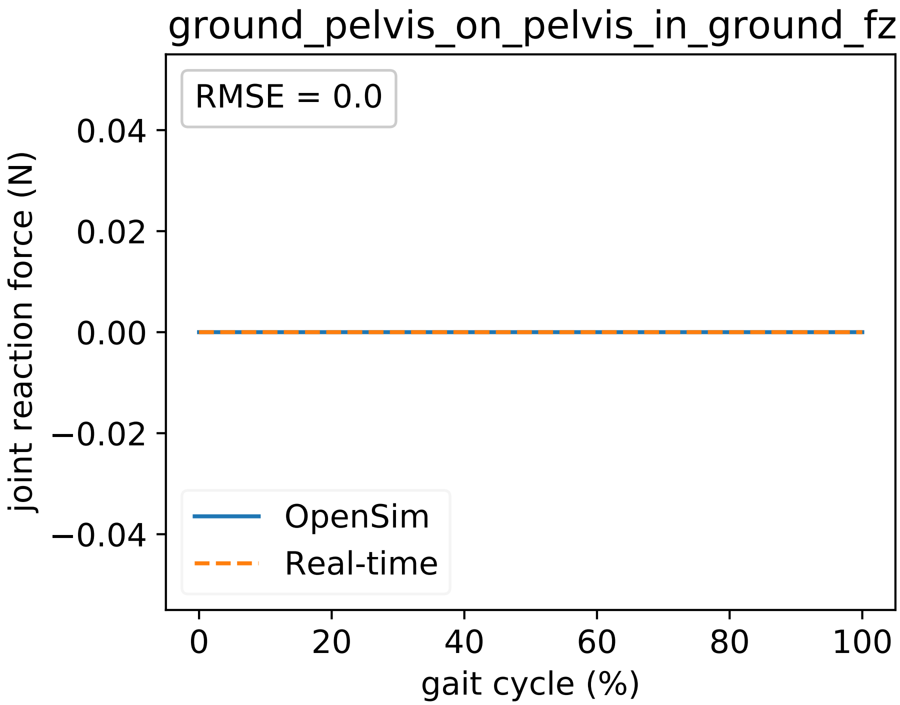

ground_pelvis_on_pelvis_in_ground_fz
RMSE = 0.0
0.04
0.02
0.00
−0.02
−0.04
joint reaction force (N)
OpenSim
Real-time
0
20
40
60
80
100
gait cycle (%)

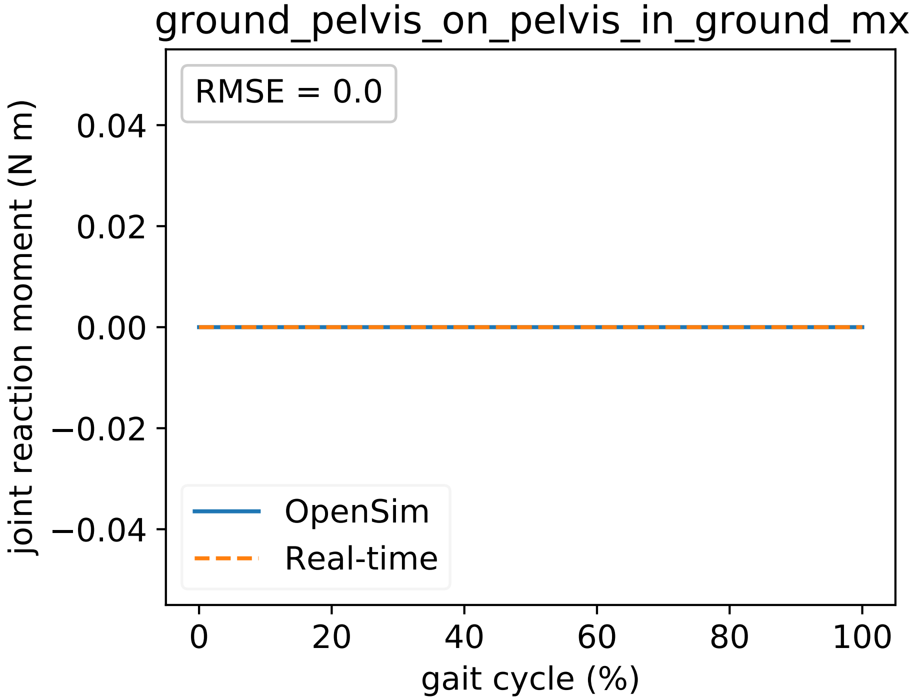
ground_pelvis_on_pelvis_in_ground_mx
RMSE = 0.0
0.04
0.02
0.00
−0.02
−0.04
joint reaction moment (N m)
OpenSim
Real-time
0
20
40
60
80
100
gait cycle (%)

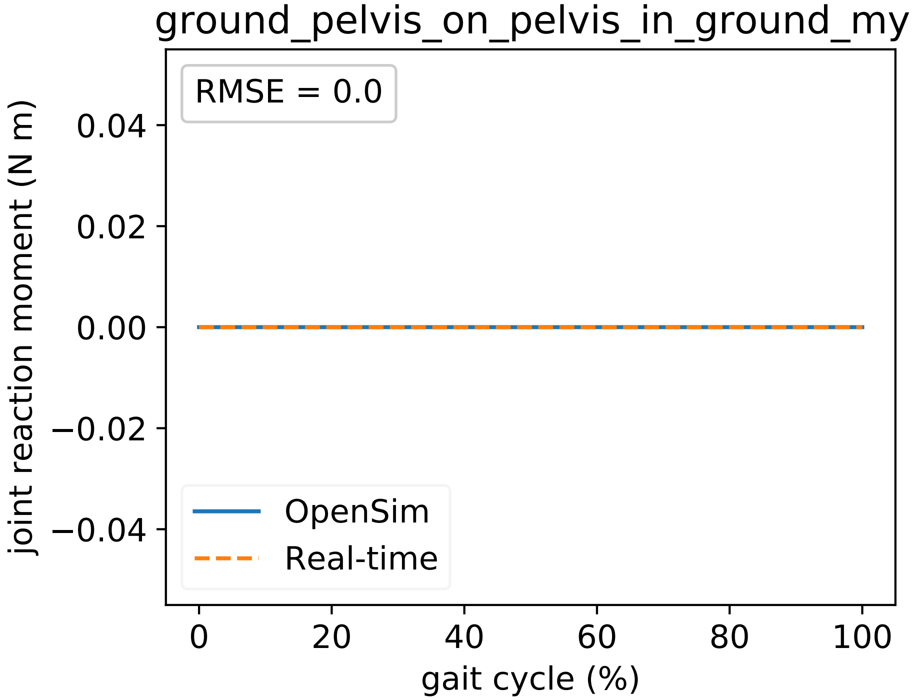

ground_pelvis_on_pelvis_in_ground_my
RMSE = 0.0
joint reaction moment (N m)
0.04
0.02
0.00
−0.02
−0.04
OpenSim
Real-time
0
20
40
60
80
100
gait cycle (%)

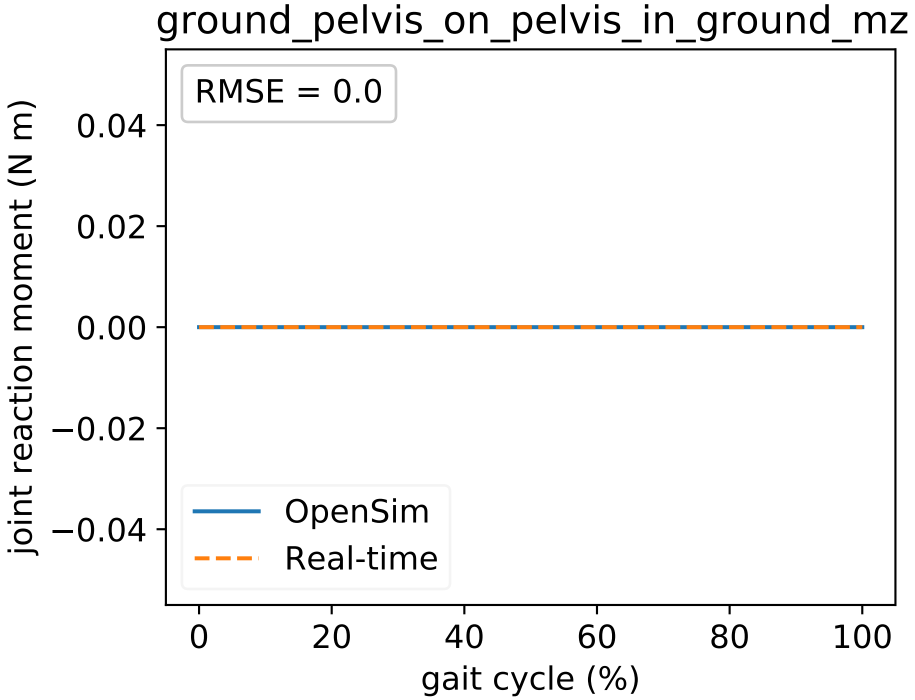
ground_pelvis_on_pelvis_in_ground_mz
RMSE = 0.0
joint reaction moment (N m)
0.04
0.02
0.00
−0.02
−0.04
OpenSim
Real-time
0
20
40
60
80
100
gait cycle (%)

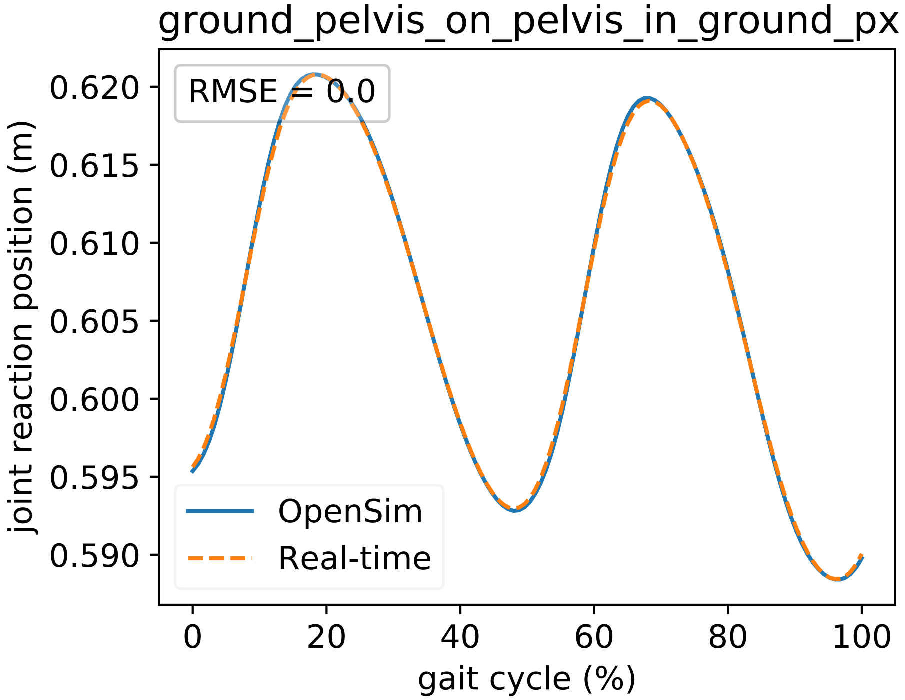

ground_pelvis_on_pelvis_in_ground_px
RMSE = 0.0
joint reaction position (m)
0.620
0.615
0.610
0.605
0.600
0.595
0.590
OpenSim
Real-time
0
20
40
60
80
100
gait cycle (%)

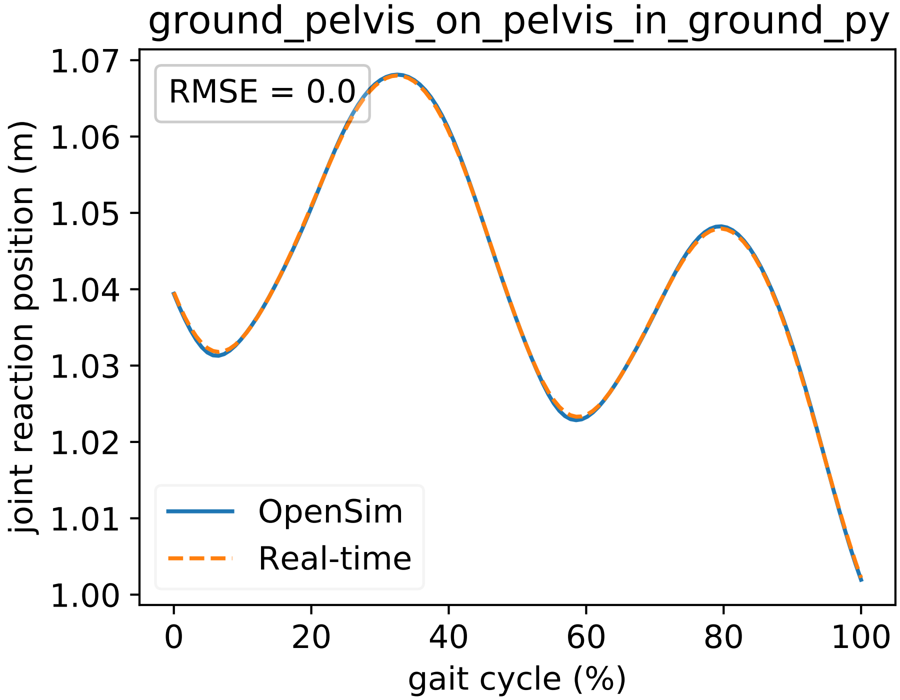

ground_pelvis_on_pelvis_in_ground_py
RMSE = 0.0
OpenSim
Real-time
joint reaction position (m)
gait cycle (%)
1.07
1.06
1.05
1.04
1.03
1.02
1.01
1.00
0
20
40
60
80
100

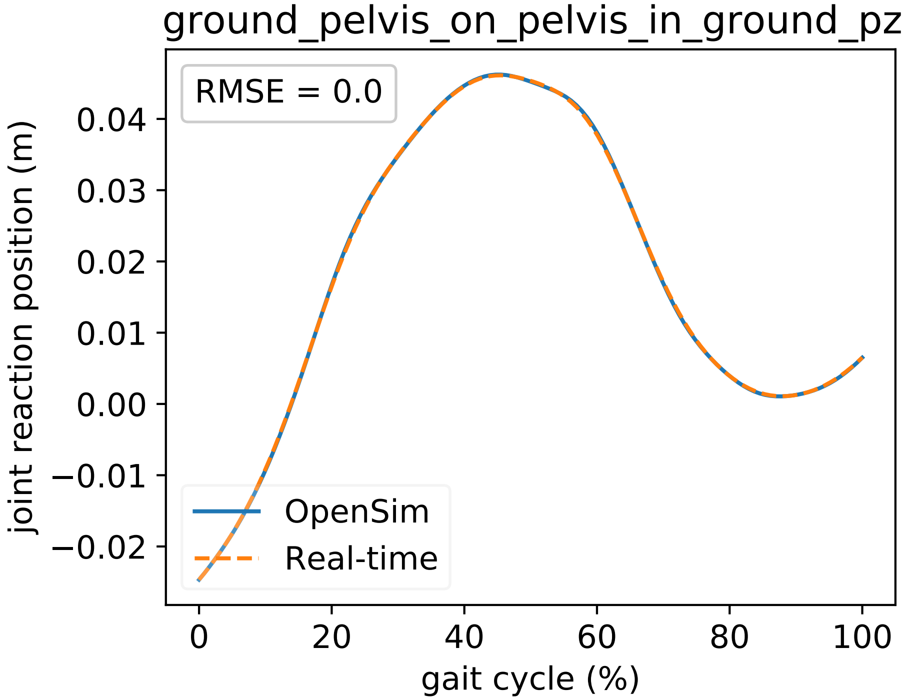
ground_pelvis_on_pelvis_in_ground_pz
RMSE = 0.0
OpenSim
Real-time
joint reaction position (m)
gait cycle (%)
0.04
0.03
0.02
0.01
0.00
−0.01
−0.02
0
20
40
60
80
100

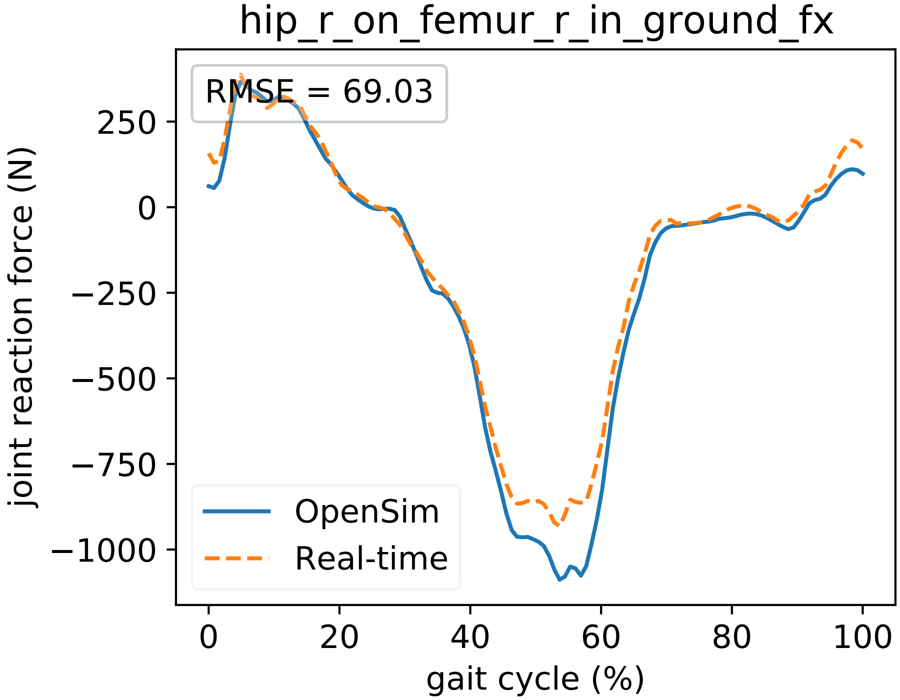
hip_r_on_femur_r_in_ground_fx
RMSE = 69.03
joint reaction force (N)
250
0
−250
−500
−750
−1000
0
20
40
60
80
100
gait cycle (%)
OpenSim
Real-time

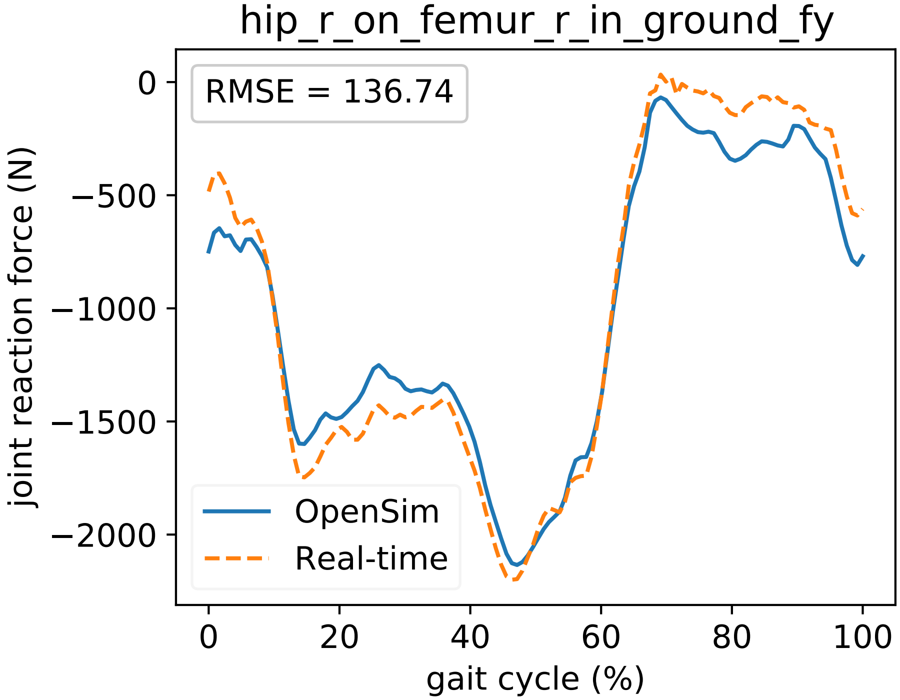

hip_r_on_femur_r_in_ground_fy
RMSE = 136.74
OpenSim
Real-time
joint reaction force (N)
gait cycle (%)
0
−500
−1000
−1500
−2000
0
20
40
60
80
100

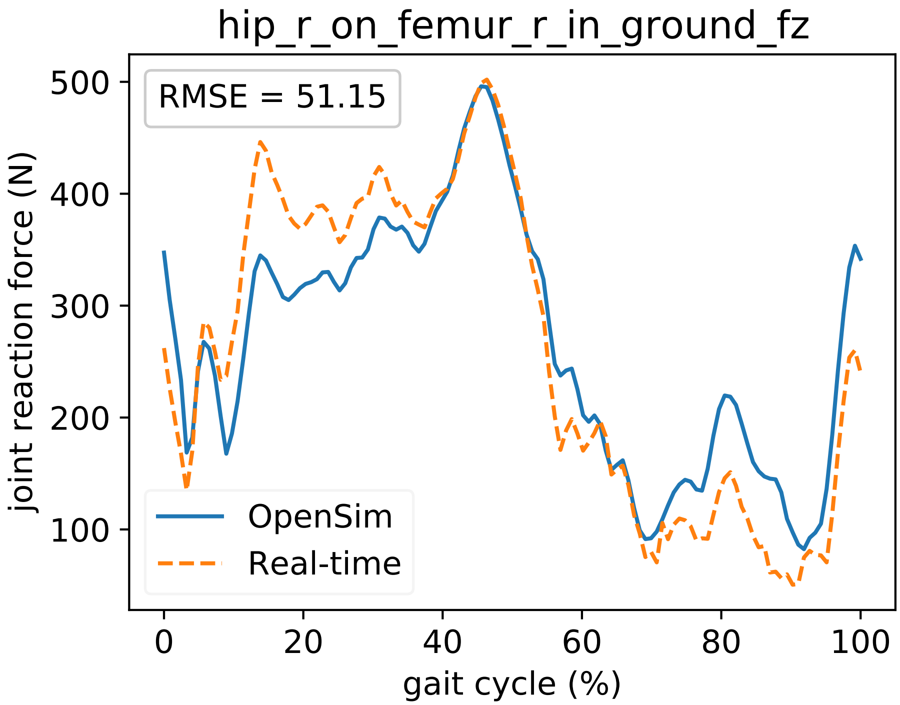
hip_r_on_femur_r_in_ground_fz
RMSE = 51.15
joint reaction force (N)
gait cycle (%)
OpenSim
Real-time
500
400
300
200
100
0
20
40
60
80
100

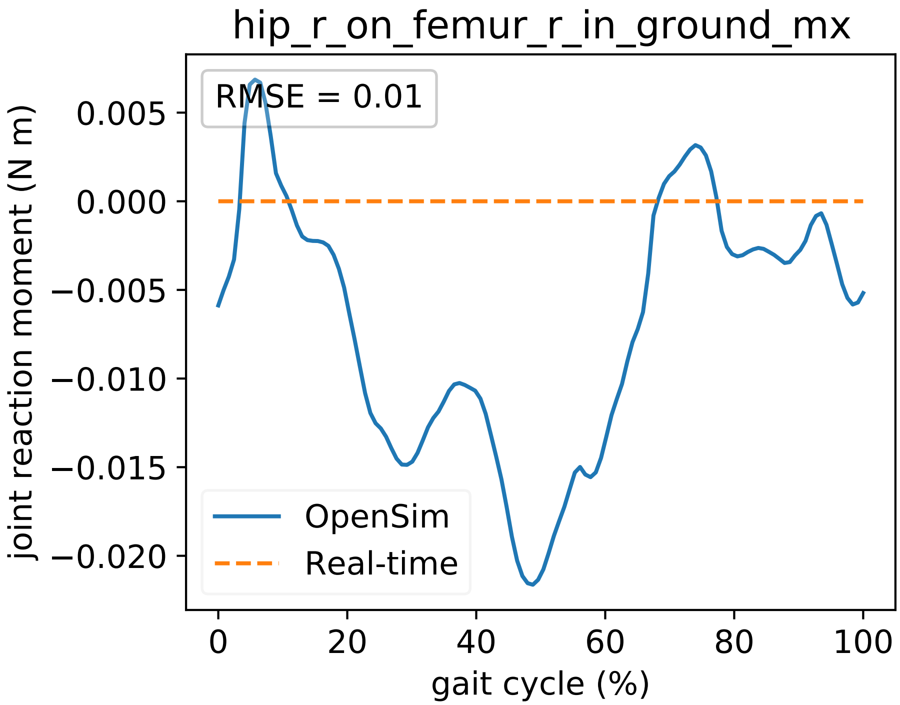

hip_r_on_femur_r_in_ground_mx
RMSE = 0.01
joint reaction moment (N m)
0.005
0.000
−0.005
−0.010
−0.015
−0.020
OpenSim
Real-time
0
20
40
60
80
100
gait cycle (%)

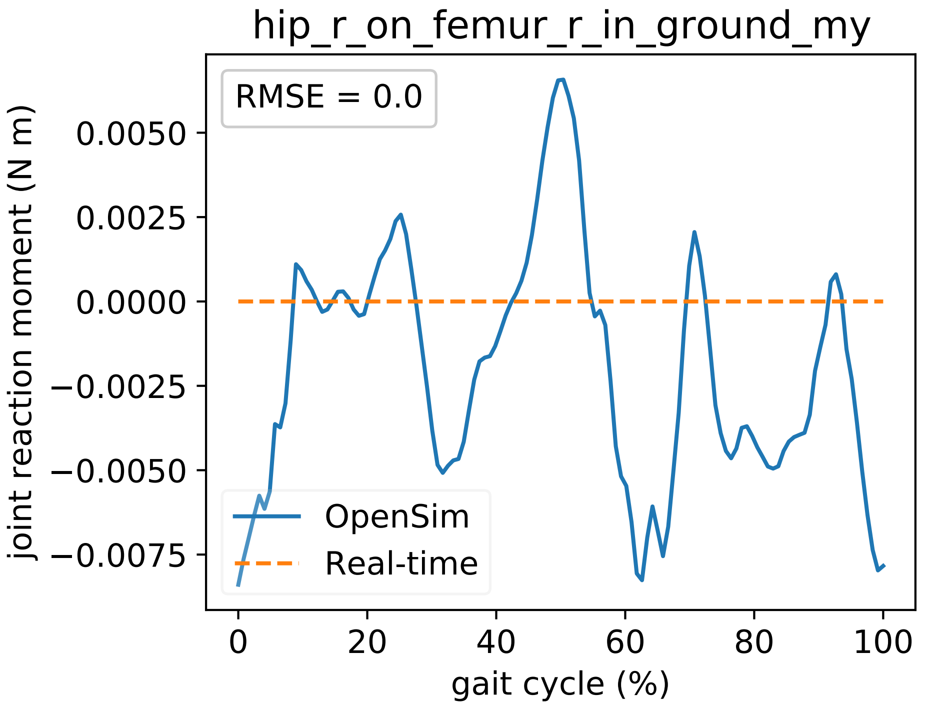
hip_r_on_femur_r_in_ground_my
RMSE = 0.0
joint reaction moment (N m)
0.0050
0.0025
0.0000
−0.0025
−0.0050
−0.0075
OpenSim
Real-time
0
20
40
60
80
100
gait cycle (%)

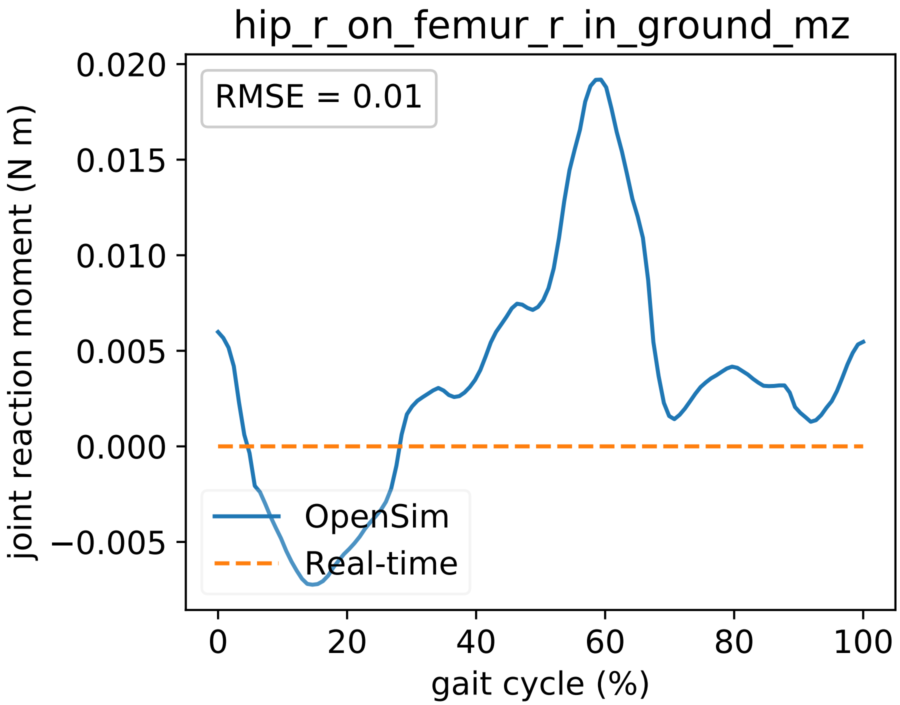
hip_r_on_femur_r_in_ground_mz
RMSE = 0.01
joint reaction moment (N m)
0.020
0.015
0.010
0.005
0.000
−0.005
OpenSim
Real-time
0
20
40
60
80
100
gait cycle (%)

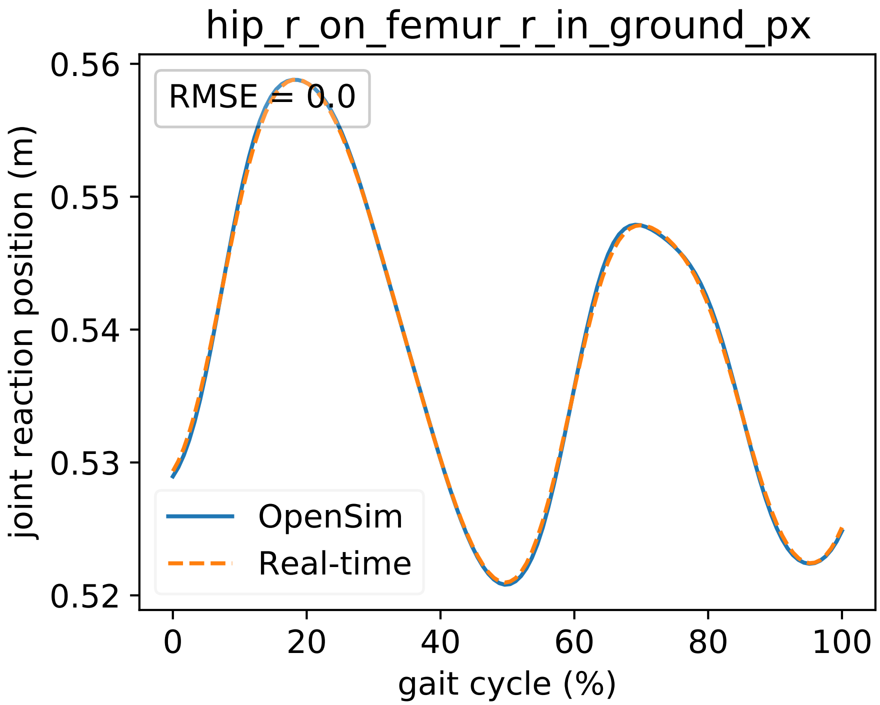

hip_r_on_femur_r_in_ground_px
RMSE = 0.0
OpenSim
Real-time
joint reaction position (m)
gait cycle (%)
0.56
0.55
0.54
0.53
0.52
0
20
40
60
80
100

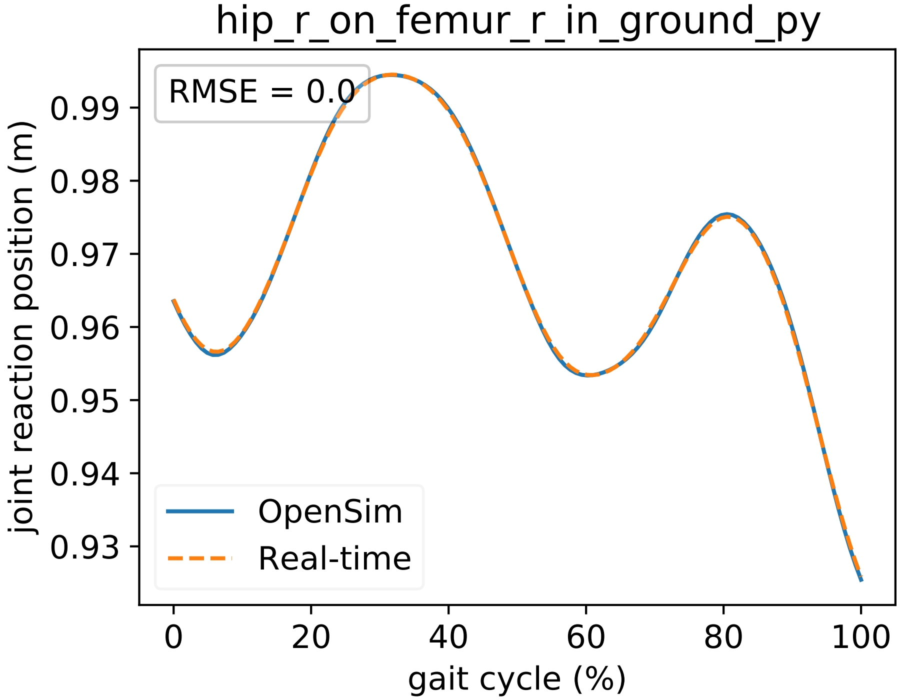
hip_r_on_femur_r_in_ground_py
RMSE = 0.0
OpenSim
Real-time
joint reaction position (m)
gait cycle (%)
0.99
0.98
0.97
0.96
0.95
0.94
0.93
0
20
40
60
80
100

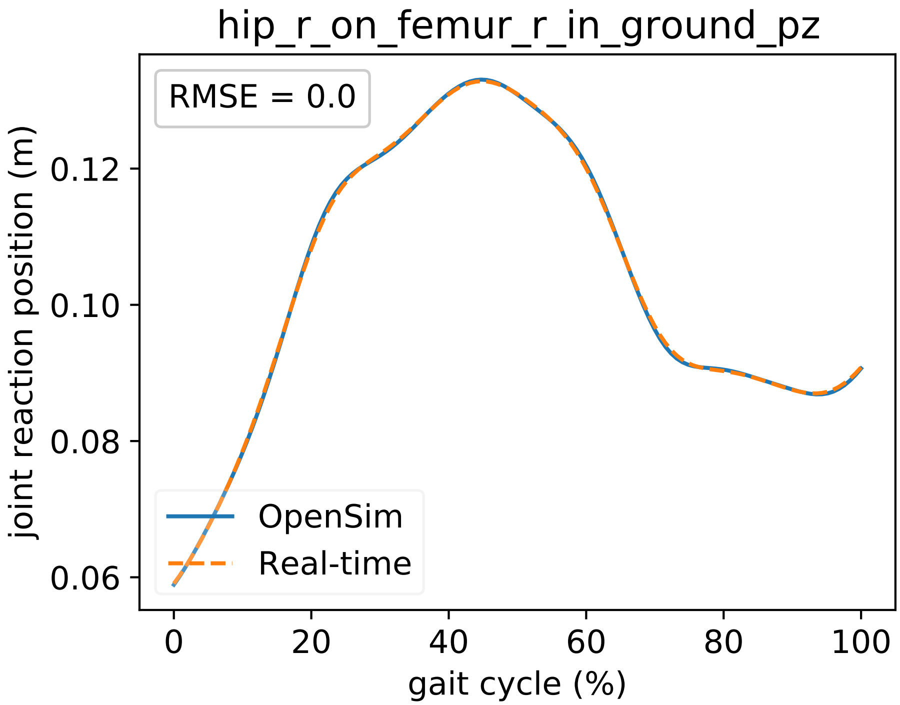
hip_r_on_femur_r_in_ground_pz
RMSE = 0.0
OpenSim
Real-time
joint reaction position (m)
0.06
0.08
0.10
0.12
0
20
40
60
80
100
gait cycle (%)

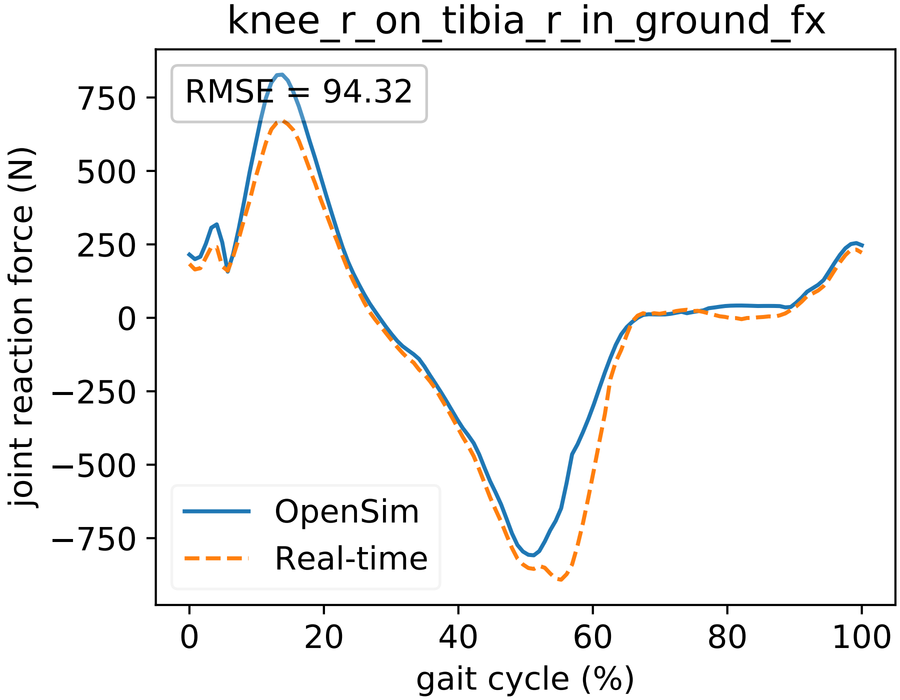
knee_r_on_tibia_r_in_ground_fx
RMSE = 94.32
750
500
250
0
−250
−500
−750
joint reaction force (N)
OpenSim
Real-time
0
20
40
60
80
100
gait cycle (%)

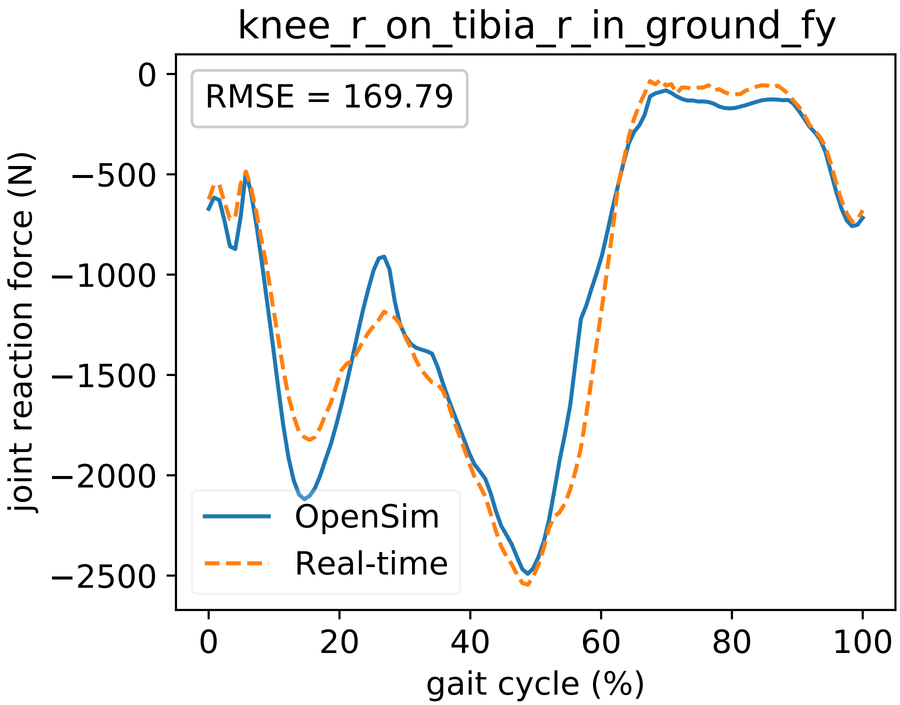
knee_r_on_tibia_r_in_ground_fy
RMSE = 169.79
OpenSim
Real-time
joint reaction force (N)
gait cycle (%)
0
−500
−1000
−1500
−2000
−2500
0
20
40
60
80
100

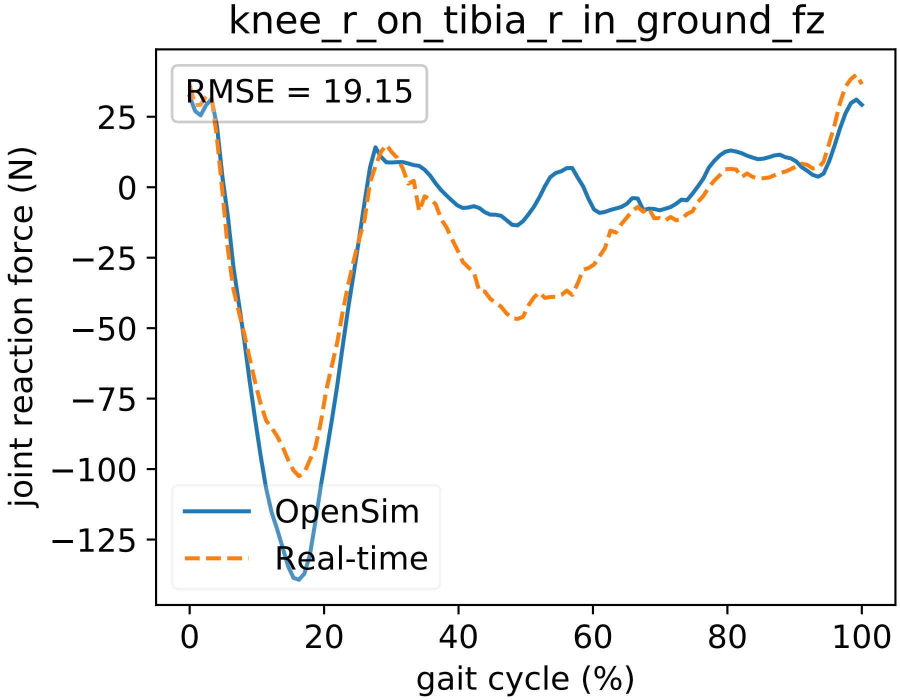
knee_r_on_tibia_r_in_ground_fz
RMSE = 19.15
OpenSim
Real-time
joint reaction force (N)
gait cycle (%)
25
0
−25
−50
−75
−100
−125
0
20
40
60
80
100

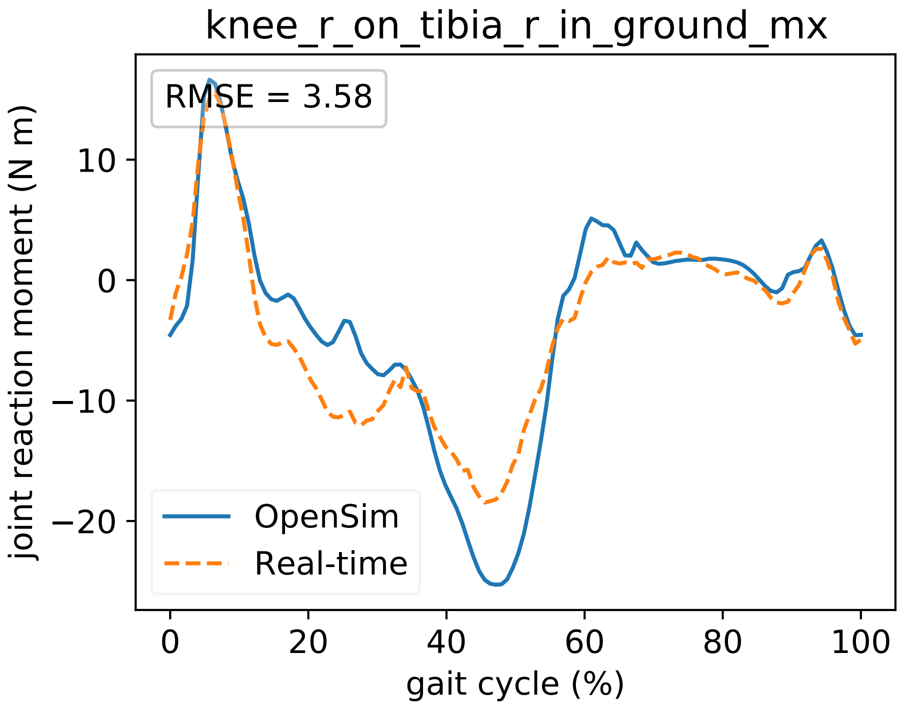

knee_r_on_tibia_r_in_ground_mx
RMSE = 3.58
OpenSim
Real-time
joint reaction moment (N m)
gait cycle (%)
10
0
−10
−20
0
20
40
60
80
100

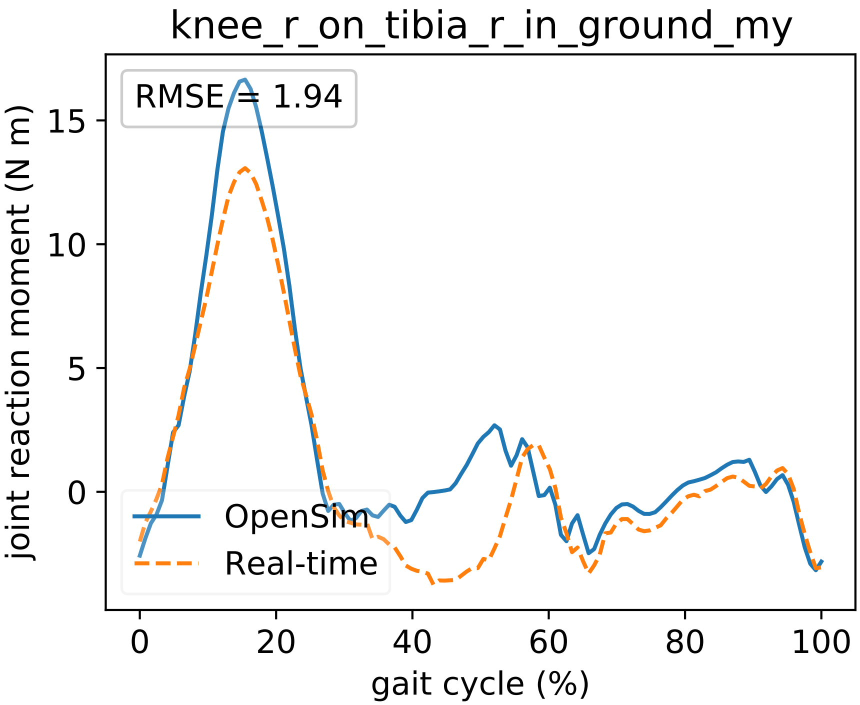

knee_r_on_tibia_r_in_ground_my
RMSE = 1.94
joint reaction moment (N m)
15
10
5
0
0
20
40
60
80
100
gait cycle (%)
OpenSim
Real-time

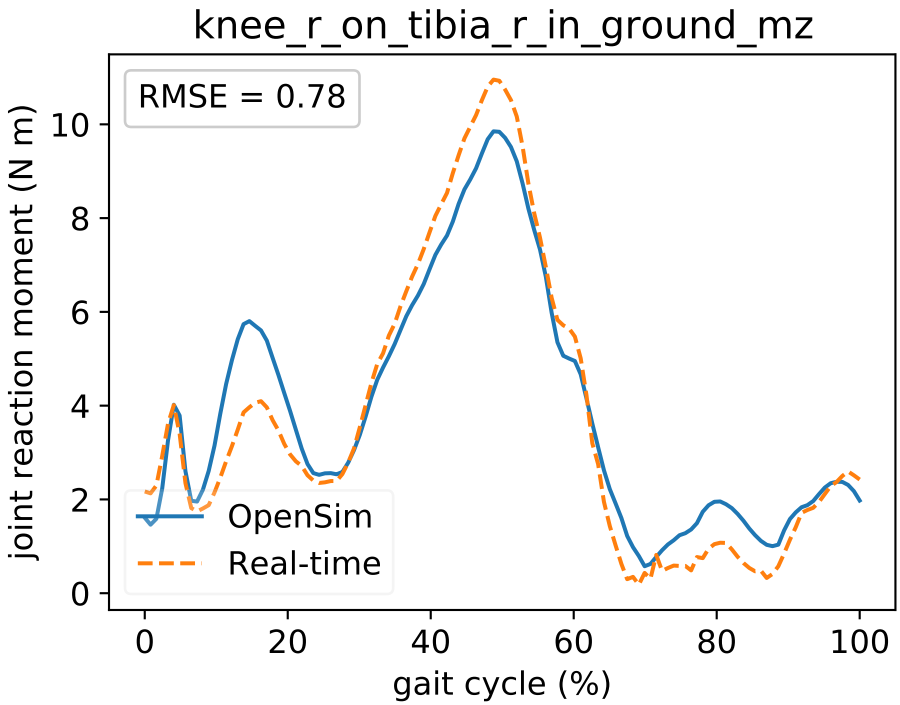

knee_r_on_tibia_r_in_ground_mz
RMSE = 0.78
OpenSim
Real-time
joint reaction moment (N m)
gait cycle (%)
0
2
4
6
8
10
0
20
40
60
80
100

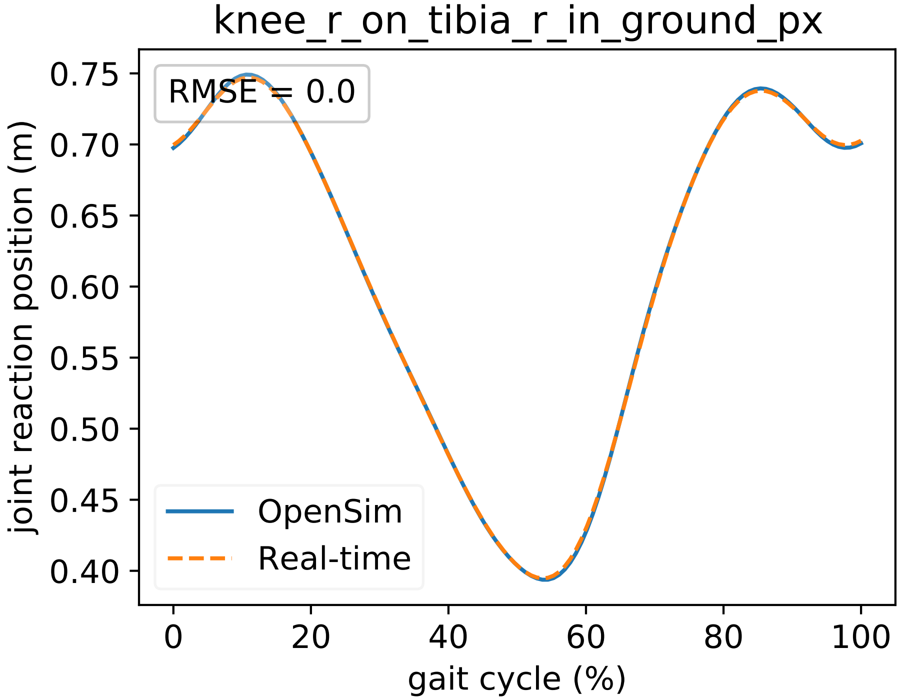

knee_r_on_tibia_r_in_ground_px
RMSE = 0.0
joint reaction position (m)
0.75
0.70
0.65
0.60
0.55
0.50
0.45
0.40
0
20
40
60
80
100
gait cycle (%)
OpenSim
Real-time

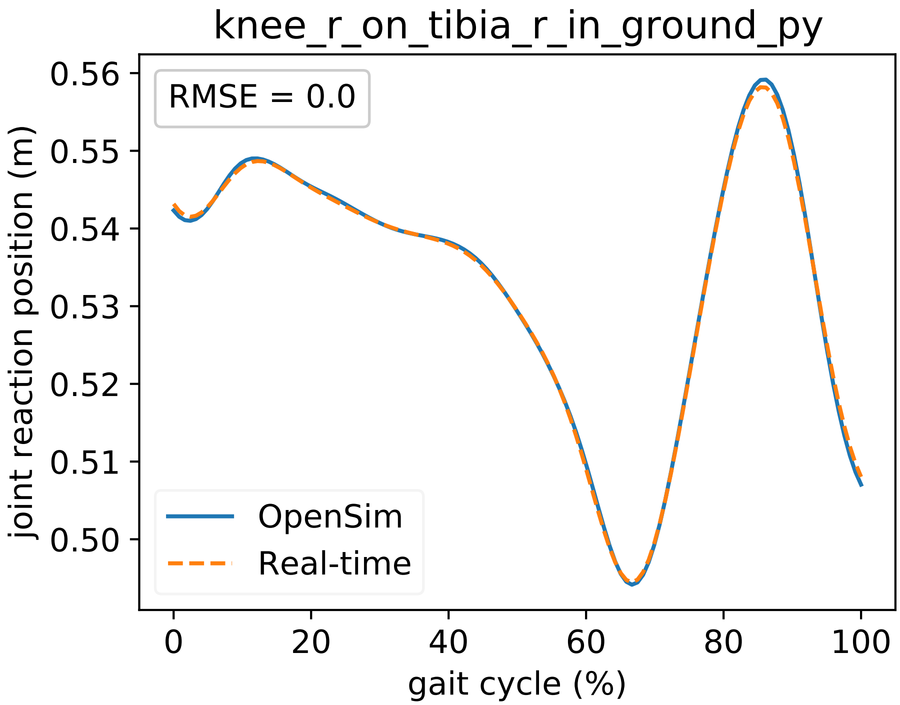
knee_r_on_tibia_r_in_ground_py
RMSE = 0.0
OpenSim
Real-time
joint reaction position (m)
0.56
0.55
0.54
0.53
0.52
0.51
0.50
0
20
40
60
80
100
gait cycle (%)

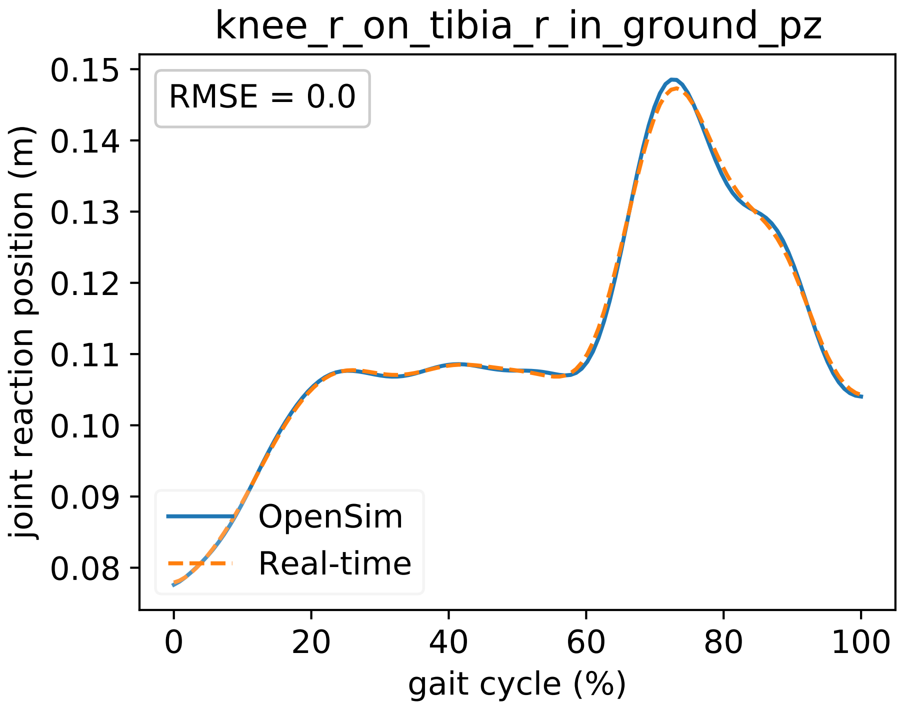
knee_r_on_tibia_r_in_ground_pz
RMSE = 0.0
joint reaction position (m)
0.15
0.14
0.13
0.12
0.11
0.10
0.09
0.08
OpenSim
Real-time
0
20
40
60
80
100
gait cycle (%)

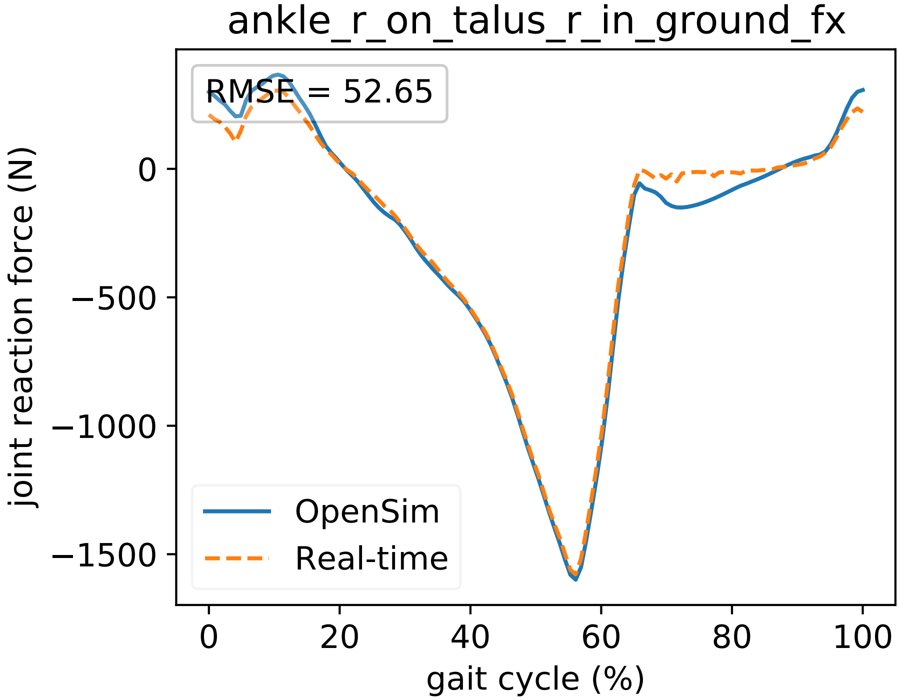
ankle_r_on_talus_r_in_ground_fx
RMSE = 52.65
joint reaction force (N)
0
−500
−1000
−1500
0
20
40
60
80
100
gait cycle (%)
OpenSim
Real-time

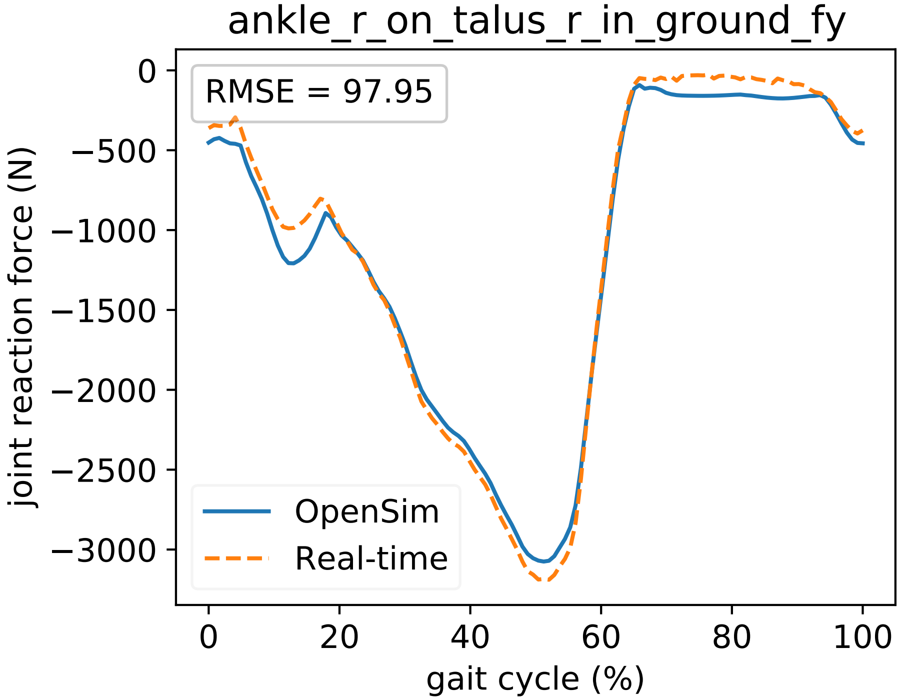
ankle_r_on_talus_r_in_ground_fy
RMSE = 97.95
OpenSim
Real-time
joint reaction force (N)
gait cycle (%)
0
−500
−1000
−1500
−2000
−2500
−3000
0
20
40
60
80
100

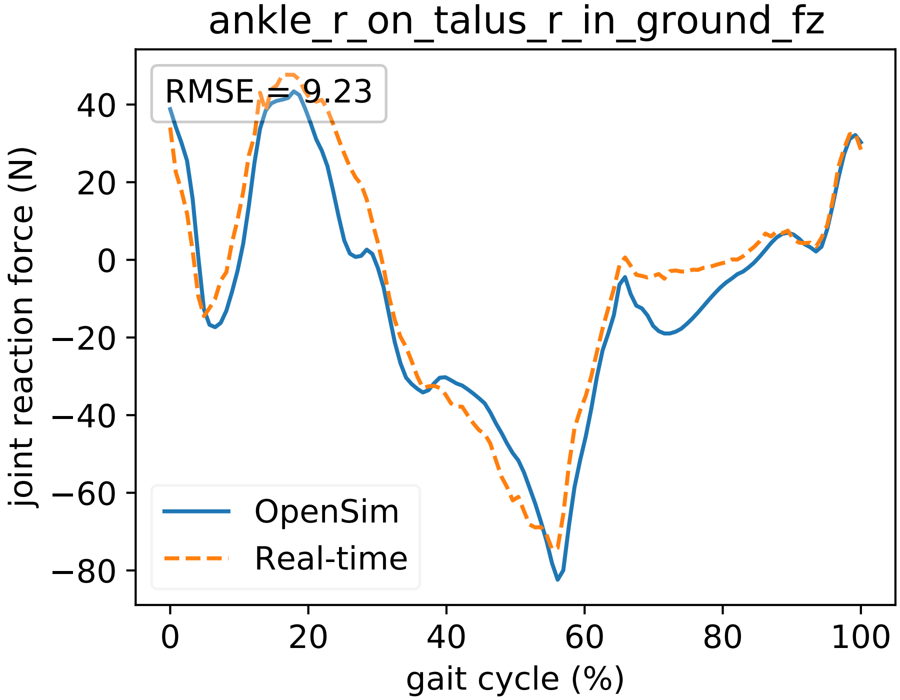

ankle_r_on_talus_r_in_ground_fz
RMSE = 9.23
joint reaction force (N)
40
20
0
−20
−40
−60
−80
OpenSim
Real-time
0
20
40
60
80
100
gait cycle (%)

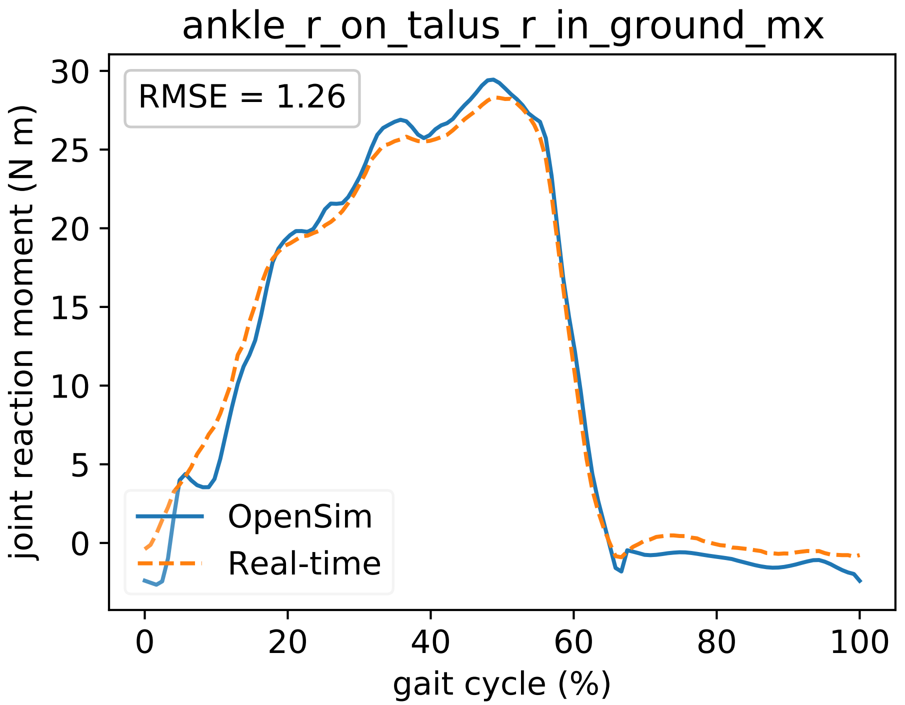
ankle_r_on_talus_r_in_ground_mx
RMSE = 1.26
joint reaction moment (N m)
gait cycle (%)
OpenSim
Real-time
0
5
10
15
20
25
30
0
20
40
60
80
100

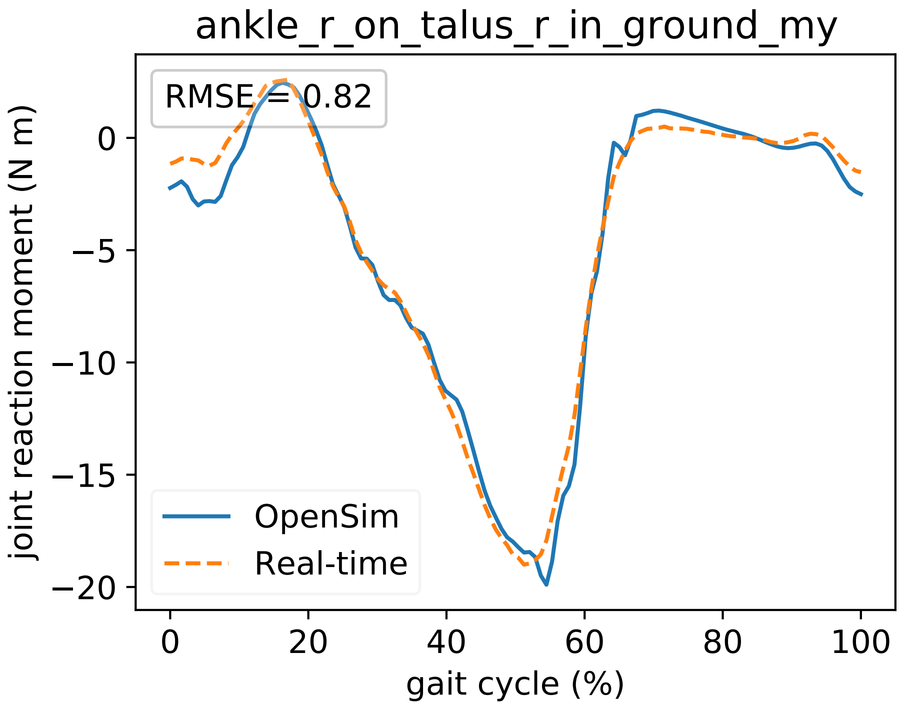

ankle_r_on_talus_r_in_ground_my
RMSE = 0.82
OpenSim
Real-time
joint reaction moment (N m)
gait cycle (%)
0
−5
−10
−15
−20
0
20
40
60
80
100

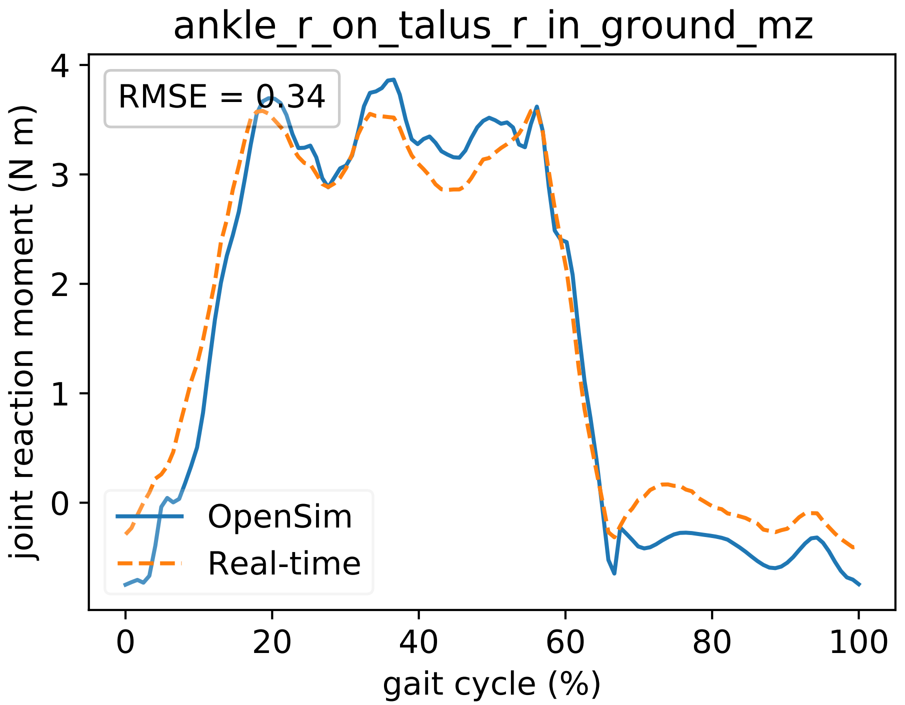

ankle_r_on_talus_r_in_ground_mz
RMSE = 0.34
OpenSim
Real-time
joint reaction moment (N m)
gait cycle (%)
4
3
2
1
0
0
20
40
60
80
100

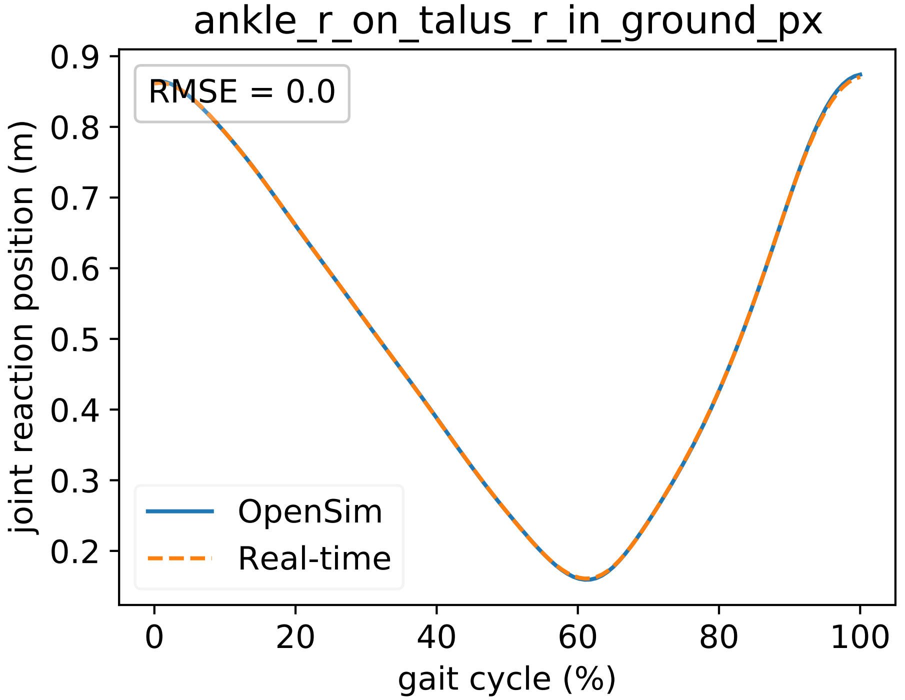
ankle_r_on_talus_r_in_ground_px
RMSE = 0.0
OpenSim
Real-time
joint reaction position (m)
gait cycle (%)
0.9
0.8
0.7
0.6
0.5
0.4
0.3
0.2
0
20
40
60
80
100

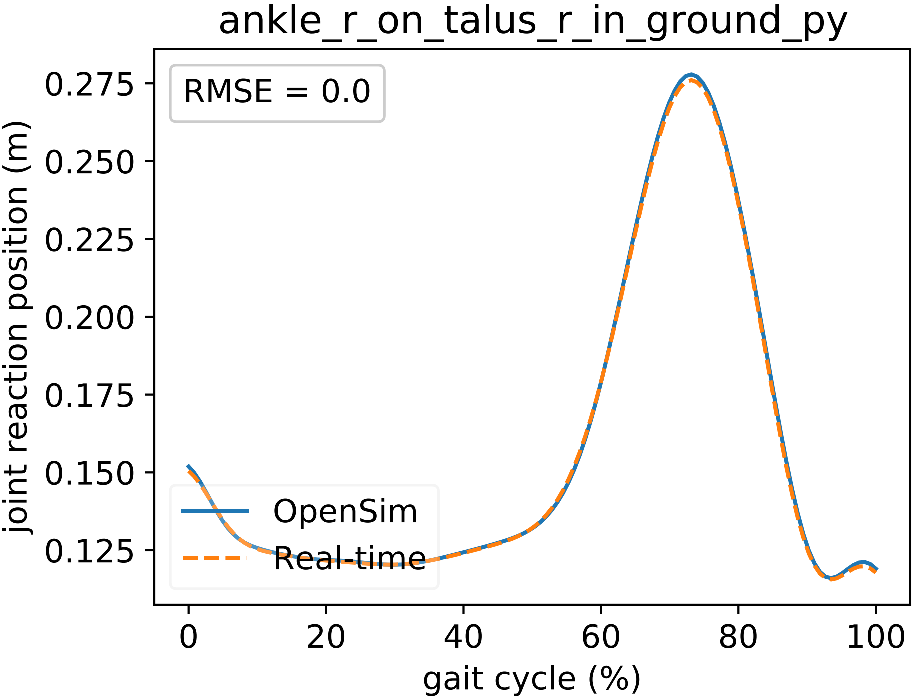
ankle_r_on_talus_r_in_ground_py
RMSE = 0.0
OpenSim
Real-time
joint reaction position (m)
0.275
0.250
0.225
0.200
0.175
0.150
0.125
0
20
40
60
80
100
gait cycle (%)

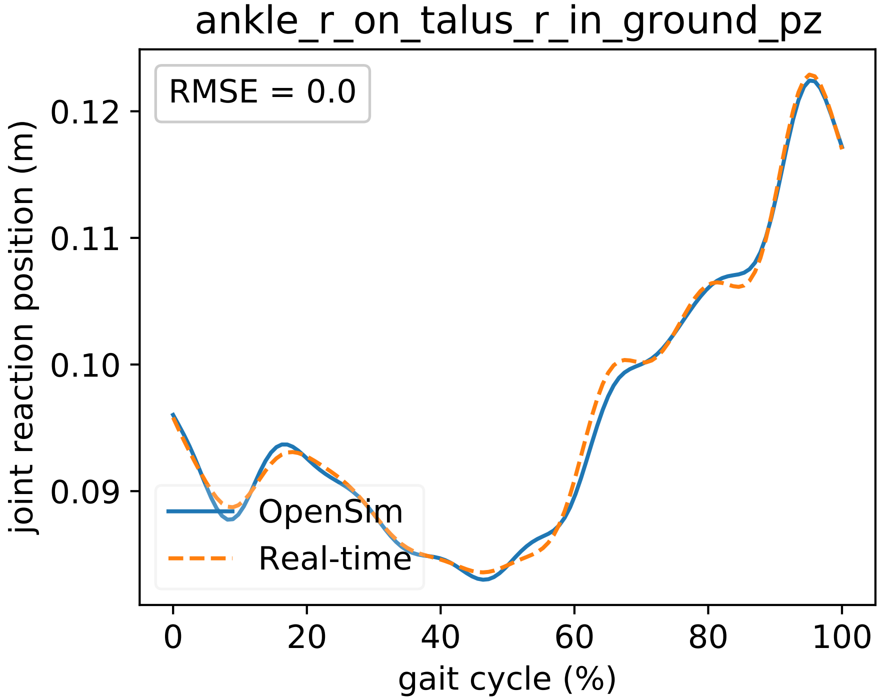
ankle_r_on_talus_r_in_ground_pz
RMSE = 0.0
OpenSim
Real-time
joint reaction position (m)
0.09
0.10
0.11
0.12
0
20
40
60
80
100
gait cycle (%)

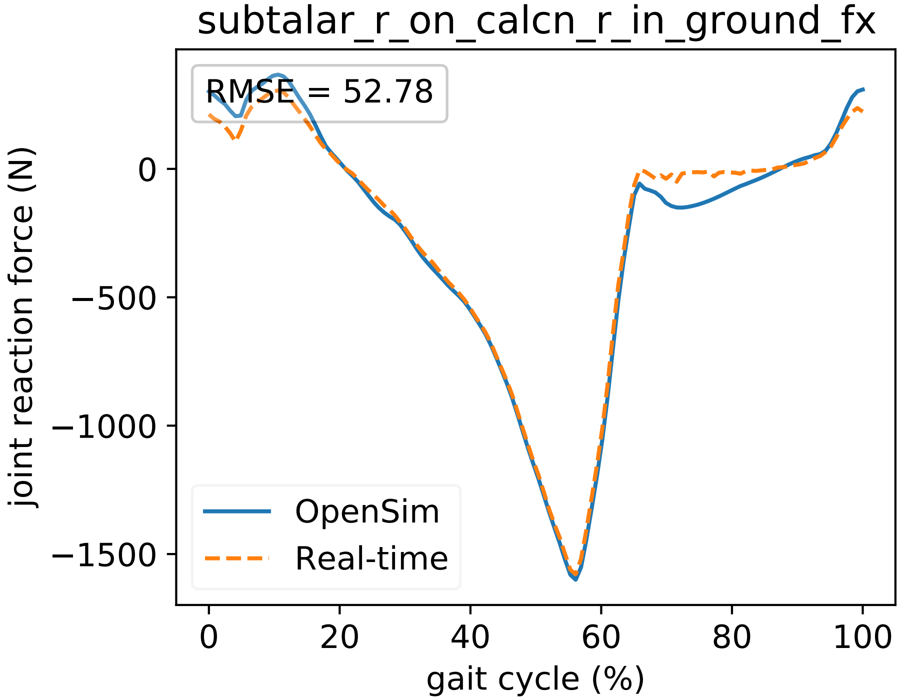
subtalar_r_on_calcn_r_in_ground_fx
RMSE = 52.78
OpenSim
Real-time
joint reaction force (N)
0
−500
−1000
−1500
0
20
40
60
80
100
gait cycle (%)

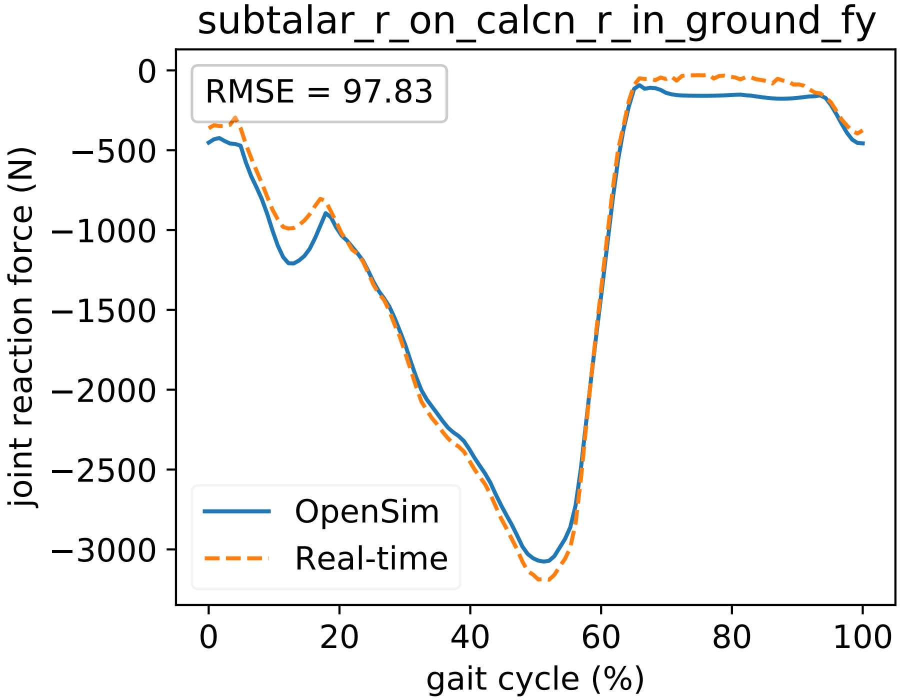

subtalar_r_on_calcn_r_in_ground_fy
RMSE = 97.83
0
−500
−1000
−1500
−2000
−2500
−3000
joint reaction force (N)
OpenSim
Real-time
0
20
40
60
80
100
gait cycle (%)

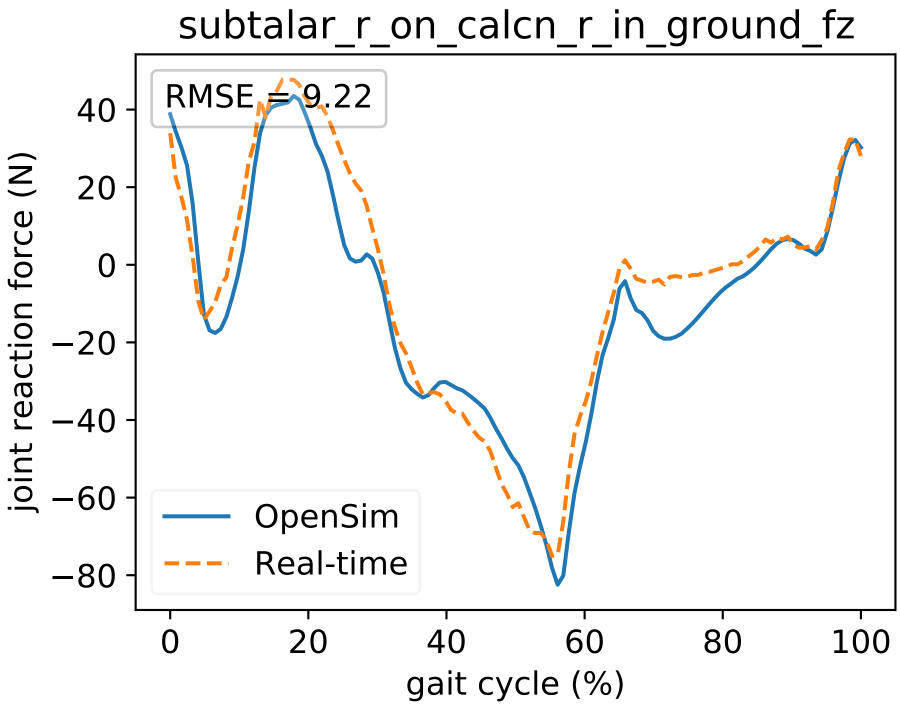

subtalar_r_on_calcn_r_in_ground_fz
RMSE = 9.22
joint reaction force (N)
40
20
0
−20
−40
−60
−80
OpenSim
Real-time
0
20
40
60
80
100
gait cycle (%)

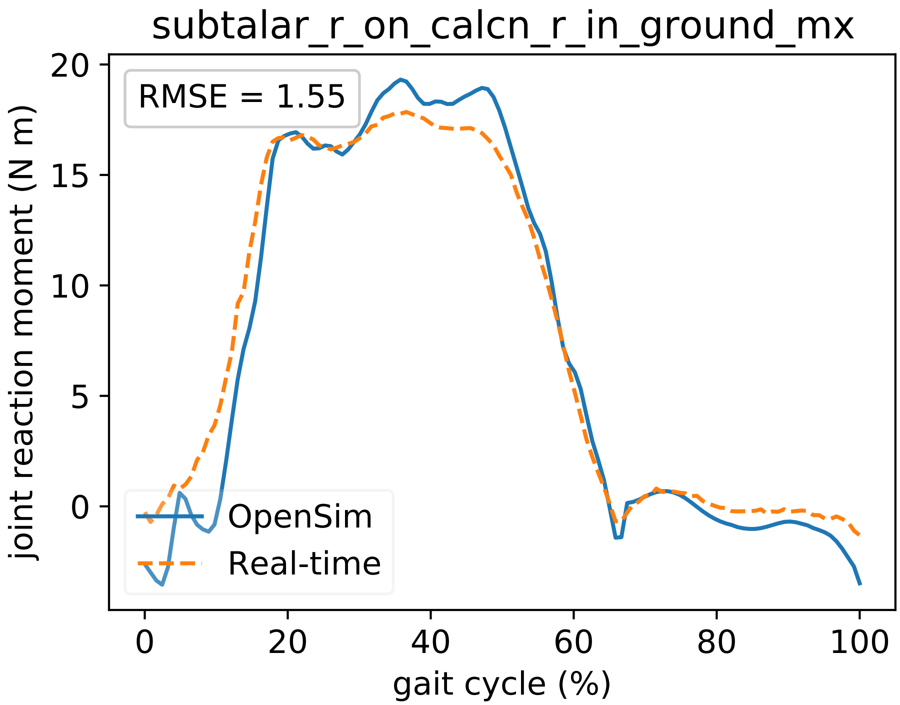
subtalar_r_on_calcn_r_in_ground_mx
RMSE = 1.55
OpenSim
Real-time
joint reaction moment (N m)
gait cycle (%)
20
15
10
5
0
0
20
40
60
80
100

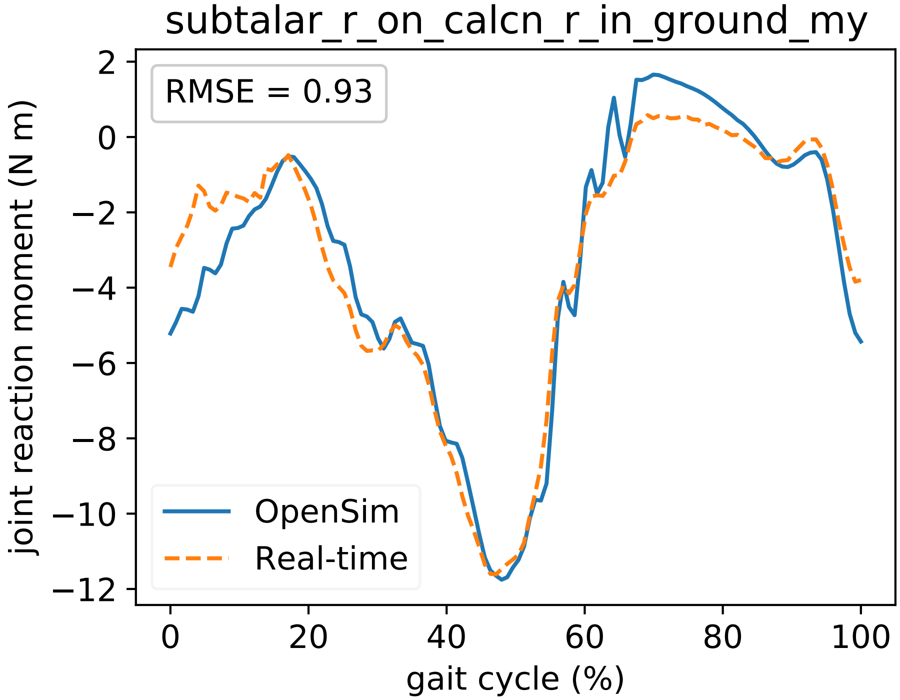
subtalar_r_on_calcn_r_in_ground_my
RMSE = 0.93
joint reaction moment (N m)
gait cycle (%)
OpenSim
Real-time
2
0
−2
−4
−6
−8
−10
−12
0
20
40
60
80
100

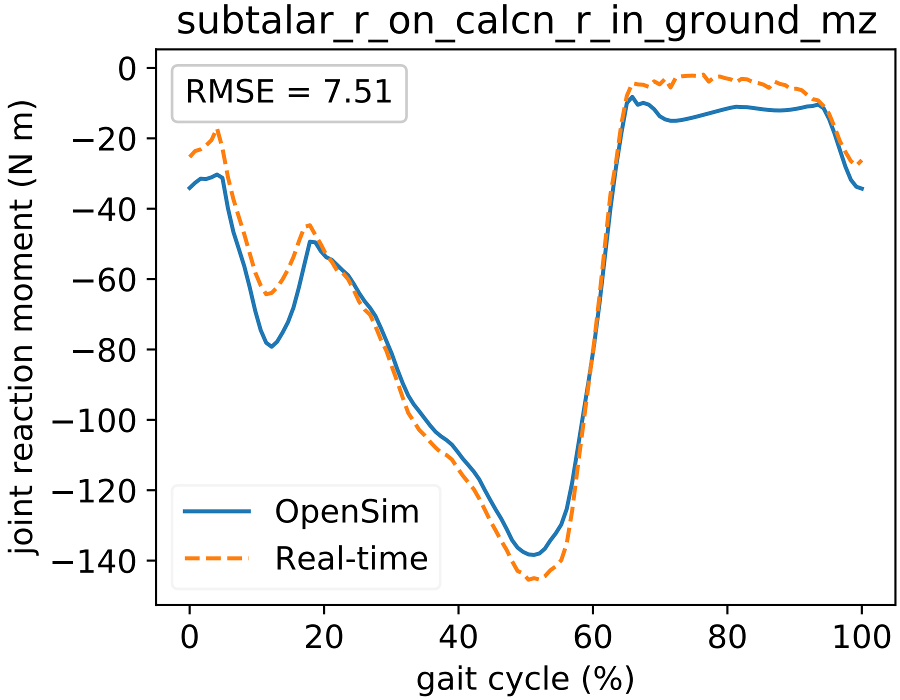
subtalar_r_on_calcn_r_in_ground_mz
RMSE = 7.51
OpenSim
Real-time
joint reaction moment (N m)
gait cycle (%)
0
−20
−40
−60
−80
−100
−120
−140
0
20
40
60
80
100

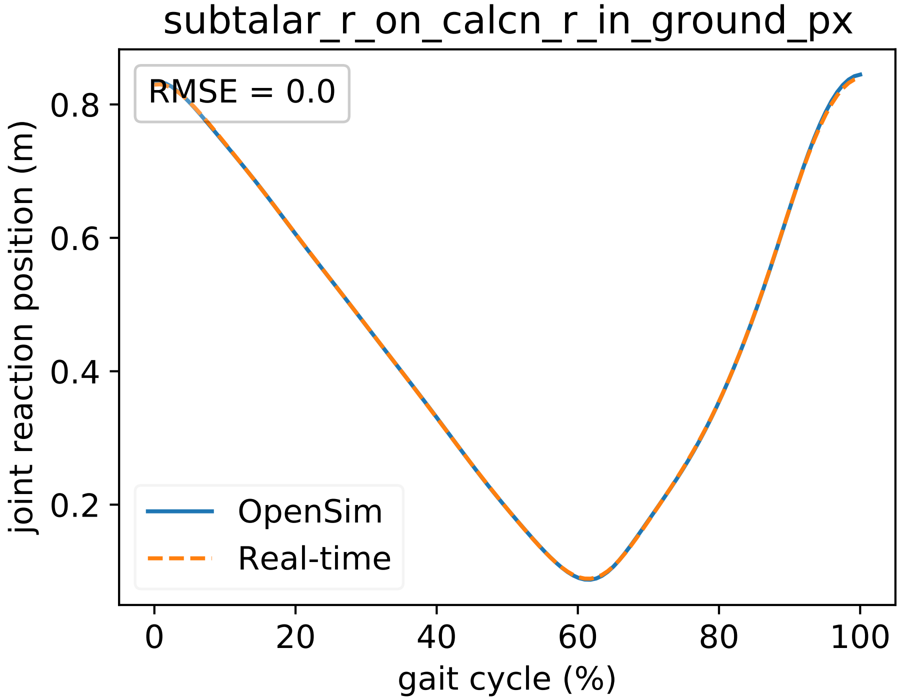

subtalar_r_on_calcn_r_in_ground_px
RMSE = 0.0
joint reaction position (m)
0.2
0.4
0.6
0.8
0
20
40
60
80
100
gait cycle (%)
OpenSim
Real-time

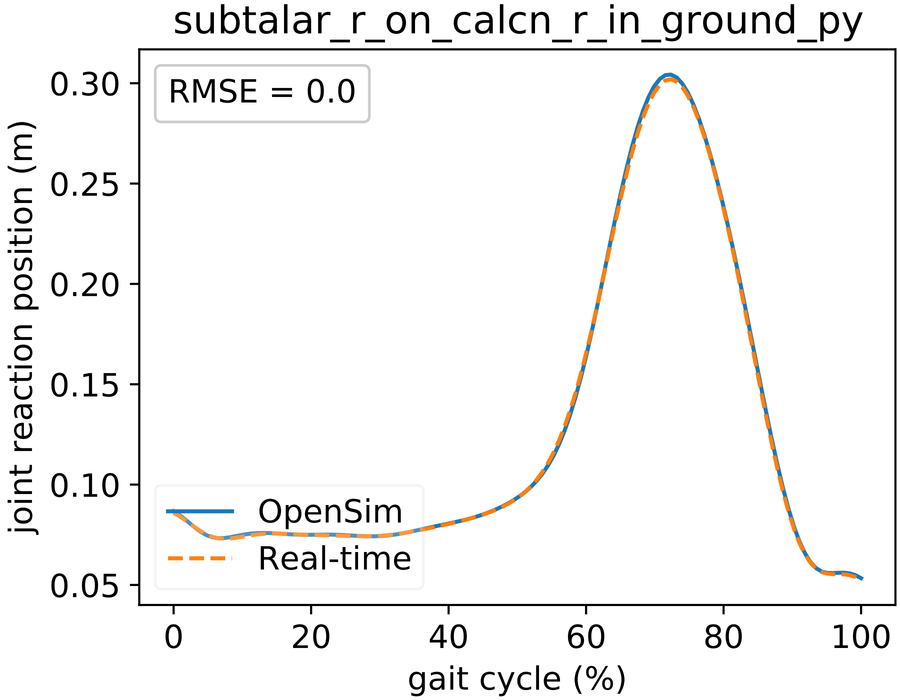

subtalar_r_on_calcn_r_in_ground_py
RMSE = 0.0
OpenSim
Real-time
joint reaction position (m)
0.30
0.25
0.20
0.15
0.10
0.05
0
20
40
60
80
100
gait cycle (%)

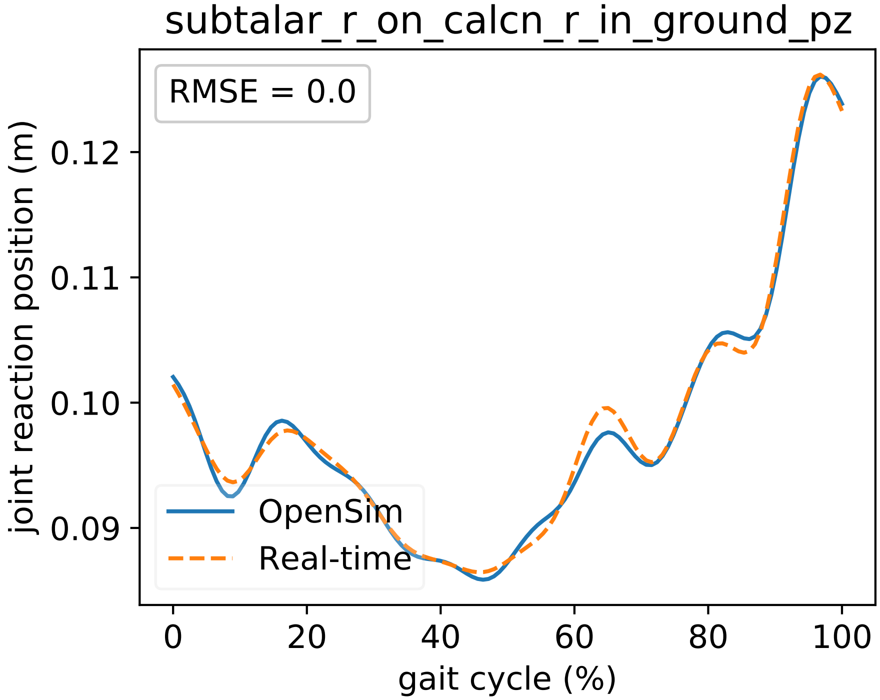
subtalar_r_on_calcn_r_in_ground_pz
RMSE = 0.0
OpenSim
Real-time
joint reaction position (m)
0.09
0.10
0.11
0.12
0
20
40
60
80
100
gait cycle (%)

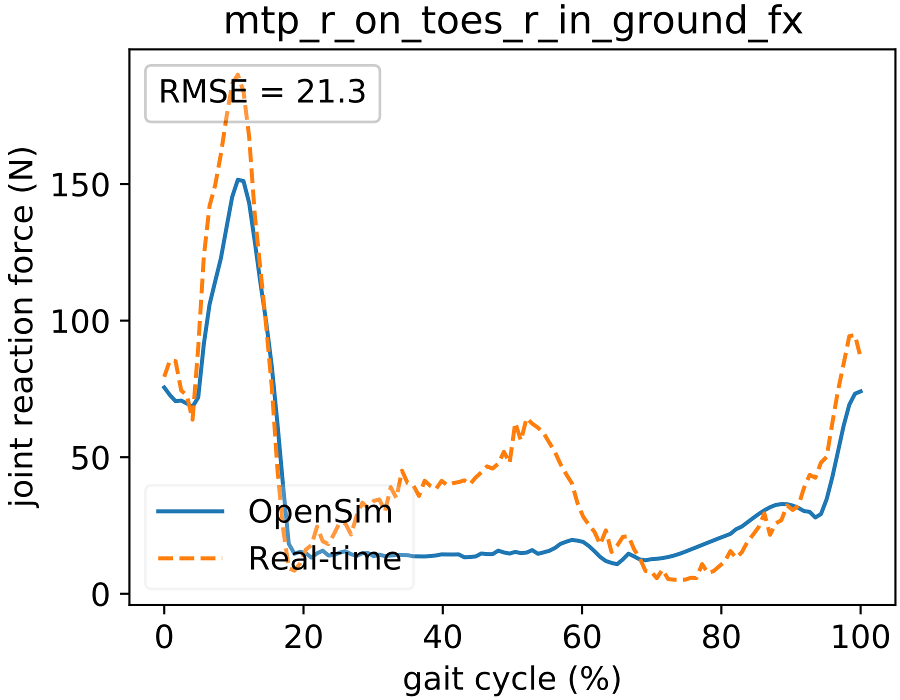

mtp_r_on_toes_r_in_ground_fx
RMSE = 21.3
joint reaction force (N)
0
50
100
150
0
20
40
60
80
100
gait cycle (%)
OpenSim
Real-time

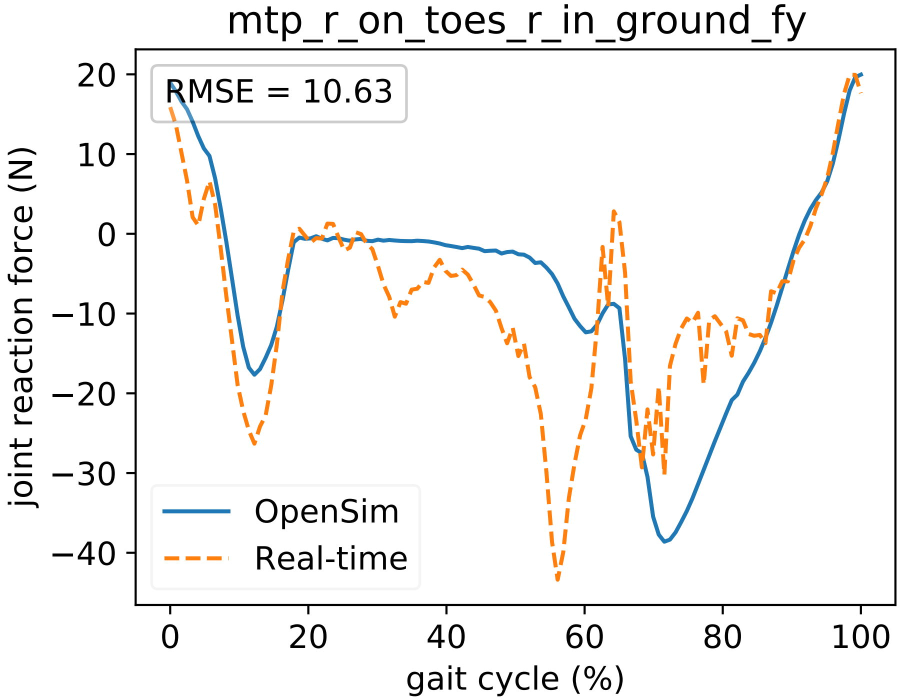
mtp_r_on_toes_r_in_ground_fy
RMSE = 10.63
OpenSim
Real-time
joint reaction force (N)
gait cycle (%)
20
10
0
−10
−20
−30
−40
0
20
40
60
80
100

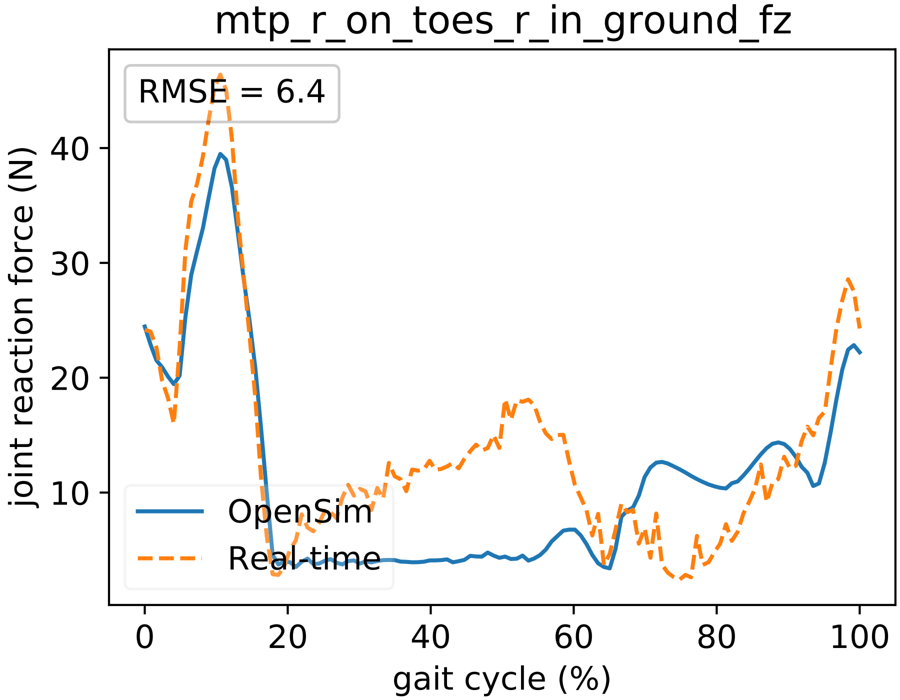
mtp_r_on_toes_r_in_ground_fz
RMSE = 6.4
joint reaction force (N)
10
20
30
40
OpenSim
Real-time
0
20
40
60
80
100
gait cycle (%)

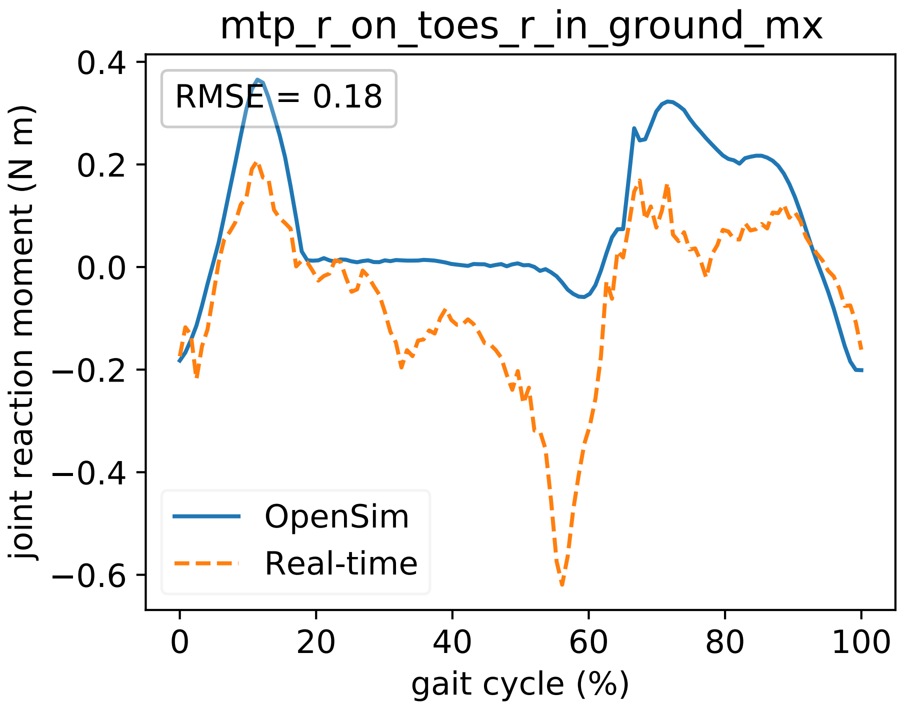

mtp_r_on_toes_r_in_ground_mx
RMSE = 0.18
OpenSim
Real-time
joint reaction moment (N m)
gait cycle (%)
0.4
0.2
0.0
−0.2
−0.4
−0.6
0
20
40
60
80
100

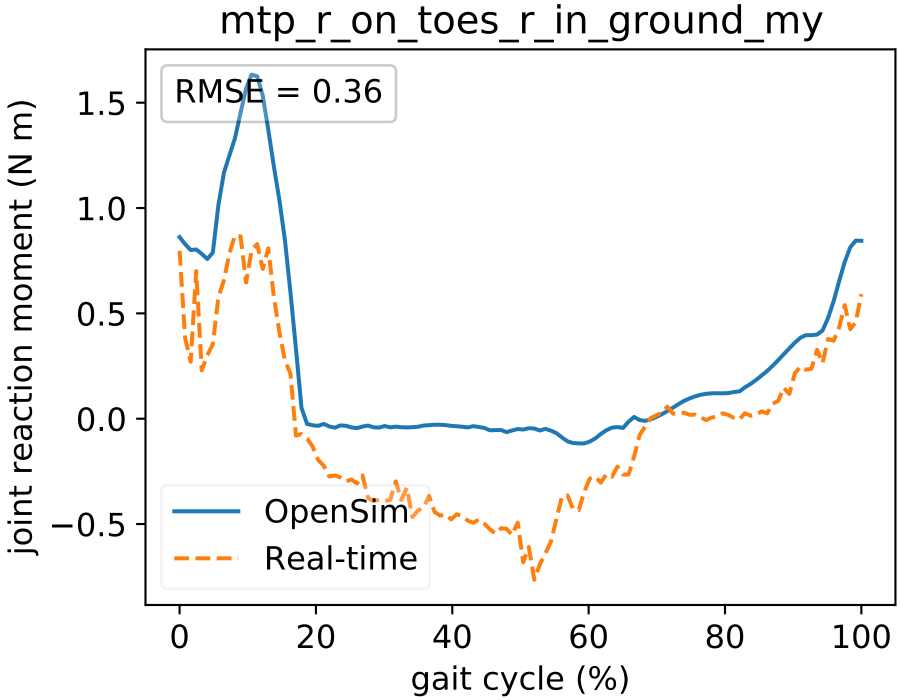

mtp_r_on_toes_r_in_ground_my
RMSE = 0.36
joint reaction moment (N m)
1.5
1.0
0.5
0.0
−0.5
OpenSim
Real-time
0
20
40
60
80
100
gait cycle (%)

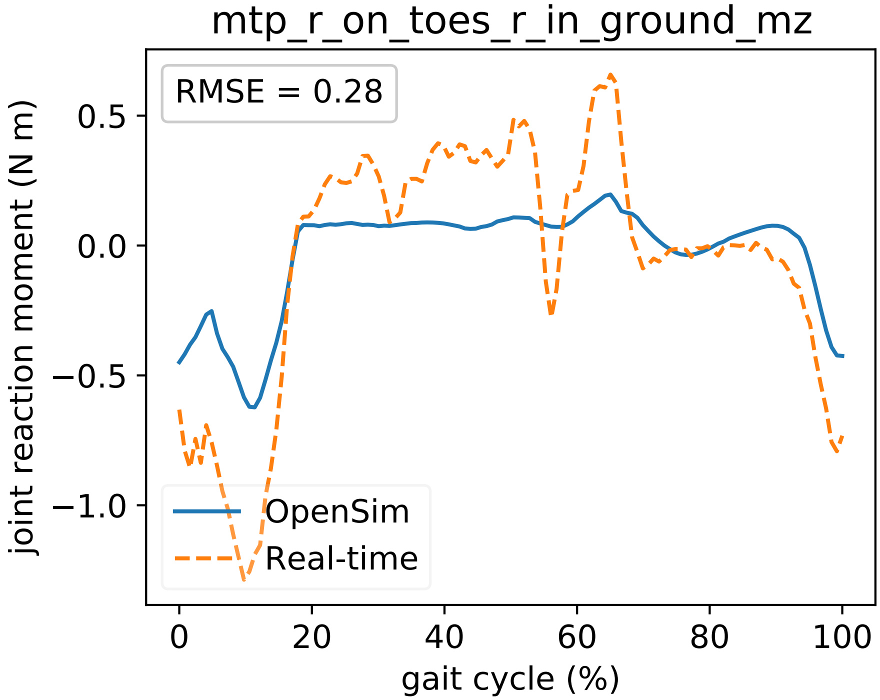

mtp_r_on_toes_r_in_ground_mz
RMSE = 0.28
OpenSim
Real-time
joint reaction moment (N m)
gait cycle (%)
0.5
0.0
−0.5
−1.0
0
20
40
60
80
100

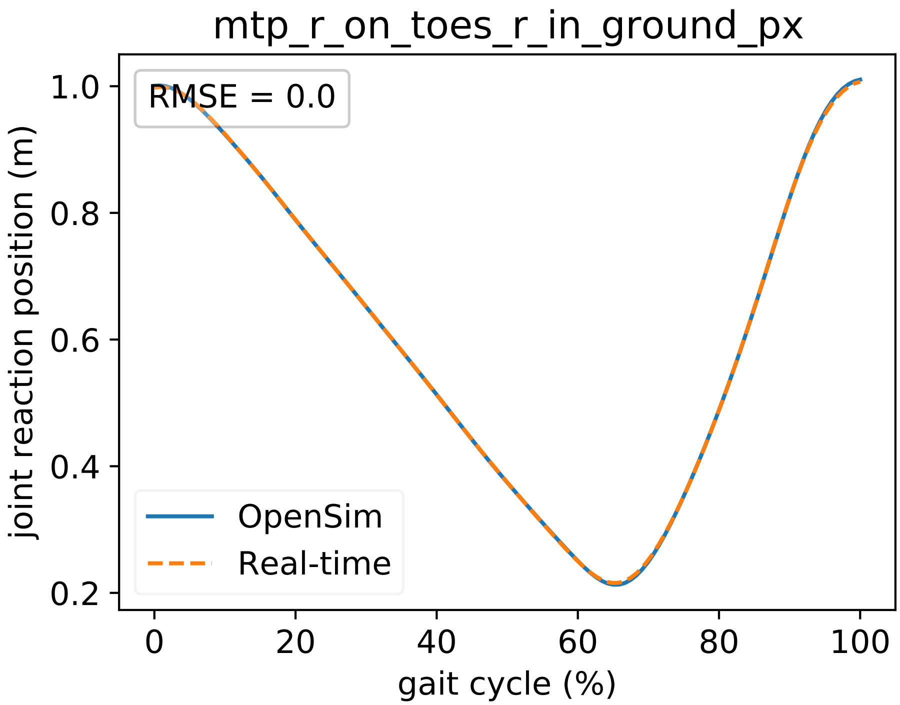

mtp_r_on_toes_r_in_ground_px
RMSE = 0.0
OpenSim
Real-time
joint reaction position (m)
gait cycle (%)
1.0
0.8
0.6
0.4
0.2
0
20
40
60
80
100

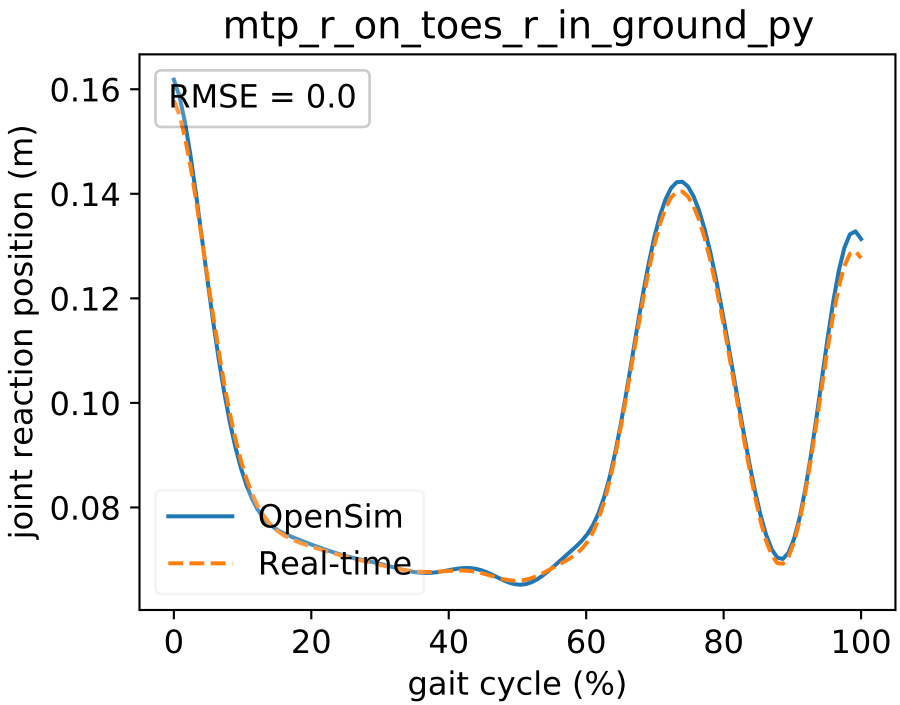

mtp_r_on_toes_r_in_ground_py
RMSE = 0.0
OpenSim
Real-time
joint reaction position (m)
gait cycle (%)
0.16
0.14
0.12
0.10
0.08
0
20
40
60
80
100

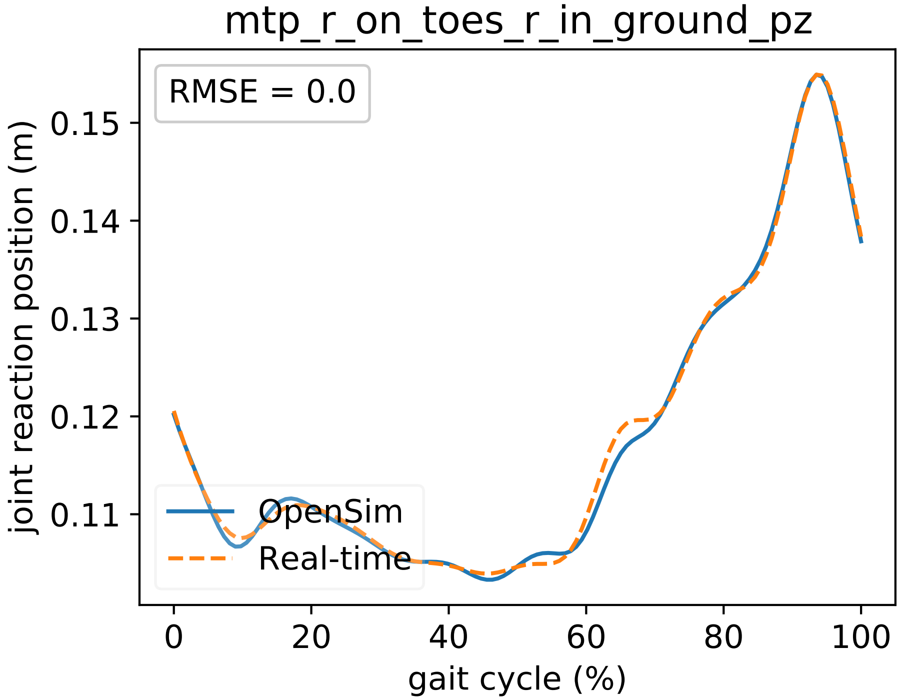
mtp_r_on_toes_r_in_ground_pz
RMSE = 0.0
OpenSim
Real-time
joint reaction position (m)
0.11
0.12
0.13
0.14
0.15
0
20
40
60
80
100
gait cycle (%)

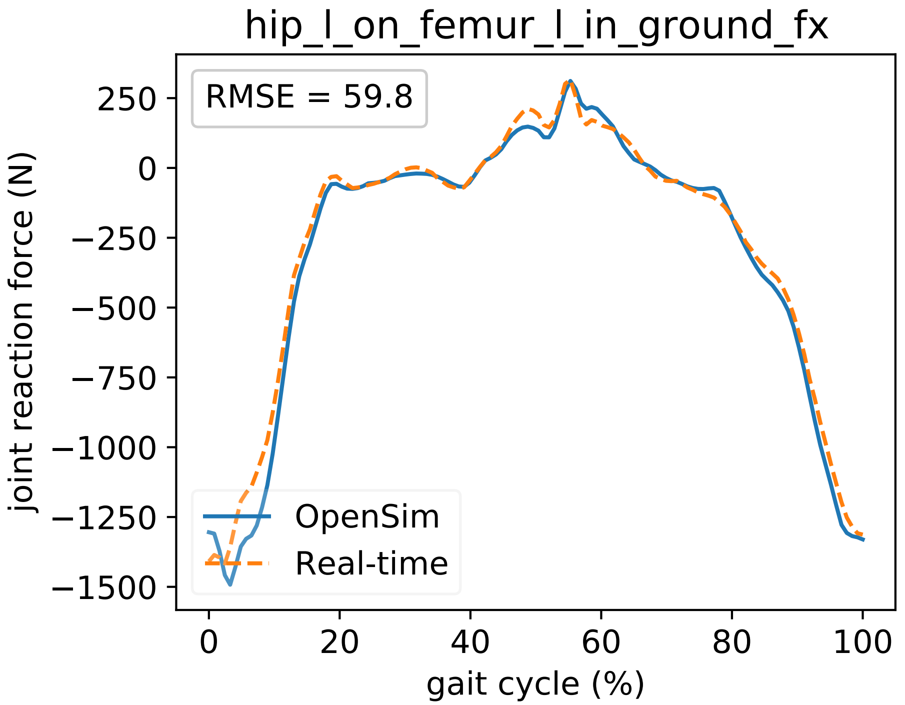

hip_l_on_femur_l_in_ground_fx
RMSE = 59.8
joint reaction force (N)
250
0
−250
−500
−750
−1000
−1250
−1500
0
20
40
60
80
100
gait cycle (%)
OpenSim
Real-time

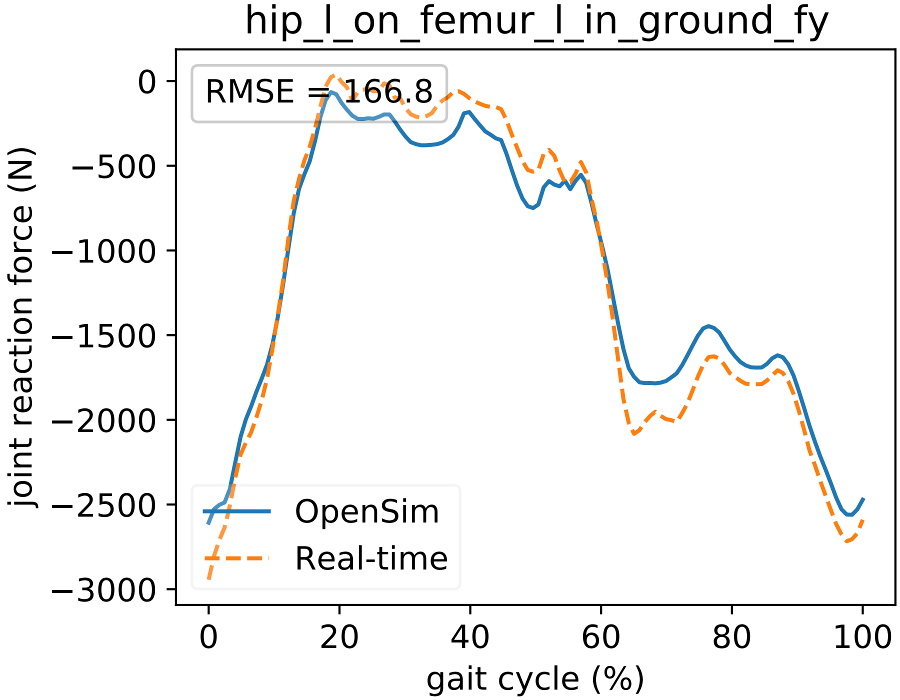
hip_l_on_femur_l_in_ground_fy
RMSE = 166.8
OpenSim
Real-time
joint reaction force (N)
gait cycle (%)
0
−500
−1000
−1500
−2000
−2500
−3000
0
20
40
60
80
100

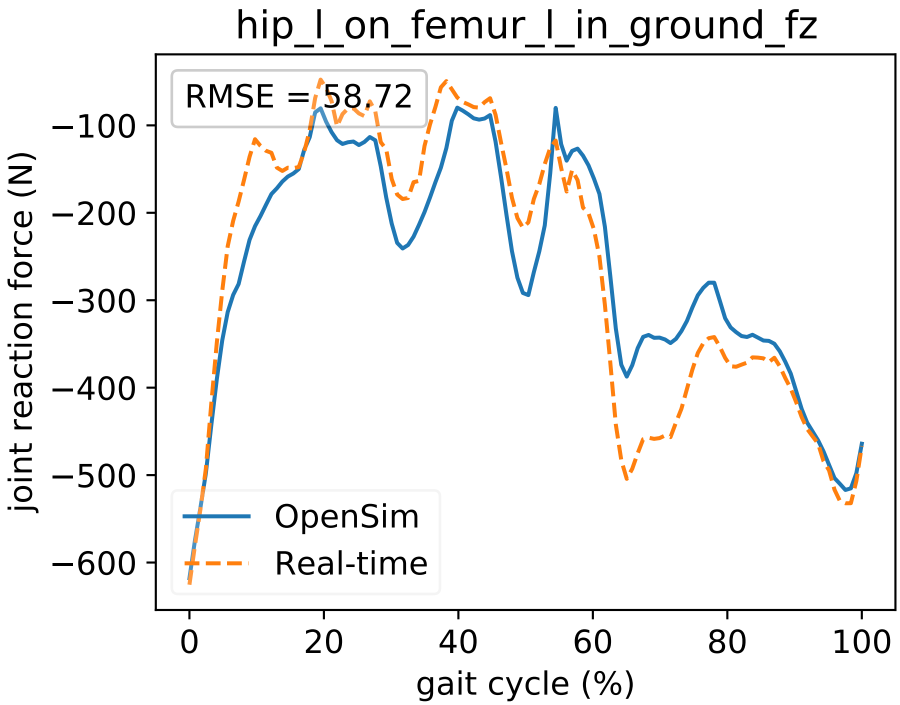
hip_l_on_femur_l_in_ground_fz
RMSE = 58.72
OpenSim
Real-time
joint reaction force (N)
gait cycle (%)
−100
−200
−300
−400
−500
−600
0
20
40
60
80
100

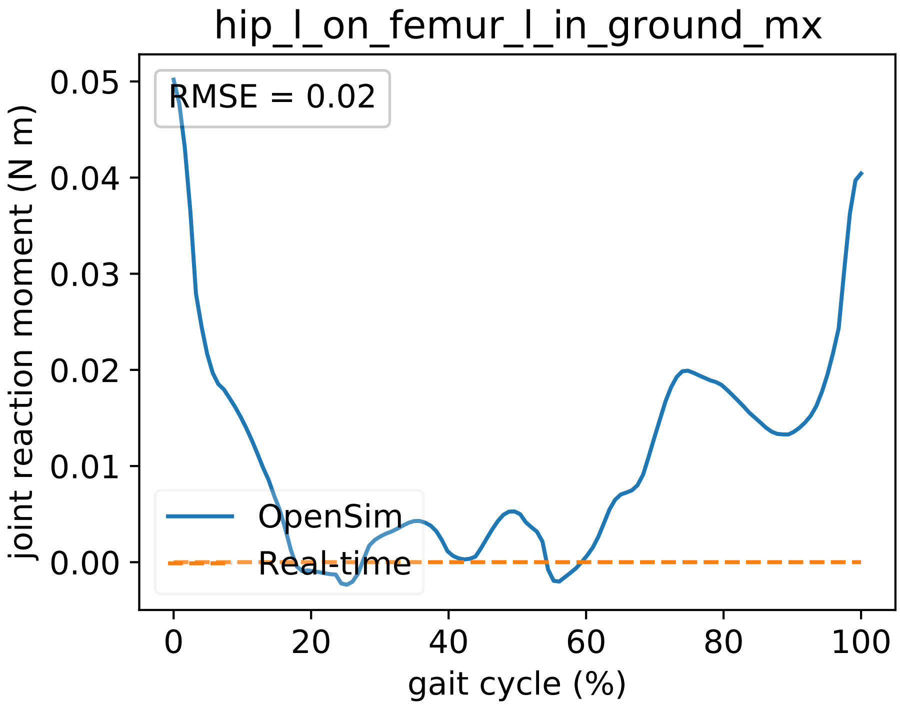
hip_l_on_femur_l_in_ground_mx
RMSE = 0.02
OpenSim
Real-time
joint reaction moment (N m)
0.05
0.04
0.03
0.02
0.01
0.00
0
20
40
60
80
100
gait cycle (%)

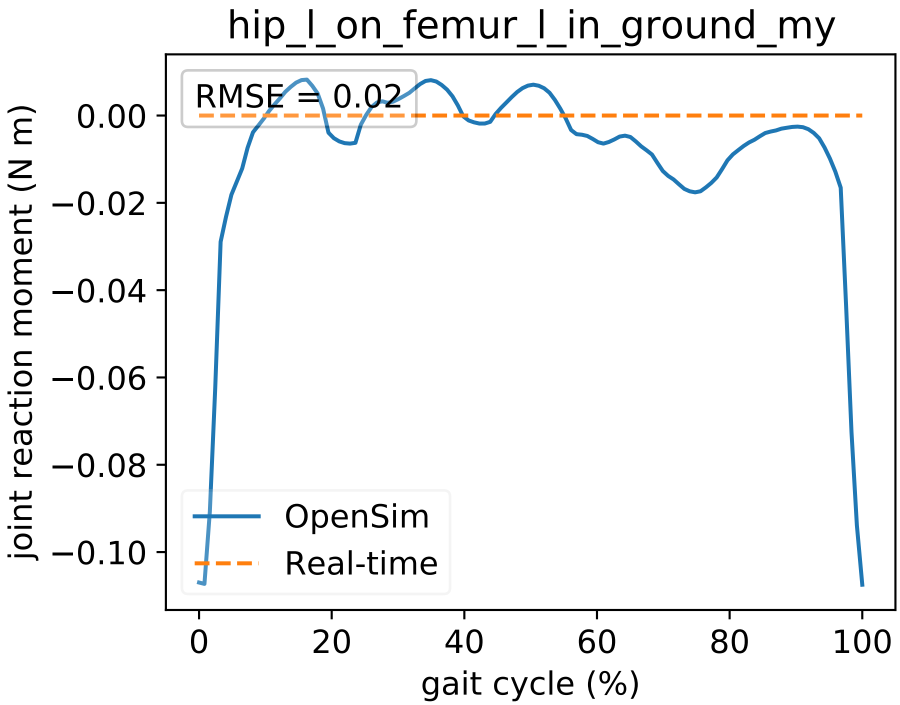
hip_l_on_femur_l_in_ground_my
RMSE = 0.02
joint reaction moment (N m)
0.00
−0.02
−0.04
−0.06
−0.08
−0.10
0
20
40
60
80
100
gait cycle (%)
OpenSim
Real-time

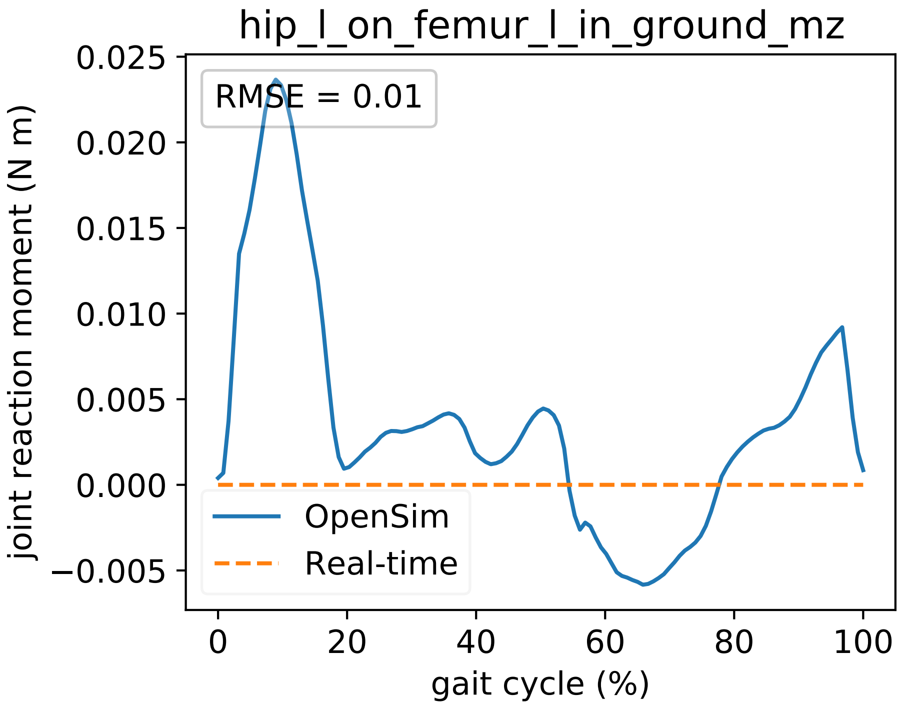
hip_l_on_femur_l_in_ground_mz
RMSE = 0.01
joint reaction moment (N m)
0.025
0.020
0.015
0.010
0.005
0.000
−0.005
OpenSim
Real-time
0
20
40
60
80
100
gait cycle (%)

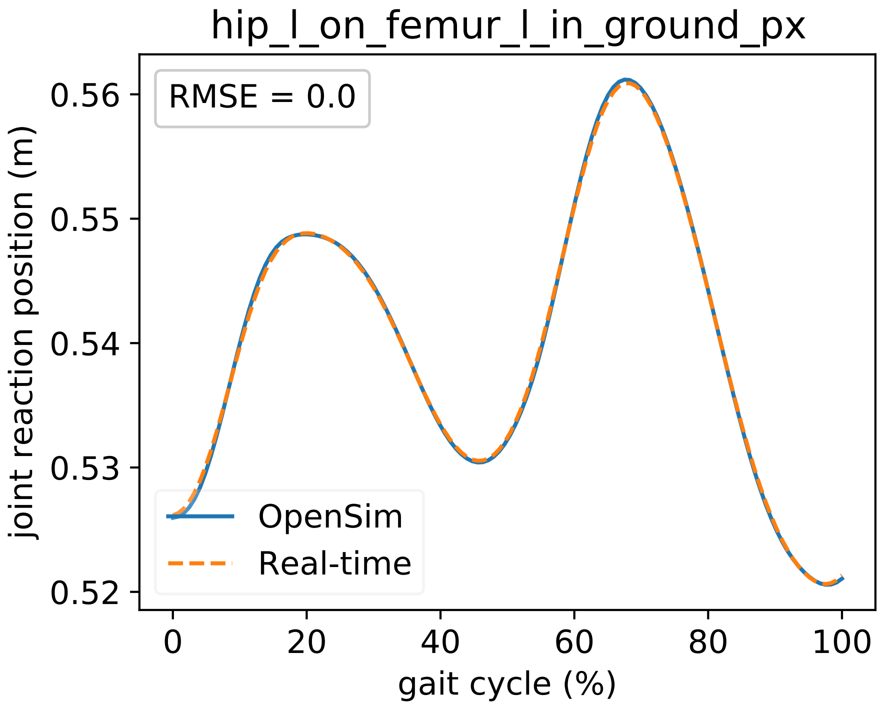

hip_l_on_femur_l_in_ground_px
RMSE = 0.0
OpenSim
Real-time
joint reaction position (m)
gait cycle (%)
0.52
0.53
0.54
0.55
0.56
0
20
40
60
80
100

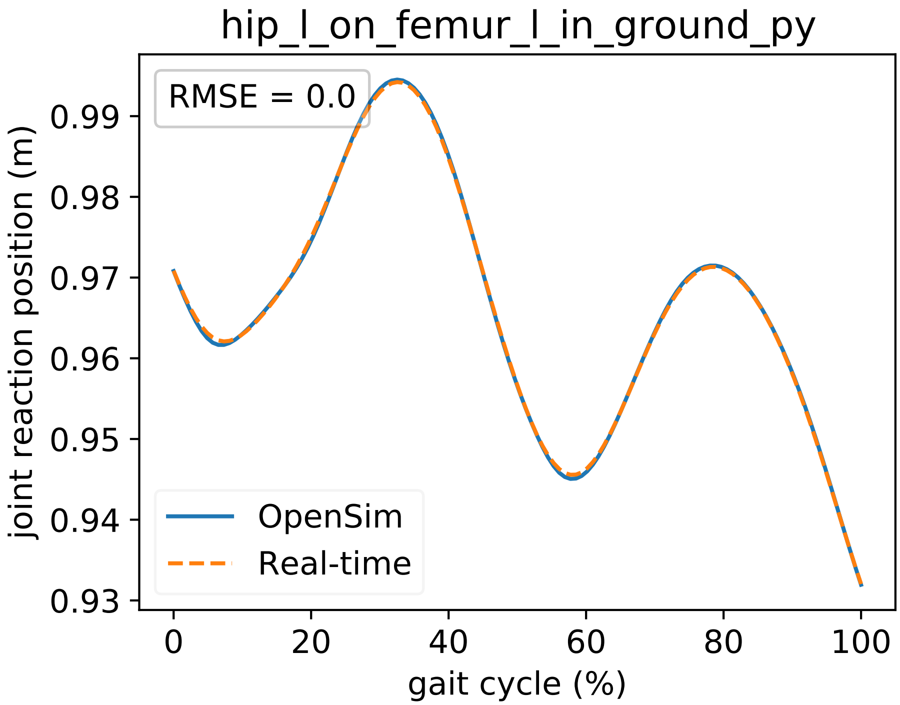
hip_l_on_femur_l_in_ground_py
RMSE = 0.0
OpenSim
Real-time
joint reaction position (m)
gait cycle (%)
0.93
0.94
0.95
0.96
0.97
0.98
0.99
0
20
40
60
80
100

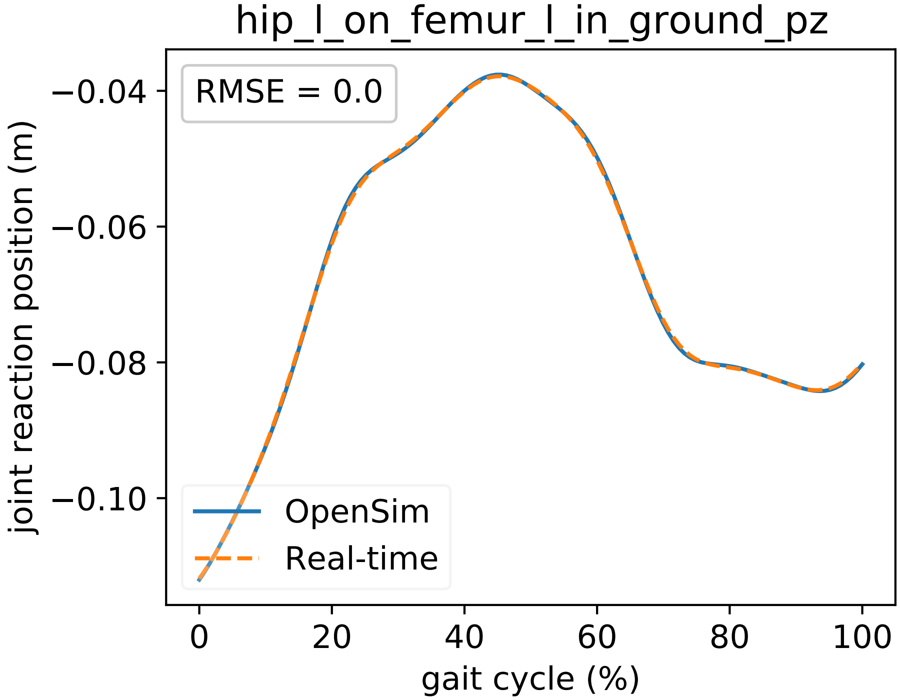

hip_l_on_femur_l_in_ground_pz
RMSE = 0.0
OpenSim
Real-time
joint reaction position (m)
gait cycle (%)
−0.04
−0.06
−0.08
−0.10
0
20
40
60
80
100

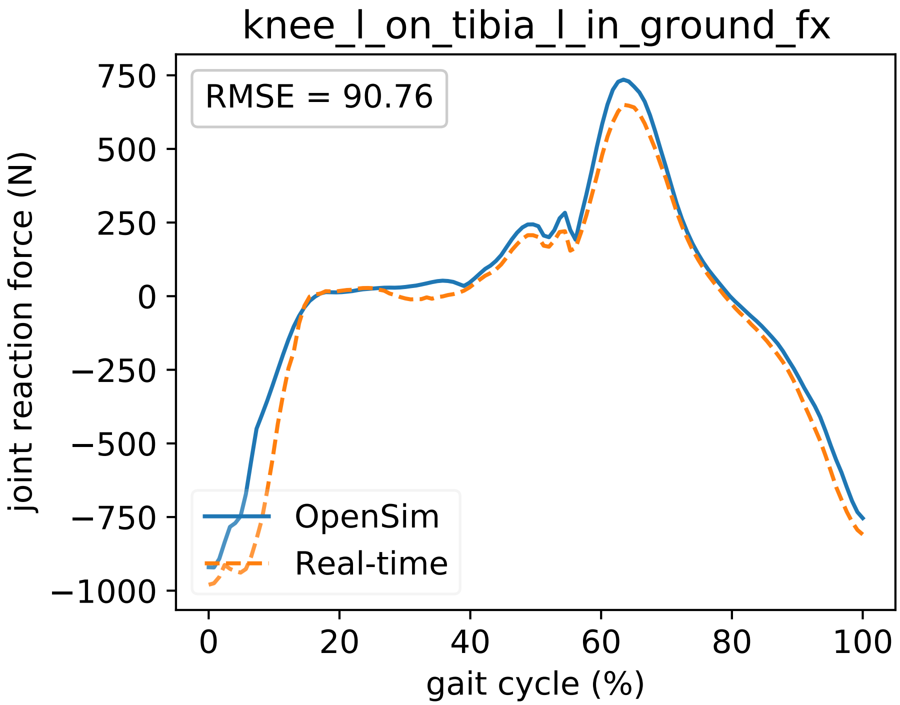

knee_l_on_tibia_l_in_ground_fx
RMSE = 90.76
OpenSim
Real-time
joint reaction force (N)
gait cycle (%)
750
500
250
0
−250
−500
−750
−1000
0
20
40
60
80
100

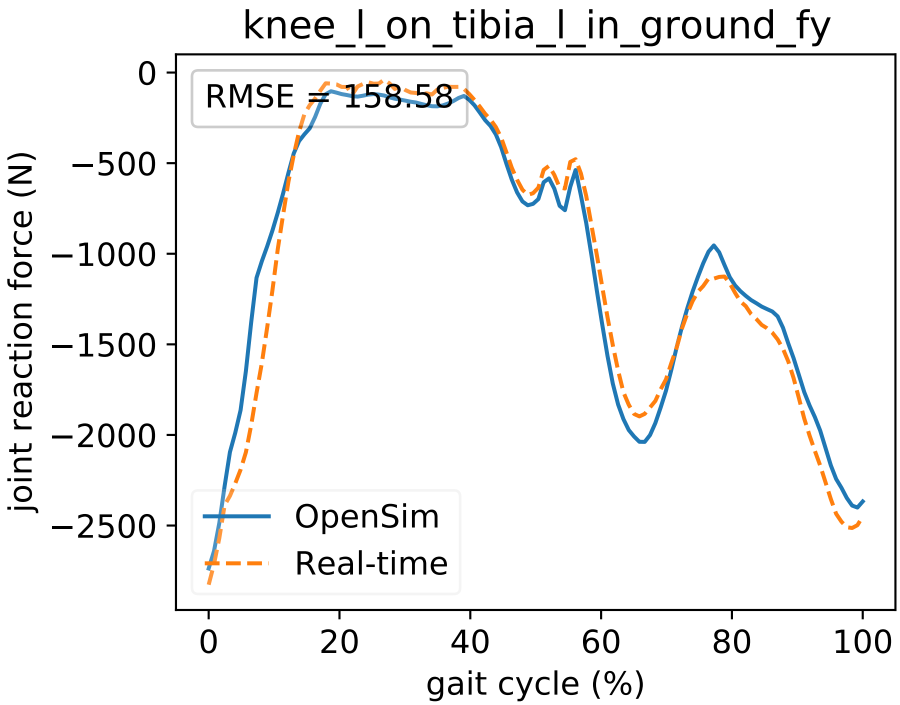

knee_l_on_tibia_l_in_ground_fy
RMSE = 158.58
OpenSim
Real-time
joint reaction force (N)
0
−500
−1000
−1500
−2000
−2500
0
20
40
60
80
100
gait cycle (%)

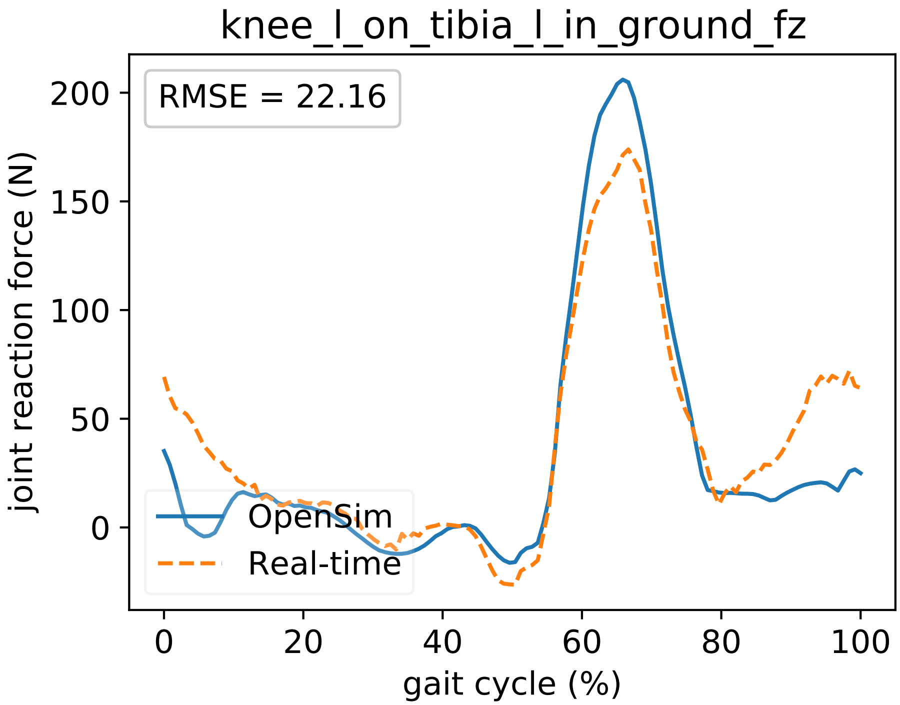
knee_l_on_tibia_l_in_ground_fz
RMSE = 22.16
OpenSim
Real-time
joint reaction force (N)
gait cycle (%)
0
50
100
150
200
0
20
40
60
80
100

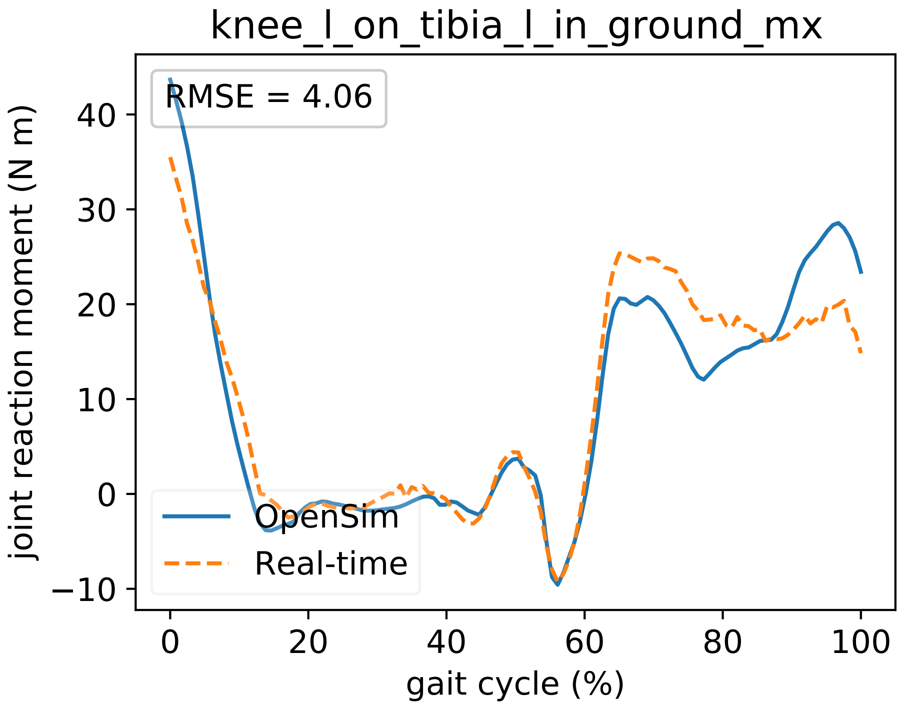
knee_l_on_tibia_l_in_ground_mx
RMSE = 4.06
OpenSim
Real-time
joint reaction moment (N m)
gait cycle (%)
40
30
20
10
0
−10
0
20
40
60
80
100

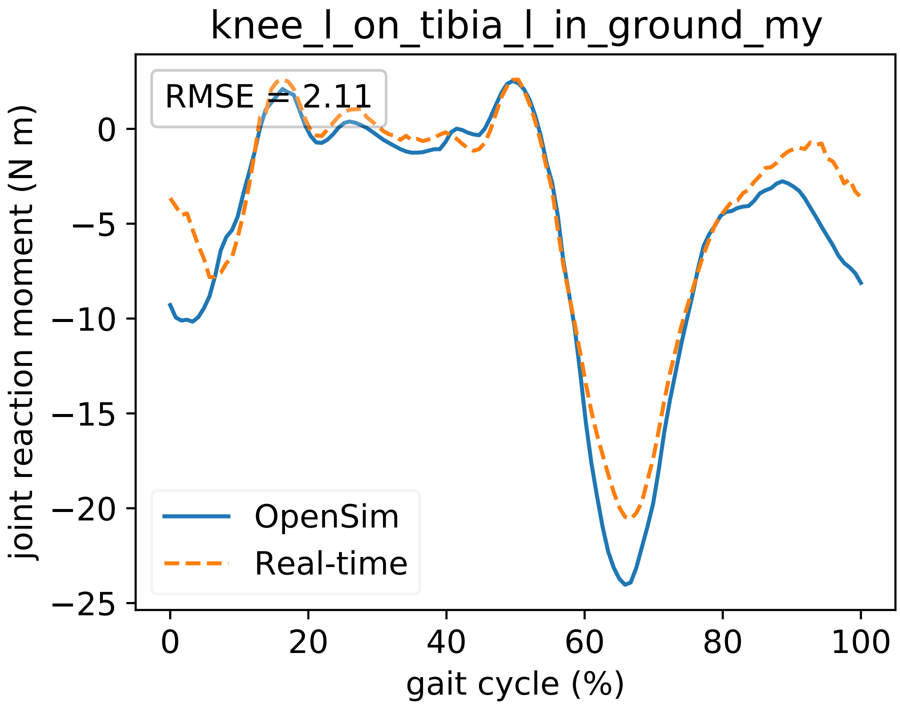

knee_l_on_tibia_l_in_ground_my
RMSE = 2.11
OpenSim
Real-time
joint reaction moment (N m)
gait cycle (%)
0
−5
−10
−15
−20
−25
0
20
40
60
80
100

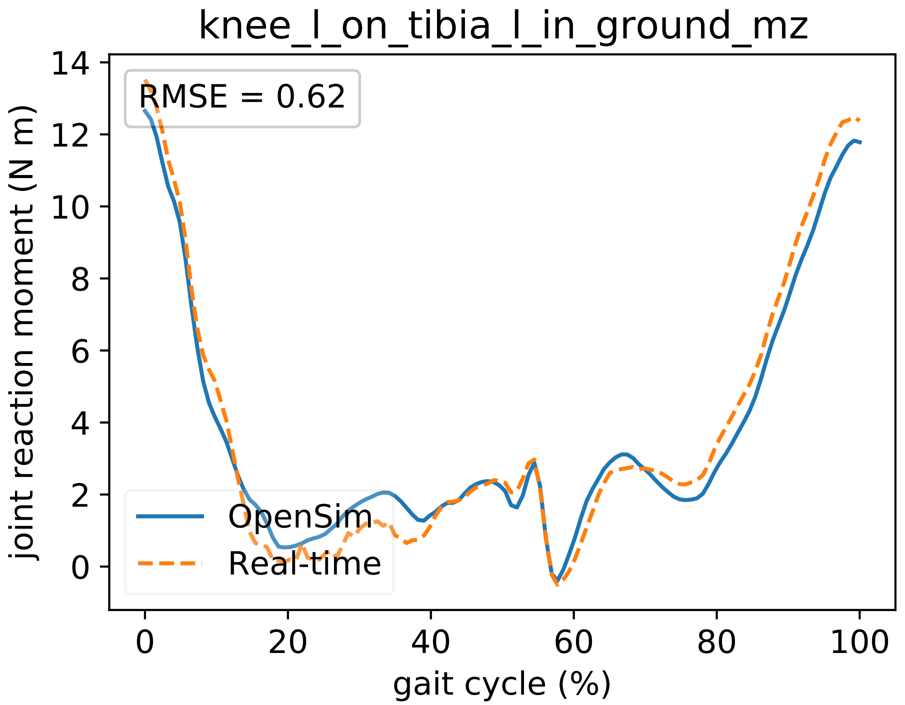
knee_l_on_tibia_l_in_ground_mz
RMSE = 0.62
OpenSim
Real-time
joint reaction moment (N m)
gait cycle (%)
0
2
4
6
8
10
12
14
0
20
40
60
80
100

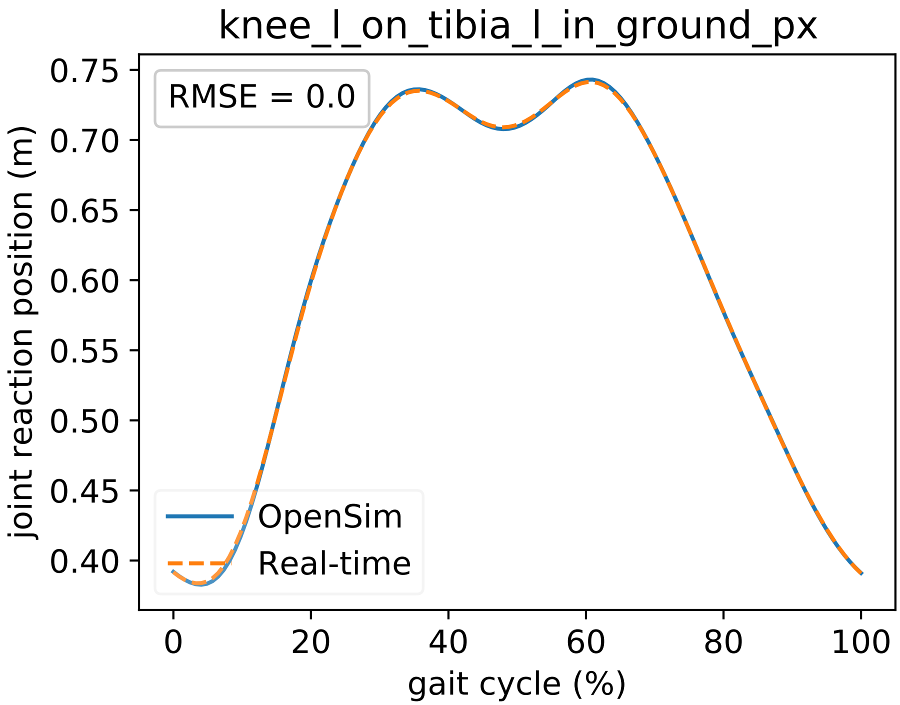
knee_l_on_tibia_l_in_ground_px
RMSE = 0.0
OpenSim
Real-time
joint reaction position (m)
0.75
0.70
0.65
0.60
0.55
0.50
0.45
0.40
0
20
40
60
80
100
gait cycle (%)

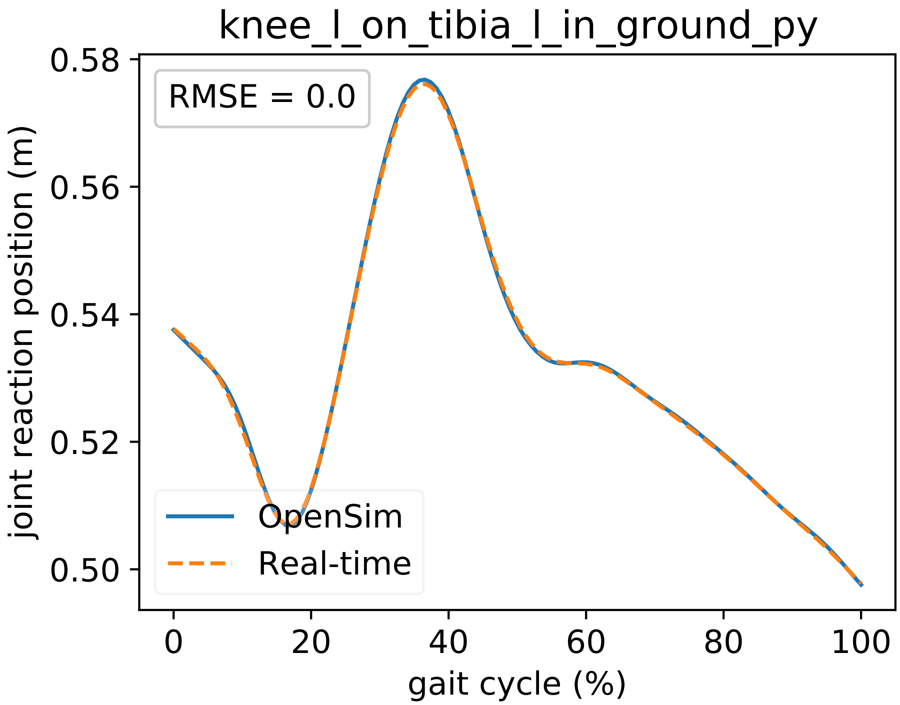
knee_l_on_tibia_l_in_ground_py
RMSE = 0.0
OpenSim
Real-time
joint reaction position (m)
gait cycle (%)
0.58
0.56
0.54
0.52
0.50
0
20
40
60
80
100

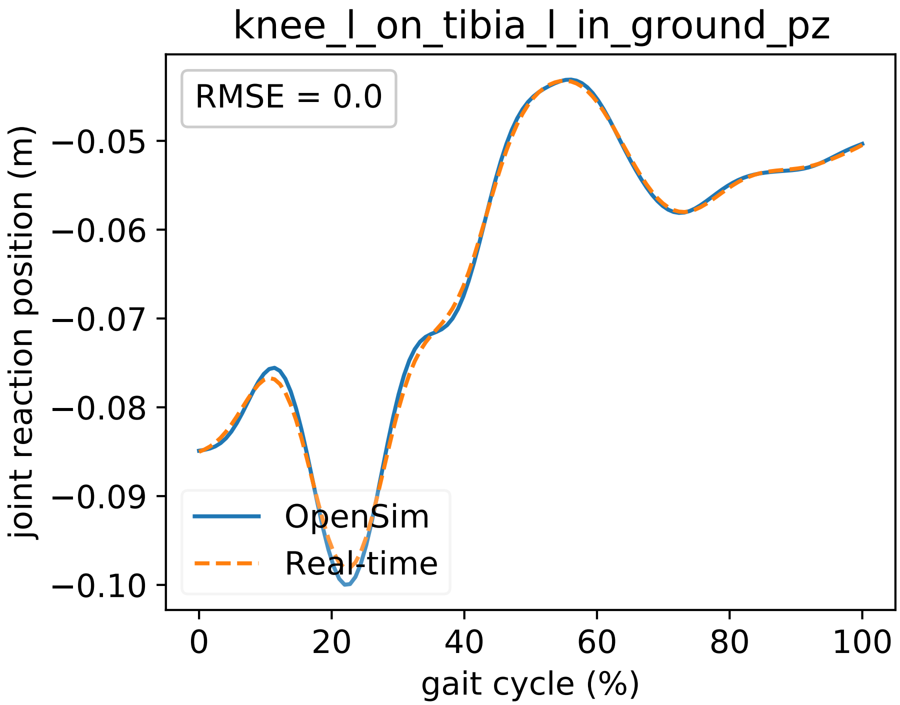
knee_l_on_tibia_l_in_ground_pz
RMSE = 0.0
OpenSim
Real-time
joint reaction position (m)
−0.05
−0.06
−0.07
−0.08
−0.09
−0.10
0
20
40
60
80
100
gait cycle (%)

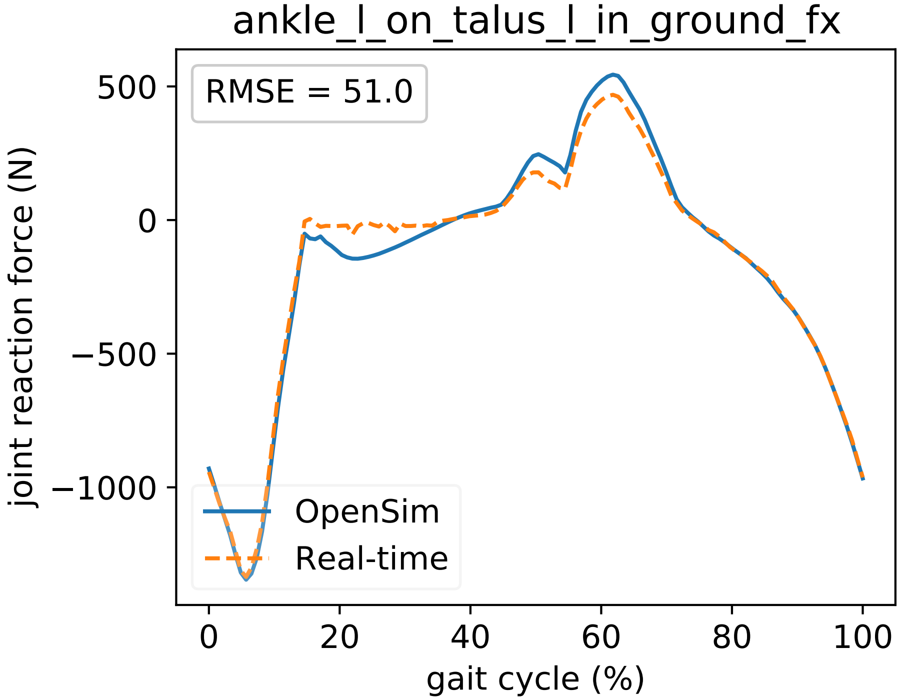

ankle_l_on_talus_l_in_ground_fx
RMSE = 51.0
500
0
−500
−1000
joint reaction force (N)
OpenSim
Real-time
0
20
40
60
80
100
gait cycle (%)

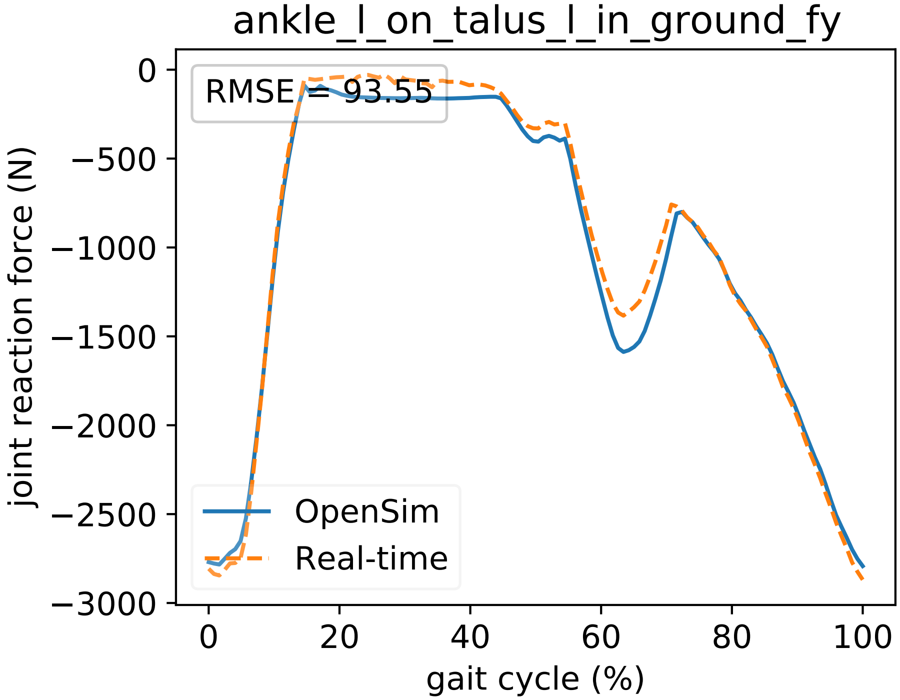

ankle_l_on_talus_l_in_ground_fy
RMSE = 93.55
joint reaction force (N)
0
−500
−1000
−1500
−2000
−2500
−3000
OpenSim
Real-time
0
20
40
60
80
100
gait cycle (%)

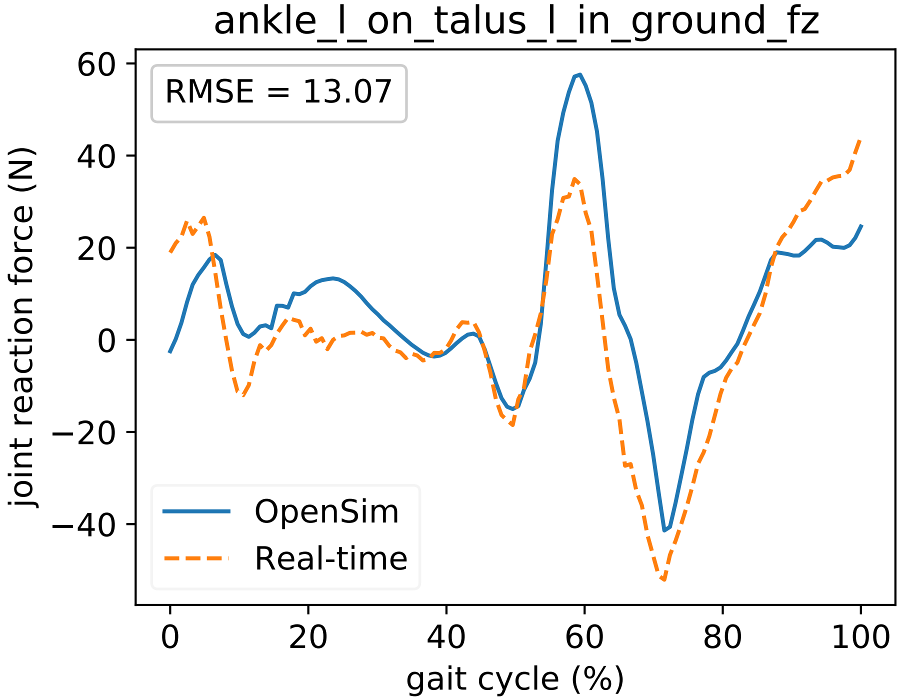

ankle_l_on_talus_l_in_ground_fz
RMSE = 13.07
OpenSim
Real-time
joint reaction force (N)
gait cycle (%)
60
40
20
0
−20
−40
0
20
40
60
80
100

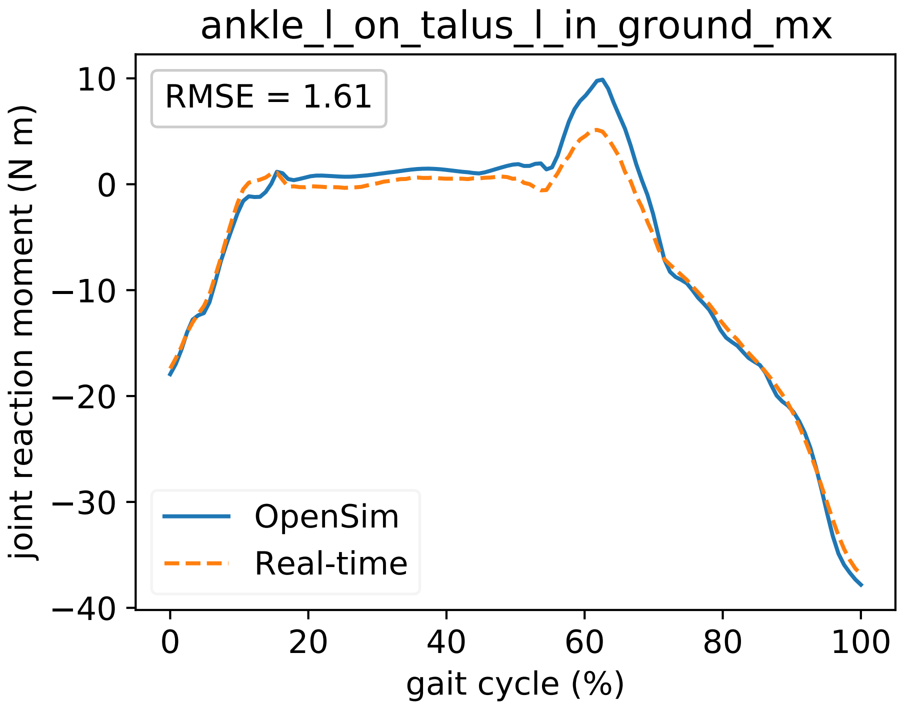
ankle_l_on_talus_l_in_ground_mx
RMSE = 1.61
OpenSim
Real-time
joint reaction moment (N m)
gait cycle (%)
10
0
−10
−20
−30
−40
0
20
40
60
80
100

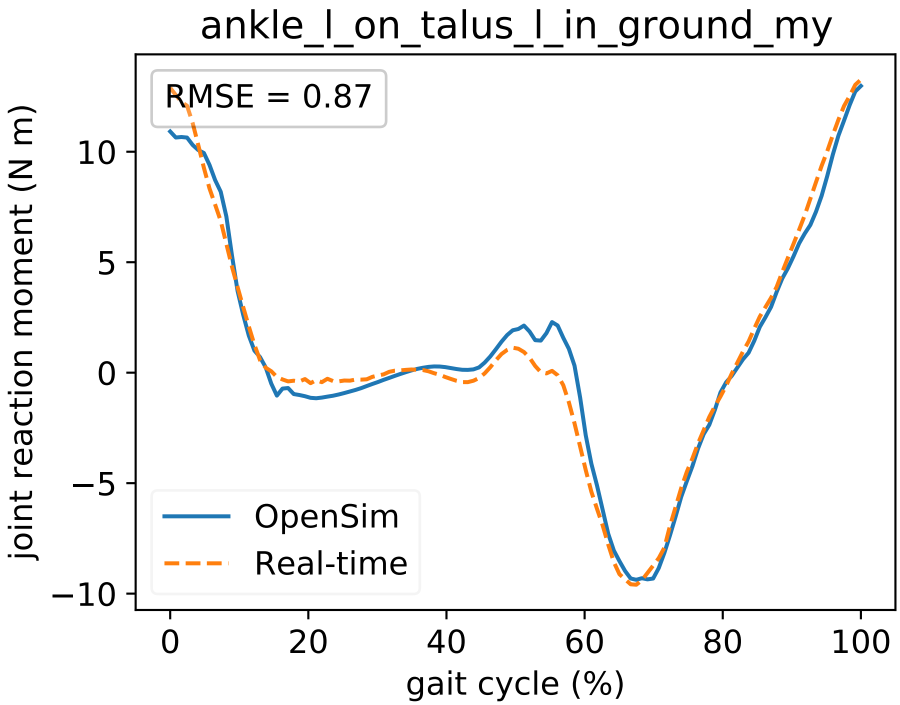
ankle_l_on_talus_l_in_ground_my
RMSE = 0.87
OpenSim
Real-time
joint reaction moment (N m)
gait cycle (%)
10
5
0
−5
−10
0
20
40
60
80
100

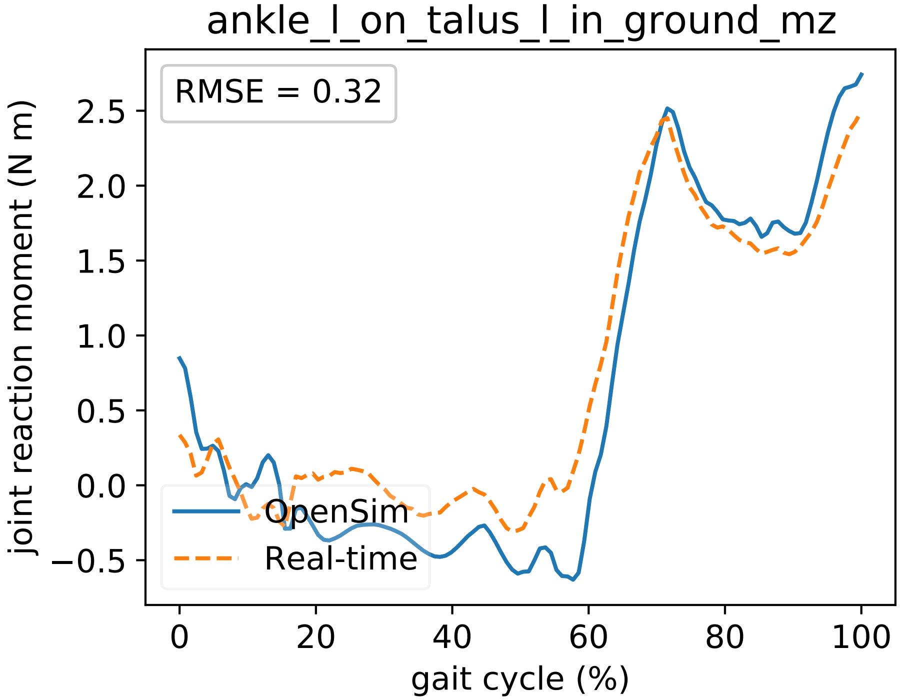

ankle_l_on_talus_l_in_ground_mz
RMSE = 0.32
joint reaction moment (N m)
2.5
2.0
1.5
1.0
0.5
0.0
−0.5
OpenSim
Real-time
0
20
40
60
80
100
gait cycle (%)

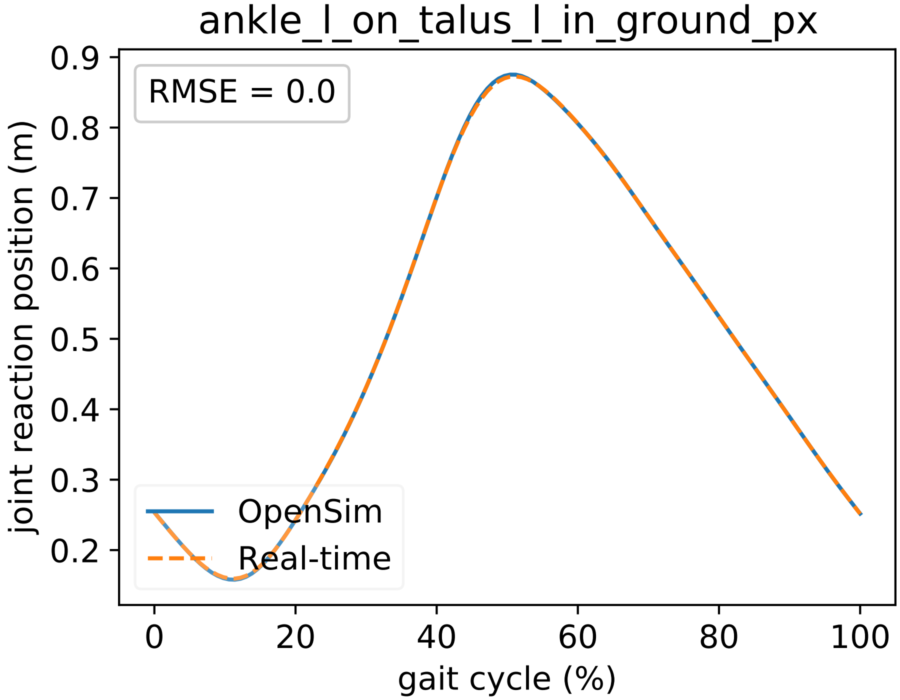
ankle_l_on_talus_l_in_ground_px
RMSE = 0.0
OpenSim
Real-time
joint reaction position (m)
gait cycle (%)
0.9
0.8
0.7
0.6
0.5
0.4
0.3
0.2
0
20
40
60
80
100

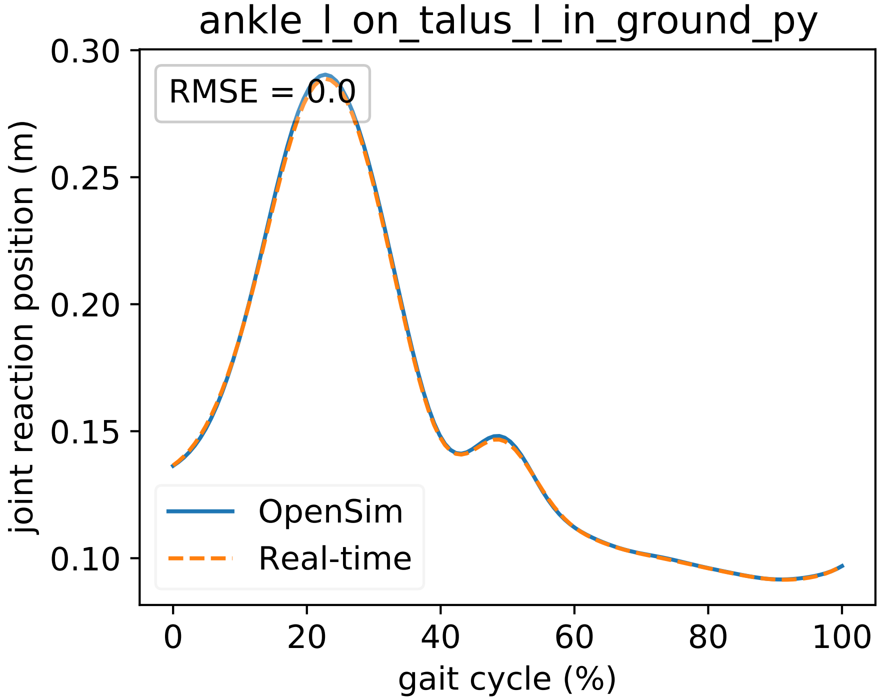

ankle_l_on_talus_l_in_ground_py
RMSE = 0.0
OpenSim
Real-time
joint reaction position (m)
gait cycle (%)
0.30
0.25
0.20
0.15
0.10
0
20
40
60
80
100

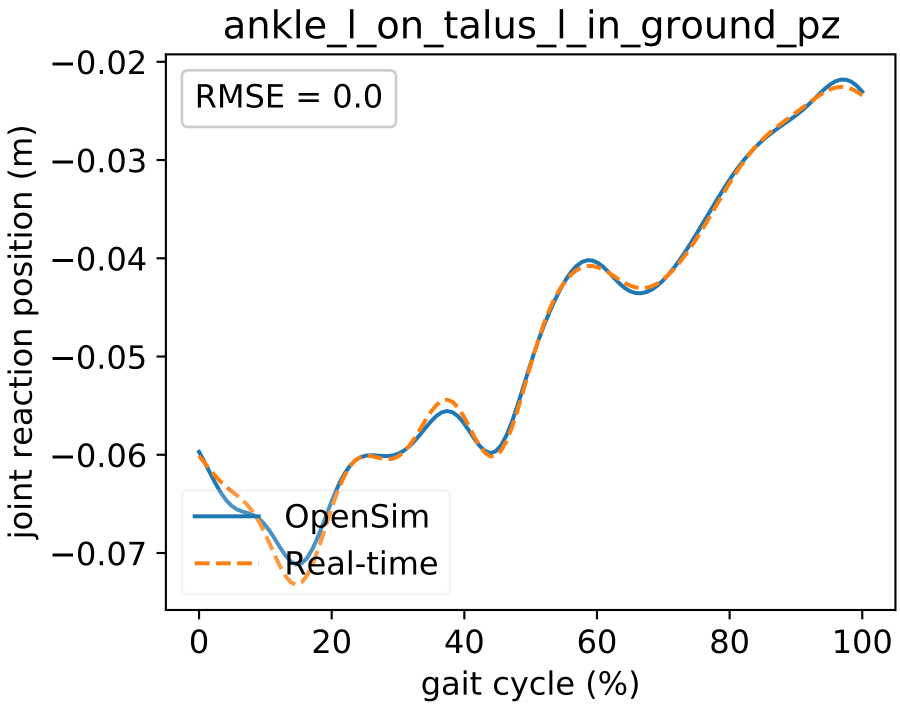
ankle_l_on_talus_l_in_ground_pz
RMSE = 0.0
OpenSim
Real-time
joint reaction position (m)
gait cycle (%)
−0.02
−0.03
−0.04
−0.05
−0.06
−0.07
0
20
40
60
80
100

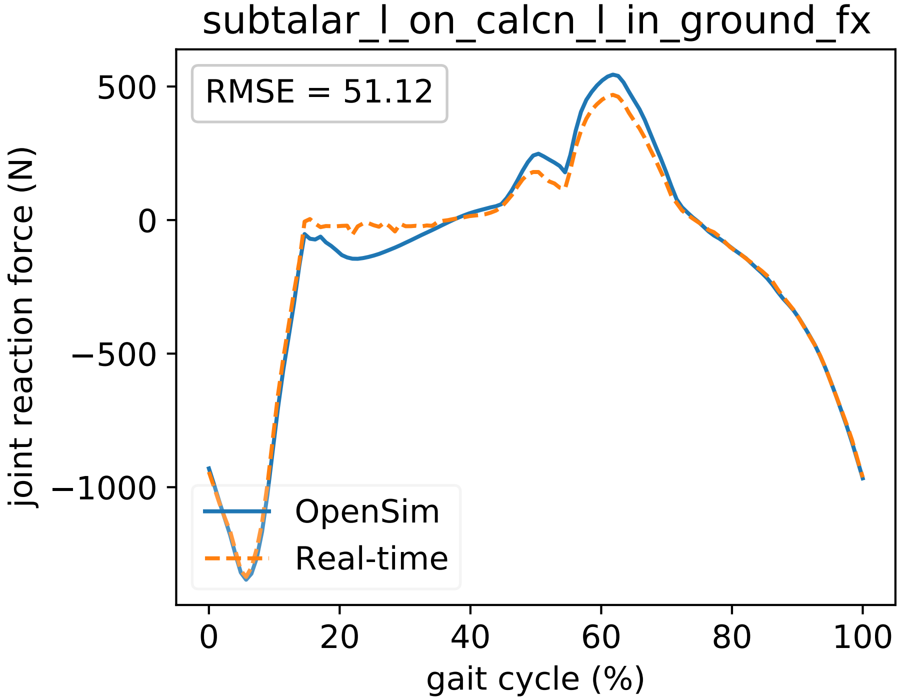

subtalar_l_on_calcn_l_in_ground_fx
RMSE = 51.12
OpenSim
Real-time
joint reaction force (N)
500
0
−500
−1000
0
20
40
60
80
100
gait cycle (%)

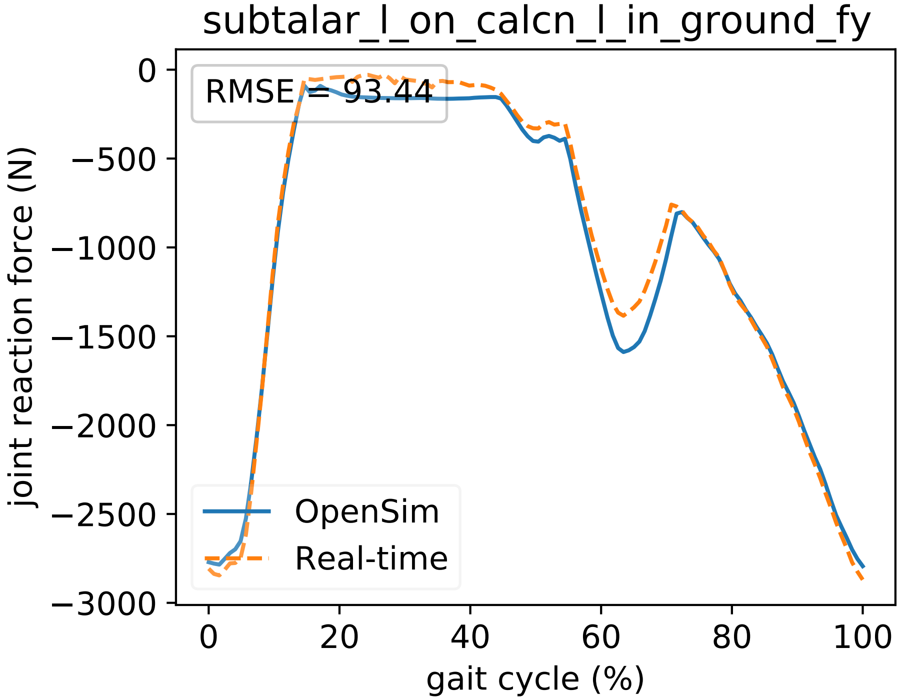

subtalar_l_on_calcn_l_in_ground_fy
RMSE = 93.44
OpenSim
Real-time
joint reaction force (N)
gait cycle (%)
0
−500
−1000
−1500
−2000
−2500
−3000
0
20
40
60
80
100

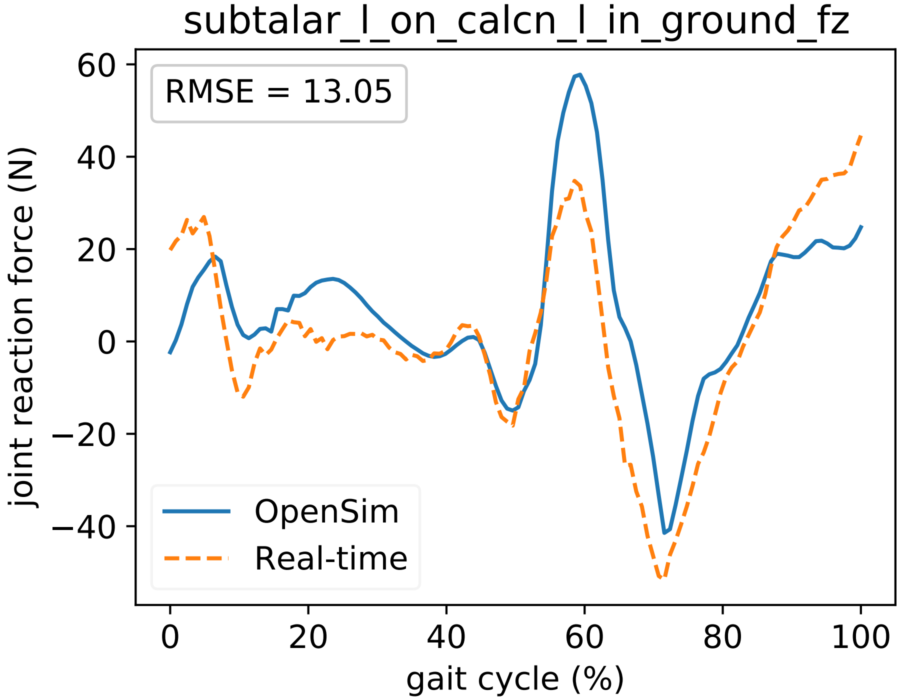

subtalar_l_on_calcn_l_in_ground_fz
RMSE = 13.05
OpenSim
Real-time
joint reaction force (N)
gait cycle (%)
60
40
20
0
−20
−40
0
20
40
60
80
100

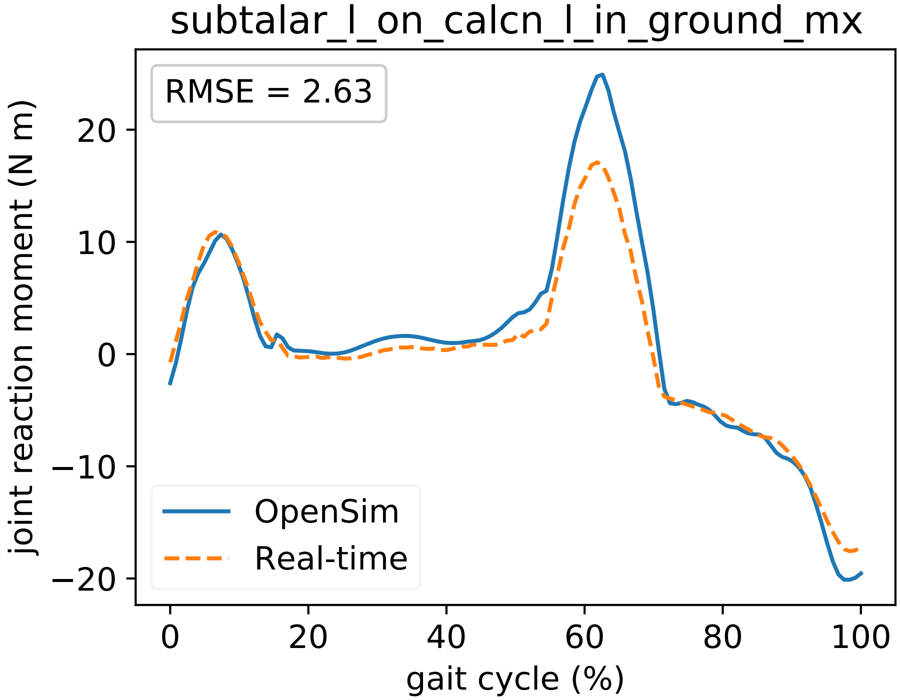
subtalar_l_on_calcn_l_in_ground_mx
RMSE = 2.63
OpenSim
Real-time
joint reaction moment (N m)
gait cycle (%)
20
10
0
−10
−20
0
20
40
60
80
100

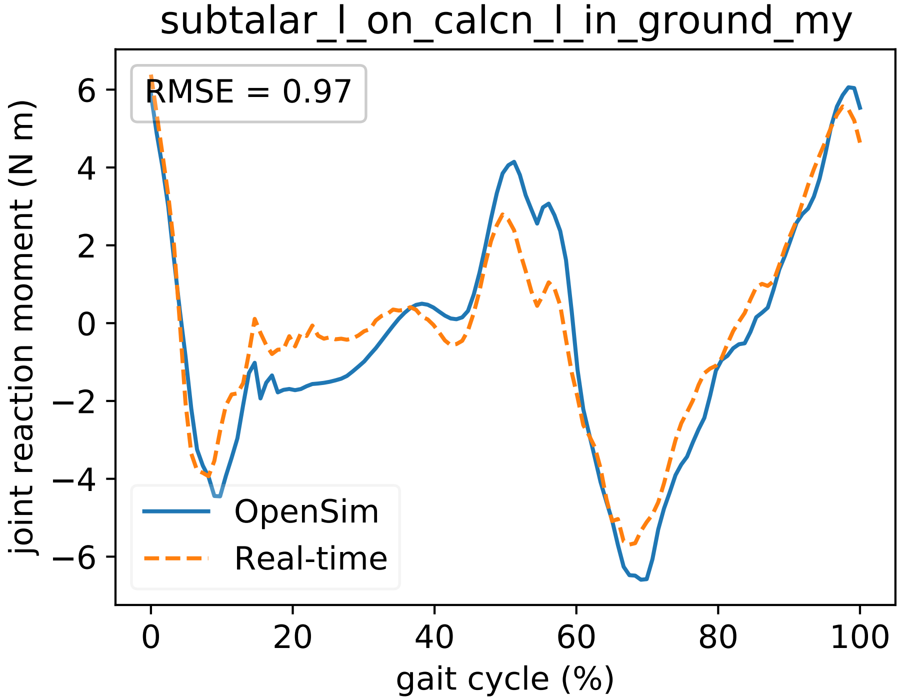
subtalar_l_on_calcn_l_in_ground_my
RMSE = 0.97
joint reaction moment (N m)
gait cycle (%)
OpenSim
Real-time
6
4
2
0
−2
−4
−6
0
20
40
60
80
100

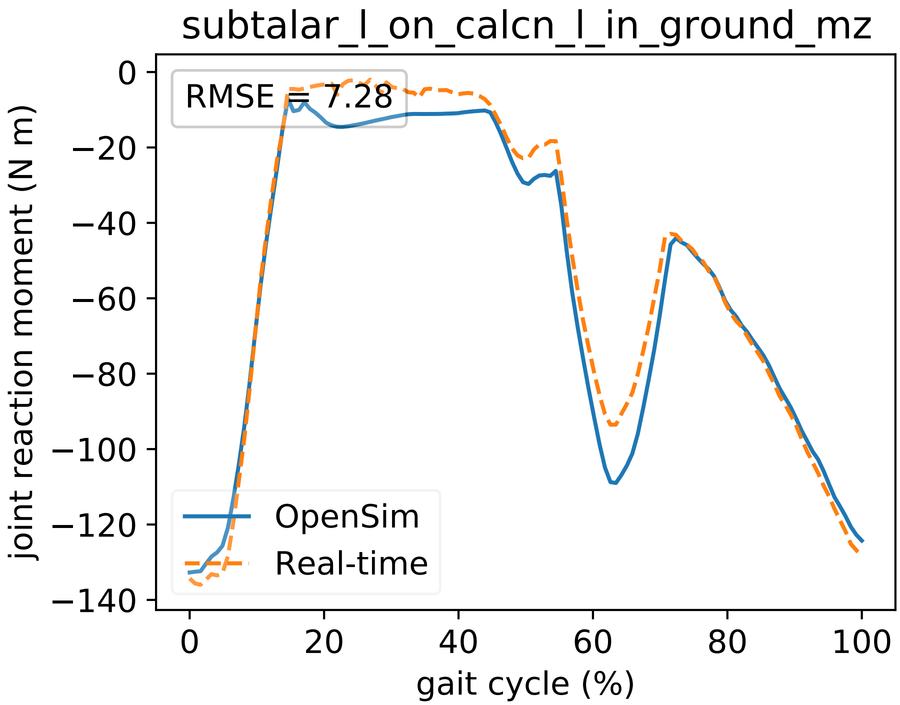

subtalar_l_on_calcn_l_in_ground_mz
RMSE = 7.28
joint reaction moment (N m)
0
−20
−40
−60
−80
−100
−120
−140
0
20
40
60
80
100
gait cycle (%)
OpenSim
Real-time

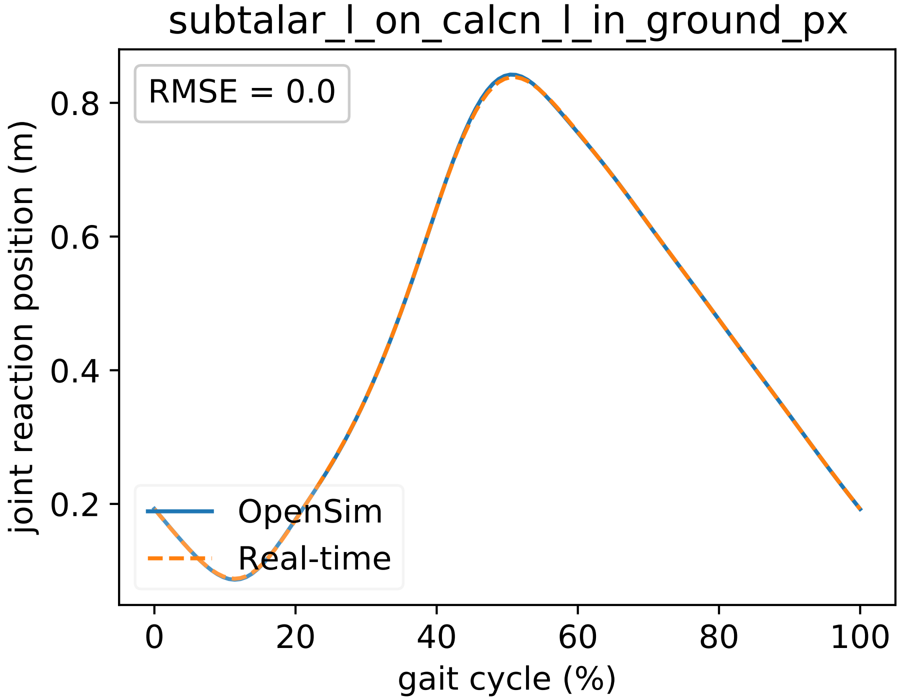
subtalar_l_on_calcn_l_in_ground_px
RMSE = 0.0
OpenSim
Real-time
joint reaction position (m)
gait cycle (%)
0.2
0.4
0.6
0.8
0
20
40
60
80
100

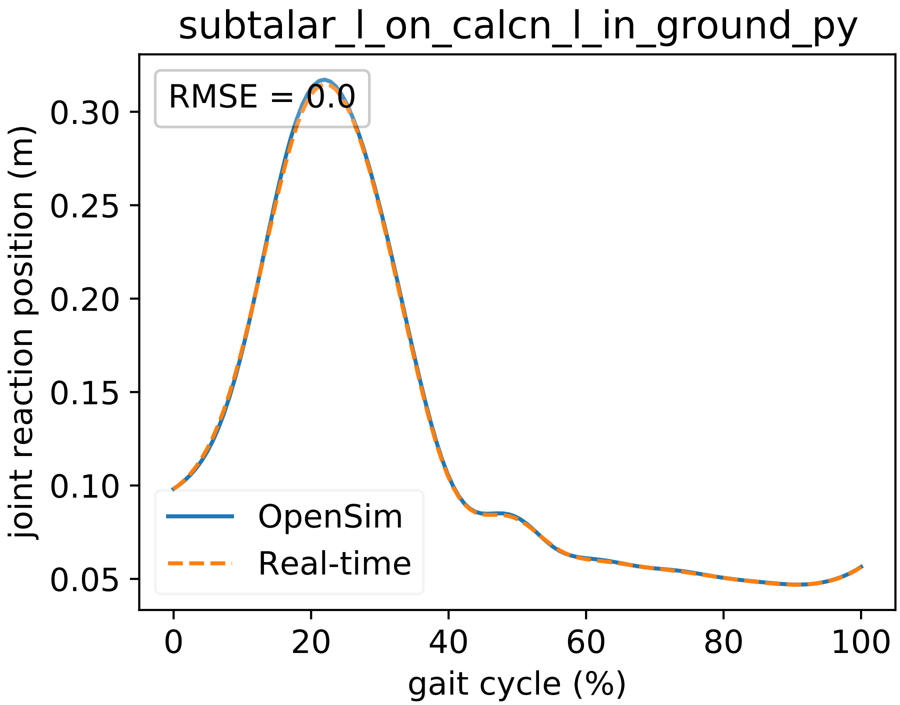
subtalar_l_on_calcn_l_in_ground_py
RMSE = 0.0
OpenSim
Real-time
joint reaction position (m)
gait cycle (%)
0.30
0.25
0.20
0.15
0.10
0.05
0
20
40
60
80
100

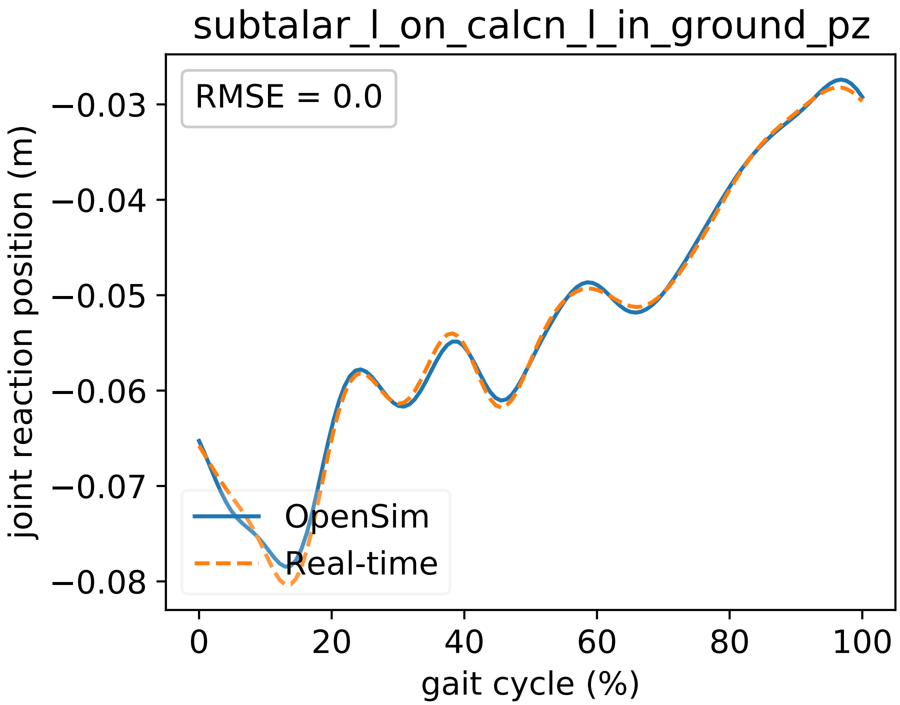

subtalar_l_on_calcn_l_in_ground_pz
RMSE = 0.0
joint reaction position (m)
−0.03
−0.04
−0.05
−0.06
−0.07
−0.08
OpenSim
Real-time
0
20
40
60
80
100
gait cycle (%)

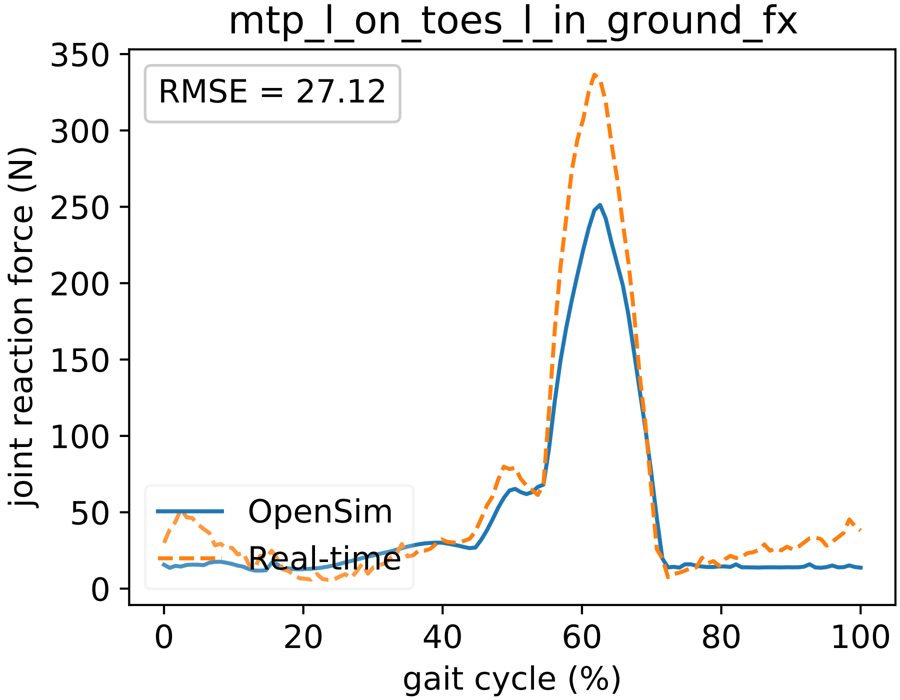
mtp_l_on_toes_l_in_ground_fx
RMSE = 27.12
OpenSim
Real-time
joint reaction force (N)
gait cycle (%)
0
50
100
150
200
250
300
350
0
20
40
60
80
100

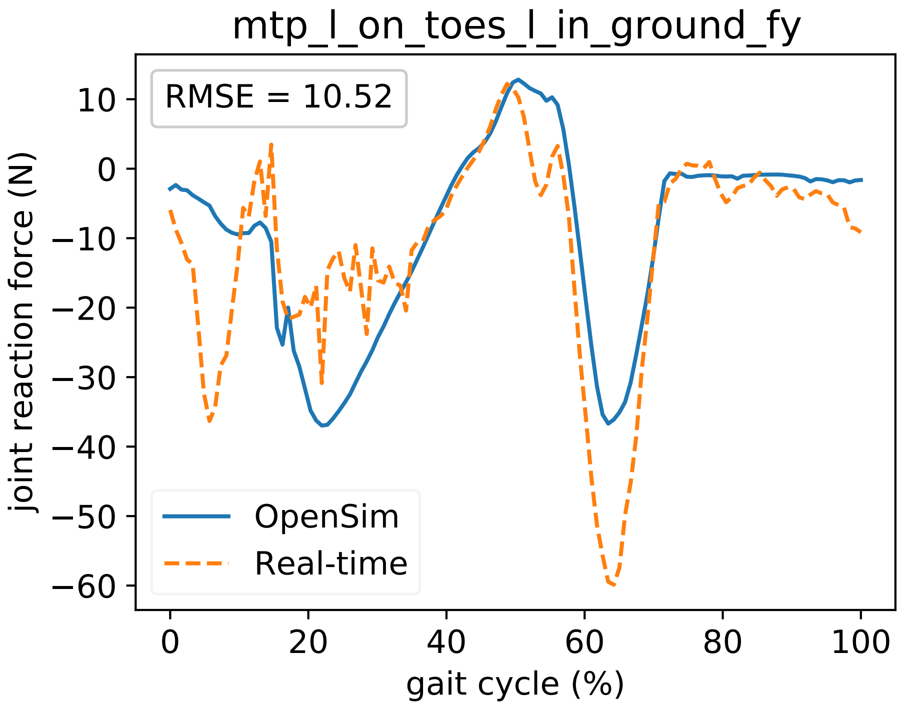

mtp_l_on_toes_l_in_ground_fy
RMSE = 10.52
OpenSim
Real-time
joint reaction force (N)
gait cycle (%)
10
0
−10
−20
−30
−40
−50
−60
0
20
40
60
80
100

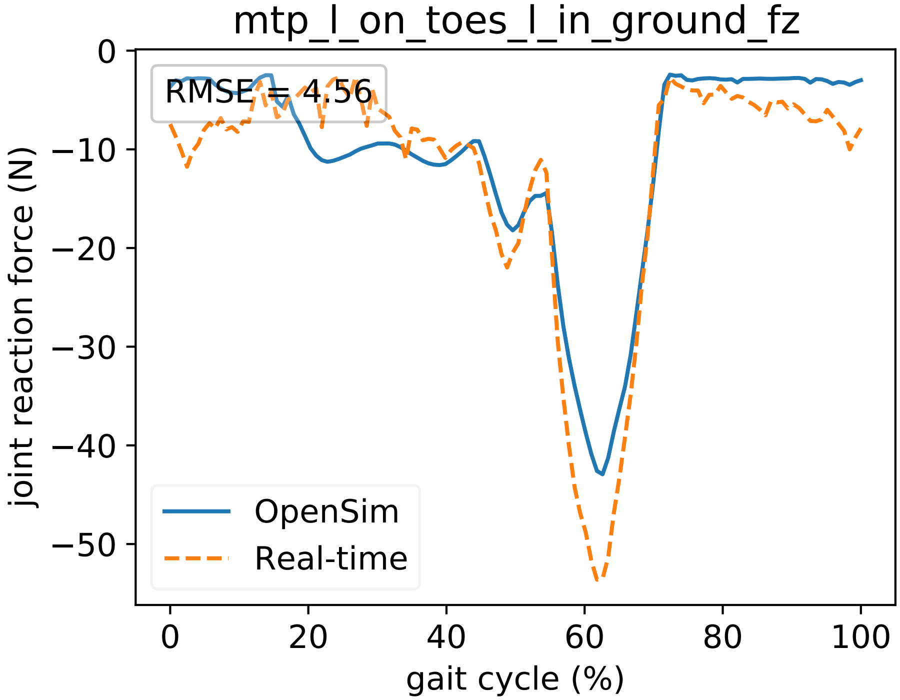

mtp_l_on_toes_l_in_ground_fz
RMSE = 4.56
OpenSim
Real-time
joint reaction force (N)
gait cycle (%)
0
−10
−20
−30
−40
−50
0
20
40
60
80
100

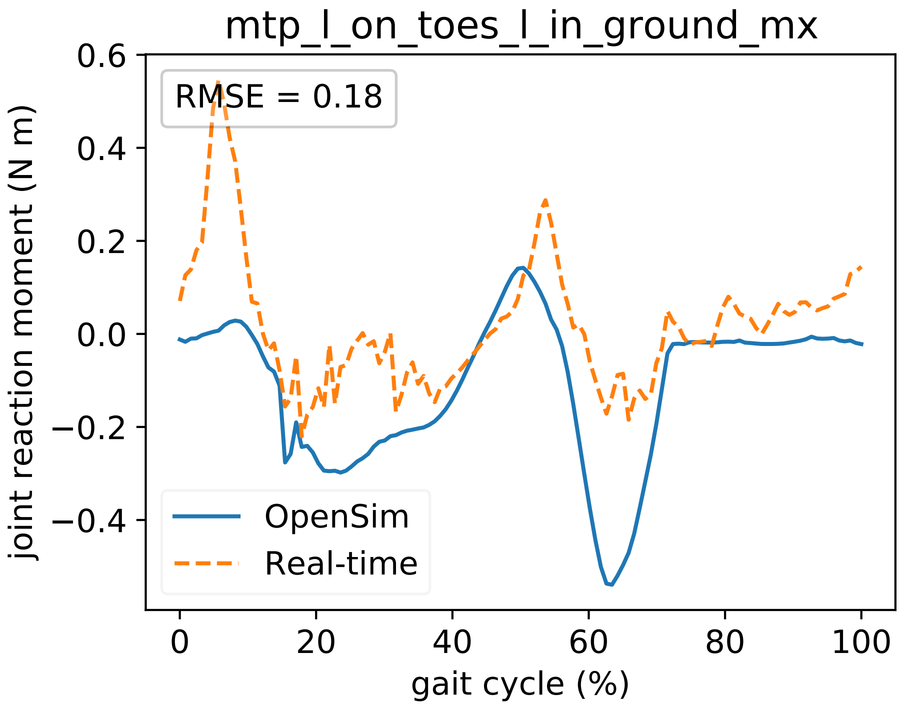

mtp_l_on_toes_l_in_ground_mx
RMSE = 0.18
0.6
0.4
0.2
0.0
−0.2
−0.4
joint reaction moment (N m)
OpenSim
Real-time
0
20
40
60
80
100
gait cycle (%)

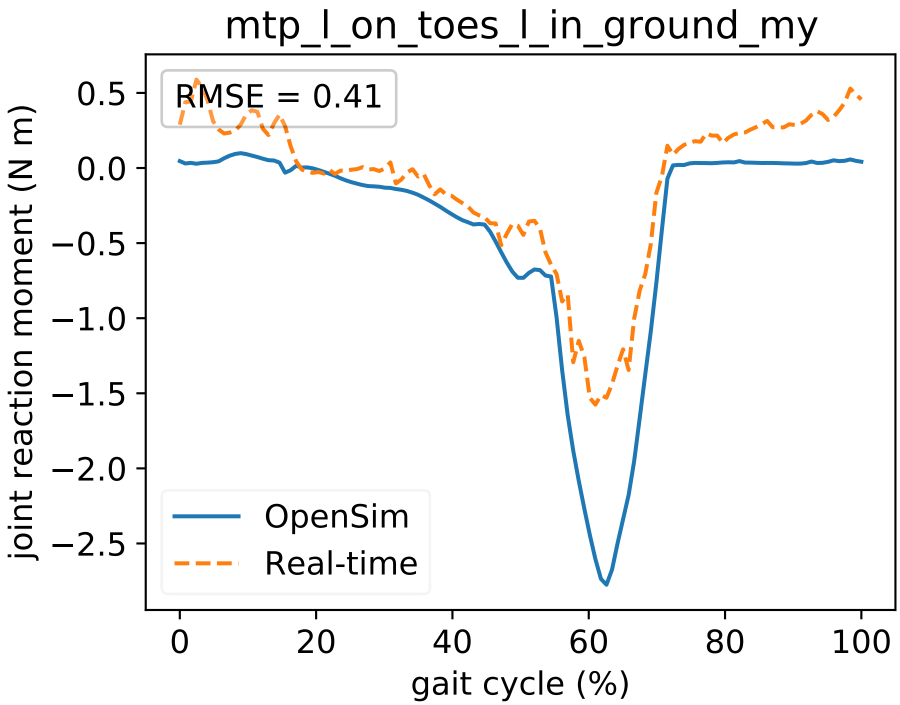

mtp_l_on_toes_l_in_ground_my
RMSE = 0.41
joint reaction moment (N m)
0.5
0.0
−0.5
−1.0
−1.5
−2.0
−2.5
0
20
40
60
80
100
gait cycle (%)
OpenSim
Real-time

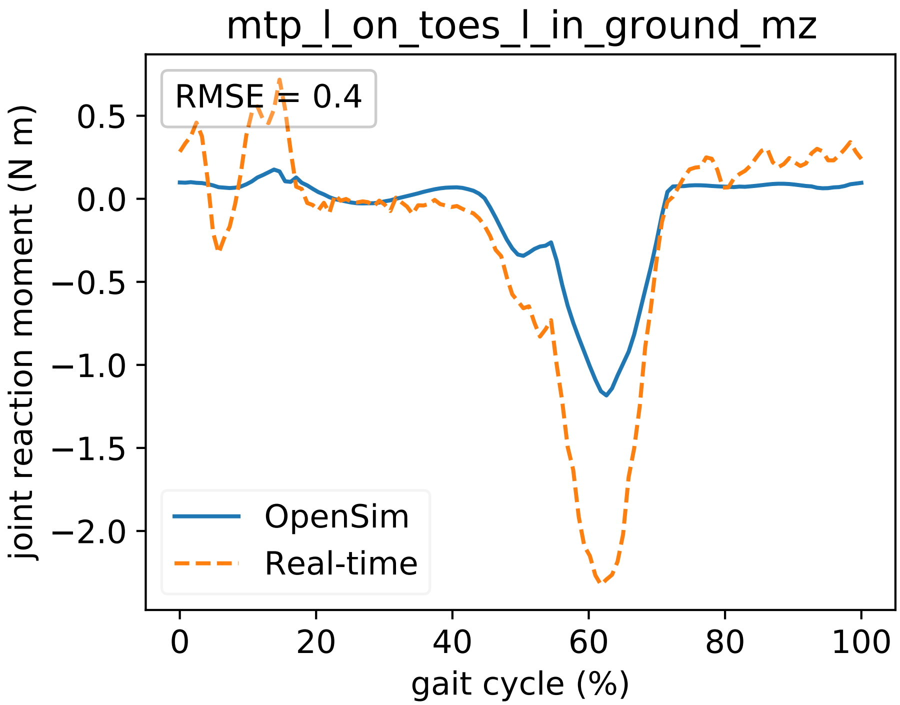

mtp_l_on_toes_l_in_ground_mz
RMSE = 0.4
OpenSim
Real-time
joint reaction moment (N m)
gait cycle (%)
0.5
0.0
−0.5
−1.0
−1.5
−2.0
0
20
40
60
80
100

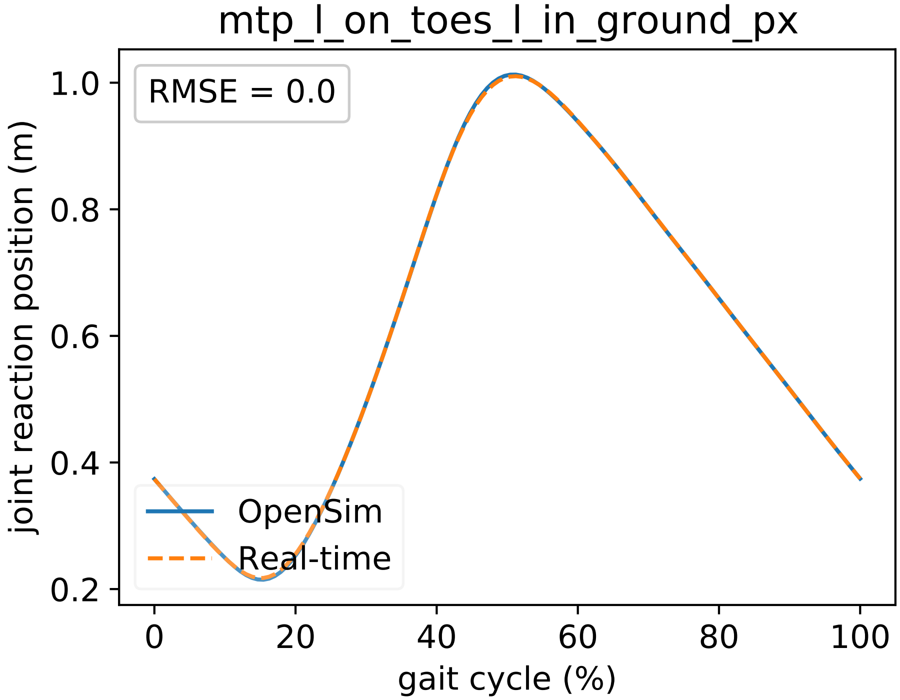
mtp_l_on_toes_l_in_ground_px
RMSE = 0.0
OpenSim
Real-time
joint reaction position (m)
gait cycle (%)
1.0
0.8
0.6
0.4
0.2
0
20
40
60
80
100

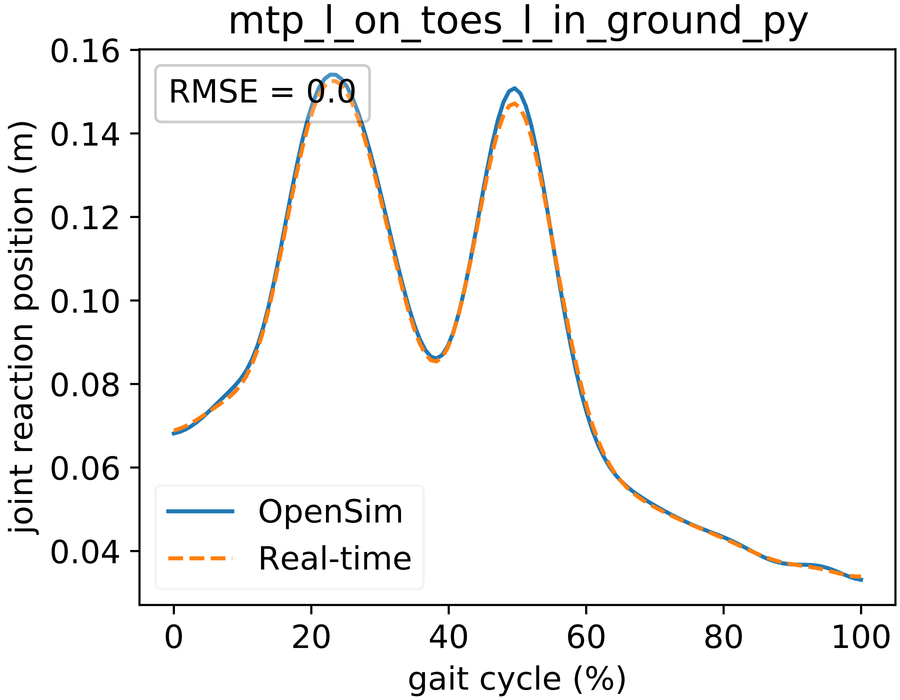
mtp_l_on_toes_l_in_ground_py
RMSE = 0.0
OpenSim
Real-time
joint reaction position (m)
gait cycle (%)
0.16
0.14
0.12
0.10
0.08
0.06
0.04
0
20
40
60
80
100

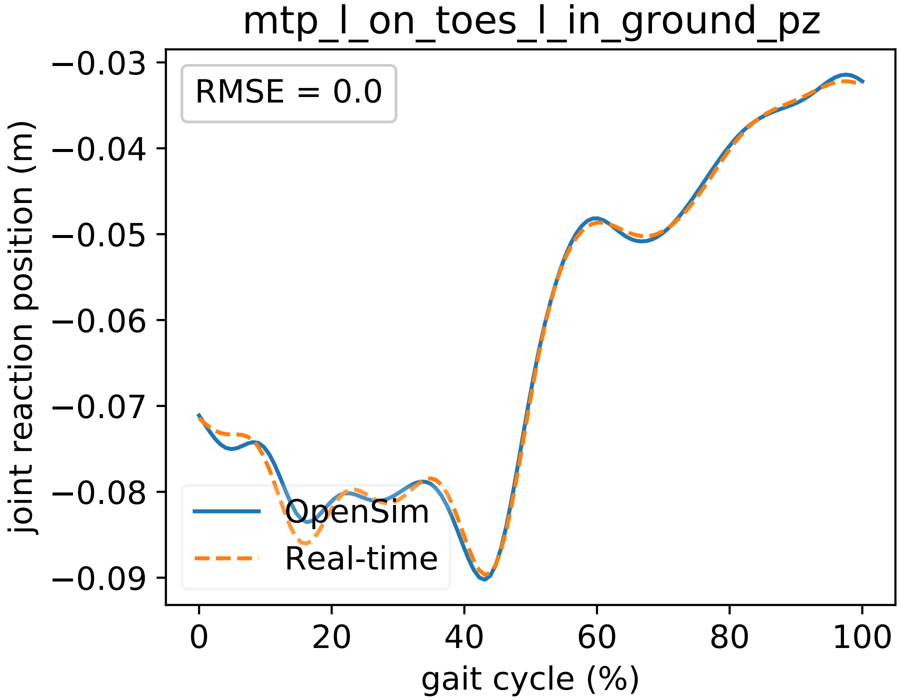
mtp_l_on_toes_l_in_ground_pz
RMSE = 0.0
−0.03
−0.04
−0.05
−0.06
−0.07
−0.08
−0.09
joint reaction position (m)
0
20
40
60
80
100
gait cycle (%)
OpenSim
Real-time

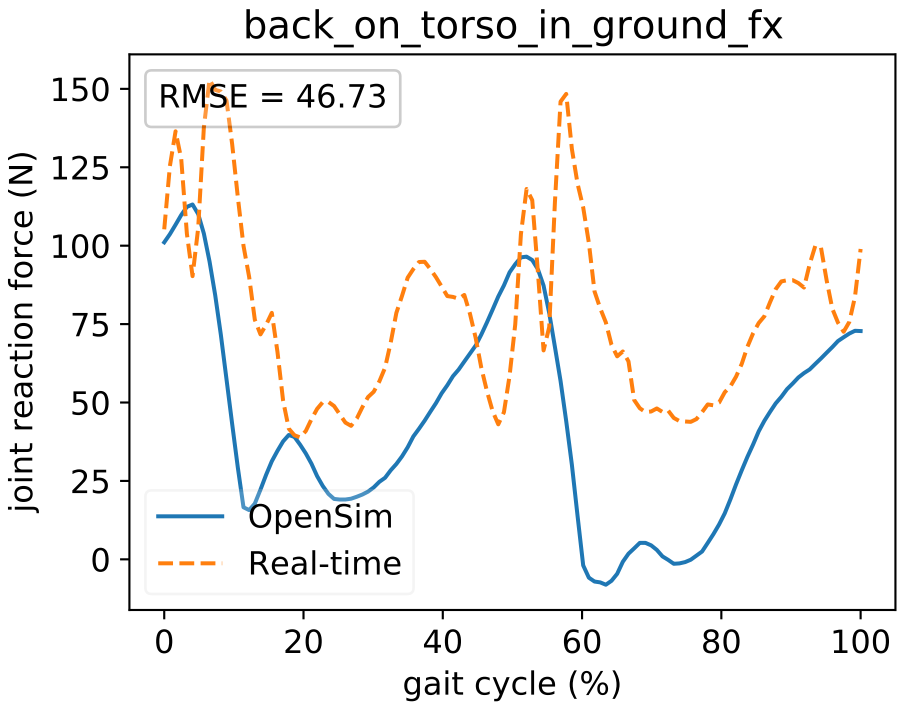
back_on_torso_in_ground_fx
RMSE = 46.73
joint reaction force (N)
150
125
100
75
50
25
0
OpenSim
Real-time
0
20
40
60
80
100
gait cycle (%)

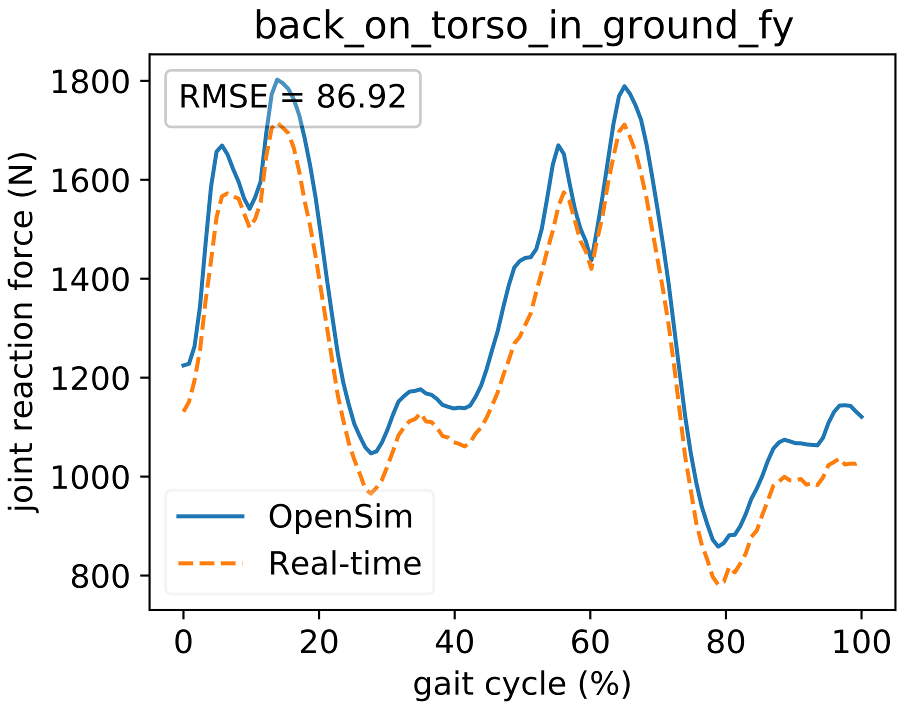
back_on_torso_in_ground_fy
RMSE = 86.92
OpenSim
Real-time
joint reaction force (N)
1800
1600
1400
1200
1000
800
0
20
40
60
80
100
gait cycle (%)

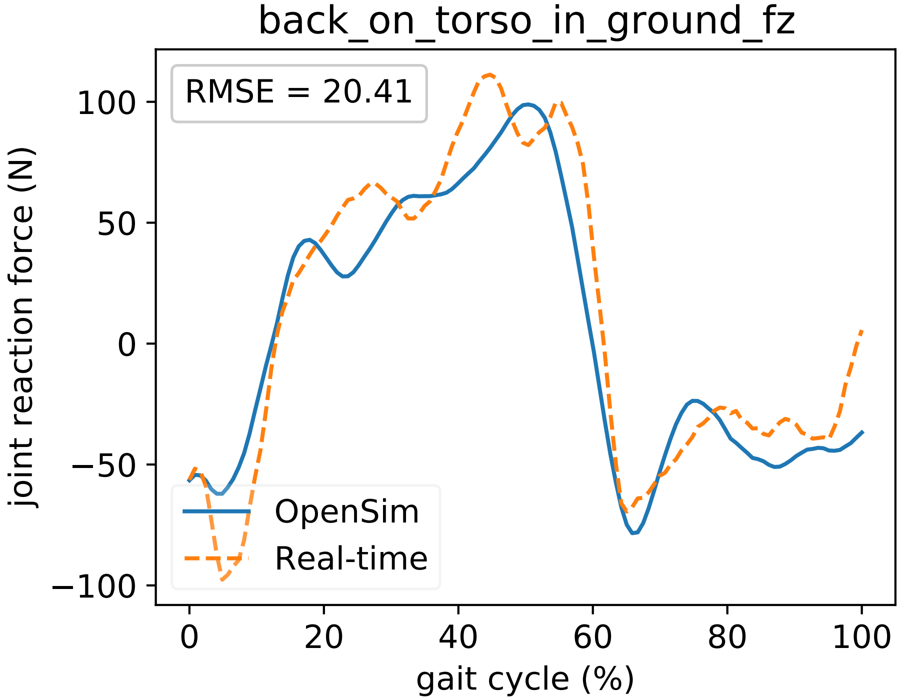

back_on_torso_in_ground_fz
RMSE = 20.41
OpenSim
Real-time
joint reaction force (N)
gait cycle (%)
100
50
0
−50
−100
0
20
40
60
80
100

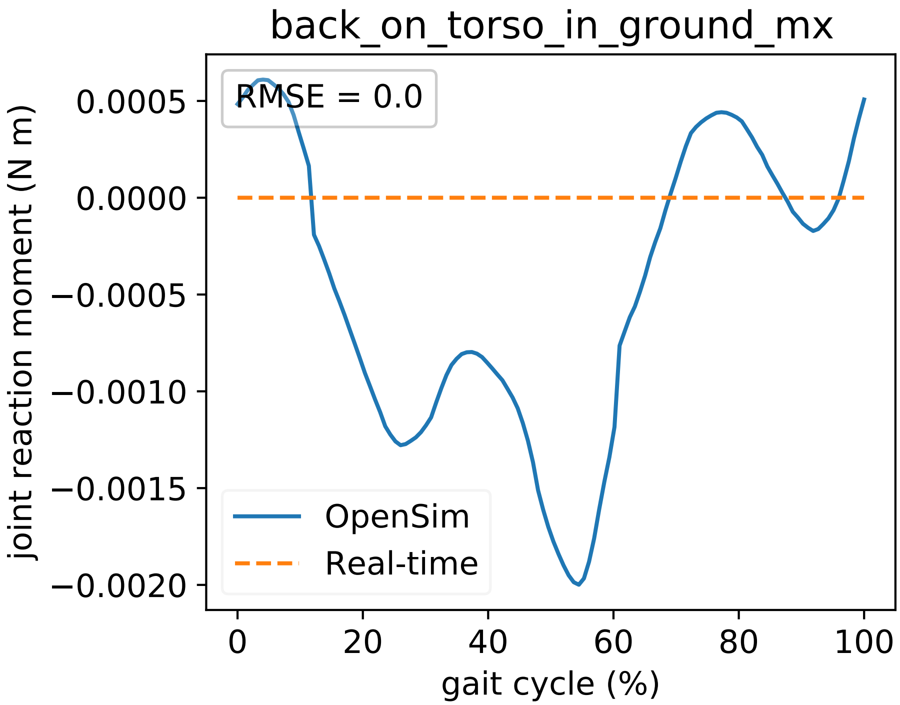

back_on_torso_in_ground_mx
RMSE = 0.0
OpenSim
Real-time
joint reaction moment (N m)
gait cycle (%)
0.0005
0.0000
−0.0005
−0.0010
−0.0015
−0.0020
0
20
40
60
80
100

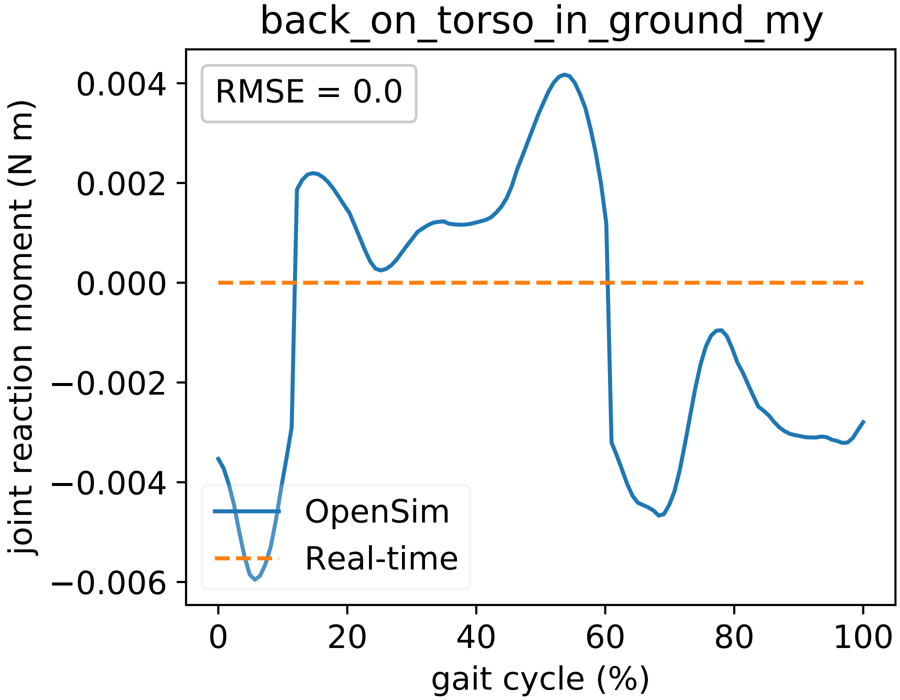
back_on_torso_in_ground_my
RMSE = 0.0
OpenSim
Real-time
joint reaction moment (N m)
gait cycle (%)
0.004
0.002
0.000
−0.002
−0.004
−0.006
0
20
40
60
80
100

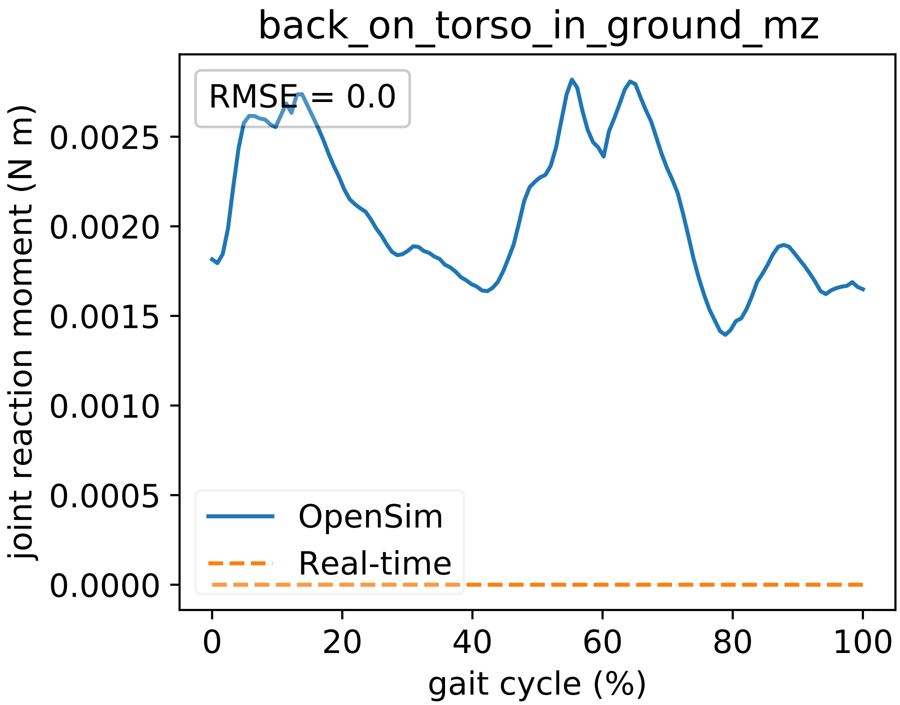
back_on_torso_in_ground_mz
RMSE = 0.0
OpenSim
Real-time
joint reaction moment (N m)
gait cycle (%)
0.0025
0.0020
0.0015
0.0010
0.0005
0.0000
0
20
40
60
80
100

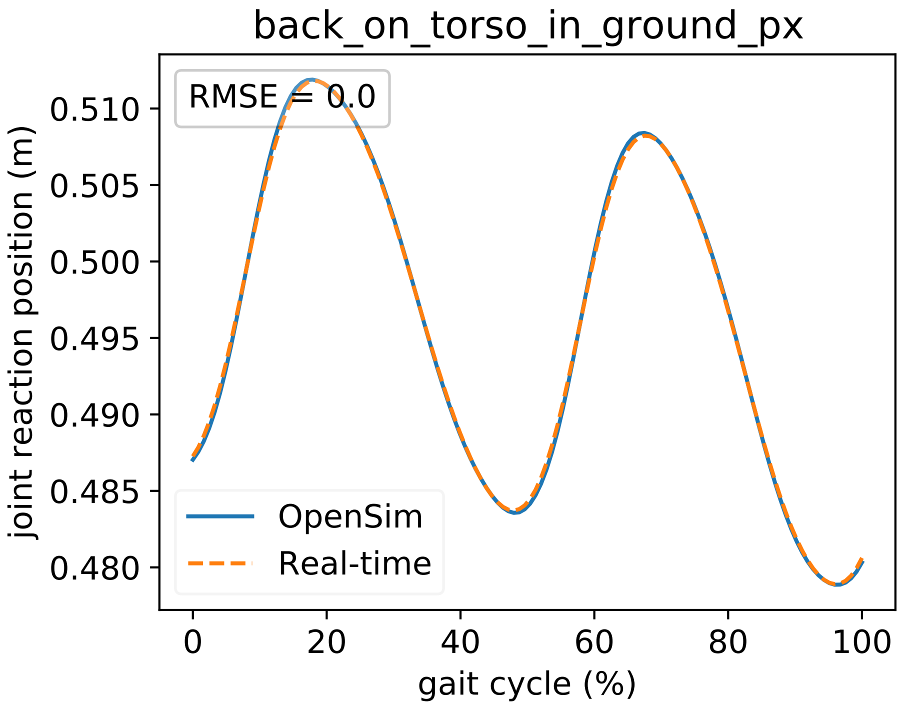
back_on_torso_in_ground_px
RMSE = 0.0
OpenSim
Real-time
joint reaction position (m)
gait cycle (%)
0.510
0.505
0.500
0.495
0.490
0.485
0.480
0
20
40
60
80
100

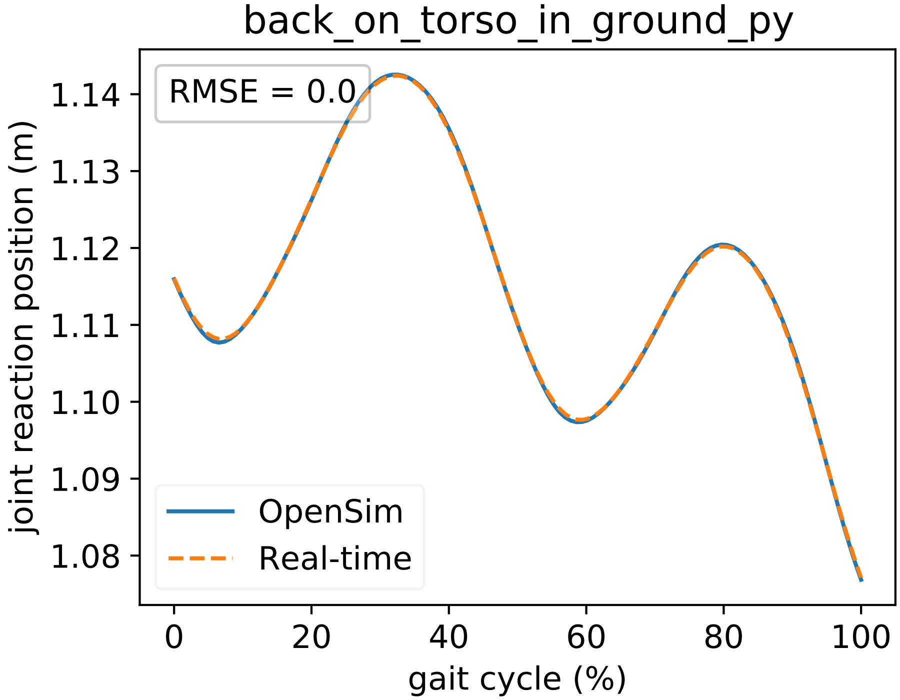
back_on_torso_in_ground_py
RMSE = 0.0
OpenSim
Real-time
joint reaction position (m)
1.14
1.13
1.12
1.11
1.10
1.09
1.08
0
20
40
60
80
100
gait cycle (%)

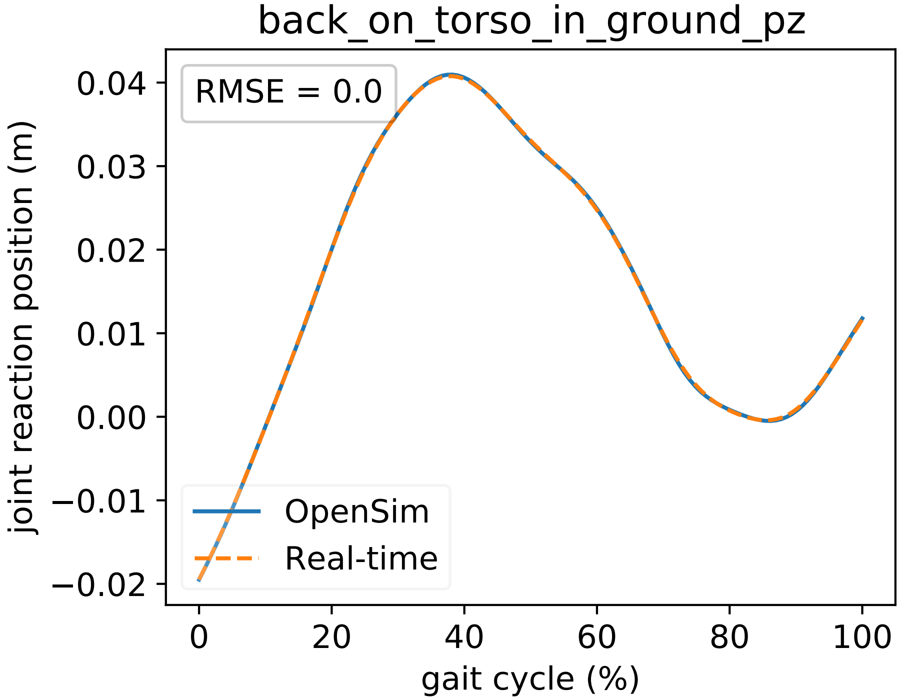
back_on_torso_in_ground_pz
RMSE = 0.0
OpenSim
Real-time
joint reaction position (m)
gait cycle (%)
0.04
0.03
0.02
0.01
0.00
−0.01
−0.02
0
20
40
60
80
100
