# Supplementary material for: Real-Time Musculoskeletal Kinematics and Dynamics Analysis Using Marker- and IMU-Based Solutions in Rehabilitation
Source: Sensors (Basel). 2021 Mar 5;21(5):1804. doi: 10.3390/s21051804 (PMC7961635; doi:10.3390/s21051804)
Supplement: Supplementary file 1 [file sensors-21-01804-s001.zip › supplementary_muscle_optimization.pdf]

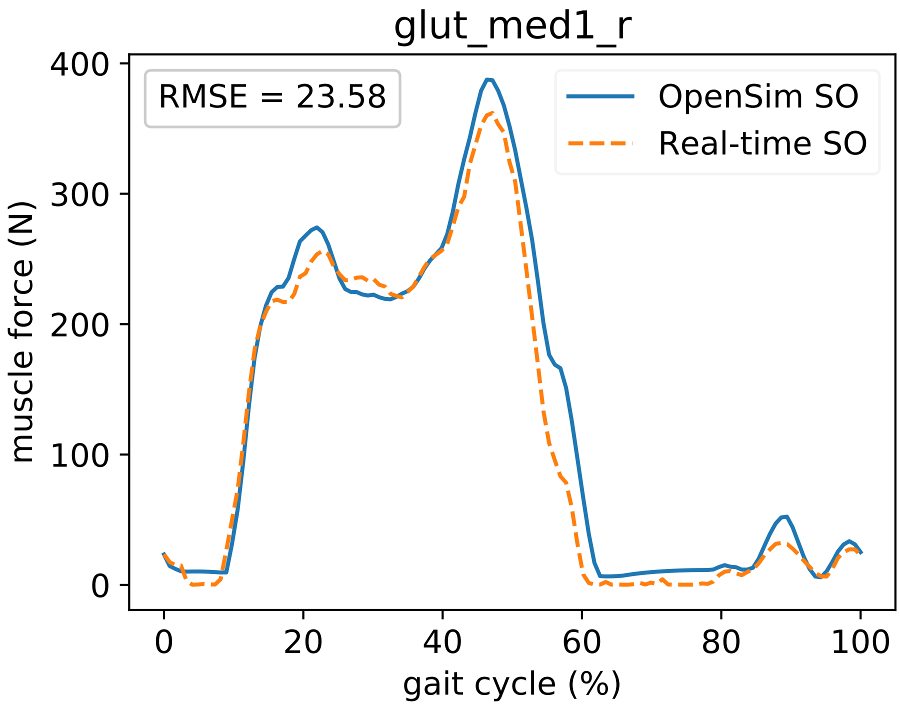

glut_med1_r
RMSE = 23.58
OpenSim SO
Real-time SO
muscle force (N)
gait cycle (%)
0
100
200
300
400
0
20
40
60
80
100

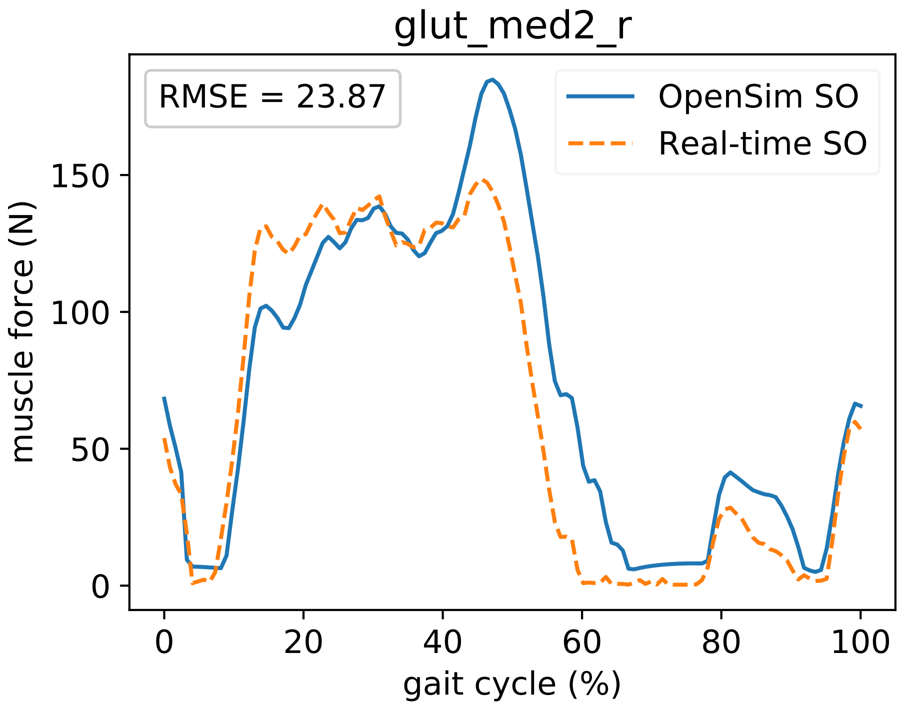
glut_med2_r
RMSE = 23.87
OpenSim SO
Real-time SO
muscle force (N)
0
50
100
150
0
20
40
60
80
100
gait cycle (%)

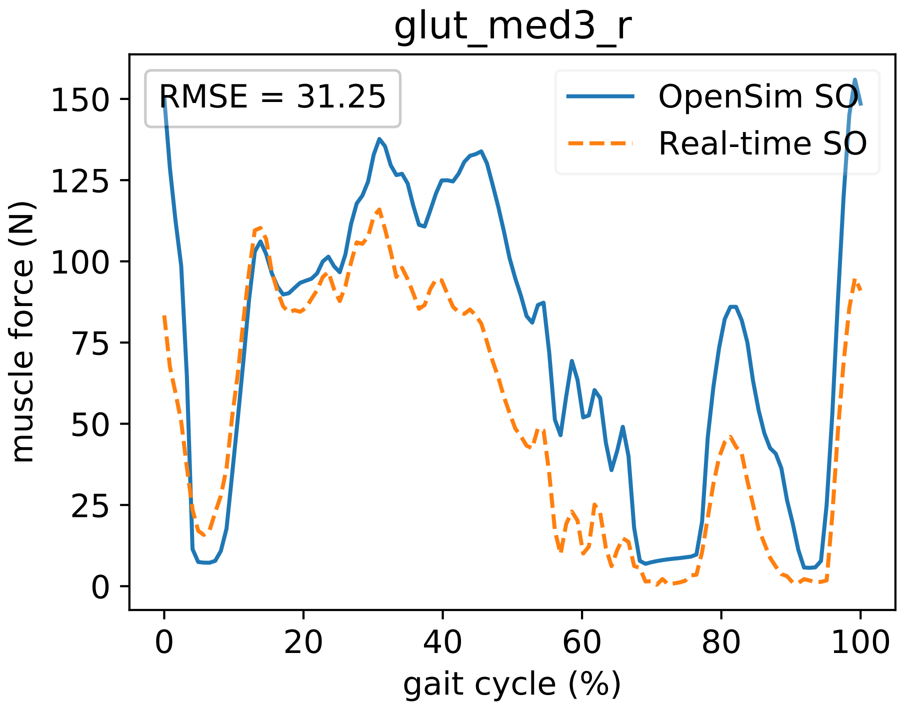
glut_med3_r
RMSE = 31.25
OpenSim SO
Real-time SO
muscle force (N)
gait cycle (%)
0
25
50
75
100
125
150
0
20
40
60
80
100

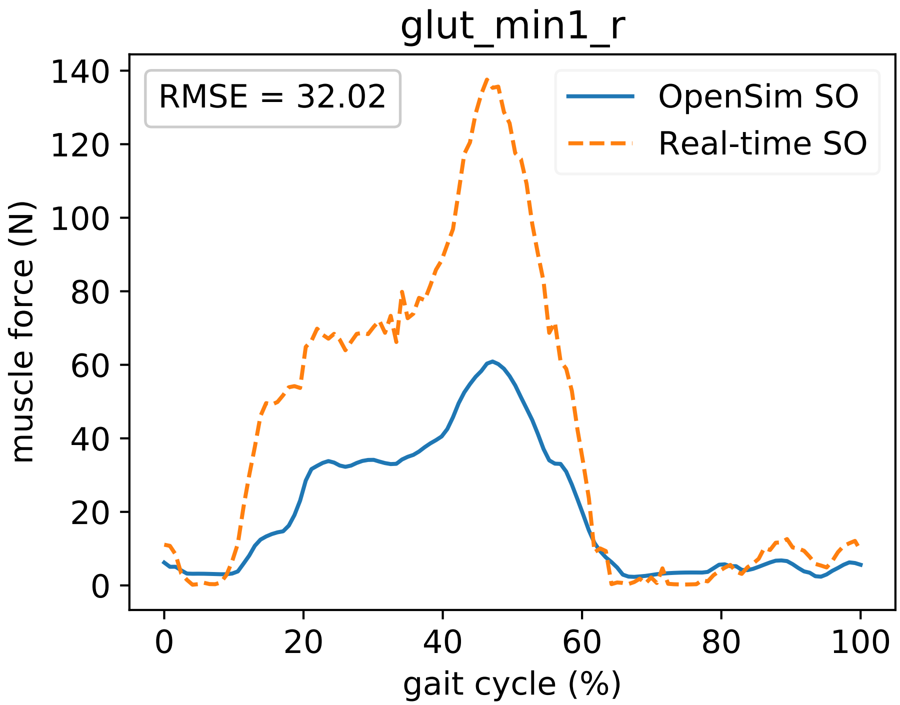
glut_min1_r
RMSE = 32.02
OpenSim SO
Real-time SO
muscle force (N)
gait cycle (%)
0
20
40
60
80
100
120
140

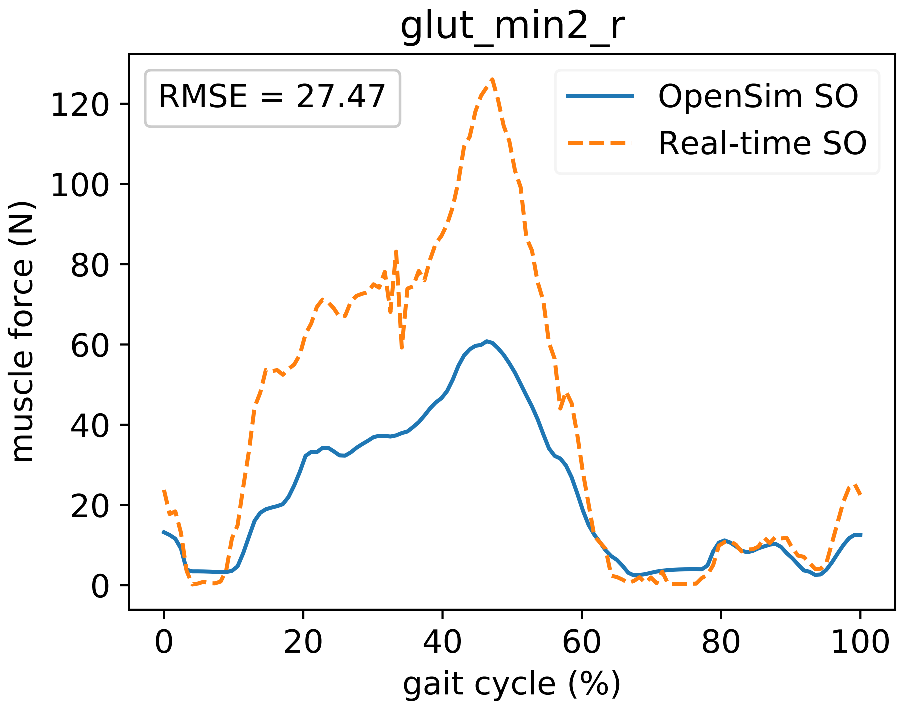

glut_min2_r
RMSE = 27.47
OpenSim SO
Real-time SO
muscle force (N)
gait cycle (%)
0
20
40
60
80
100
120

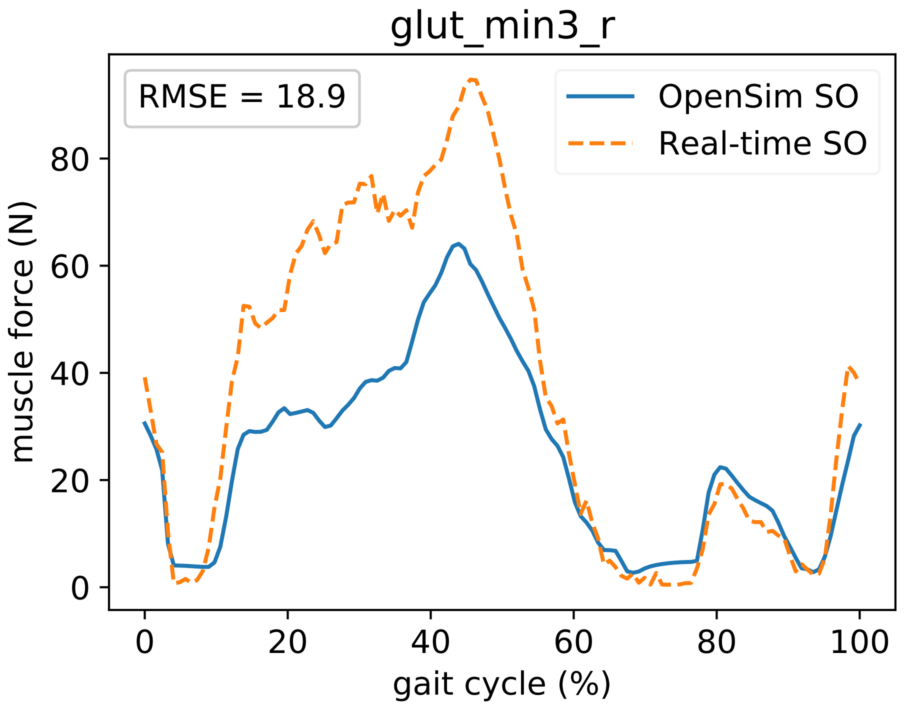
glut_min3_r
RMSE = 18.9
OpenSim SO
Real-time SO
muscle force (N)
0
20
40
60
80
0
20
40
60
80
100
gait cycle (%)

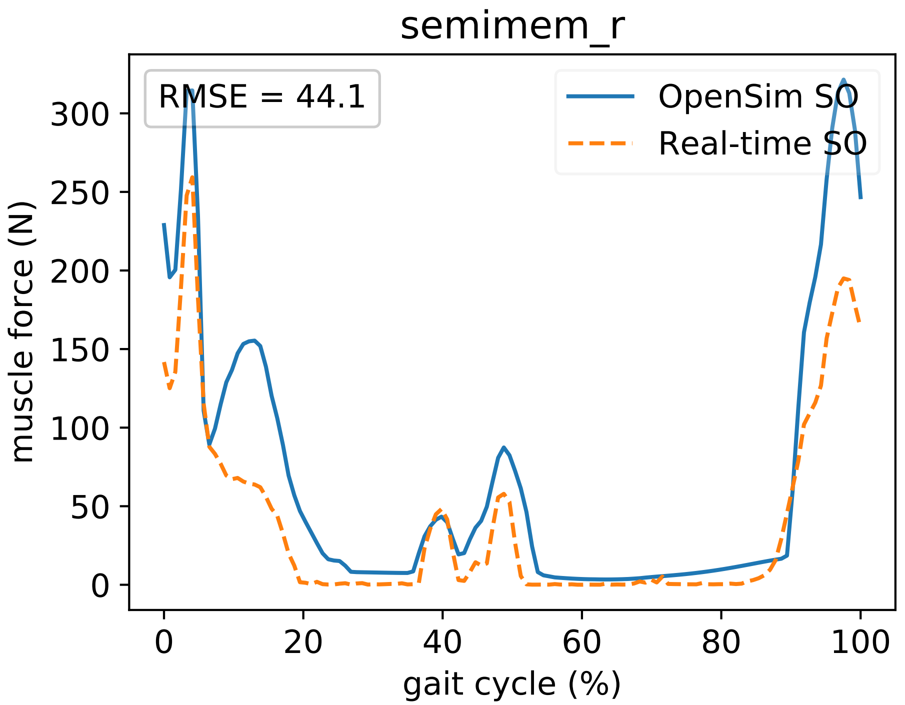

semimem_r
RMSE = 44.1
OpenSim SO
Real-time SO
muscle force (N)
0
50
100
150
200
250
300
0
20
40
60
80
100
gait cycle (%)

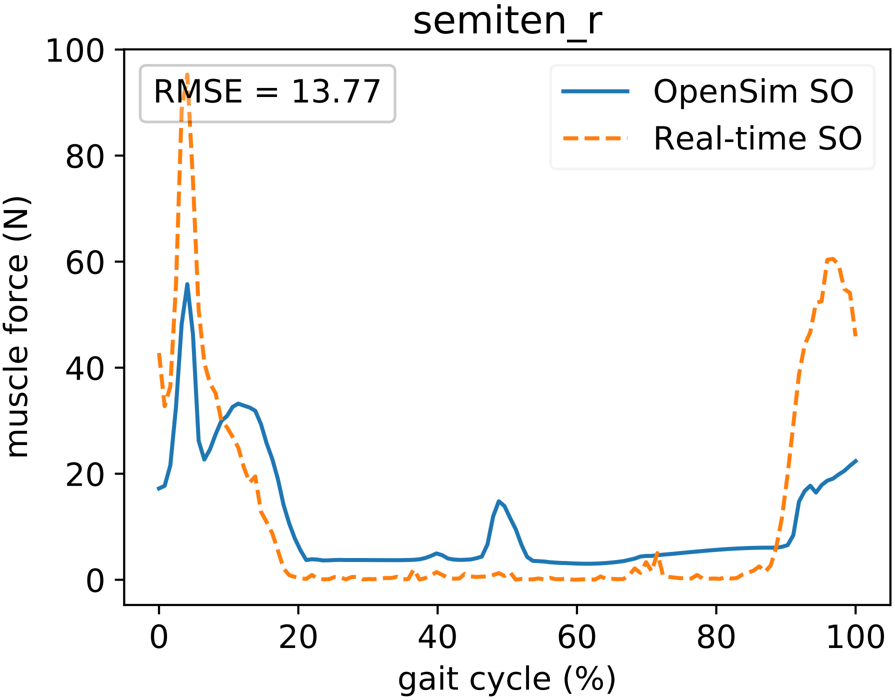

semiten_r
RMSE = 13.77
OpenSim SO
Real-time SO
muscle force (N)
gait cycle (%)
0
20
40
60
80
100
0
20
40
60
80
100

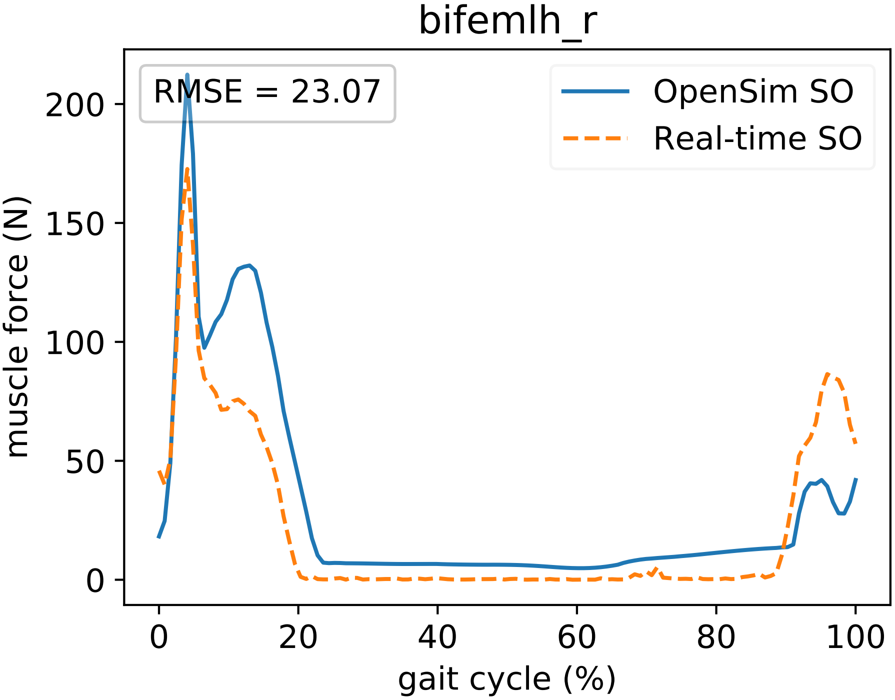

bifemlh_r
RMSE = 23.07
OpenSim SO
Real-time SO
muscle force (N)
0
50
100
150
200
0
20
40
60
80
100
gait cycle (%)

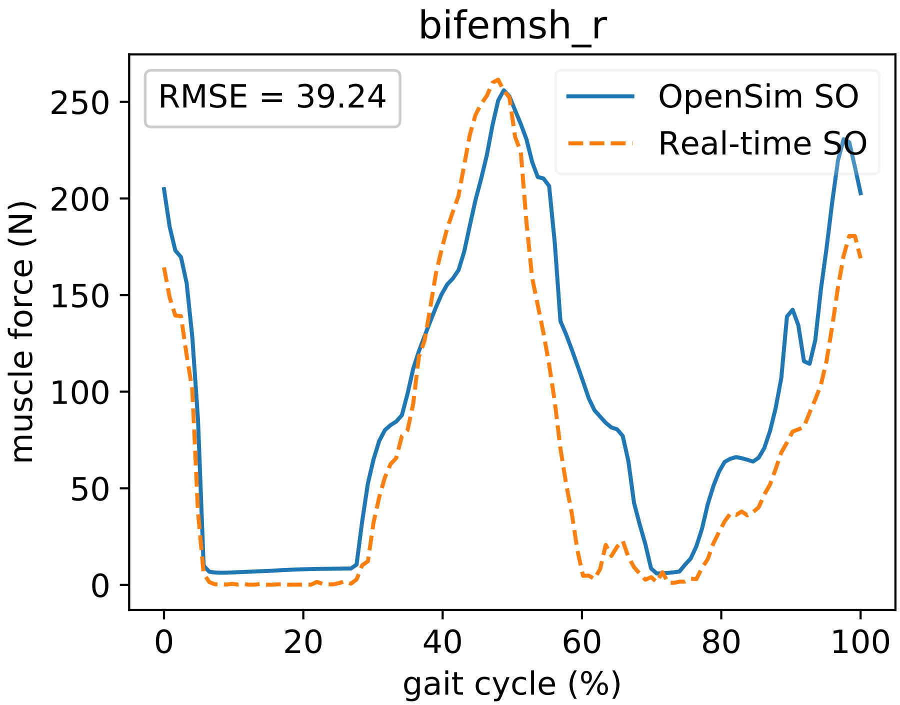
bifemsh_r
RMSE = 39.24
OpenSim SO
Real-time SO
muscle force (N)
gait cycle (%)
0
50
100
150
200
250
0
20
40
60
80
100

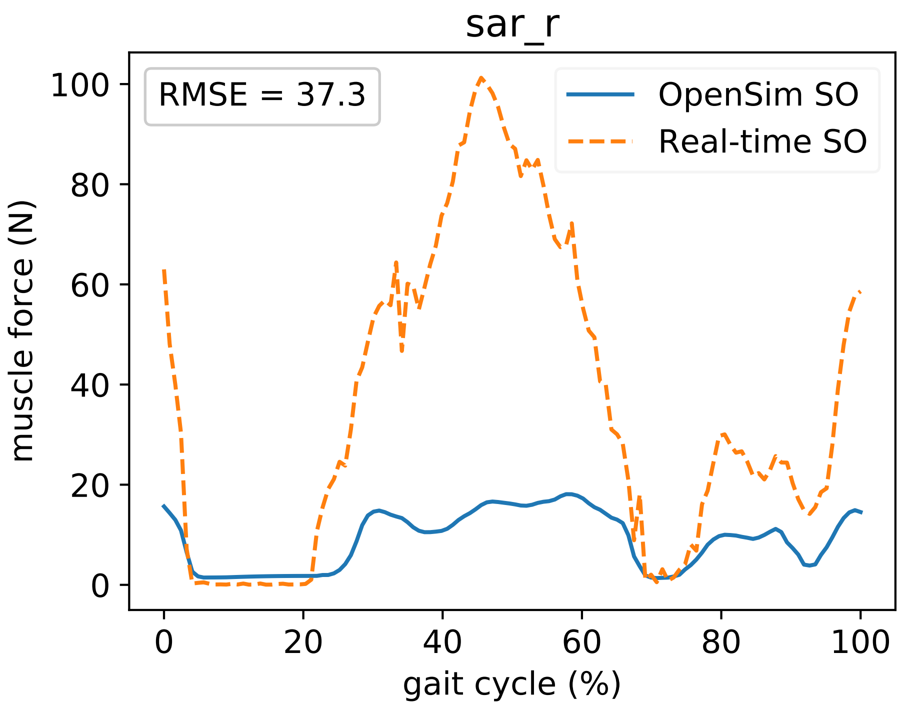
sar_r
RMSE = 37.3
OpenSim SO
Real-time SO
muscle force (N)
gait cycle (%)
0
20
40
60
80
100
0
20
40
60
80
100

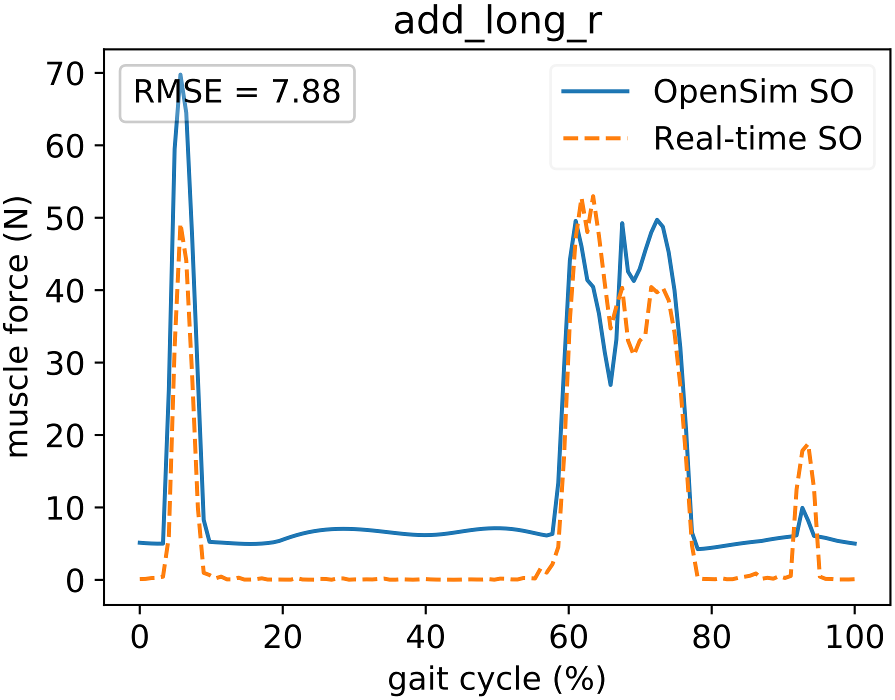
add_long_r
RMSE = 7.88
OpenSim SO
Real-time SO
muscle force (N)
gait cycle (%)
0
10
20
30
40
50
60
70
0
20
40
60
80
100

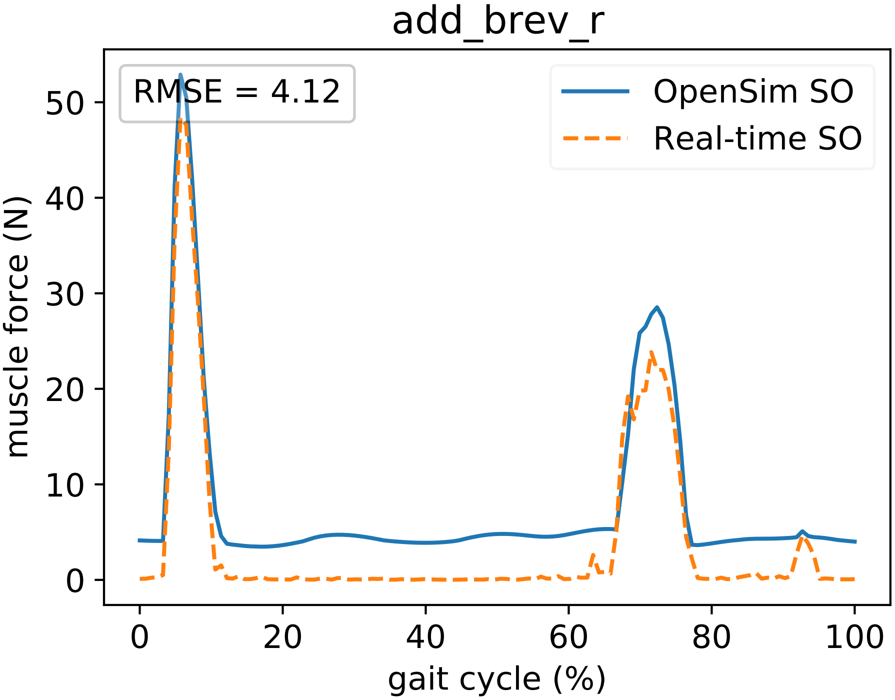
add_brev_r
RMSE = 4.12
OpenSim SO
Real-time SO
muscle force (N)
gait cycle (%)
0
10
20
30
40
50
0
20
40
60
80
100

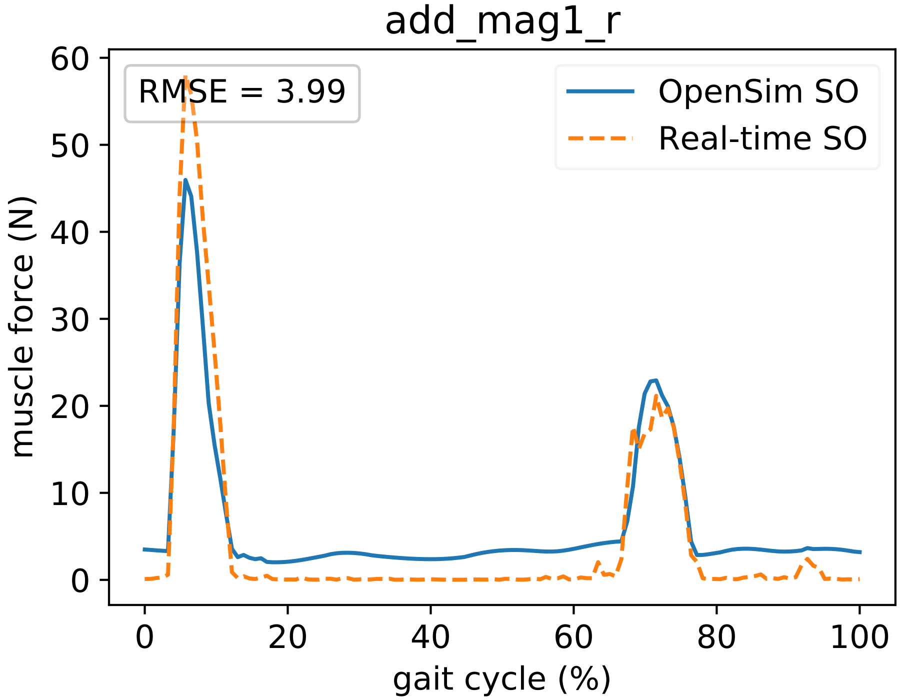

add_mag1_r
RMSE = 3.99
OpenSim SO
Real-time SO
muscle force (N)
gait cycle (%)
0
10
20
30
40
50
60
0
20
40
60
80
100

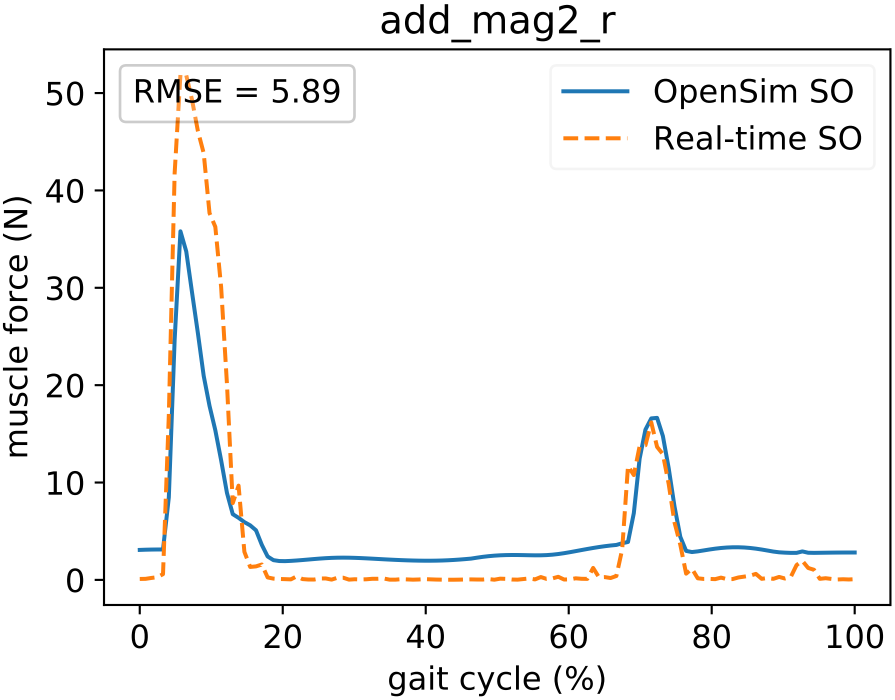

add_mag2_r
RMSE = 5.89
OpenSim SO
Real-time SO
muscle force (N)
gait cycle (%)
0
10
20
30
40
50
0
20
40
60
80
100

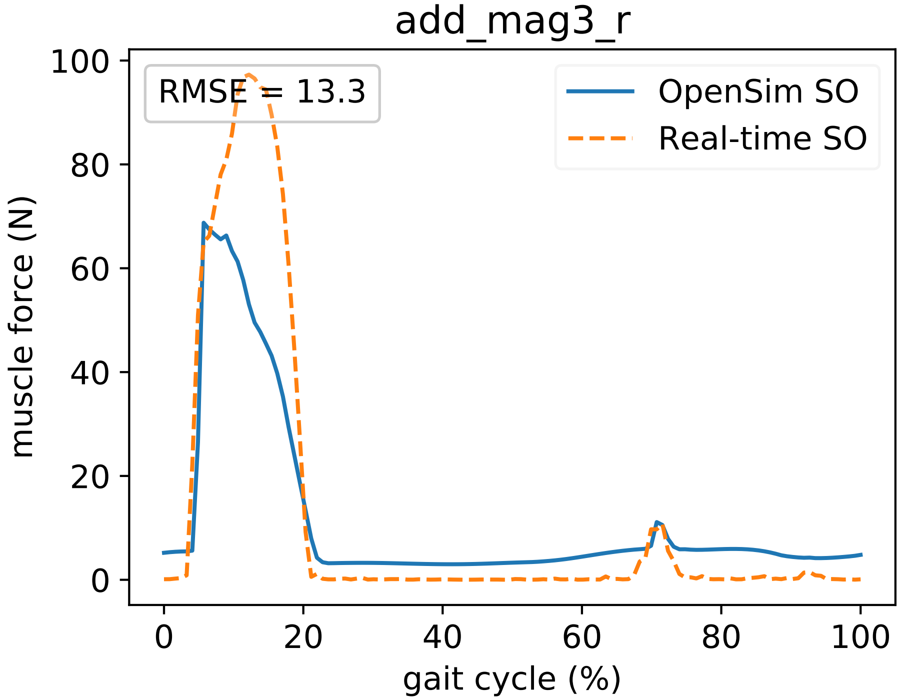
add_mag3_r
RMSE = 13.3
OpenSim SO
Real-time SO
muscle force (N)
gait cycle (%)
0
20
40
60
80
100
0
20
40
60
80
100

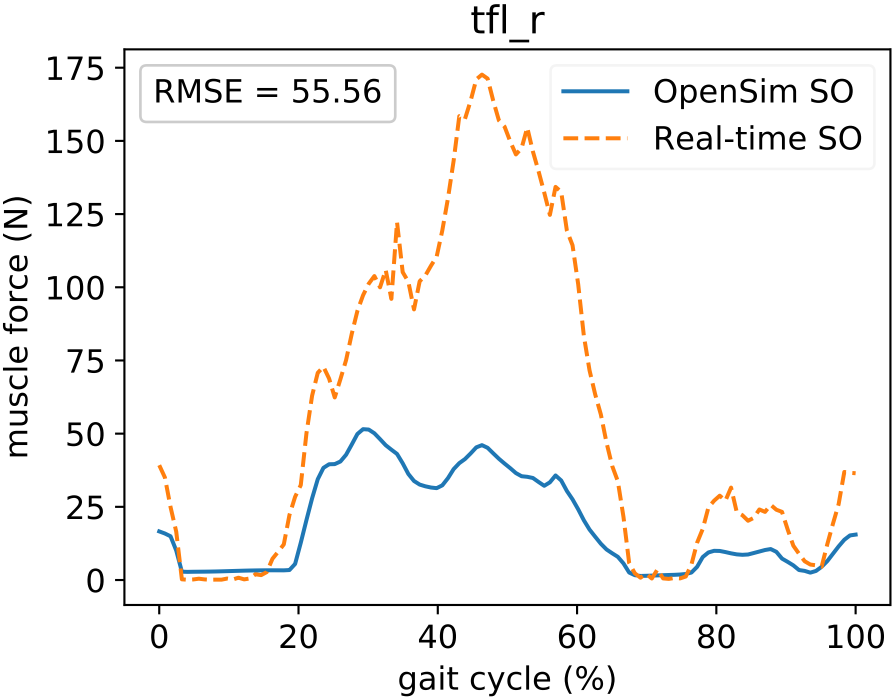
tfl_r
RMSE = 55.56
OpenSim SO
Real-time SO
muscle force (N)
gait cycle (%)
0
25
50
75
100
125
150
175
0
20
40
60
80
100

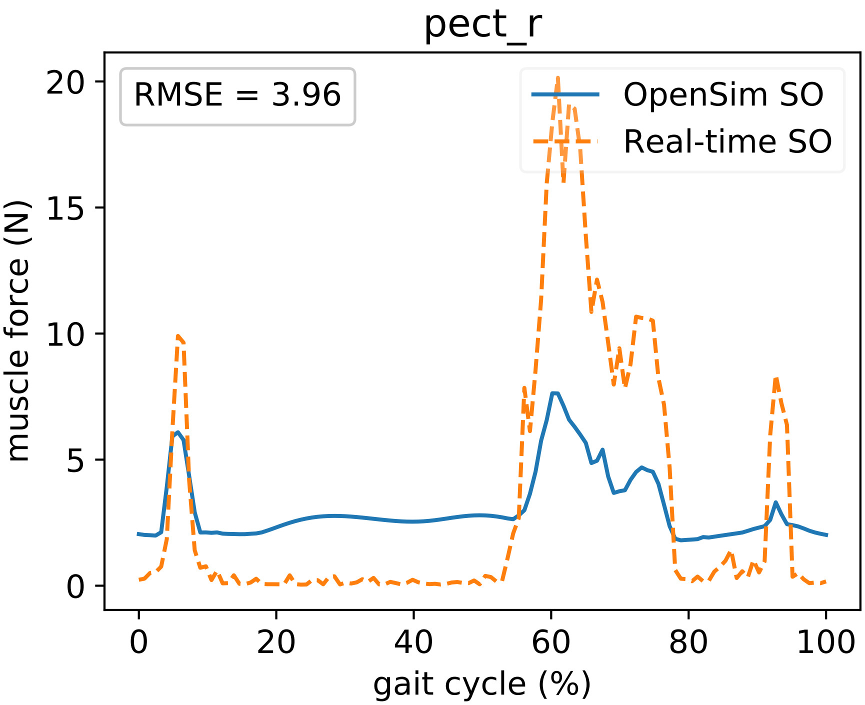
pect_r
RMSE = 3.96
OpenSim SO
Real-time SO
muscle force (N)
0
5
10
15
20
0
20
40
60
80
100
gait cycle (%)

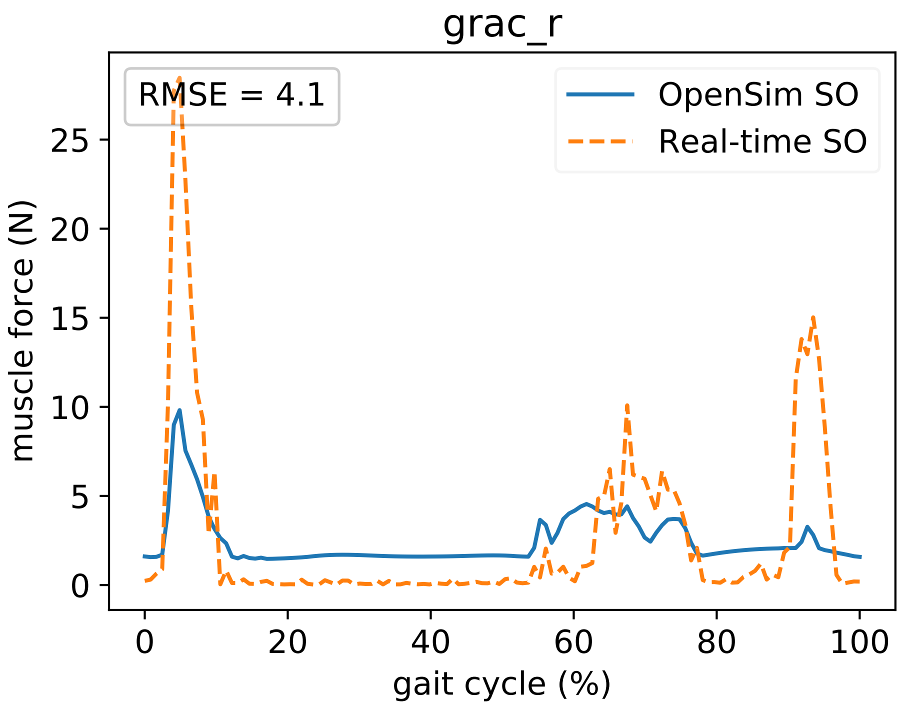
grac_r
RMSE = 4.1
OpenSim SO
Real-time SO
muscle force (N)
0
5
10
15
20
25
0
20
40
60
80
100
gait cycle (%)

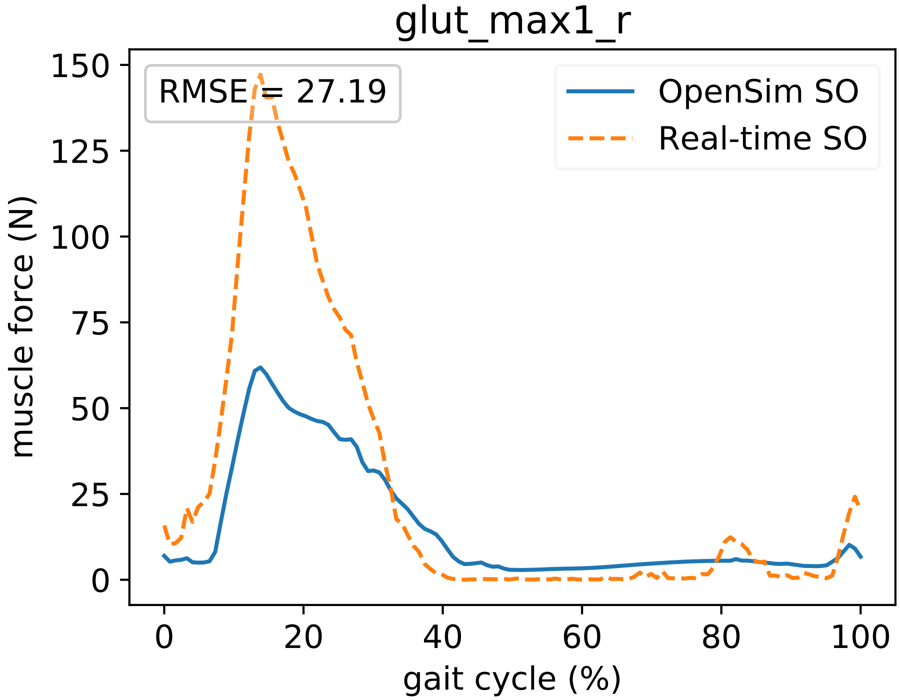

glut_max1_r
RMSE = 27.19
OpenSim SO
Real-time SO
muscle force (N)
gait cycle (%)
0
25
50
75
100
125
150
0
20
40
60
80
100

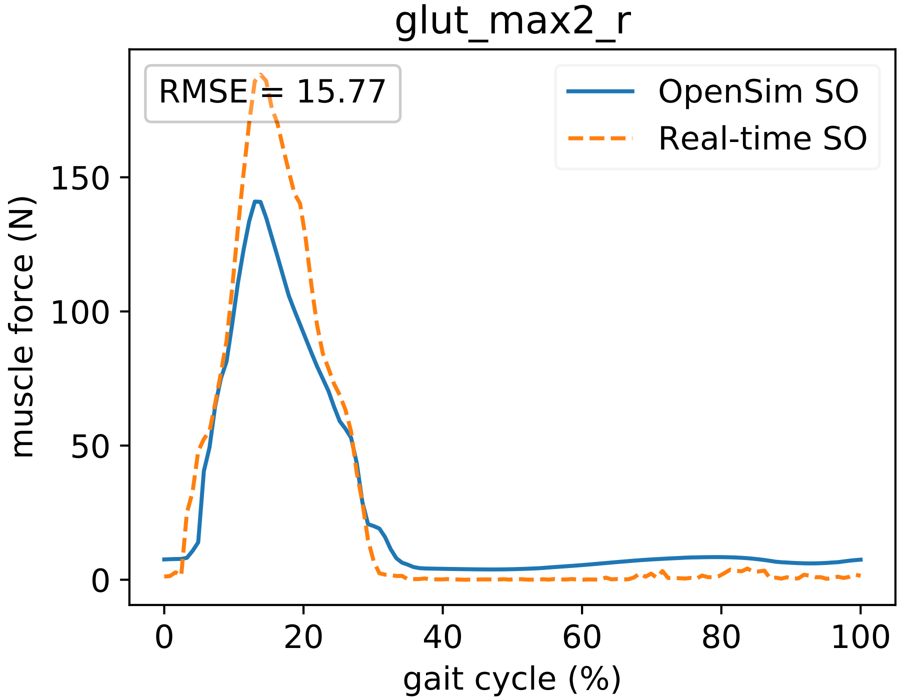

glut_max2_r
RMSE = 15.77
OpenSim SO
Real-time SO
muscle force (N)
0
50
100
150
0
20
40
60
80
100
gait cycle (%)

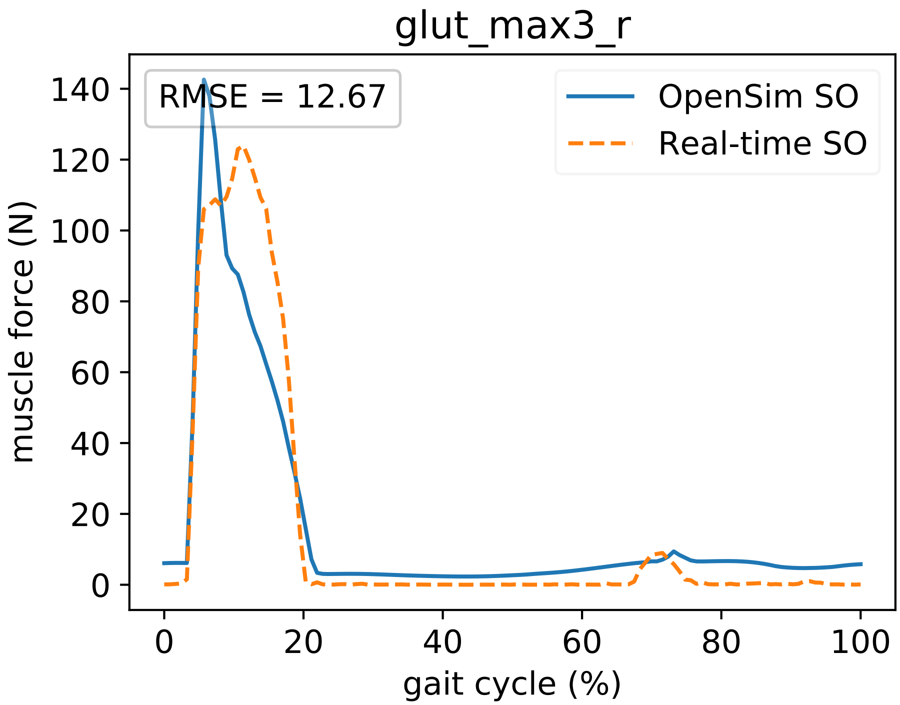
glut_max3_r
RMSE = 12.67
OpenSim SO
Real-time SO
muscle force (N)
gait cycle (%)
0
20
40
60
80
100
120
140

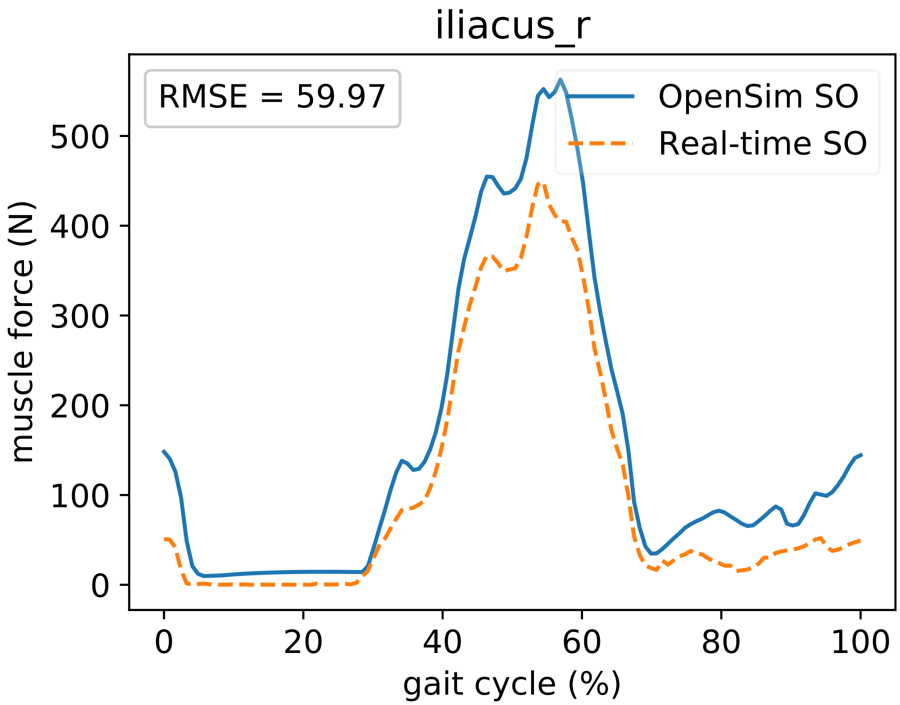
iliacus_r
RMSE = 59.97
OpenSim SO
Real-time SO
muscle force (N)
0
100
200
300
400
500
0
20
40
60
80
100
gait cycle (%)

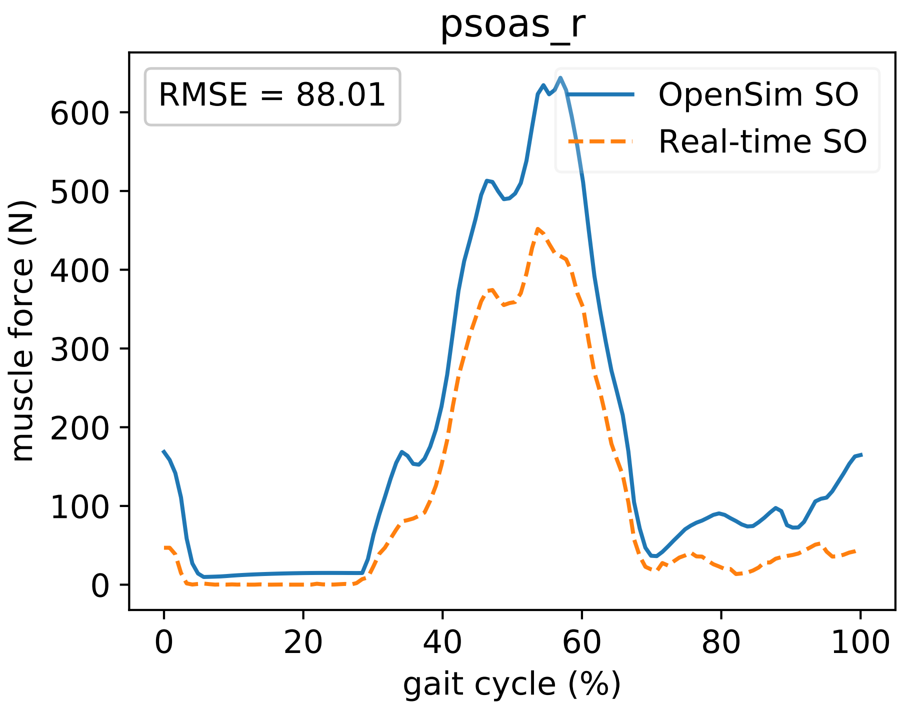
psoas_r
RMSE = 88.01
OpenSim SO
Real-time SO
muscle force (N)
gait cycle (%)
0
100
200
300
400
500
600
0
20
40
60
80
100

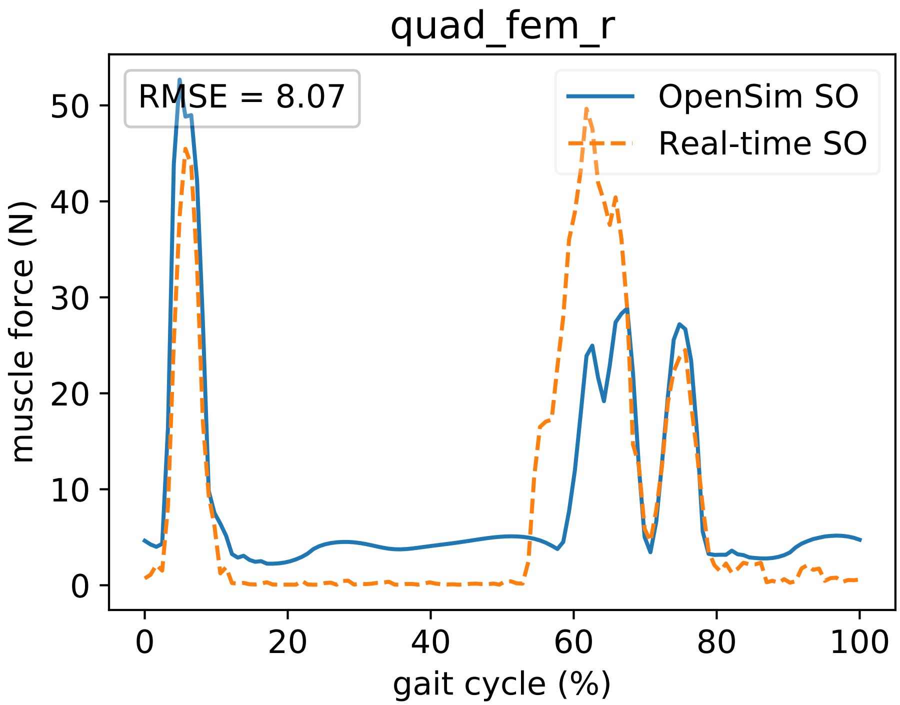
quad_fem_r
RMSE = 8.07
OpenSim SO
Real-time SO
muscle force (N)
gait cycle (%)
0
10
20
30
40
50
0
20
40
60
80
100

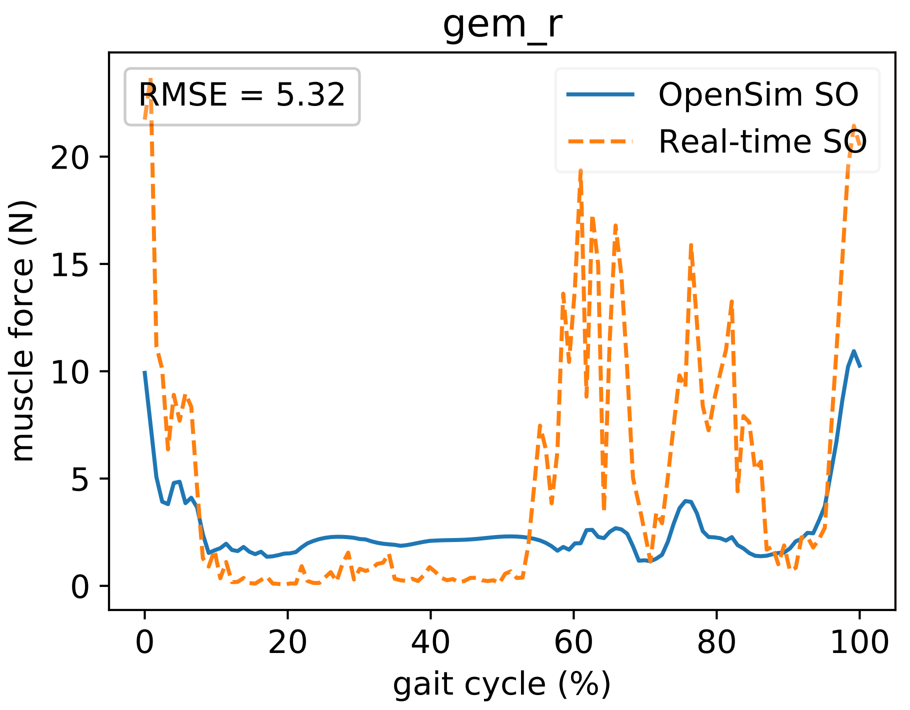
gem_r
RMSE = 5.32
OpenSim SO
Real-time SO
muscle force (N)
gait cycle (%)
0
5
10
15
20
0
20
40
60
80
100

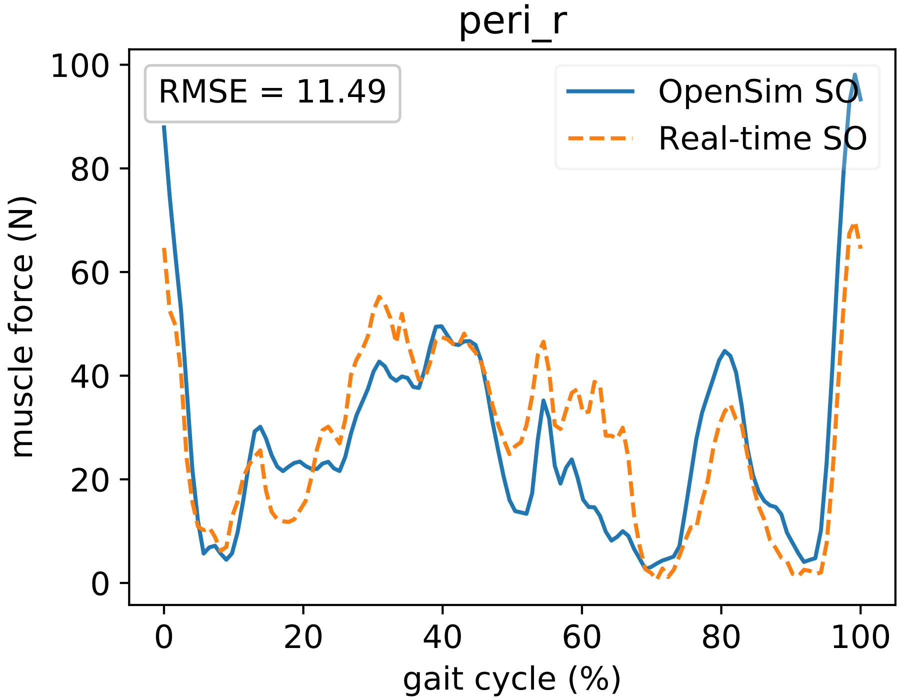

peri_r
RMSE = 11.49
OpenSim SO
Real-time SO
muscle force (N)
gait cycle (%)
0
20
40
60
80
100
0
20
40
60
80
100

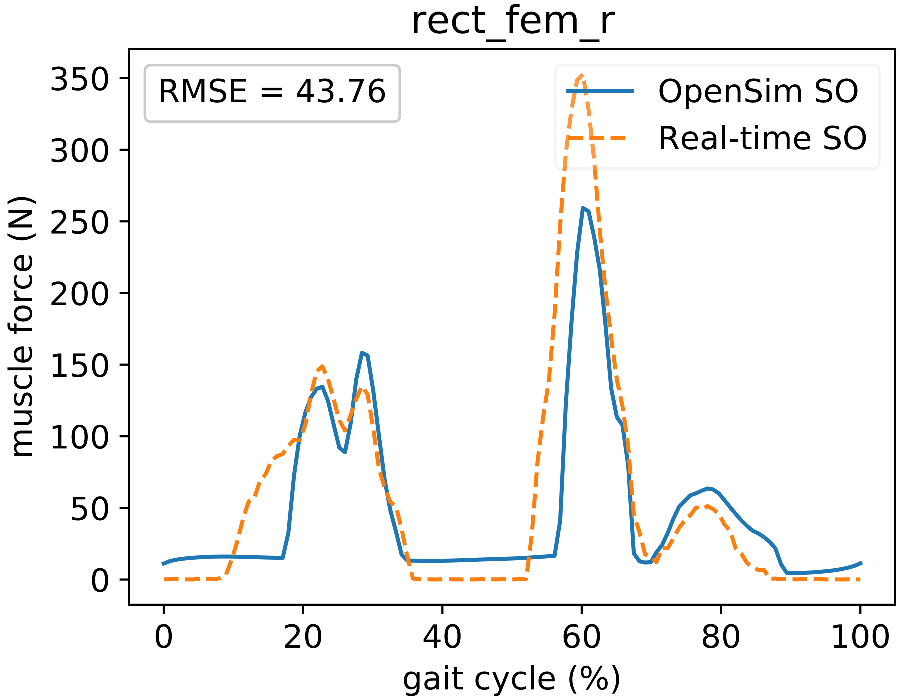
rect_fem_r
RMSE = 43.76
OpenSim SO
Real-time SO
muscle force (N)
0
50
100
150
200
250
300
350
0
20
40
60
80
100
gait cycle (%)

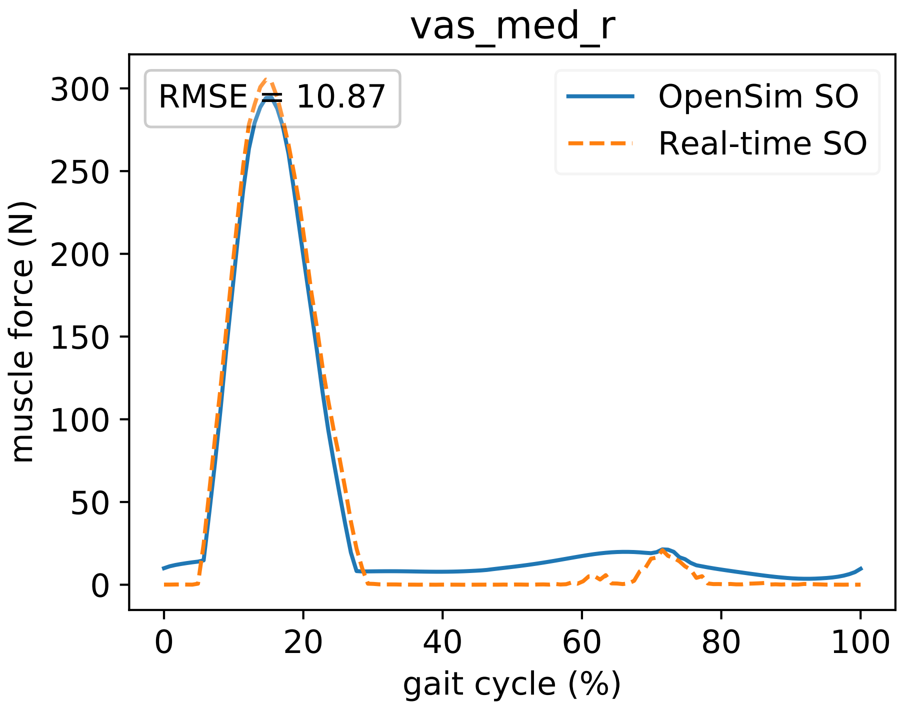

vas_med_r
RMSE = 10.87
OpenSim SO
Real-time SO
muscle force (N)
300
250
200
150
100
50
0
0
20
40
60
80
100
gait cycle (%)

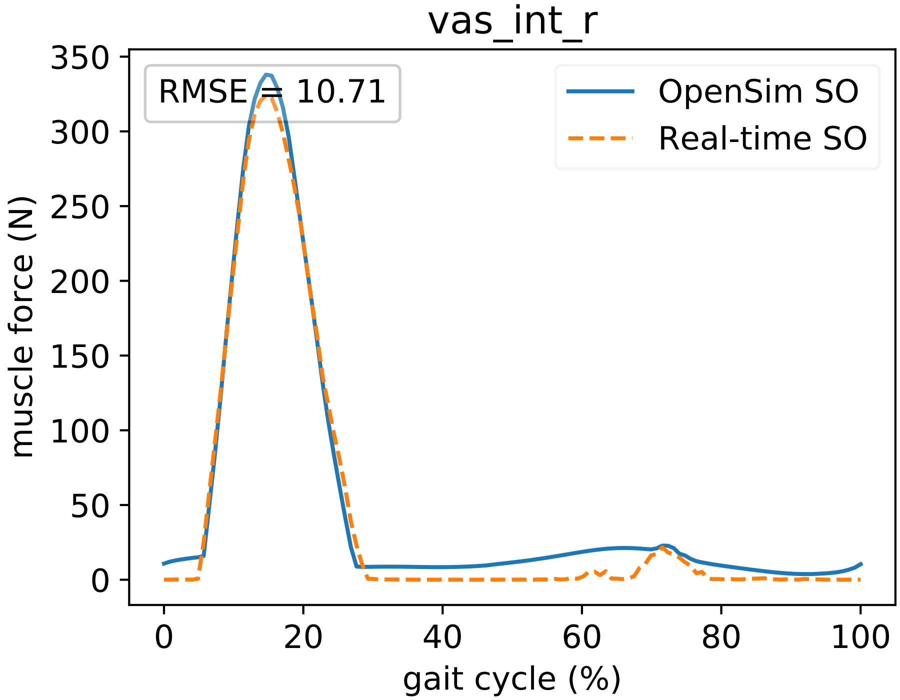
vas_int_r
RMSE = 10.71
OpenSim SO
Real-time SO
muscle force (N)
gait cycle (%)
0
50
100
150
200
250
300
350
0
20
40
60
80
100

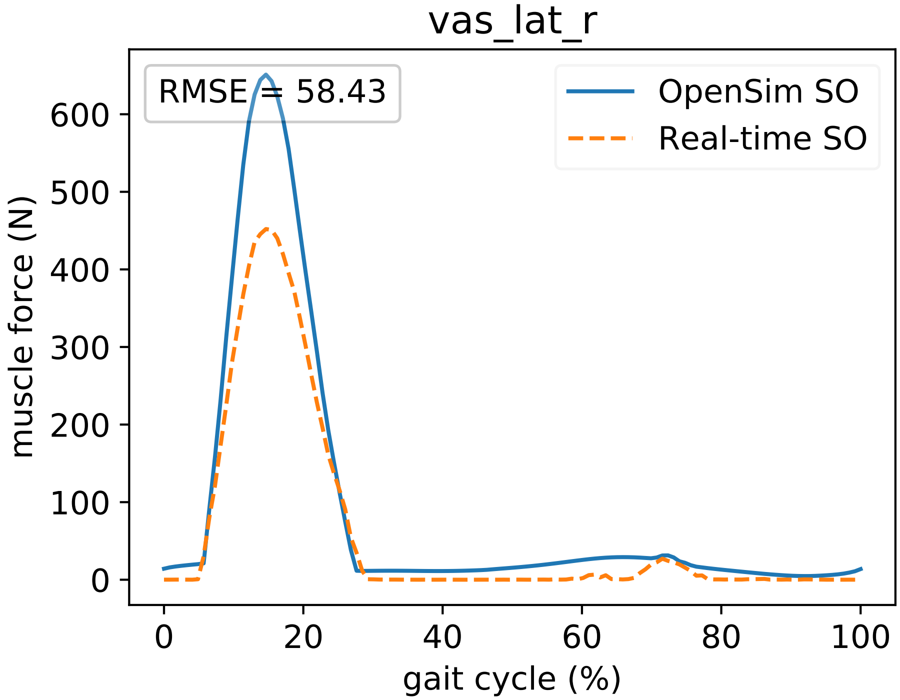

vas_lat_r
RMSE = 58.43
OpenSim SO
Real-time SO
muscle force (N)
gait cycle (%)
0
100
200
300
400
500
600
0
20
40
60
80
100

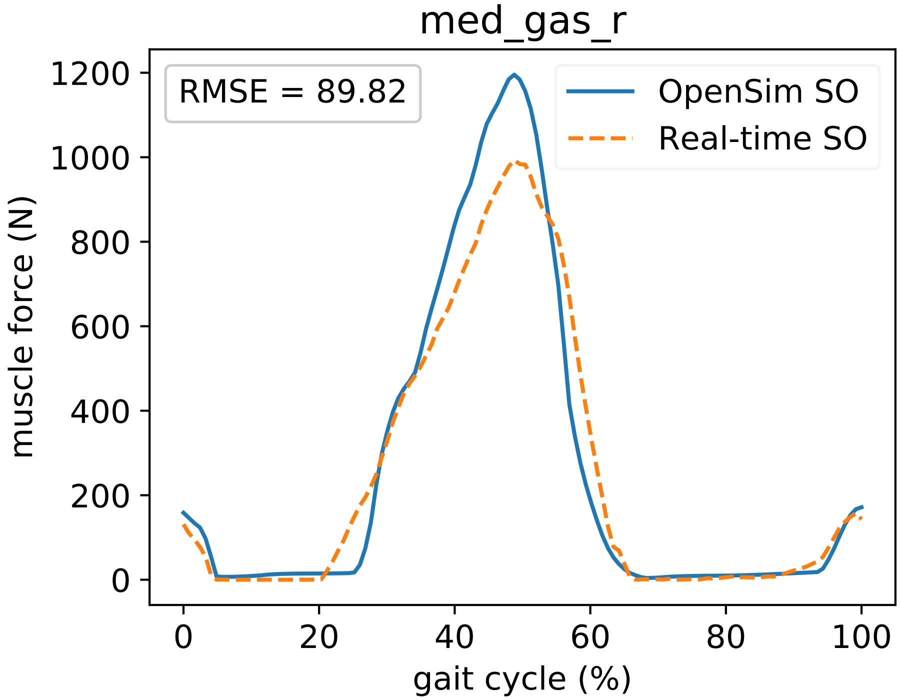

med_gas_r
RMSE = 89.82
OpenSim SO
Real-time SO
muscle force (N)
gait cycle (%)
0
200
400
600
800
1000
1200
0
20
40
60
80
100

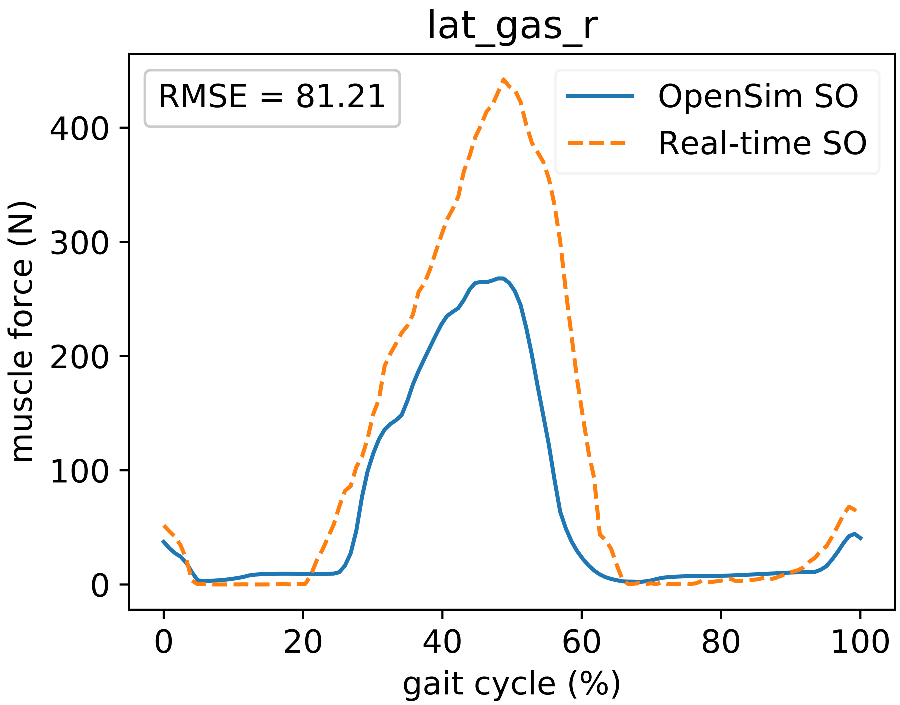

lat_gas_r
RMSE = 81.21
OpenSim SO
Real-time SO
muscle force (N)
gait cycle (%)
0
100
200
300
400
0
20
40
60
80
100

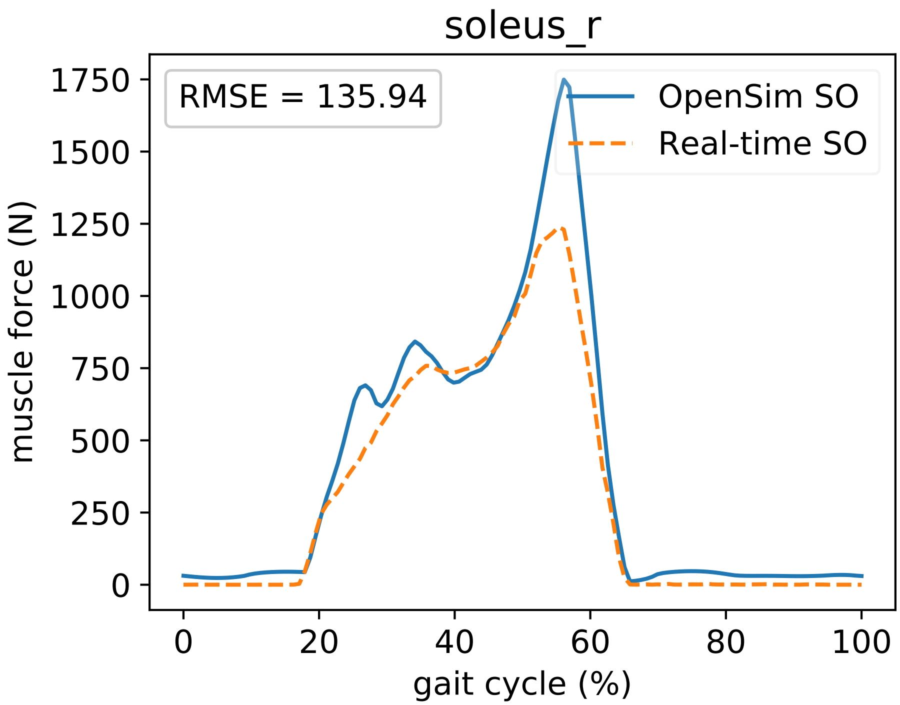

soleus_r
RMSE = 135.94
OpenSim SO
Real-time SO
muscle force (N)
0
250
500
750
1000
1250
1500
1750
0
20
40
60
80
100
gait cycle (%)

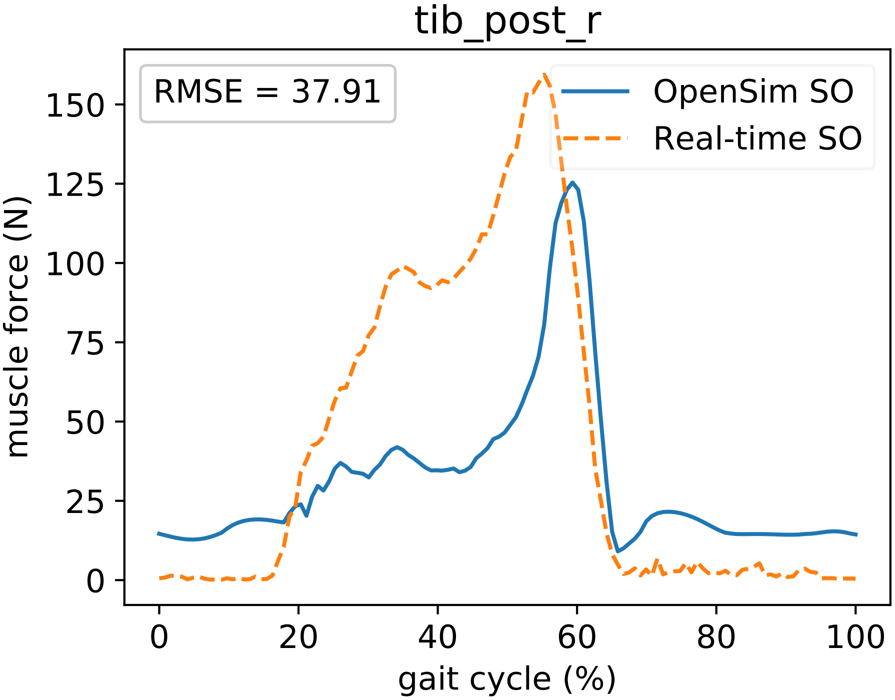
tib_post_r
RMSE = 37.91
OpenSim SO
Real-time SO
muscle force (N)
gait cycle (%)
0
25
50
75
100
125
150
0
20
40
60
80
100

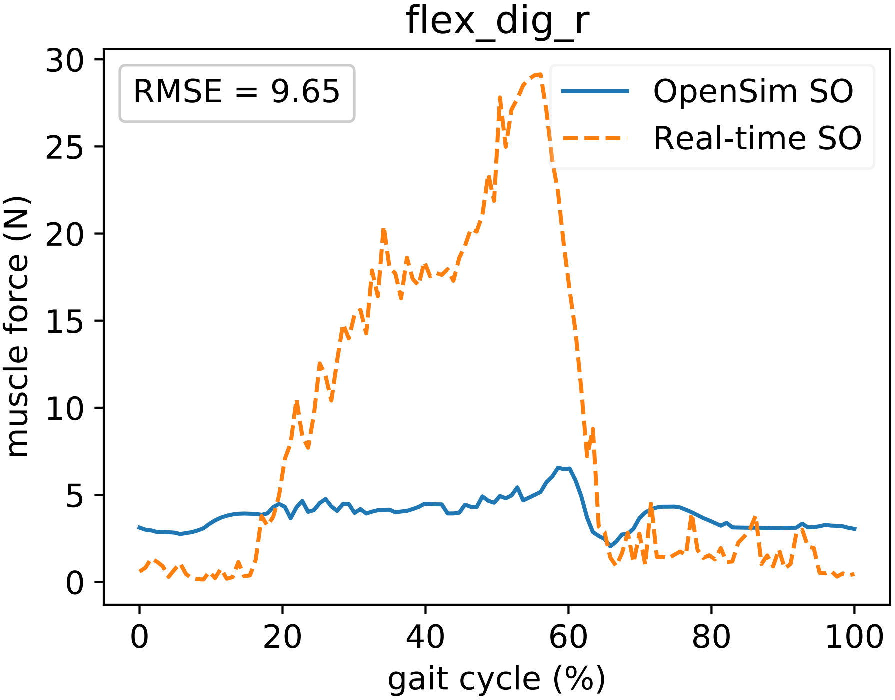
flex_dig_r
RMSE = 9.65
OpenSim SO
Real-time SO
muscle force (N)
gait cycle (%)
0
5
10
15
20
25
30
0
20
40
60
80
100

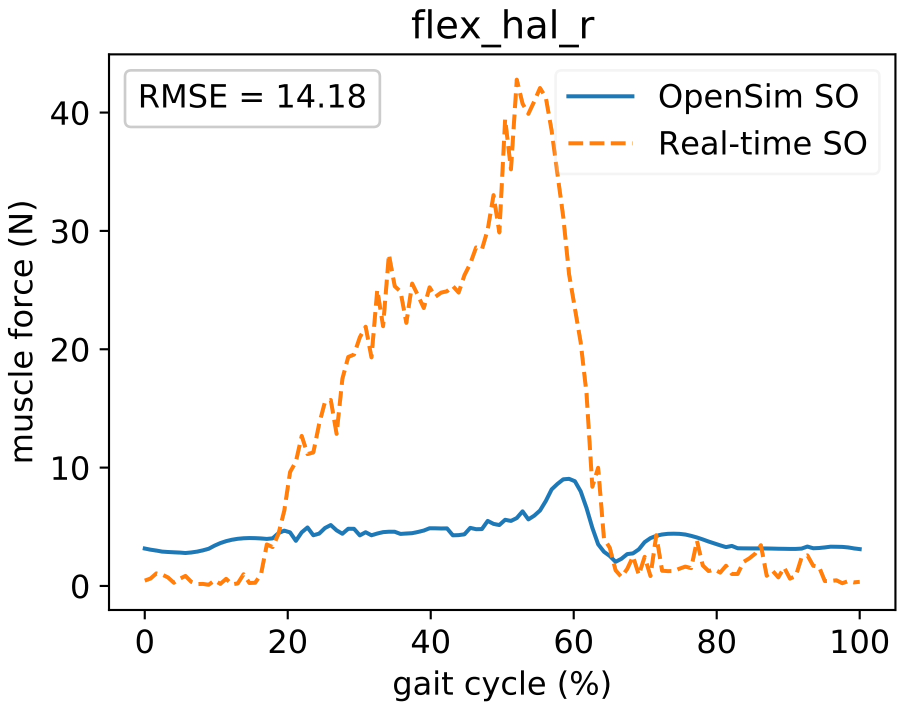

flex_hal_r
RMSE = 14.18
OpenSim SO
Real-time SO
muscle force (N)
0
10
20
30
40
0
20
40
60
80
100
gait cycle (%)

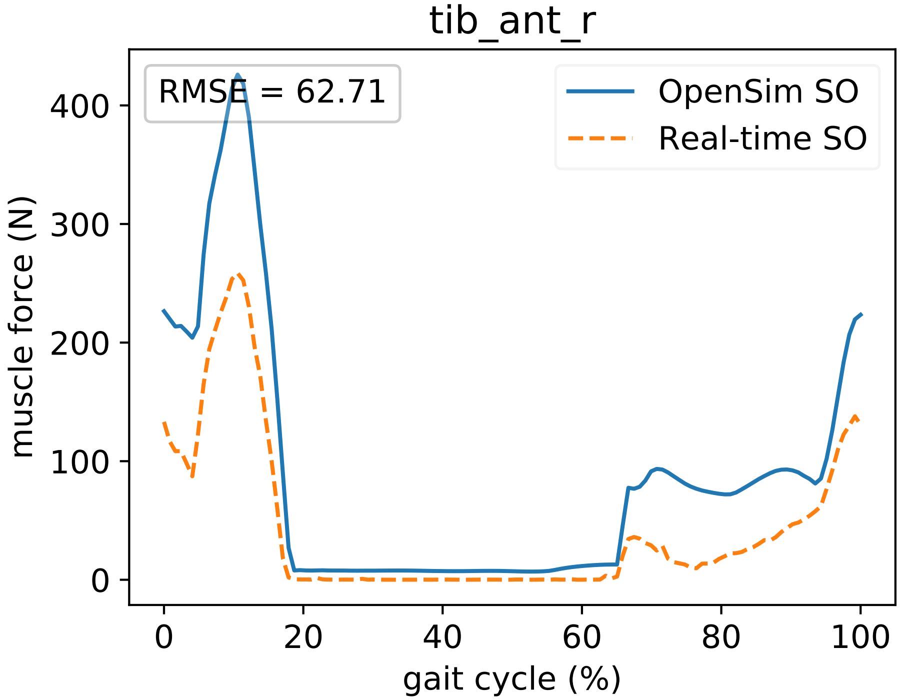
tib_ant_r
RMSE = 62.71
OpenSim SO
Real-time SO
muscle force (N)
400
300
200
100
0
0
20
40
60
80
100
gait cycle (%)

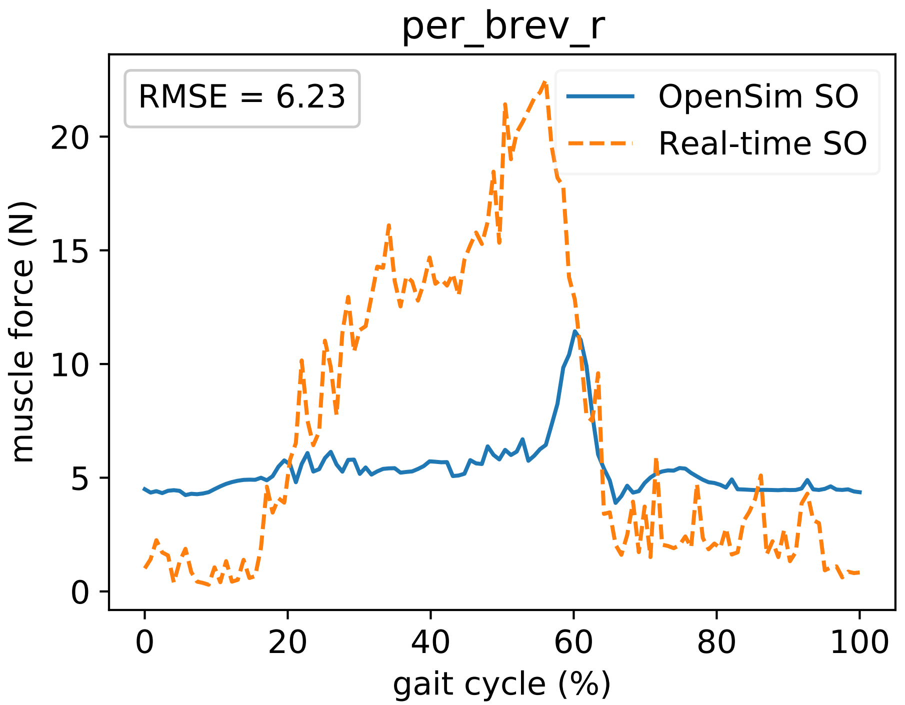
per_brev_r
RMSE = 6.23
OpenSim SO
Real-time SO
muscle force (N)
gait cycle (%)
0
5
10
15
20
0
20
40
60
80
100

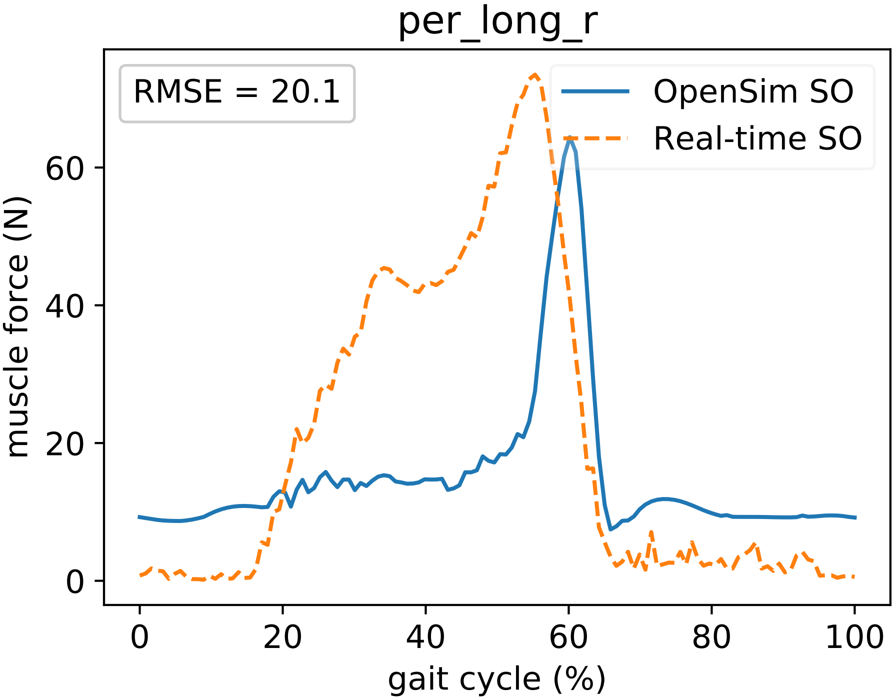
per_long_r
RMSE = 20.1
OpenSim SO
Real-time SO
muscle force (N)
0
20
40
60
0
20
40
60
80
100
gait cycle (%)

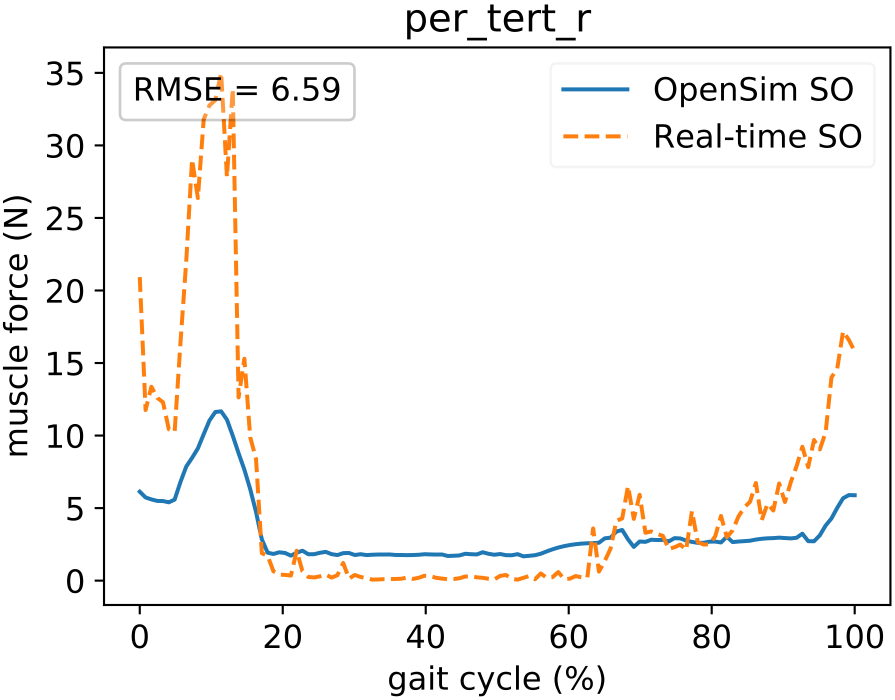

per_tert_r
RMSE = 6.59
OpenSim SO
Real-time SO
muscle force (N)
gait cycle (%)
0
5
10
15
20
25
30
35
0
20
40
60
80
100

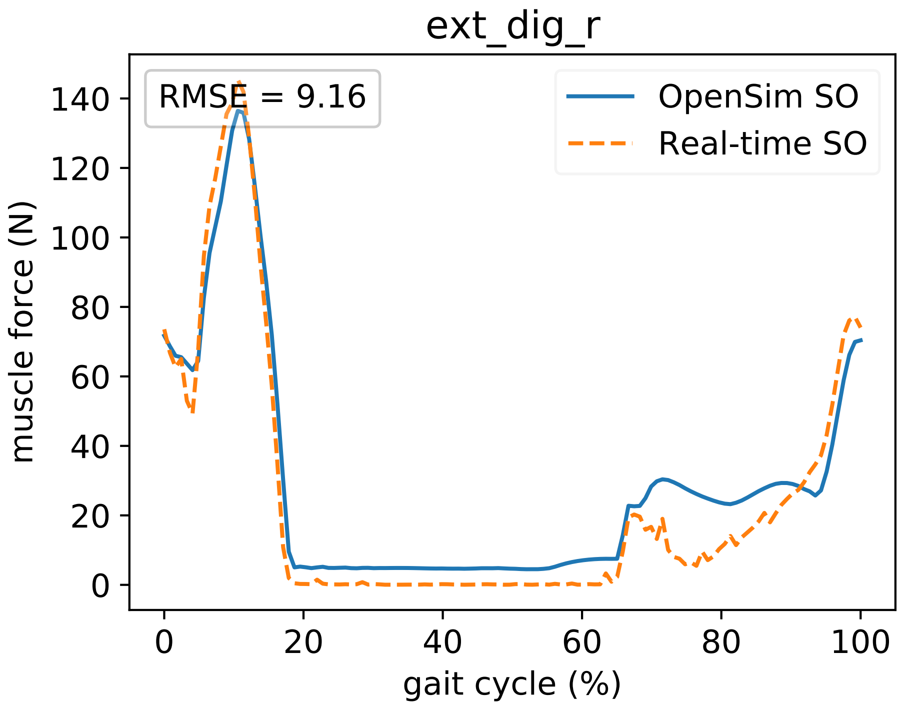
ext_dig_r
RMSE = 9.16
OpenSim SO
Real-time SO
muscle force (N)
gait cycle (%)
0
20
40
60
80
100
120
140
0
20
40
60
80
100

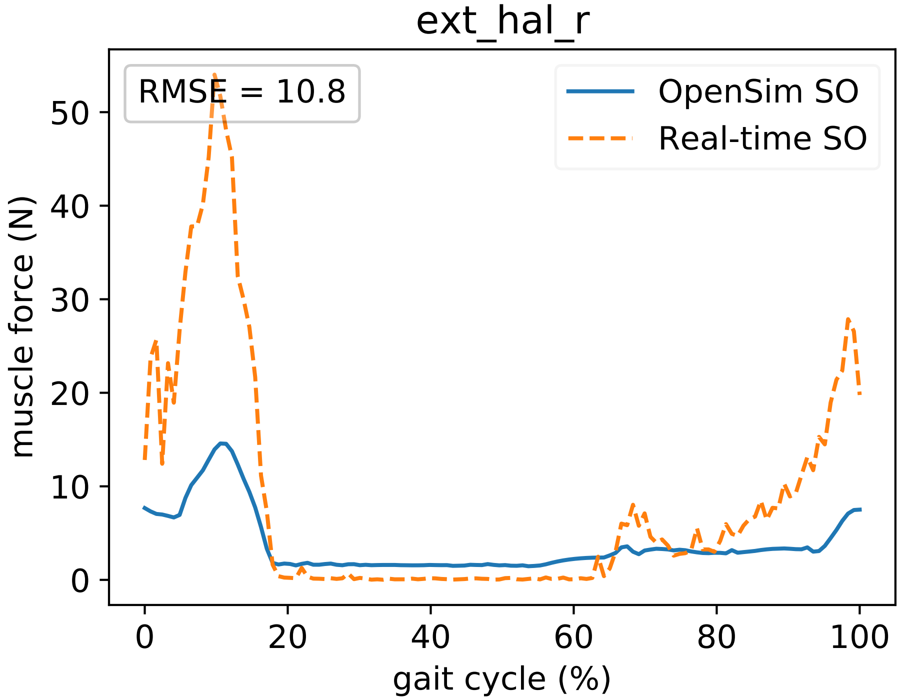
ext_hal_r
RMSE = 10.8
OpenSim SO
Real-time SO
muscle force (N)
gait cycle (%)
0
10
20
30
40
50
0
20
40
60
80
100

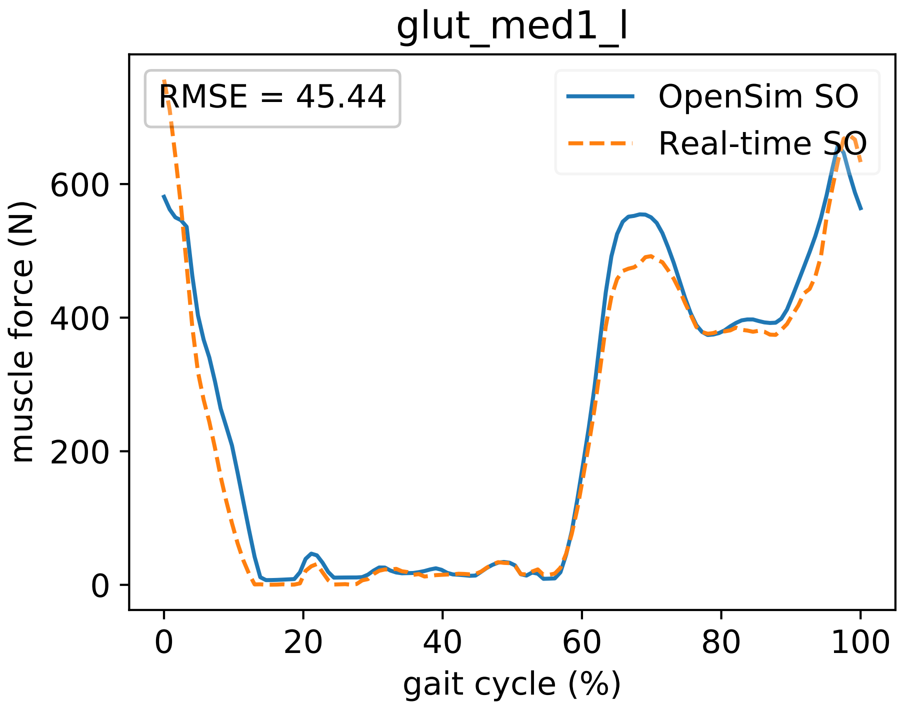

glut_med1_l
RMSE = 45.44
OpenSim SO
Real-time SO
muscle force (N)
gait cycle (%)
0
200
400
600
0
20
40
60
80
100

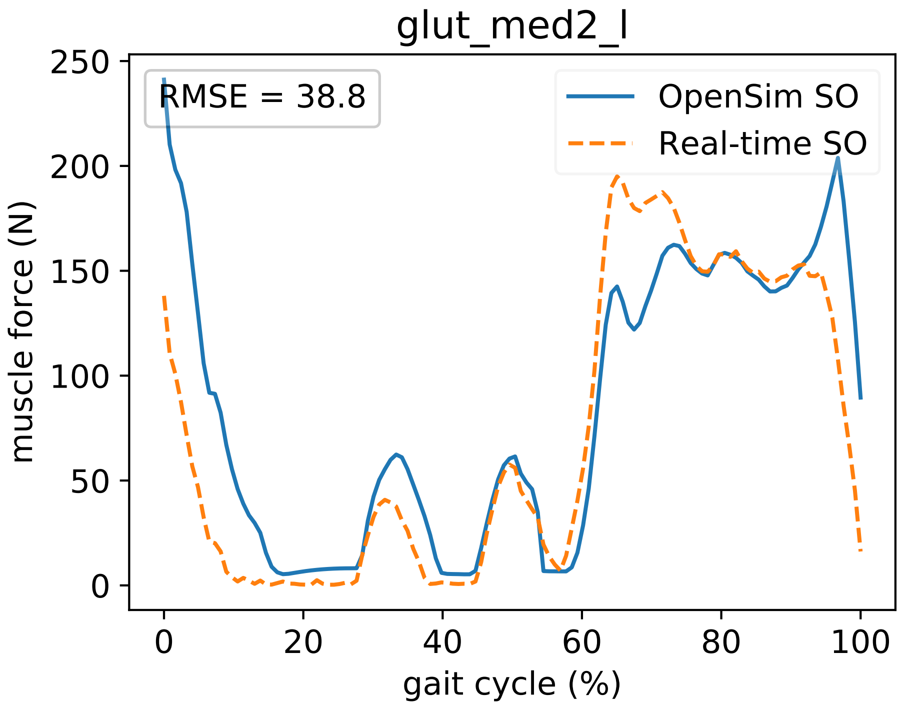
glut_med2_l
RMSE = 38.8
OpenSim SO
Real-time SO
muscle force (N)
gait cycle (%)
0
50
100
150
200
250
0
20
40
60
80
100

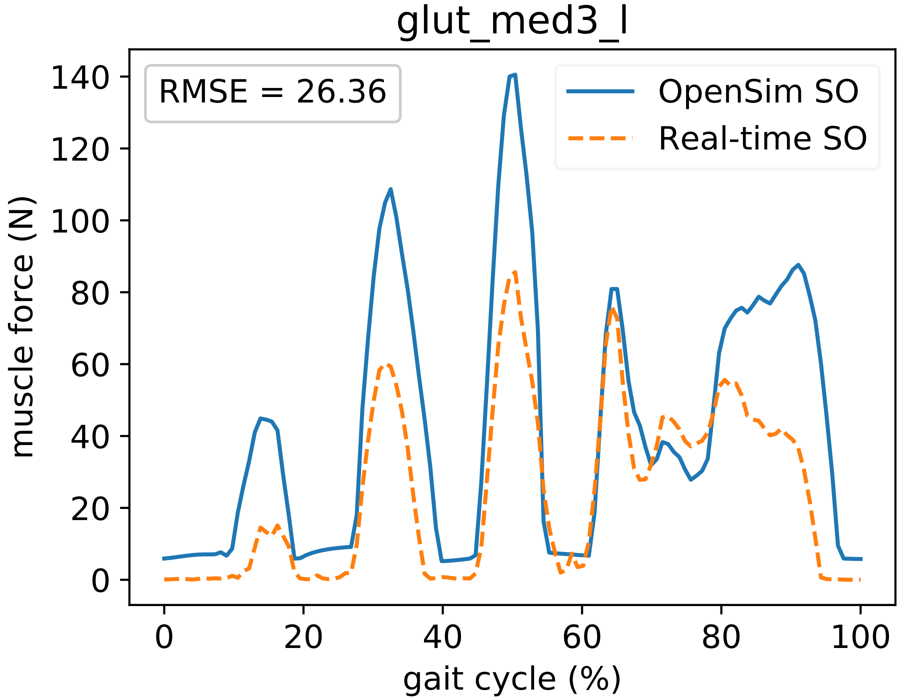

glut_med3_l
RMSE = 26.36
OpenSim SO
Real-time SO
muscle force (N)
gait cycle (%)
0
20
40
60
80
100
120
140
0
20
40
60
80
100

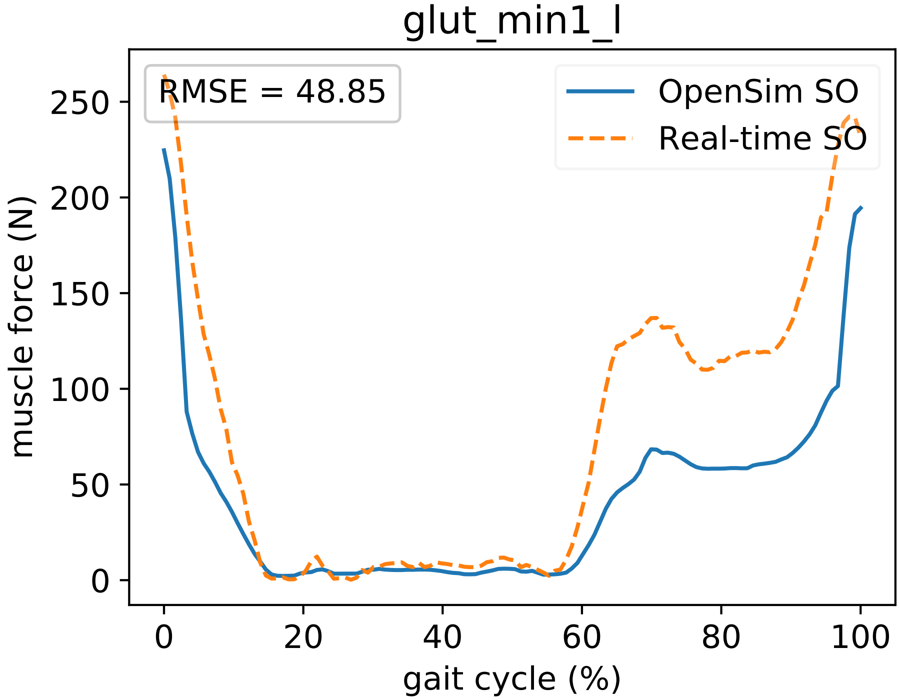
glut_min1_l
RMSE = 48.85
OpenSim SO
Real-time SO
muscle force (N)
gait cycle (%)
0
50
100
150
200
250
0
20
40
60
80
100

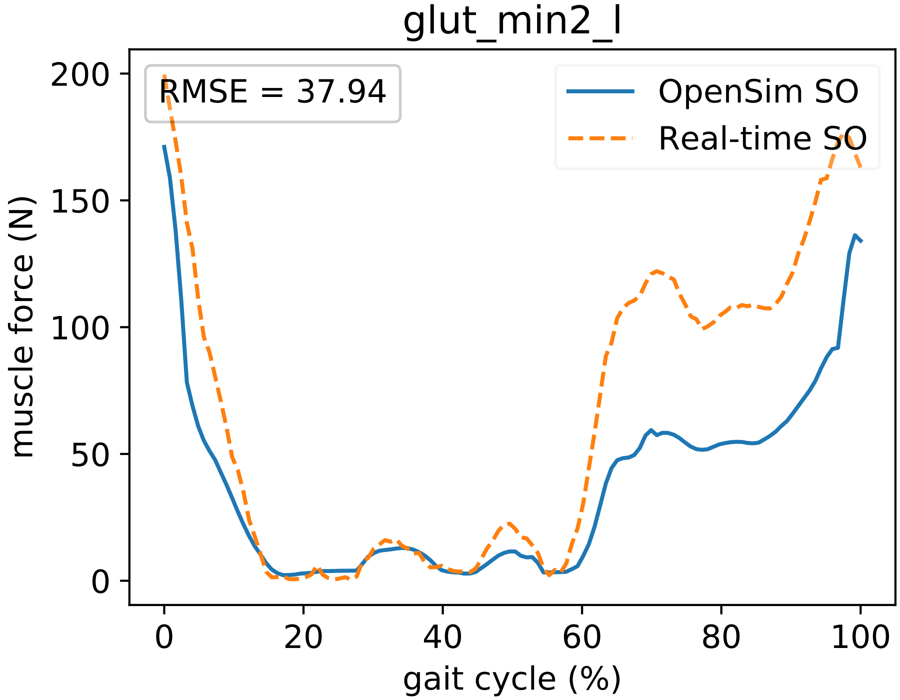

glut_min2_l
RMSE = 37.94
OpenSim SO
Real-time SO
muscle force (N)
gait cycle (%)
0
50
100
150
200
0
20
40
60
80
100

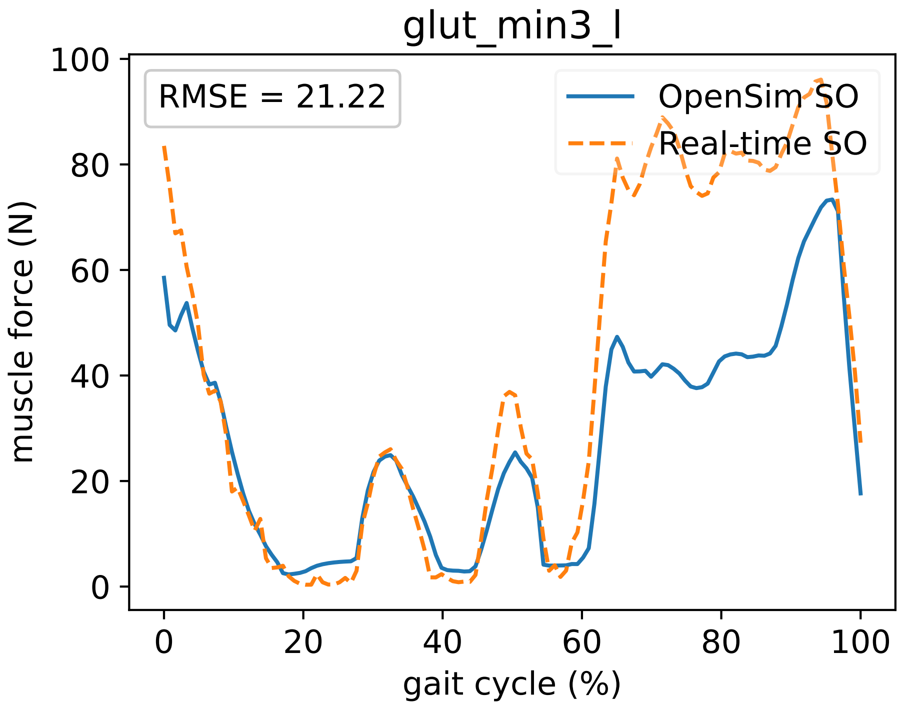
glut_min3_l
RMSE = 21.22
OpenSim SO
Real-time SO
muscle force (N)
gait cycle (%)
0
20
40
60
80
100
0
20
40
60
80
100

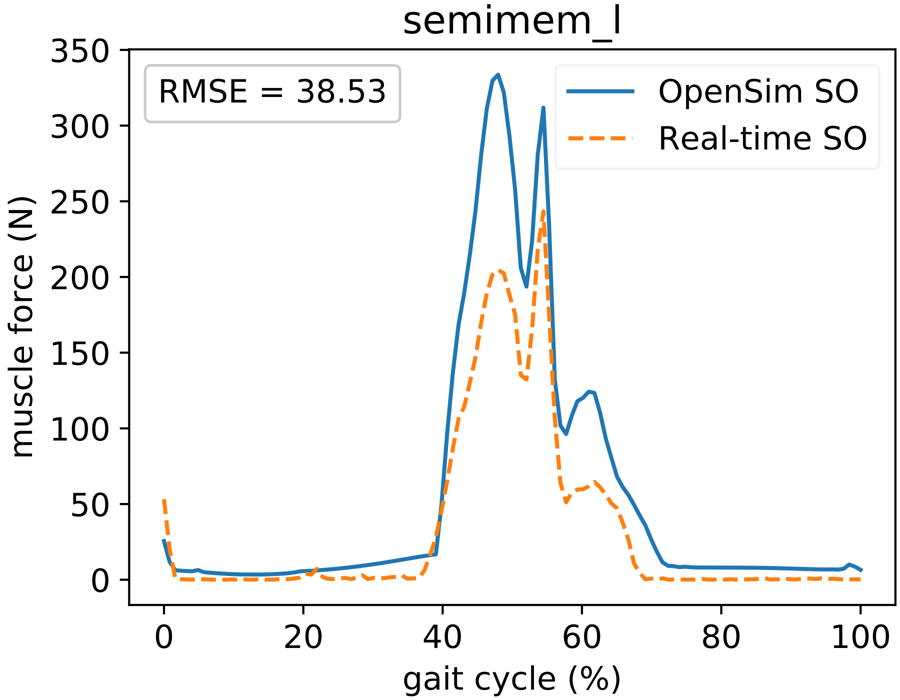

semimem_l
RMSE = 38.53
OpenSim SO
Real-time SO
muscle force (N)
350
300
250
200
150
100
50
0
0
20
40
60
80
100
gait cycle (%)

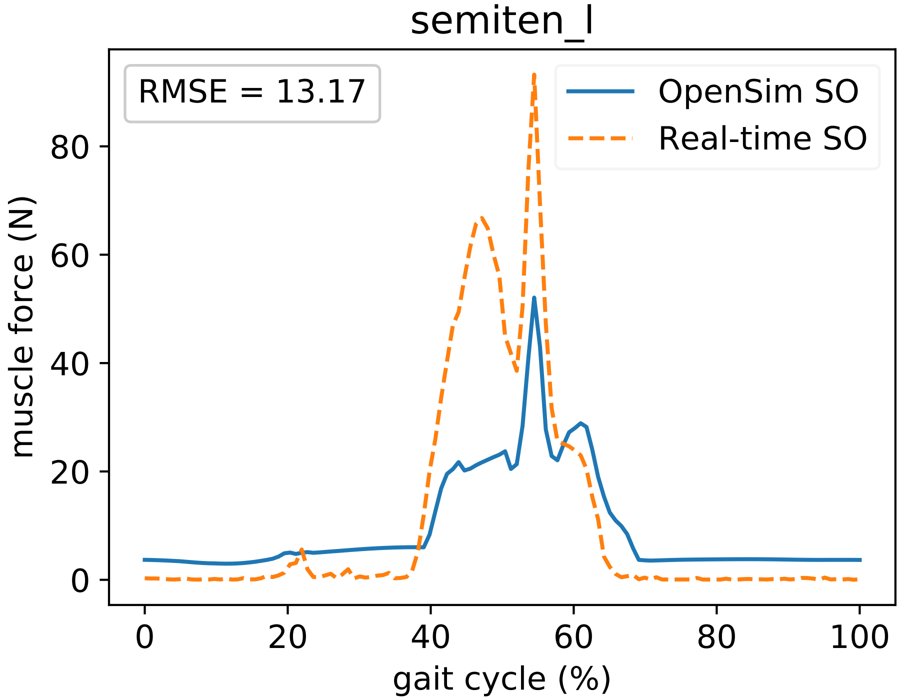

semiten_l
RMSE = 13.17
OpenSim SO
Real-time SO
muscle force (N)
0
20
40
60
80
0
20
40
60
80
100
gait cycle (%)

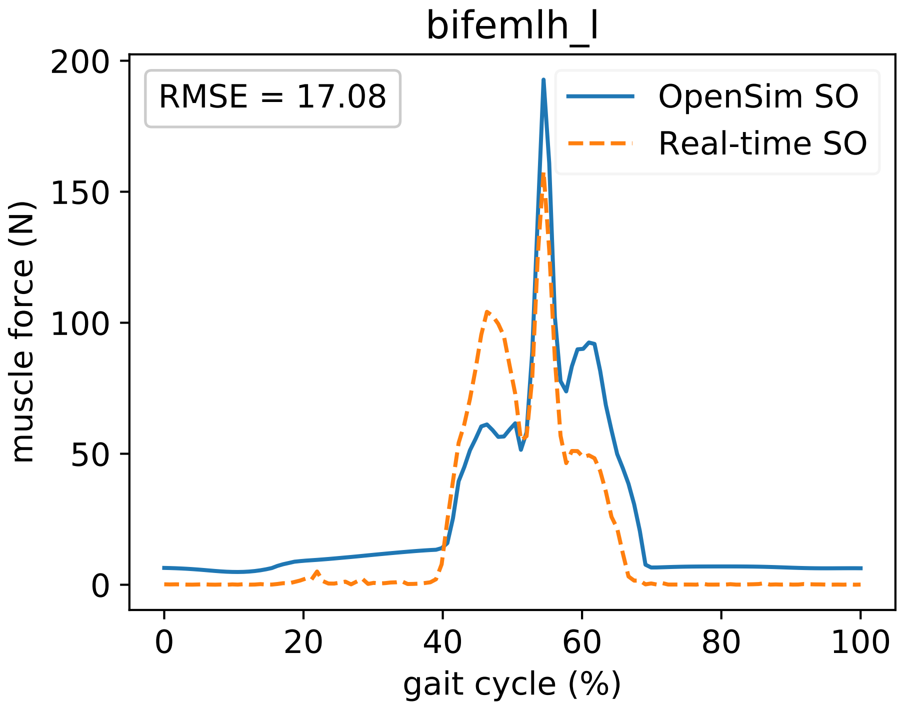
bifemlh_l
RMSE = 17.08
OpenSim SO
Real-time SO
muscle force (N)
gait cycle (%)
0
50
100
150
200
0
20
40
60
80
100

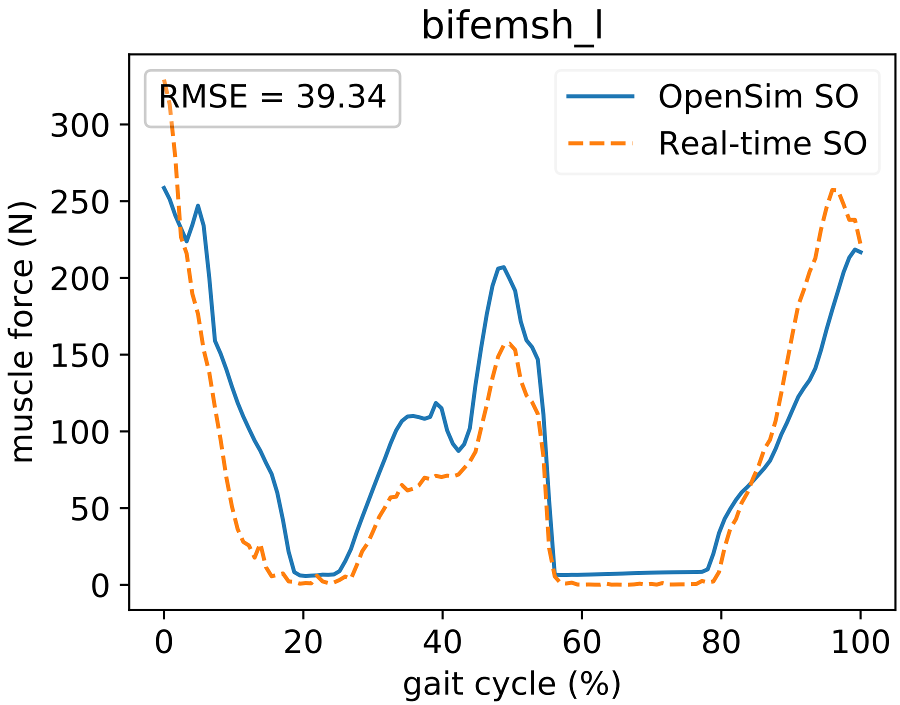

bifemsh_l
RMSE = 39.34
OpenSim SO
Real-time SO
muscle force (N)
0
50
100
150
200
250
300
0
20
40
60
80
100
gait cycle (%)

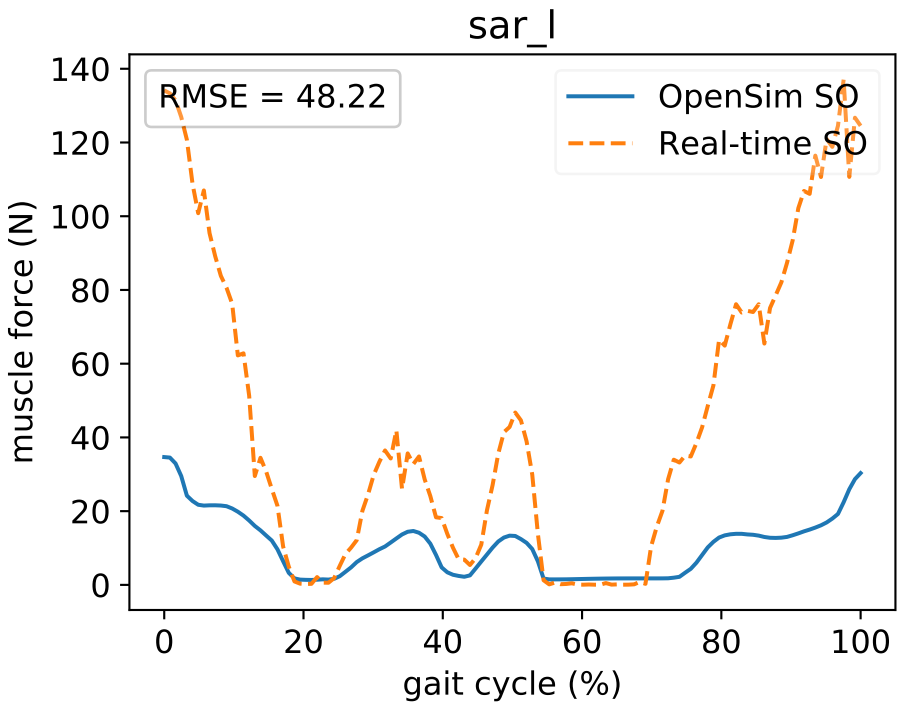

sar_l
RMSE = 48.22
OpenSim SO
Real-time SO
muscle force (N)
gait cycle (%)
0
20
40
60
80
100
120
140

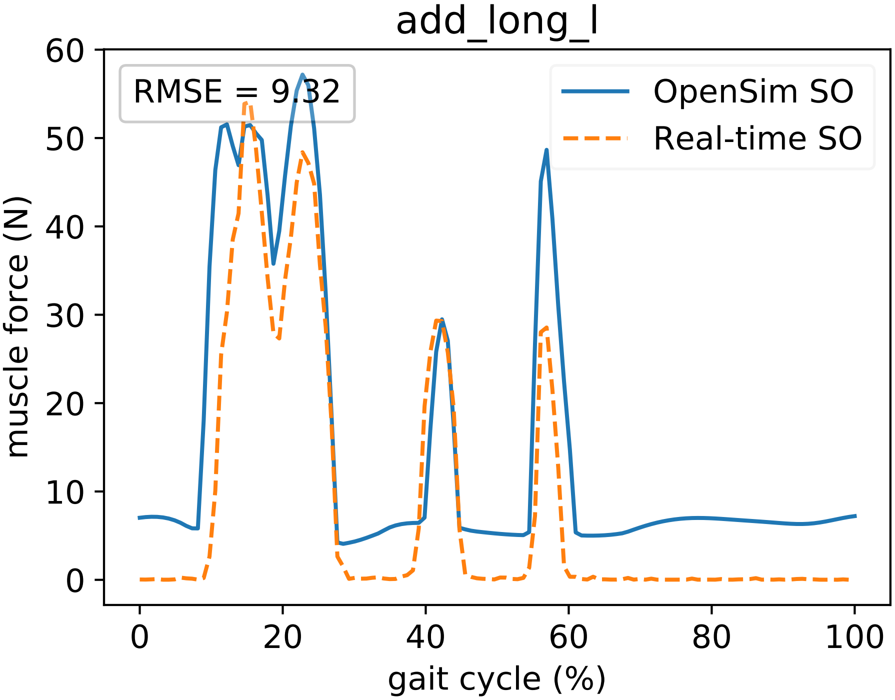
add_long_l
RMSE = 9.32
OpenSim SO
Real-time SO
muscle force (N)
gait cycle (%)
0
10
20
30
40
50
60
0
20
40
60
80
100

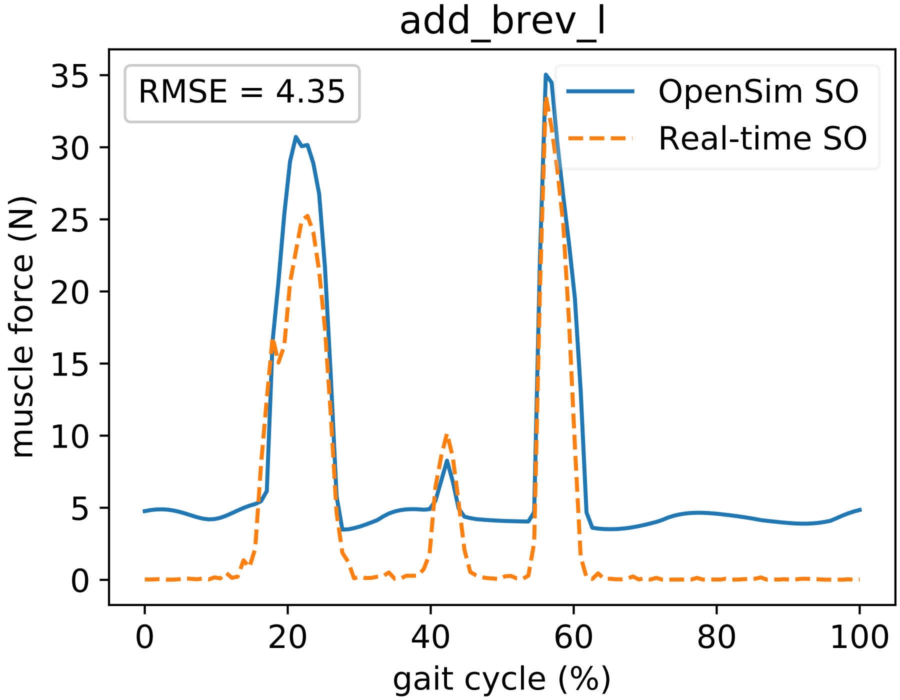

add_brev_l
RMSE = 4.35
OpenSim SO
Real-time SO
muscle force (N)
gait cycle (%)
0
5
10
15
20
25
30
35
0
20
40
60
80
100

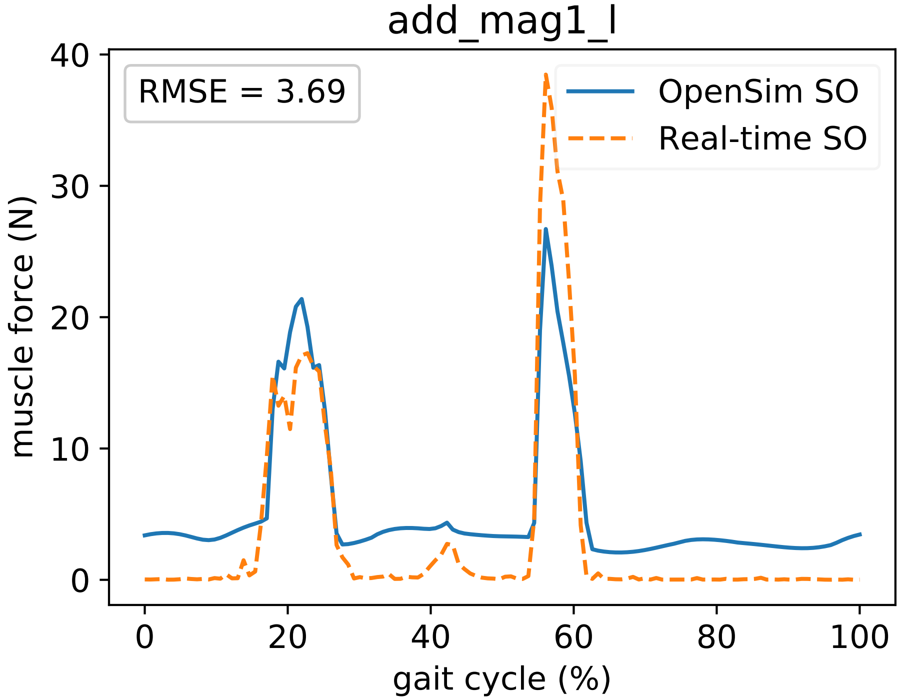
add_mag1_l
RMSE = 3.69
OpenSim SO
Real-time SO
muscle force (N)
gait cycle (%)
0
10
20
30
40
0
20
40
60
80
100

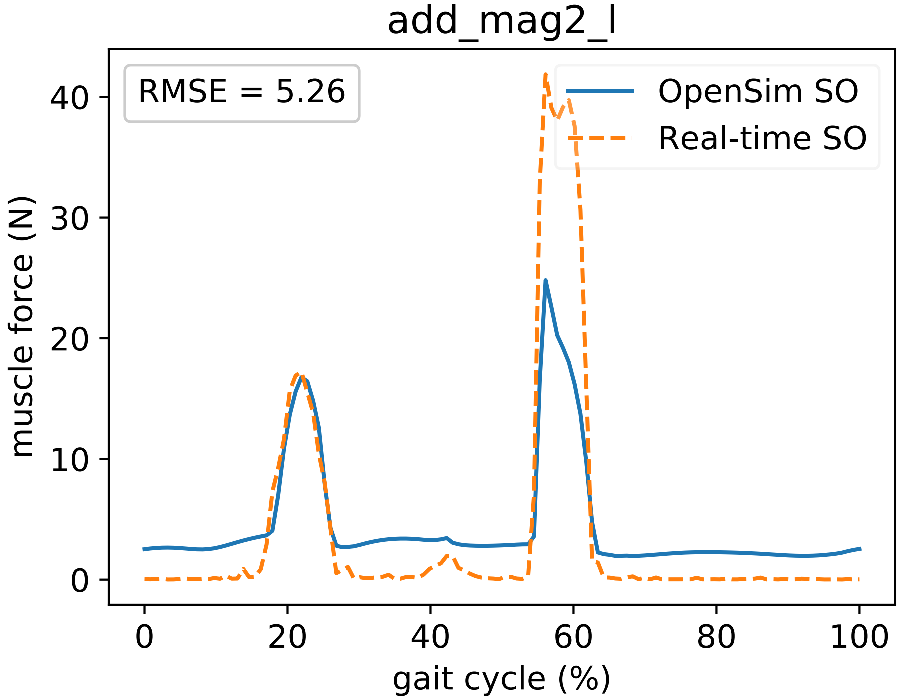

add_mag2_l
RMSE = 5.26
OpenSim SO
Real-time SO
muscle force (N)
gait cycle (%)
0
10
20
30
40
0
20
40
60
80
100

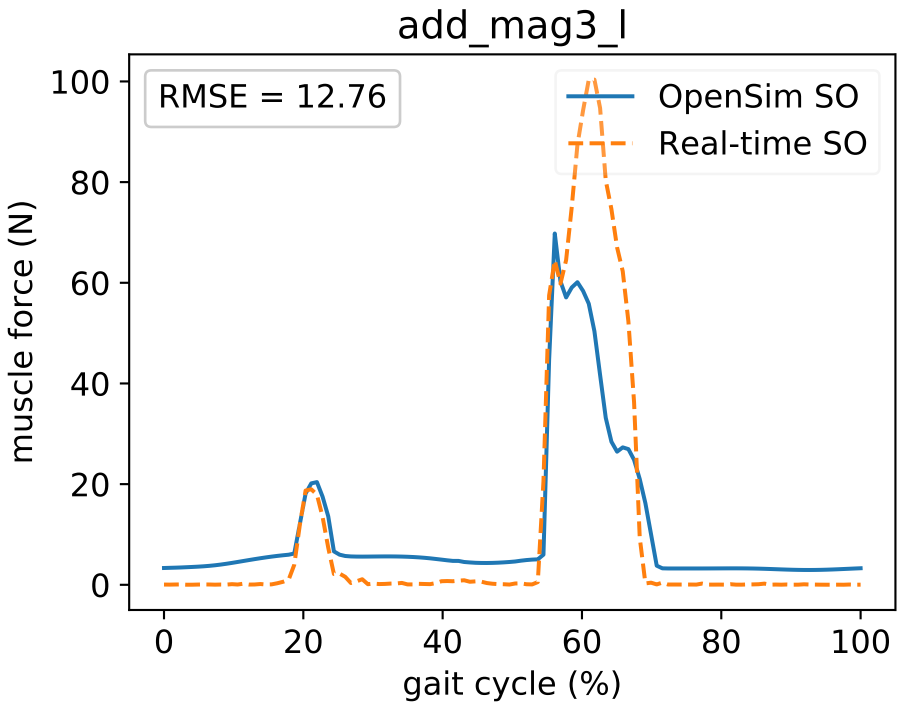

add_mag3_l
RMSE = 12.76
OpenSim SO
Real-time SO
muscle force (N)
gait cycle (%)
0
20
40
60
80
100

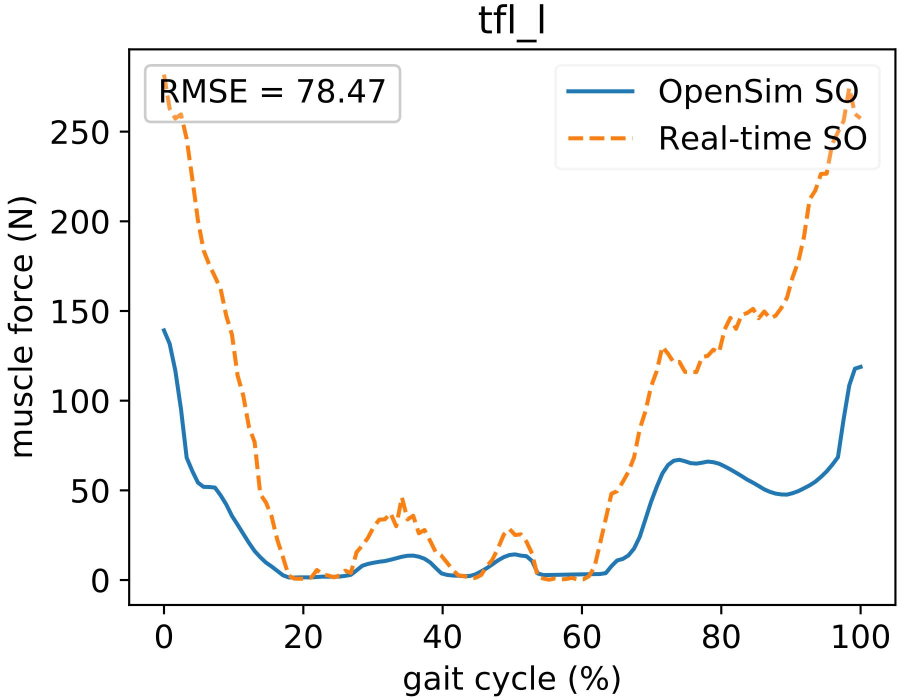
tfl_l
RMSE = 78.47
OpenSim SO
Real-time SO
muscle force (N)
0
50
100
150
200
250
0
20
40
60
80
100
gait cycle (%)

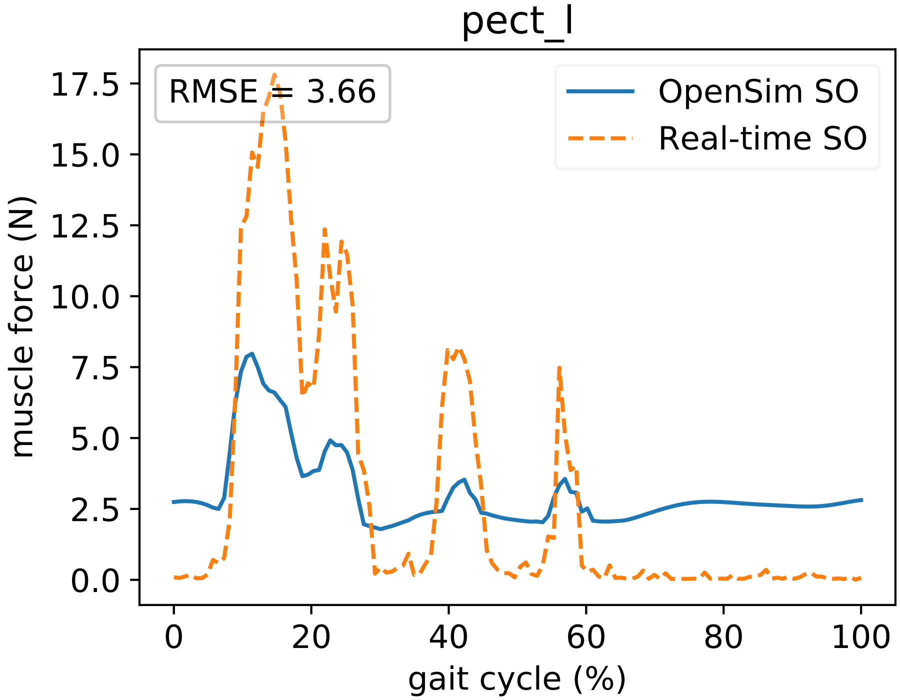
pect_l
RMSE = 3.66
OpenSim SO
Real-time SO
muscle force (N)
gait cycle (%)
0.0
2.5
5.0
7.5
10.0
12.5
15.0
17.5
0
20
40
60
80
100

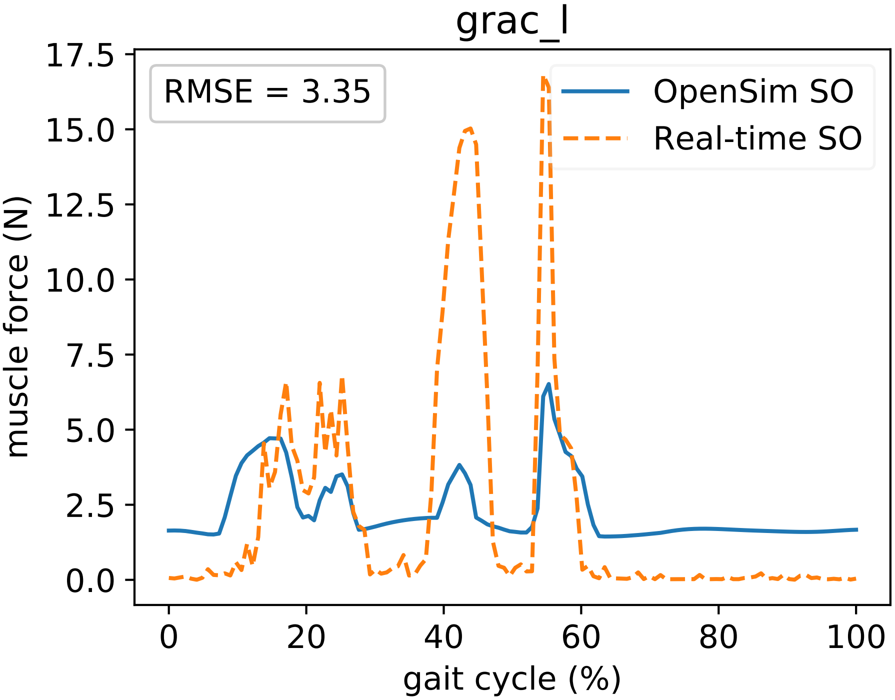
grac_l
RMSE = 3.35
OpenSim SO
Real-time SO
muscle force (N)
gait cycle (%)
0.0
2.5
5.0
7.5
10.0
12.5
15.0
17.5
0
20
40
60
80
100

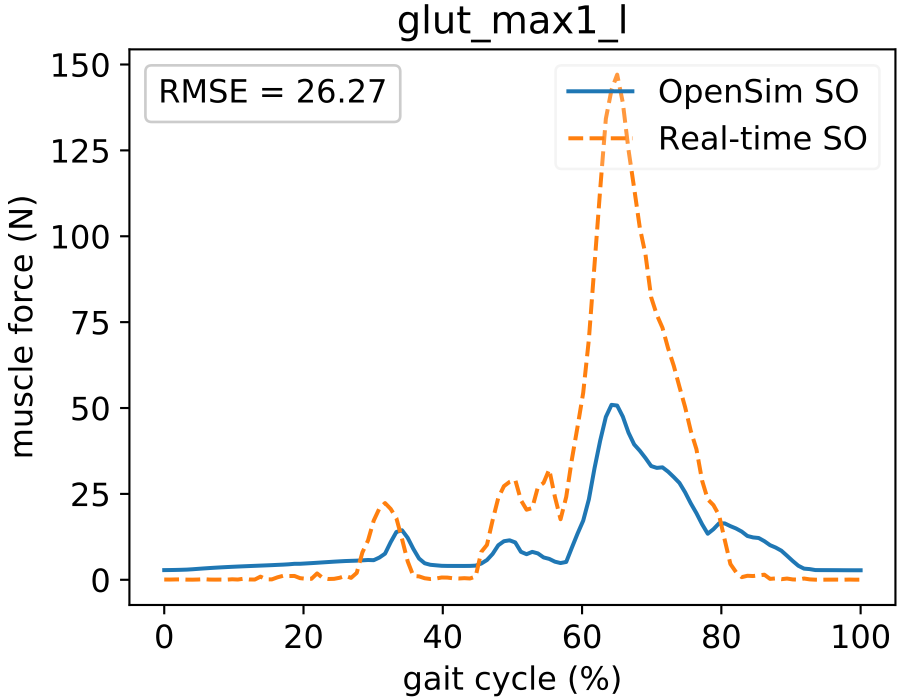

glut_max1_l
RMSE = 26.27
OpenSim SO
Real-time SO
muscle force (N)
gait cycle (%)
0
25
50
75
100
125
150
0
20
40
60
80
100

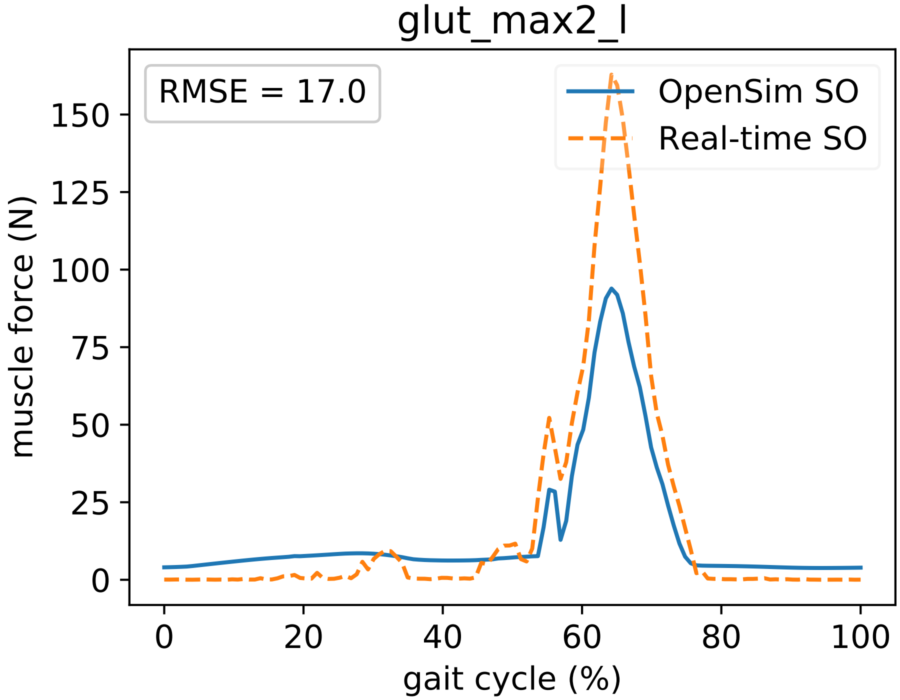

glut_max2_l
RMSE = 17.0
OpenSim SO
Real-time SO
muscle force (N)
gait cycle (%)
0
25
50
75
100
125
150
0
20
40
60
80
100

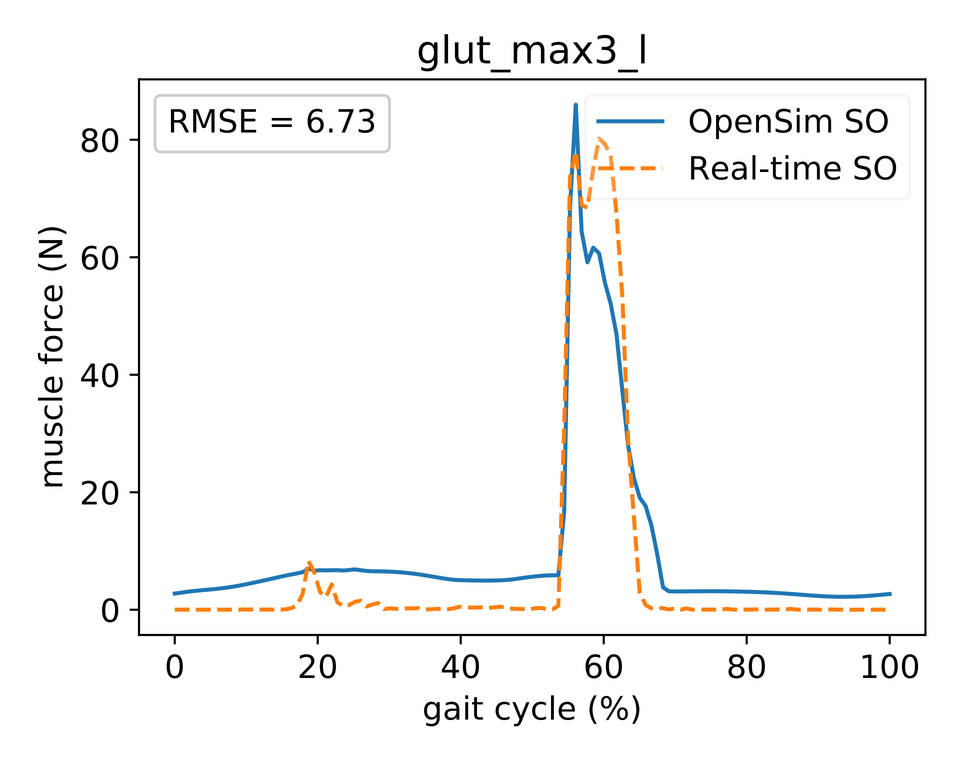

glut_max3_l
RMSE = 6.73
OpenSim SO
Real-time SO
muscle force (N)
gait cycle (%)
0
20
40
60
80
100

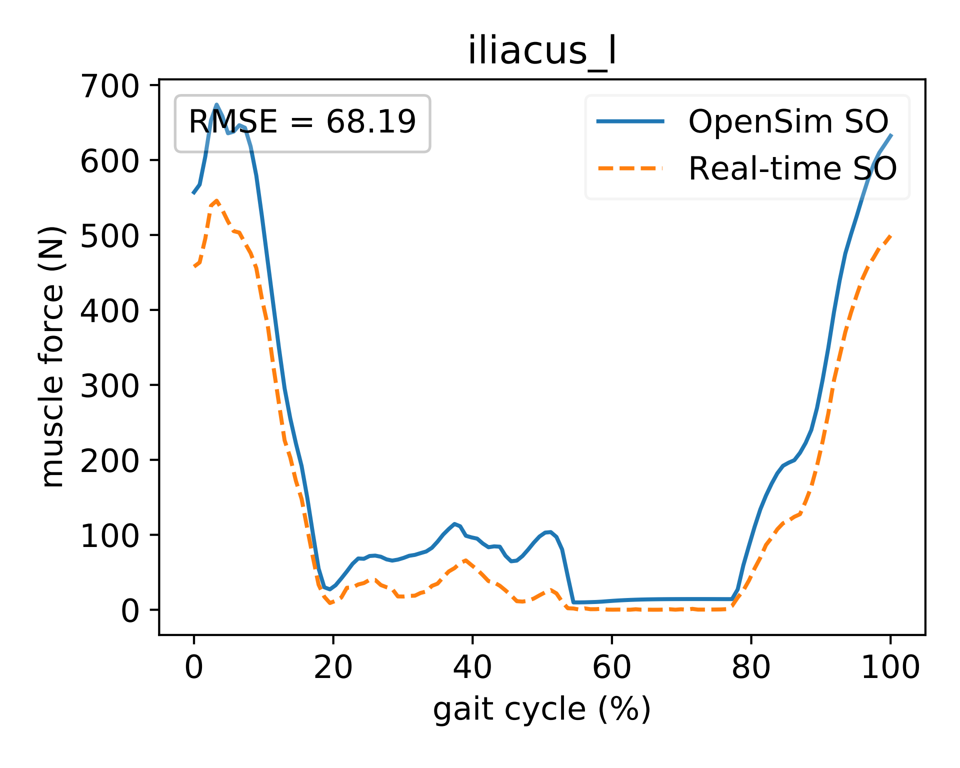

iliacus_l
RMSE = 68.19
OpenSim SO
Real-time SO
muscle force (N)
gait cycle (%)
0
100
200
300
400
500
600
700
0
20
40
60
80
100

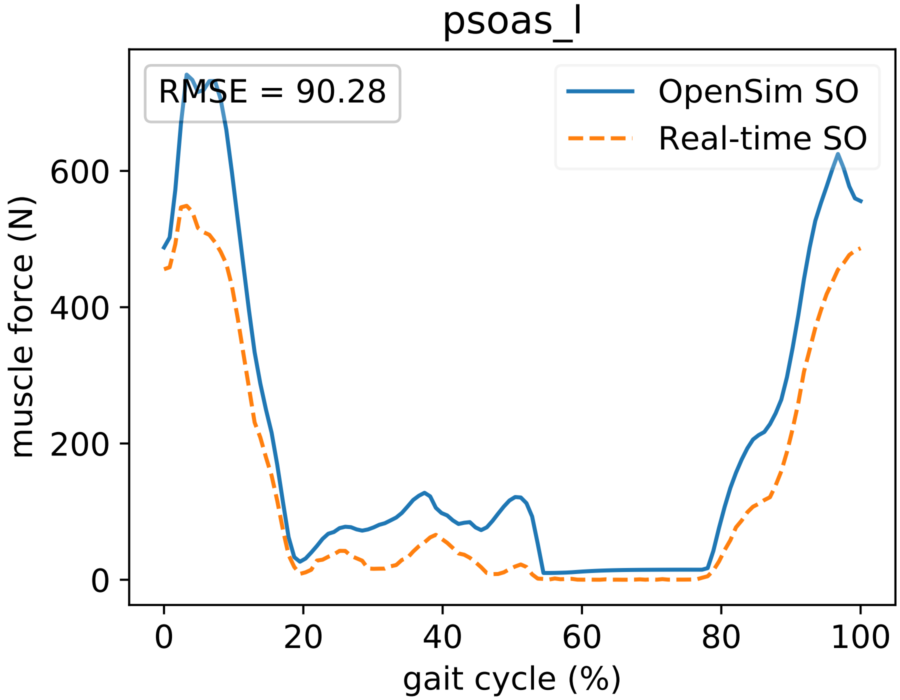
psoas_l
RMSE = 90.28
OpenSim SO
Real-time SO
muscle force (N)
0
200
400
600
0
20
40
60
80
100
gait cycle (%)

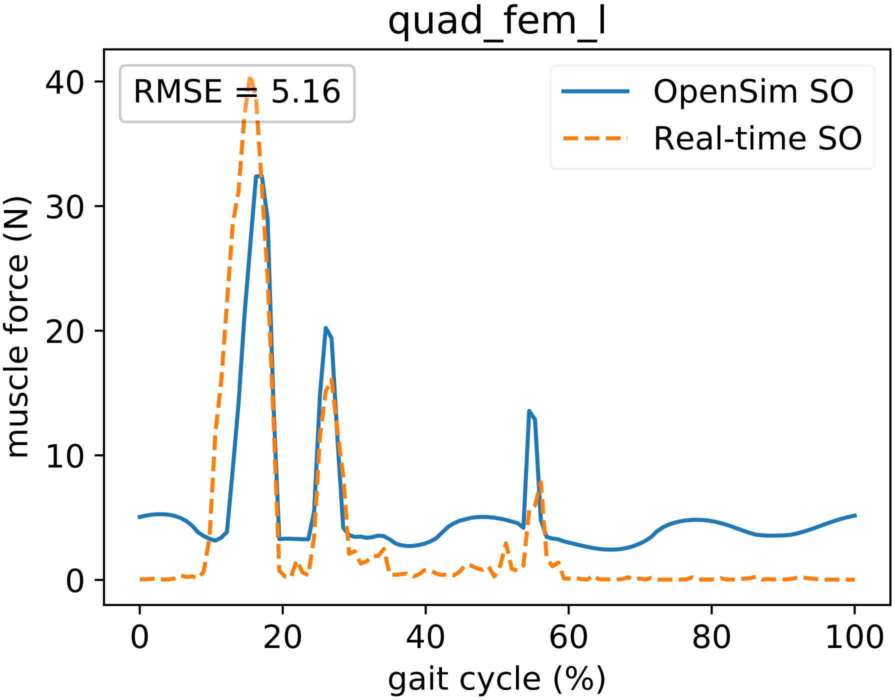
quad_fem_l
RMSE = 5.16
OpenSim SO
Real-time SO
muscle force (N)
gait cycle (%)
0
10
20
30
40
0
20
40
60
80
100

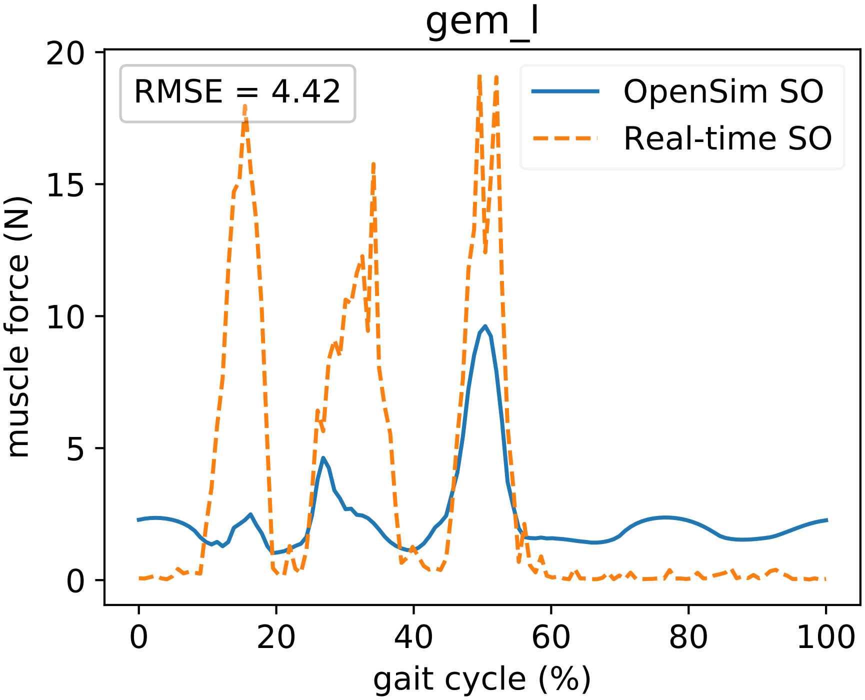

gem_l
RMSE = 4.42
OpenSim SO
Real-time SO
muscle force (N)
gait cycle (%)
0
5
10
15
20
0
20
40
60
80
100

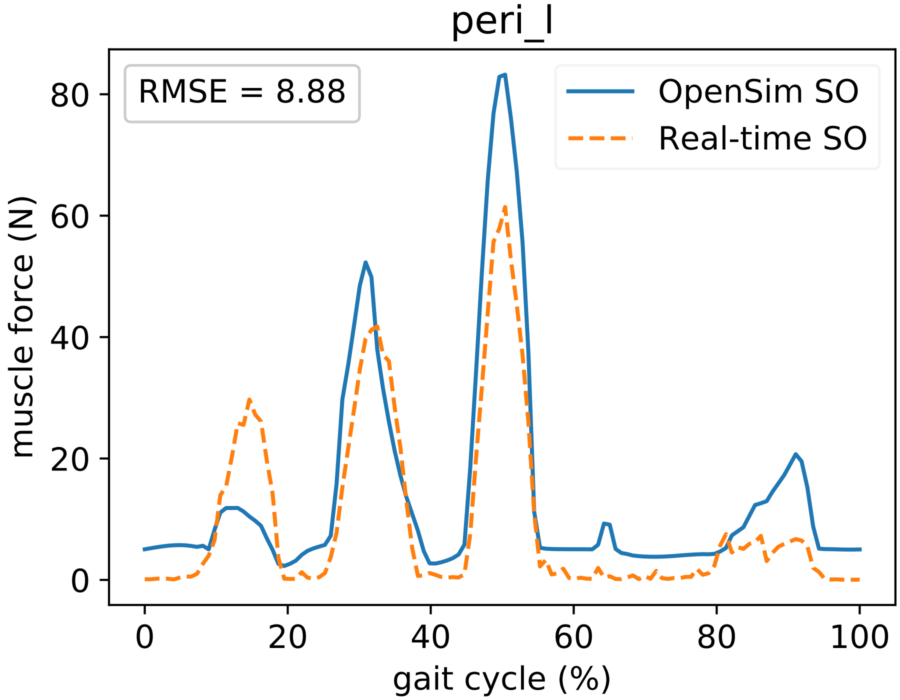

peri_l
RMSE = 8.88
OpenSim SO
Real-time SO
muscle force (N)
gait cycle (%)
0
20
40
60
80
100

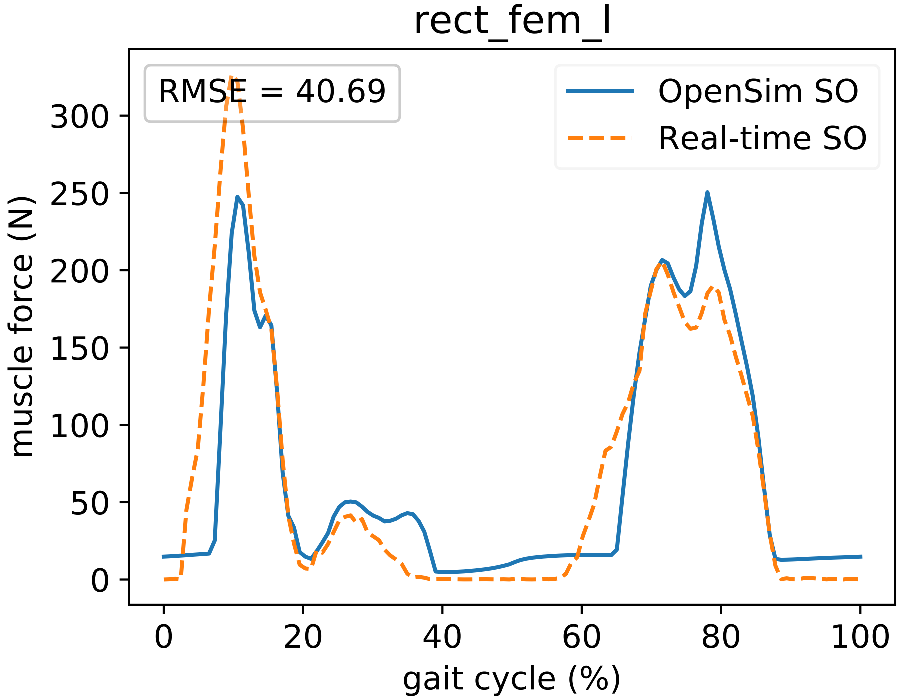
rect_fem_l
RMSE = 40.69
OpenSim SO
Real-time SO
muscle force (N)
0
50
100
150
200
250
300
0
20
40
60
80
100
gait cycle (%)

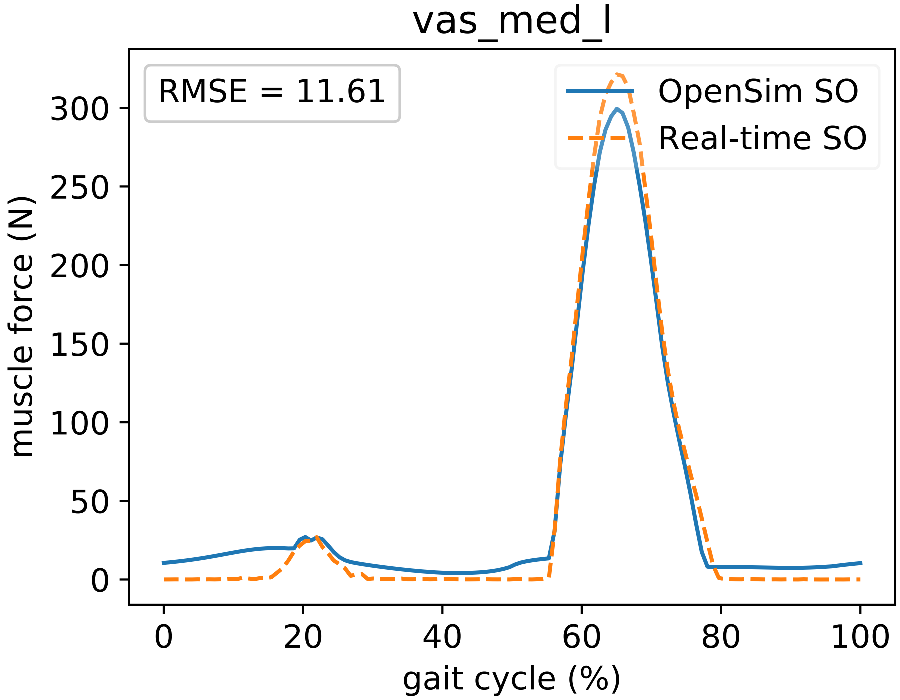

vas_med_l
RMSE = 11.61
OpenSim SO
Real-time SO
muscle force (N)
gait cycle (%)
0
50
100
150
200
250
300
0
20
40
60
80
100

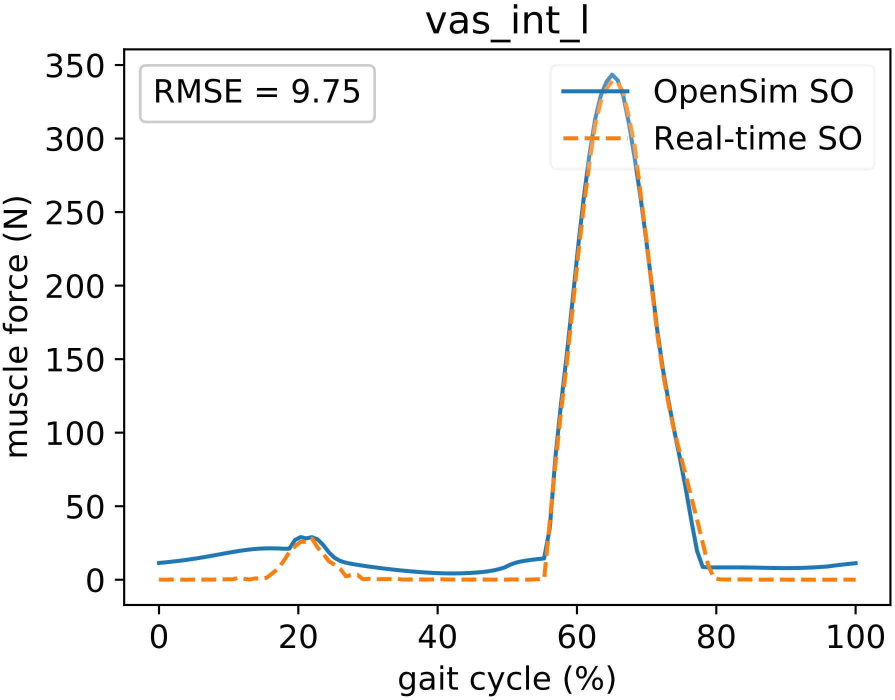

vas_int_l
RMSE = 9.75
OpenSim SO
Real-time SO
muscle force (N)
gait cycle (%)
0
50
100
150
200
250
300
350
0
20
40
60
80
100

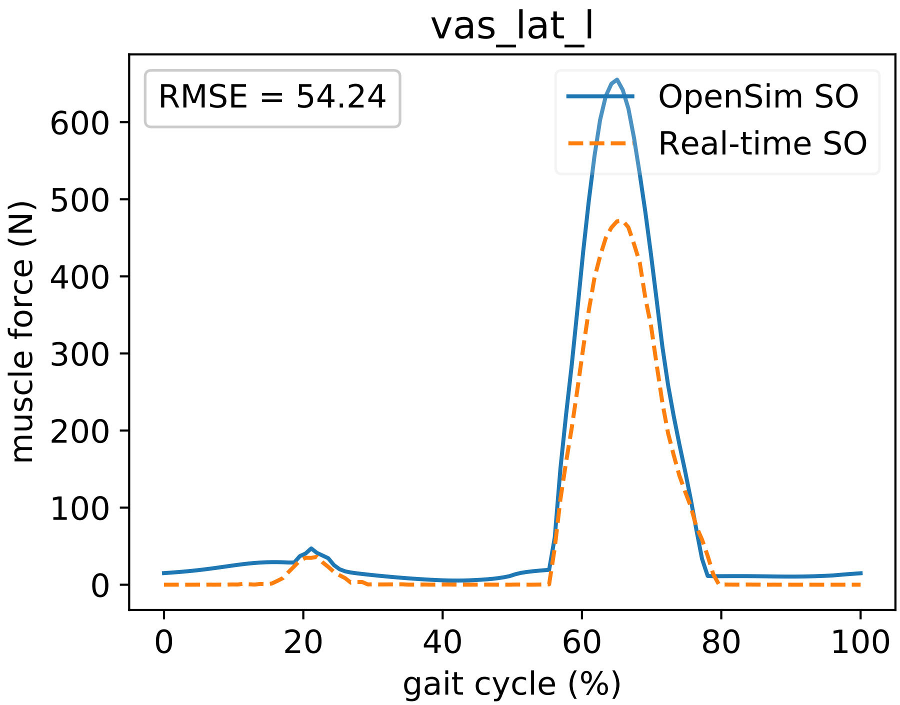
vas_lat_l
RMSE = 54.24
OpenSim SO
Real-time SO
muscle force (N)
0
100
200
300
400
500
600
0
20
40
60
80
100
gait cycle (%)

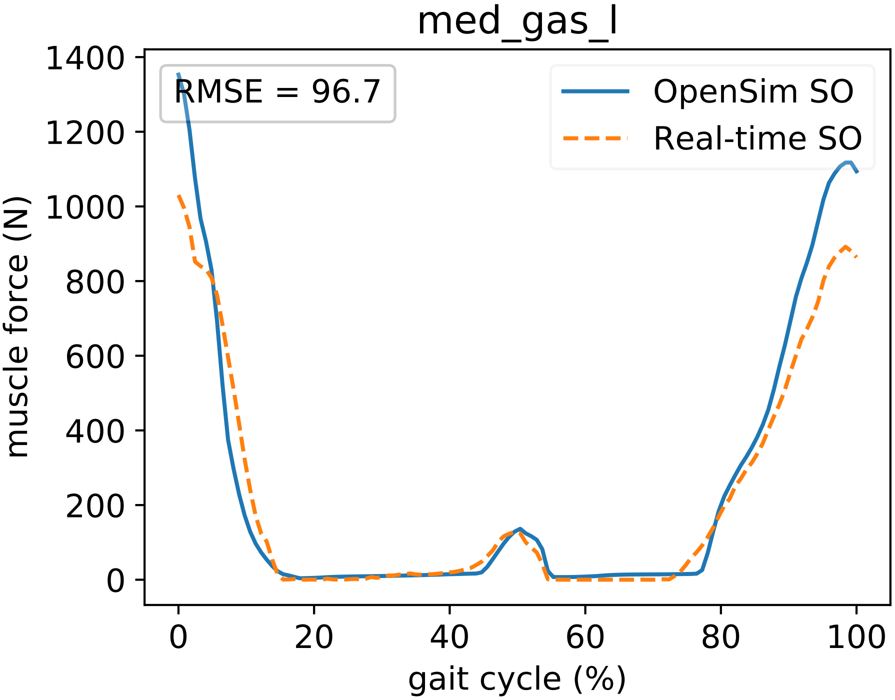
med_gas_l
RMSE = 96.7
OpenSim SO
Real-time SO
muscle force (N)
gait cycle (%)
0
200
400
600
800
1000
1200
1400
0
20
40
60
80
100

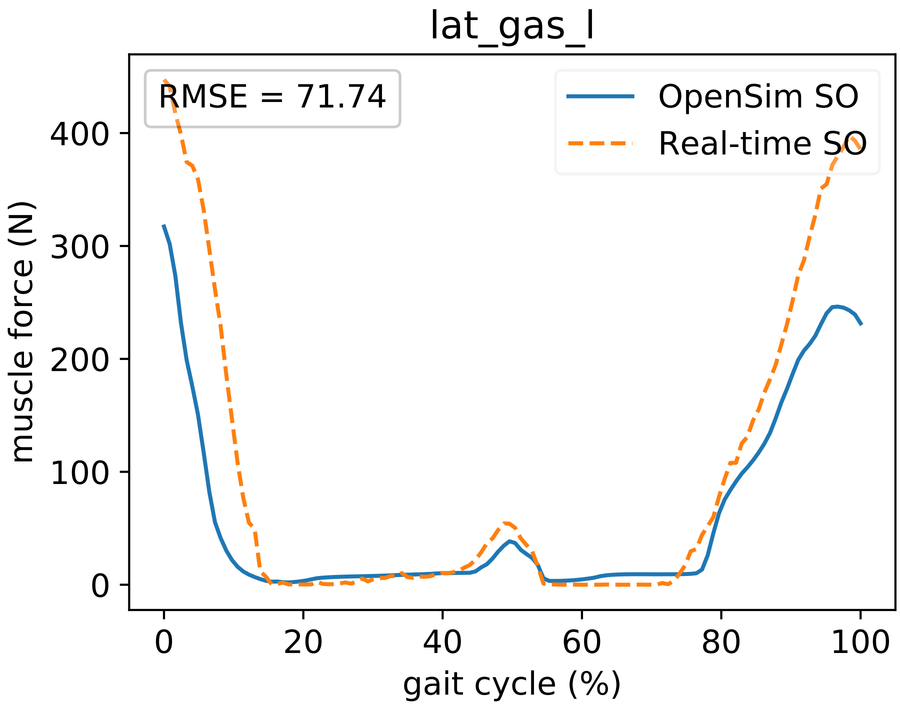
lat_gas_l
RMSE = 71.74
OpenSim SO
Real-time SO
muscle force (N)
0
100
200
300
400
0
20
40
60
80
100
gait cycle (%)

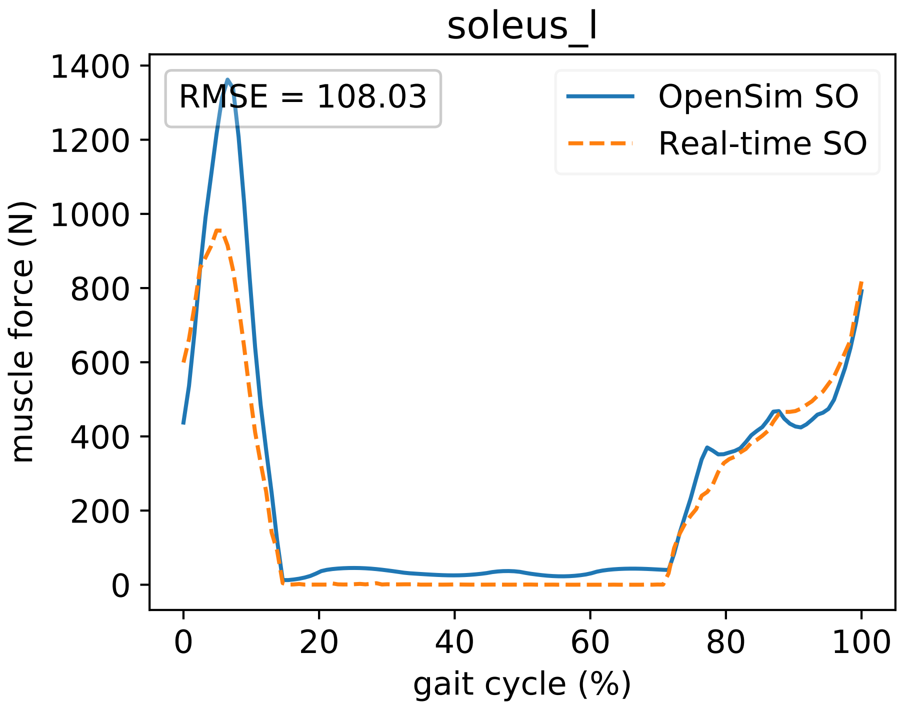

soleus_l
RMSE = 108.03
OpenSim SO
Real-time SO
muscle force (N)
gait cycle (%)
0
200
400
600
800
1000
1200
1400
0
20
40
60
80
100

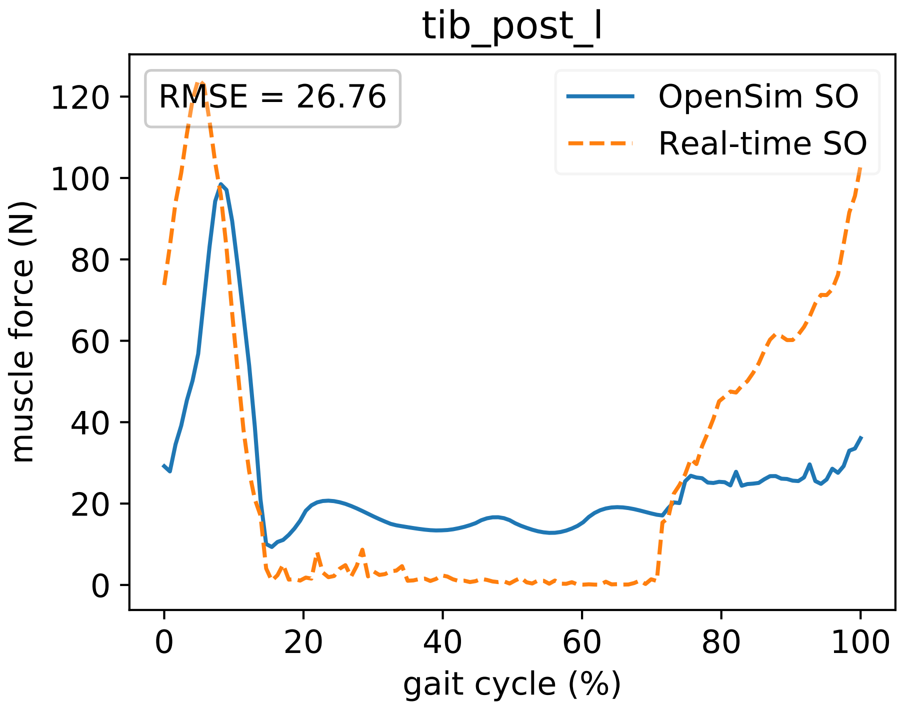
tib_post_l
RMSE = 26.76
OpenSim SO
Real-time SO
muscle force (N)
0
20
40
60
80
100
120
gait cycle (%)
0
20
40
60
80
100

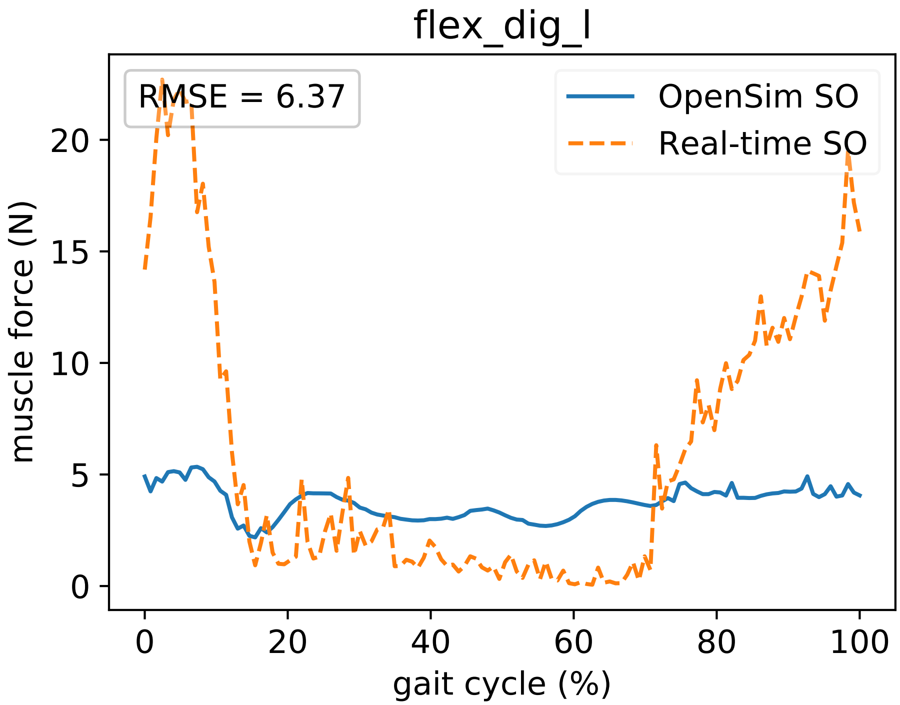

flex_dig_l
RMSE = 6.37
OpenSim SO
Real-time SO
muscle force (N)
0
5
10
15
20
0
20
40
60
80
100
gait cycle (%)

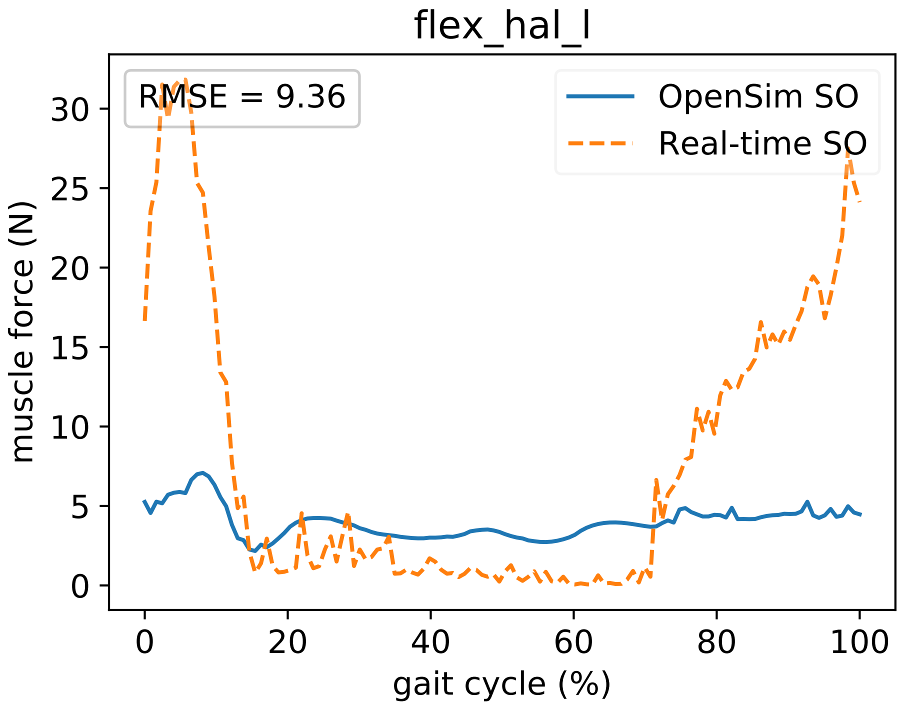

flex_hal_l
RMSE = 9.36
OpenSim SO
Real-time SO
muscle force (N)
gait cycle (%)
0
5
10
15
20
25
30
0
20
40
60
80
100

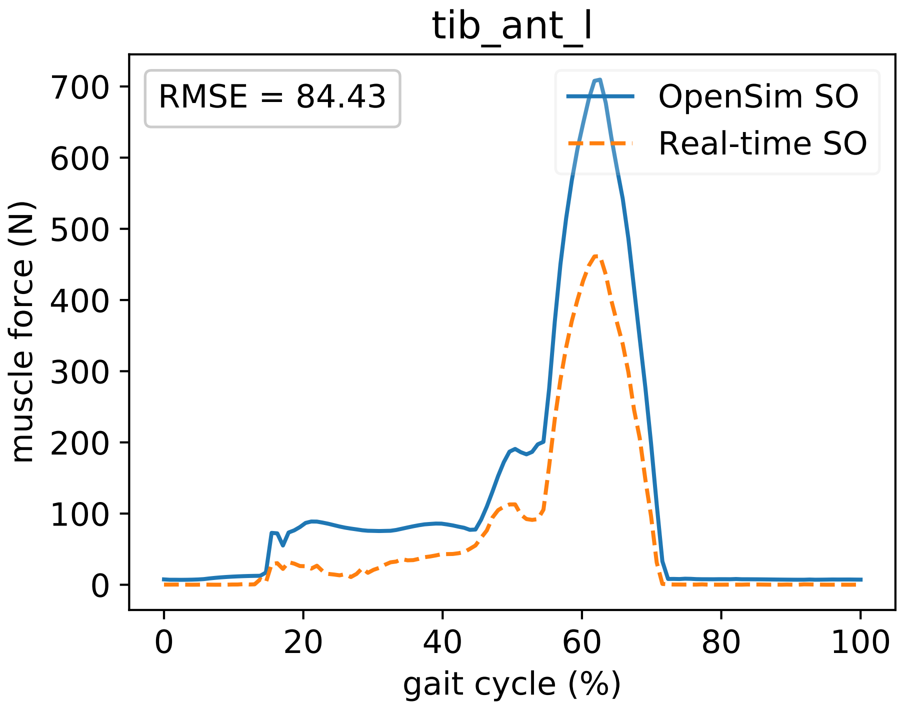
tib_ant_l
RMSE = 84.43
OpenSim SO
Real-time SO
muscle force (N)
700
600
500
400
300
200
100
0
0
20
40
60
80
100
gait cycle (%)

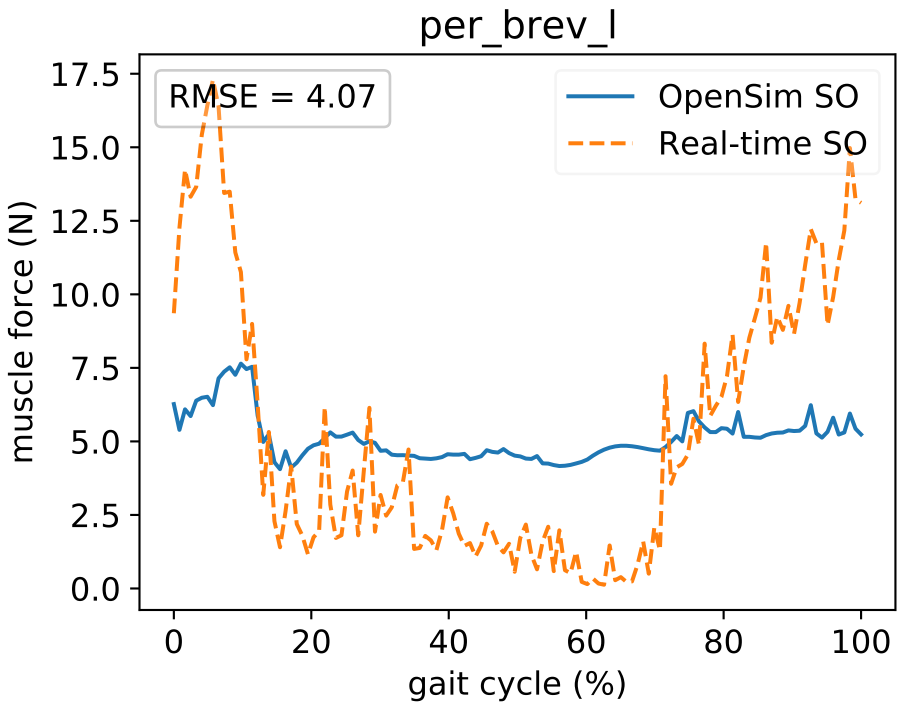
per_brev_l
RMSE = 4.07
OpenSim SO
Real-time SO
muscle force (N)
gait cycle (%)
0.0
2.5
5.0
7.5
10.0
12.5
15.0
17.5
0
20
40
60
80
100

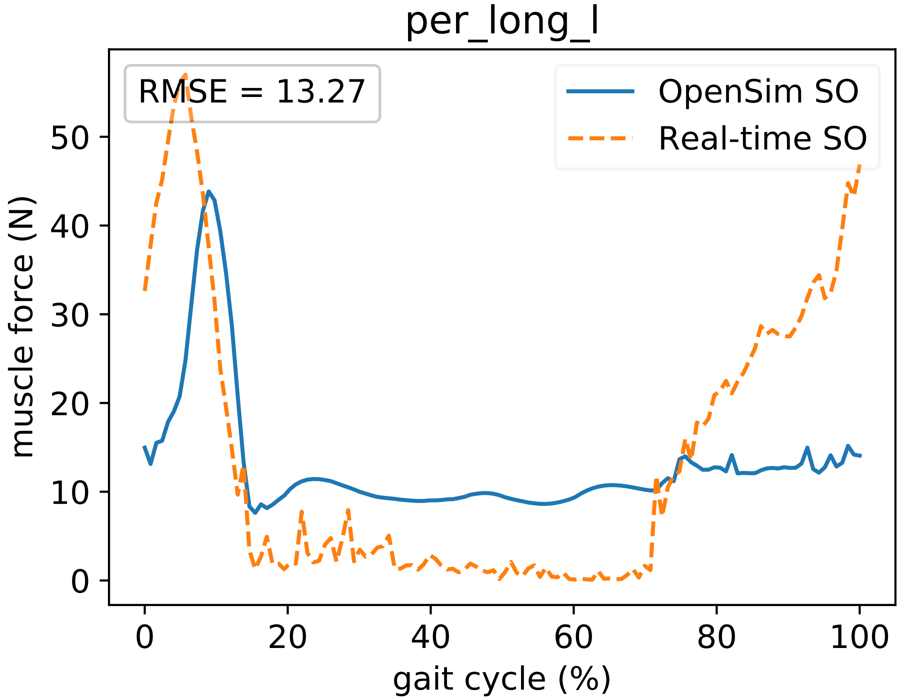

per_long_l
RMSE = 13.27
OpenSim SO
Real-time SO
muscle force (N)
gait cycle (%)
0
10
20
30
40
50
0
20
40
60
80
100

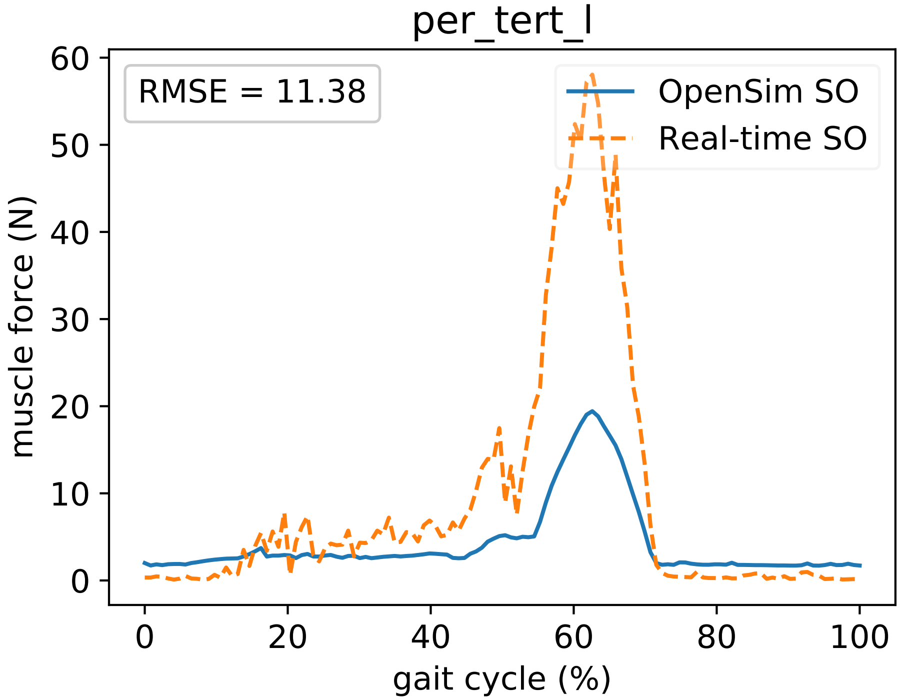

per_tert_l
RMSE = 11.38
OpenSim SO
Real-time SO
muscle force (N)
gait cycle (%)
0
10
20
30
40
50
60
0
20
40
60
80
100

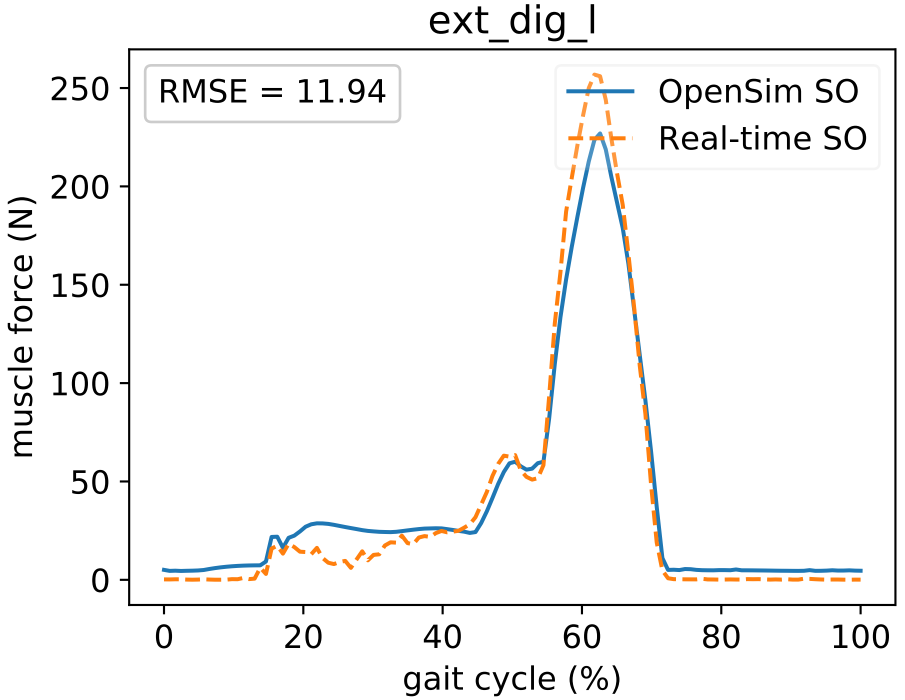

ext_dig_l
RMSE = 11.94
OpenSim SO
Real-time SO
muscle force (N)
gait cycle (%)
0
50
100
150
200
250
0
20
40
60
80
100

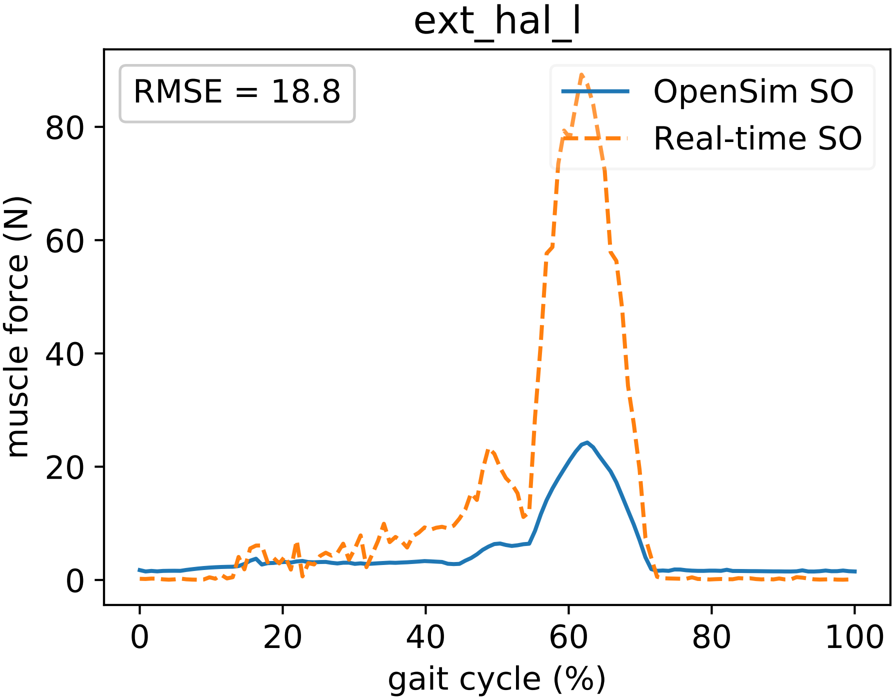

ext_hal_l
RMSE = 18.8
OpenSim SO
Real-time SO
muscle force (N)
0
20
40
60
80
0
20
40
60
80
100
gait cycle (%)

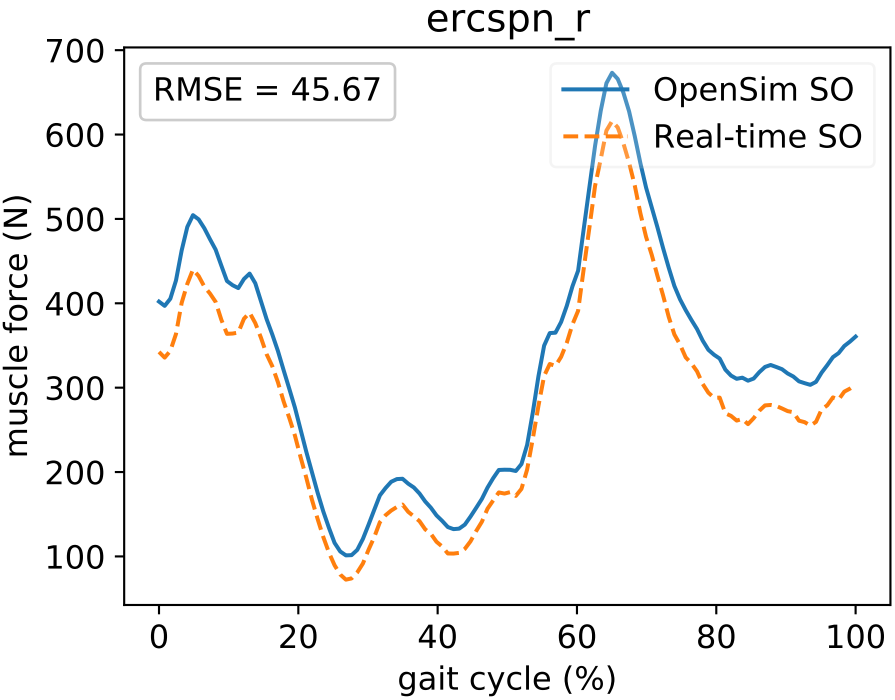
ercspn_r
RMSE = 45.67
OpenSim SO
Real-time SO
muscle force (N)
gait cycle (%)
700
600
500
400
300
200
100
0
20
40
60
80
100

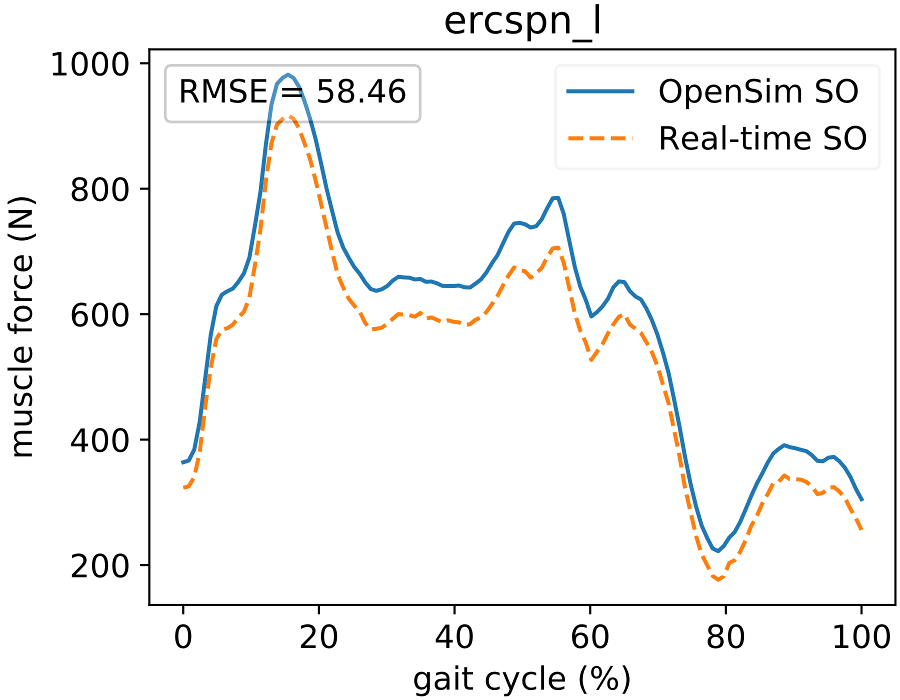

ercspn_l
RMSE = 58.46
OpenSim SO
Real-time SO
muscle force (N)
1000
800
600
400
200
0
20
40
60
80
100
gait cycle (%)

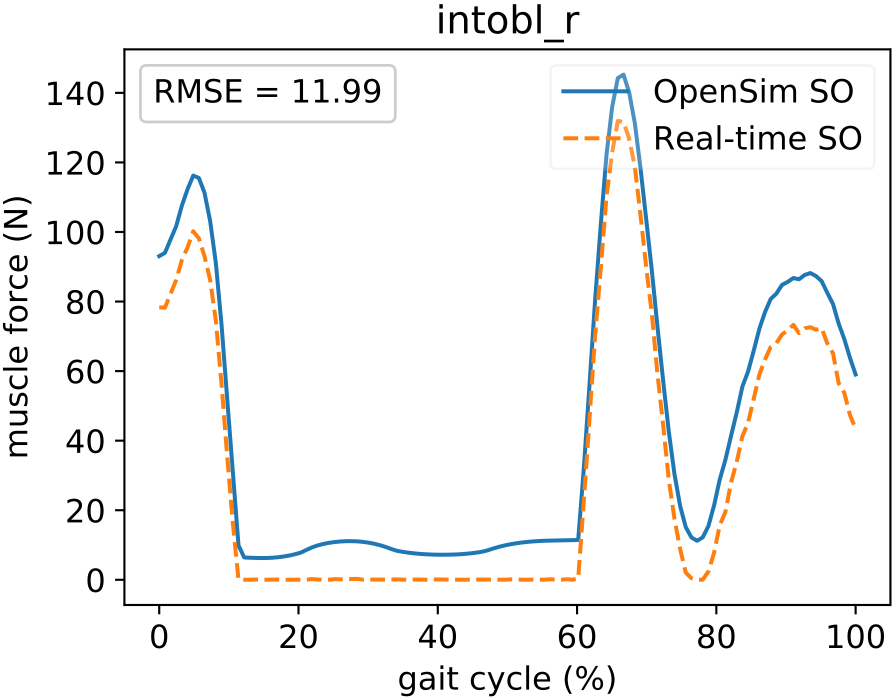

intobl_r
RMSE = 11.99
OpenSim SO
Real-time SO
muscle force (N)
gait cycle (%)
0
20
40
60
80
100
120
140

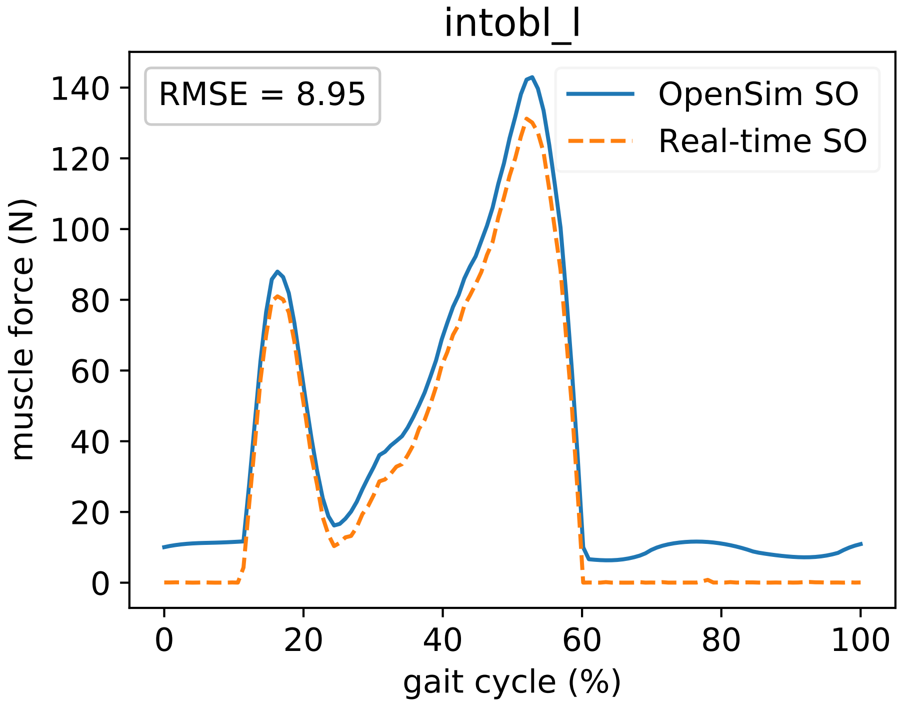

intobl_l
RMSE = 8.95
OpenSim SO
Real-time SO
muscle force (N)
gait cycle (%)
0
20
40
60
80
100
120
140

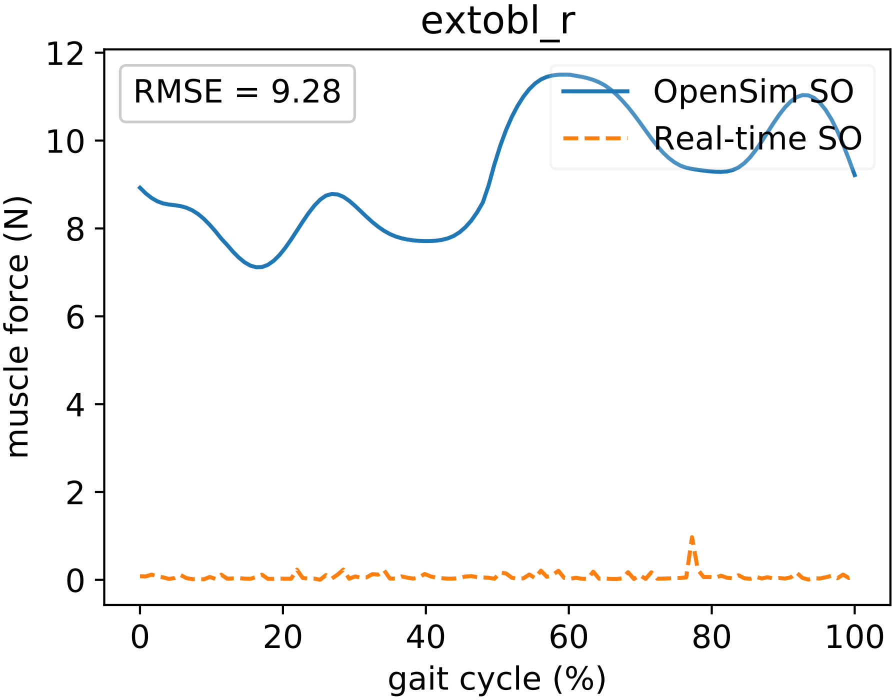

extobl_r
RMSE = 9.28
OpenSim SO
Real-time SO
muscle force (N)
gait cycle (%)
0
2
4
6
8
10
12
0
20
40
60
80
100

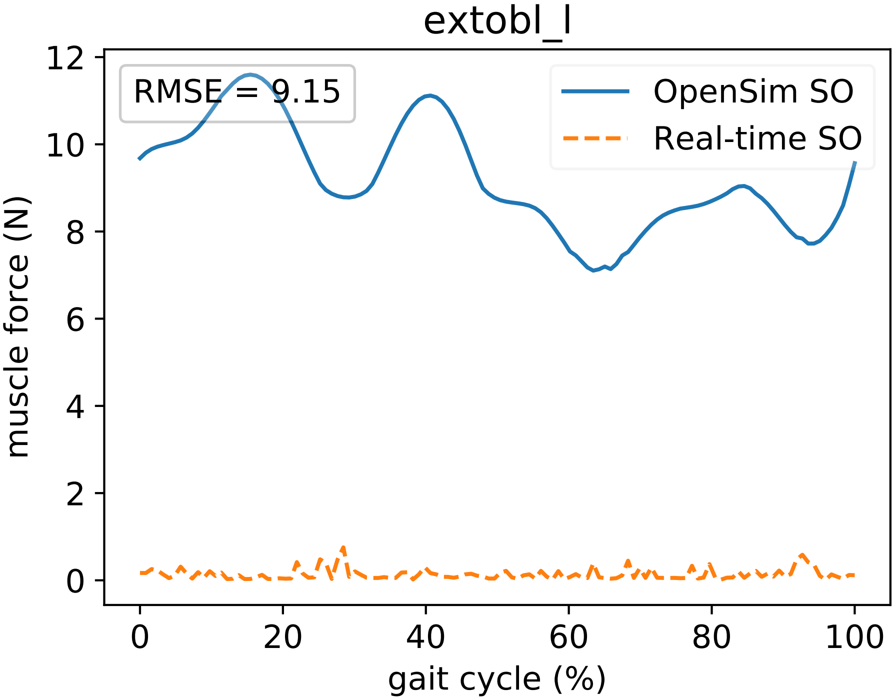
extobl_l
RMSE = 9.15
OpenSim SO
Real-time SO
muscle force (N)
gait cycle (%)
0
2
4
6
8
10
12
0
20
40
60
80
100
